# Ruthenium(ii)-catalyzed chemoselective deacylative annulation of 1,3-diones with sulfoxonium ylides *via* C–C bond activation†
†Electronic supplementary information (ESI) available. CCDC 1918495. For ESI and crystallographic data in CIF or other electronic format see DOI: 10.1039/c9sc03245b


**DOI:** 10.1039/c9sc03245b

**Published:** 2019-08-12

**Authors:** Si Wen, Weiwei Lv, Dan Ba, Jing Liu, Guolin Cheng

**Affiliations:** a College of Materials Science & Engineering , Huaqiao University , Xiamen 361021 , China . Email: glcheng@hqu.edu.cn

## Abstract

The first successful example of deacylative annulation of 1,3-diones with sulfoxonium ylides was achieved through Ru(ii)-catalyzed C–C bond activation. The excellent chemoselectivity and broad substrate scope render this method a practical and versatile approach for the preparation of (hetero)aryl and alkenyl substituted furans, which are valuable units in many biologically active compounds and functional materials. A preliminary mechanistic study reveals that this process involves a deacylative α-ruthenation to generate key alkyl Ru(ii) intermediates with the release of a benzoic acid fragment.

## Abstract 2

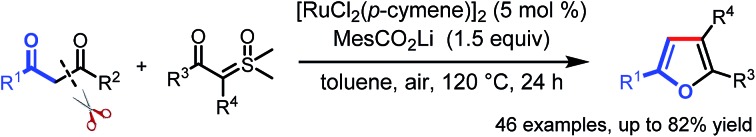
Highly chemoselective Ru(ii)-catalyzed deacylative annulation of 1,3-diones with sulfoxonium ylides was achieved to afford (hetero)aryl substituted furans.

## Introduction

**Scheme 1:**
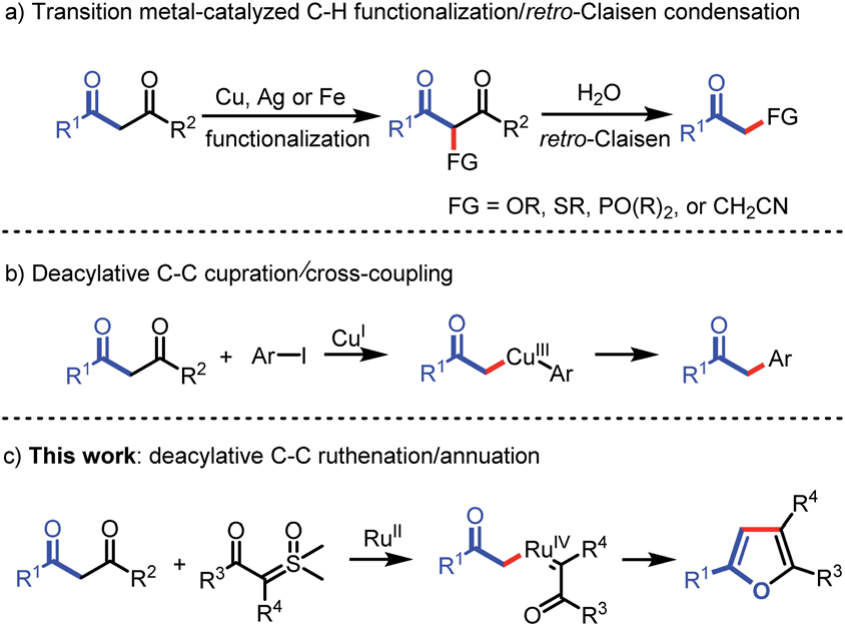
Transition metal-catalyzed C–C bond activation of 1,3-diones.

Carbon–carbon bonds are the most extensive and basic chemical bonds in organic molecules. The selective cleavage of C–C bonds in a constructive manner enables the reconstitution of the molecular skeleton and introduction of new functional groups, thus attracting great attention.^1^ In the past decade, chemoselective C–C(CO) bond cleavage has been extensively studied by employing the strategies of chelation assistance,^2^ ring-strain release,^3^ and aromatization.^4^ However, the selective cleavage of unstrained C–C(CO) moieties without an auxiliary directing group still remains an unmet challenge.^5^,^6^ Recently, transition metal-catalyzed C–C(CO) bond functionalization of 1,3-diones has been achieved by Jiao,6*a* Bolm,6*b* Peng,6*c* and Wu.6*d* These methods are understood to proceed through oxidative α-functionalization of 1,3-diones, followed by *retro*-Claisen condensation to provide α-substituted ketones (Scheme 1a). However, Lei's work demonstrated an alternative reaction pathway for the C–C(CO) bond cleavage of 1,3-diones, in which deacylative α-cupration could occur to form alkyl Cu(iii) complexes that subsequently underwent cross-coupling to give α-aryl ketones (Scheme 1b).^7^ Inspired by Lei's work, we questioned whether this open shell deacylative α-cupration mechanism might be translated to other transition metals, such as ruthenium, thereby allowing catalytic formation of alkyl Ru intermediates that can be captured by suitable coupling partners. In continuation of our research on C–C(CO) bond cleavage reactions,^8^ herein we disclose the first ruthenium-catalyzed deacylative annulation of 1,3-diones with sulfoxonium ylides (Scheme 1c). This method provides a practical and mild synthetic route to substituted furans,^9^ which are essential structural moieties in many biologically active compounds, natural products, and functional materials.^10^

On the other hand, sulfoxonium ylides are readily available and bench-stable carbene precursors, which have been extensively explored for the transition metal-catalyzed functionalization of C–H bonds.^11^ However, sulfoxonium ylide carbene-involved C–C bond functionalization has not yet been realized due to it being challenging to control the chemoselectivity from the same starting materials.^12^

## Results and discussion

We initiated our investigation on the model reaction of 1,3-diphenylpropane-1,3-dione (**1a**) with sulfoxonium ylide (**2a**) to optimize various reaction parameters. The results are summarized in Table 1. With [RuCl_2_(*p*-cymene)]_2_ as the catalyst, MesCO_2_H as the additive, and Na_3_PO_4_ as the base, the desired reaction occurred in HFIP to afford the desired furan product (**3aa**) in 23% yield (entry 1). However, the C–H carbene insertion product (**4a**)12a,b and the C–C carbene insertion product (**5a**)12b,c were also obtained as an inseparable mixture in 45% combined yield with 1 : 6 chemoselectivity. Investigations on various solvents indicated that toluene performed better than others, affording **3aa** in a yield of 35% with excellent chemoselectivity (Table 1, entries 1–6). The use of a proper base was crucial for this reaction, and the exploration of different bases revealed that ^*t*^BuOLi provided the best yield of 72% (entries 7–12). Only 10% yield of **3aa** was obtained in the absence of a base (entry 13). The MesCO_2_H additive was proved to be necessary to ensure the generation of **3aa**. In the absence of MesCO_2_H, trace **3aa** was observed (entry 14). A comparative yield was observed when MesCO_2_Li was used instead of ^*t*^BuOLi and MesCO_2_H (entry 15). The yield of **3aa** could be improved to 78% when the reaction was run at an elevated temperature (entry 16). However, a lower yield was obtained when the reaction was carried out at 130 °C (entry 17). Excitedly, when the solvent volume was increased to 2 mL, the desired product (**3aa**) was obtained in 85% yield (entry 18), but when the solvent volume was further increased to 3 mL, the yield of **3aa** was reduced to 66% (entry 19). Finally, in the absence of a catalyst, no desired product was observed (entry 20). Then, the optimized reaction conditions were identified as follows: **1a** (0.1 mmol), **2a** (2 equiv.), [RuCl_2_(*p*-cymene)]_2_ (5 mol%), MesCO_2_Li (1.5 equiv.) in toluene (2 mL) at 120 °C in air for 24 h (entry 18).

**Table 1:** Selected optimization studies^*a*^

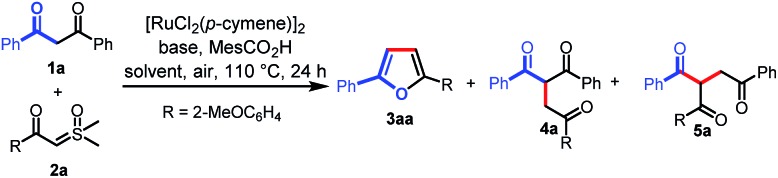				
Entry	Solvent	Base	Yield^*b*^ (%)	
			**3aa**	**4a** + **5a** (**4a** : **5a**)
1	HFIP	Na_3_PO_4_	23	45 (1 : 6)
2	^*i*^PrOH	Na_3_PO_4_	26	8 (1 : 3)
3	DMF	Na_3_PO_4_	30	20 (1 : 3)
4	CH_3_CN	Na_3_PO_4_	25	14 (1 : 2)
5	DCE	Na_3_PO_4_	15	24 (1 : 5)
6	Toluene	Na_3_PO_4_	35	Trace
7	Toluene	Na_2_CO_3_	30	Trace
8	Toluene	K_2_CO_3_	20	Trace
9	Toluene	Cs_2_CO_3_	25	Trace
10	Toluene	NaHCO_3_	24	0
11	Toluene	KH_2_PO_4_	52	0
12	Toluene	^*t*^BuOLi	72	Trace
13	Toluene	—	10	0
14^*c*^	Toluene	^*t*^BuOLi	Trace	0
15^*c*^	Toluene	MesCO_2_Li	71	Trace
16^*c*^ ^,^^*d*^	Toluene	MesCO_2_Li	78	Trace
17^*c*^ ^,^^*e*^	Toluene	MesCO_2_Li	40	Trace
18^*c*^ ^,^^*d*^ ^,^^*f*^	Toluene	MesCO_2_Li	85(82)^*g*^	Trace
19^*c*^ ^,^^*d*^ ^,^^*h*^	Toluene	MesCO_2_Li	66	Trace
20^*c*^ ^,^^*d*^ ^,^^*i*^	Toluene	MesCO_2_Li	0	0

^*a*^Reaction conditions: except where otherwise noted, all of the reactions were performed with **1a** (0.1 mmol), **2a** (0.2 mmol), base (0.15 mmol), MesCO_2_H (0.15 mmol), and [RuCl_2_(*p*-cymene)]_2_ (5 mol%) in a solvent (1 mL) at 110 °C in air for 24 h.

^*b*^The yields were determined by ^1^H NMR analysis of the crude product using CH_2_Br_2_ as the internal standard.

^*c*^Without MesCO_2_H.

^*d*^Reaction was carried out at 120 °C.

^*e*^Reaction was carried out at 130 °C.

^*f*^2 mL of toluene was used.

^*g*^Isolated yield.

^*h*^3 mL of toluene was used.

^*i*^Without [RuCl_2_(*p*-cymene)]_2_. HFIP = 1,1,1,3,3,3-hexafluoro-2-propanol. DMF = *N*,*N*-dimethylformamide. DCE = 1,2-dichloroethane. MesCO_2_H = 2,4,6-trimethylbenzoic acid.

**Scheme 2:**
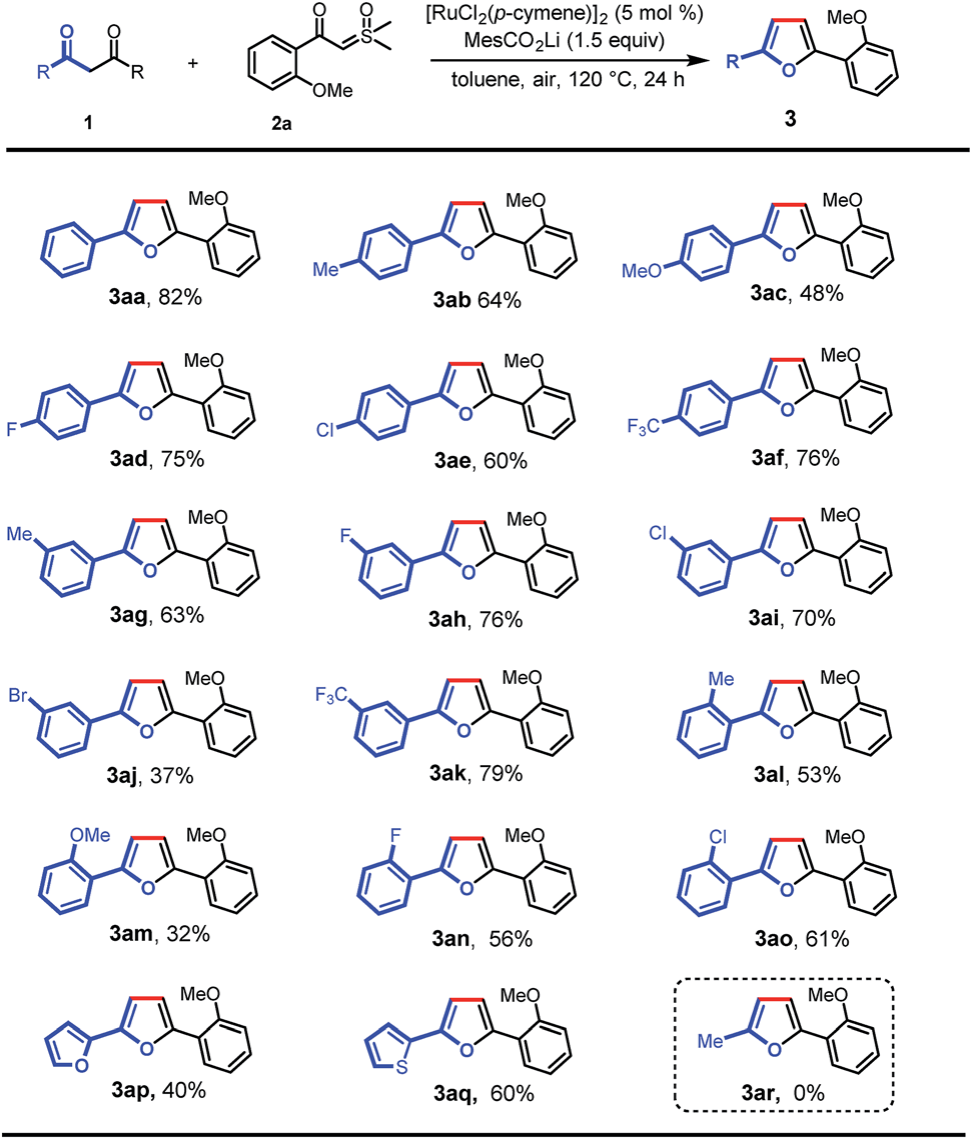
Scope of 1,3-diones. Reaction conditions: **1** (0.1 mmol), **2a** (0.2 mmol), MesCO_2_Li (0.15 mmol), and [RuCl_2_(*p*-cymene)]_2_ (5 mol%) in toluene (2 mL) at 120 °C in air for 24 h.

With the optimal conditions in hand, we turned our attention to the scope of 1,3-diones for this transformation (Scheme 2). It was found that the 1,3-diones bearing methyl, -methoxy, -halogen, and -CF_3_ groups could all be smoothly transformed to afford the substituted furan products in moderate to good yields (**3aa–ao**). The structure of **3ak** was unambiguously verified by single-crystal X-ray diffraction.^13^ The reactivity of this transformation was significantly influenced by the steric hindrance. 1,3-diones with *ortho*-substituted phenyl rings (**3al–ao**) generally gave lower yields of desired products than those with *meta*- and *para*-substituents (**3ab–af**). The electronic properties of the phenyl rings in 1,3-diones were observed to affect the reaction efficiency obviously. The substrates with electron-withdrawing groups (**3ad–af**, **3ah**, **3ai**, and **3ak**) gave higher yields than those with electron-donating groups (**3ab**, **3ac**, and **3ag**). However, a lower yield was observed when 1,3-dione with a bromo group was used (**3aj**). It is noteworthy that furan and thiophene rings were also tolerated, giving the desired products in moderate yields (**3ap** and **3aq**), which could be expected to find wide applications in organic electronics.10b,^14^ Finally, this reaction was not applicable to pentane-2,4-dione (**3ar**).

**Scheme 3:**
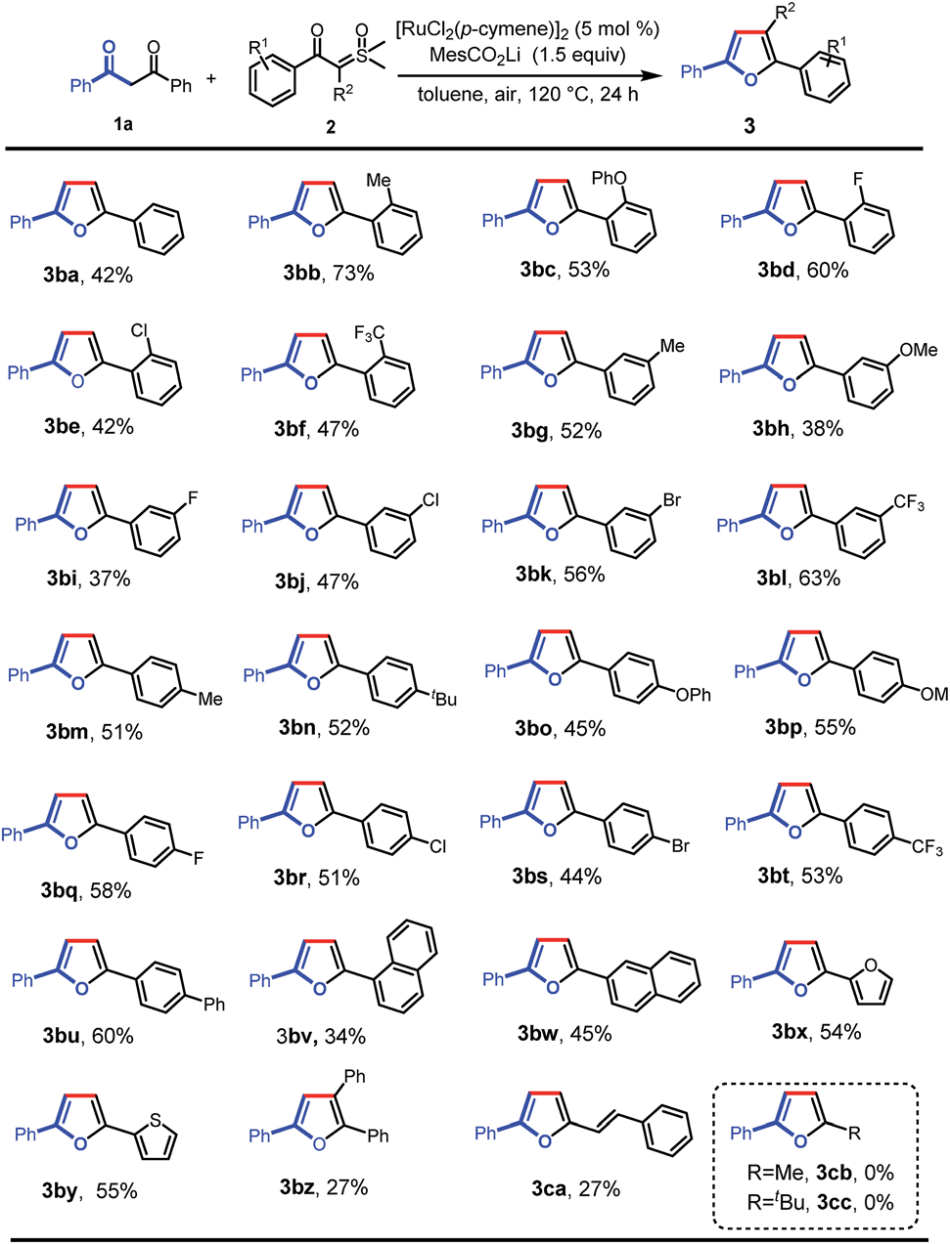
Scope of sulfoxonium ylides. Reaction conditions: **1a** (0.1 mmol), **2** (0.2 mmol), MesCO_2_Li (0.15 mmol), and [RuCl_2_(*p*-cymene)]_2_ (5 mol%) in toluene (2 mL) at 120 °C in air for 24 h.

Next, we further investigated the reaction of 1,3-diphenylpropane-1,3-dione with a variety of aroyl sulfoxonium ylides under the optimal reaction conditions (Scheme 3). Various valuable functional groups were tolerated, such as methyl, phenyl, phenoxyl, methoxyl, halogen, and trifluoromethyl. The reactivity was not sensitive to the steric hindrance and electronic properties of the phenyl rings on the sulfoxonium ylides. Substrates with *ortho*-substituted phenyl rings, having electron-donating moieties (**3ba–bc**) and electron-withdrawing groups (**3bd–bf**), gave the desired products in moderate to good yields. The substrates with *meta*- and *para*-substituted phenyl rings could be smoothly converted into the desired products in moderate yields (**3bg–bt**). Then, a phenyl group was introduced at the *para*-position which formed the tetra(aryl ring)-containing product **3bu** in 60% yield. In addition, 1-naphthalenyl (**3bv**), 2-naphthalenyl (**3bw**), 2-furyl (**3bx**), and 2-thienyl (**3by**) substrates were also tolerated, giving the corresponding products in the yields of 34%, 45%, 54%, and 55%, respectively. Importantly, triphenyl substituted furan (**3bz**) could also be obtained using α-phenyl sulfoxonium ylide as the substrate, albeit in low yield due to the increased bulkiness. Alkenoyl sulfoxonium ylide was also a capable substrate, giving 2-alkenyl substituted furan (**3ca**) in 27% yield. The aryl/alkenyl groups in conjugation with carbonyl are indispensable moieties for the successful formation of the corresponding furans, and the sulfoxonium ylides with alkanoyl groups failed to provide the desired products (**3cb** and **3cc**).

**Scheme 4:**
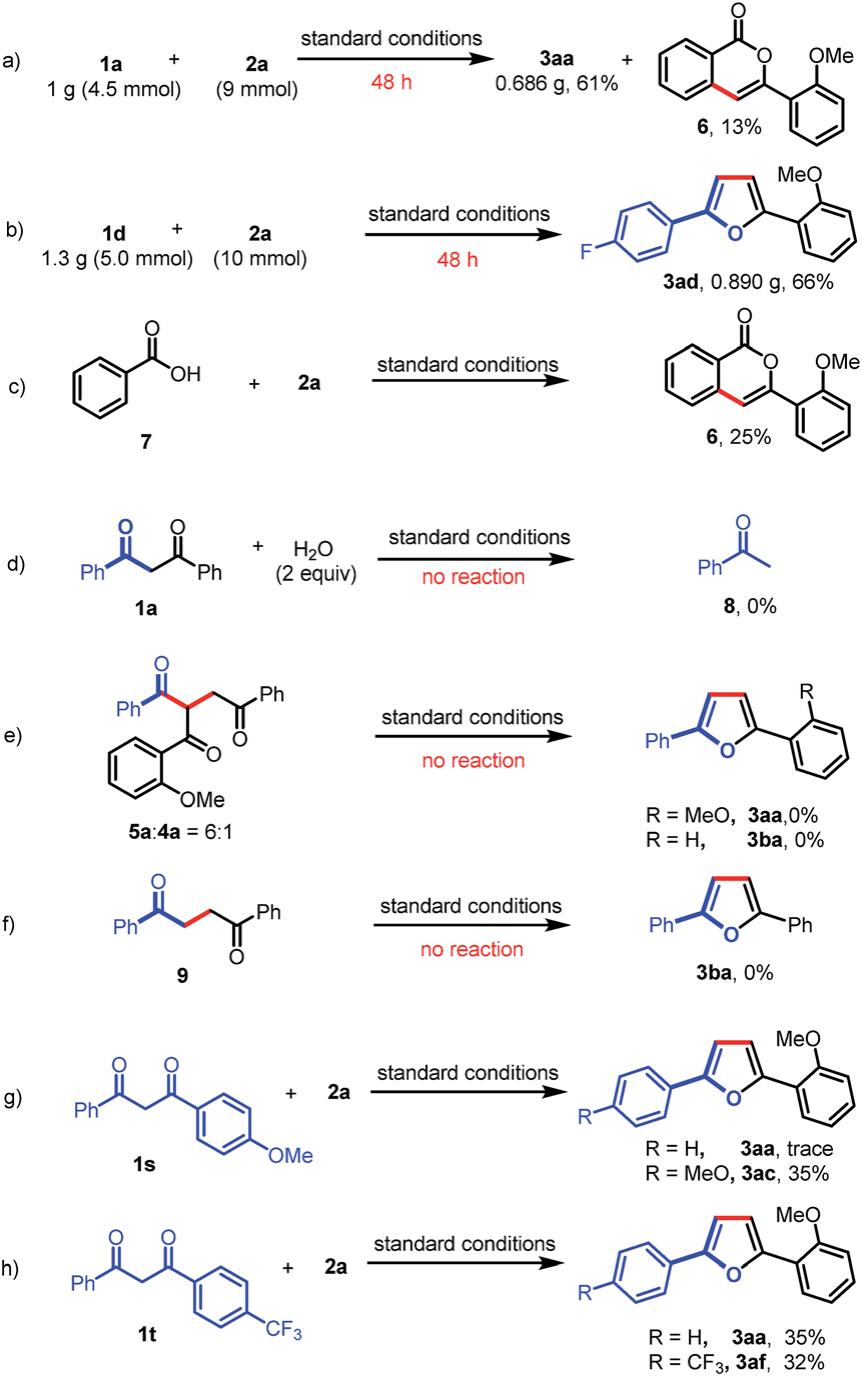
Gram-scale reactions and control experiments.

A gram-scale experiment of this deacylative annulation was demonstrated employing **1a** and **2a** as model substrates; the product **3aa** was obtained in 61% yield, along with an isocoumarin byproduct (**6**) (Scheme 4a). The 5 mmol scale reaction of **1d** and **2a** could give the product **3ad** in 66% yield (Scheme 4b). Ackermann recently reported Ru(ii)/Ag(i)-catalyzed C–H activation/annulation of benzoic acids with sulfoxonium ylides for the synthesis of isocoumarins.11*f* Indeed, we notice that isocoumarin (**6**) could also be generated under the silver-free conditions in 25% yield from benzoic acid (**7**) and **2a** (Scheme 4c). These results indicated that benzoic acid might be generated during the C–C bond cleavage process.

To further understand the reaction mechanism, the reaction of 1,3-dione (**1a**) and H_2_O was investigated under the standard reaction conditions. No reaction was observed, which indicated that the Ru(ii)-catalyzed C–C activation could not occur in the absence of a sulfoxonium ylide (Scheme 4d). Furthermore, a mixture of **5a** and **4a** (6 : 1) could not afford furan products (**3aa** or **3ba**) under the standard reaction conditions (Scheme 4e). To rule out the possibility that Paal–Knorr furan synthesis is involved in our transformation, we also prepared 1,4-diphenylbutane-1,4-dione (**9**); however, no desired product (**3ba**) was obtained when this 1,4-dione was subjected to the standard reaction conditions (Scheme 4f). Finally, the intramolecular competitive reactions of unsymmetrical 1,3-diones (**1s** or **1t**) with **2a** indicated that the chemoselectivity of this reaction was affected by the electron density of aryl-groups, and the C–C bond cleavage tended to occur at the less electron-rich moieties (Scheme 4g and h). The electron-deficient carbonyls are more likely to be attacked by a nucleophile, such as H_2_O, which may induce the subsequent C–C bond cleavage.

**Scheme 5:**
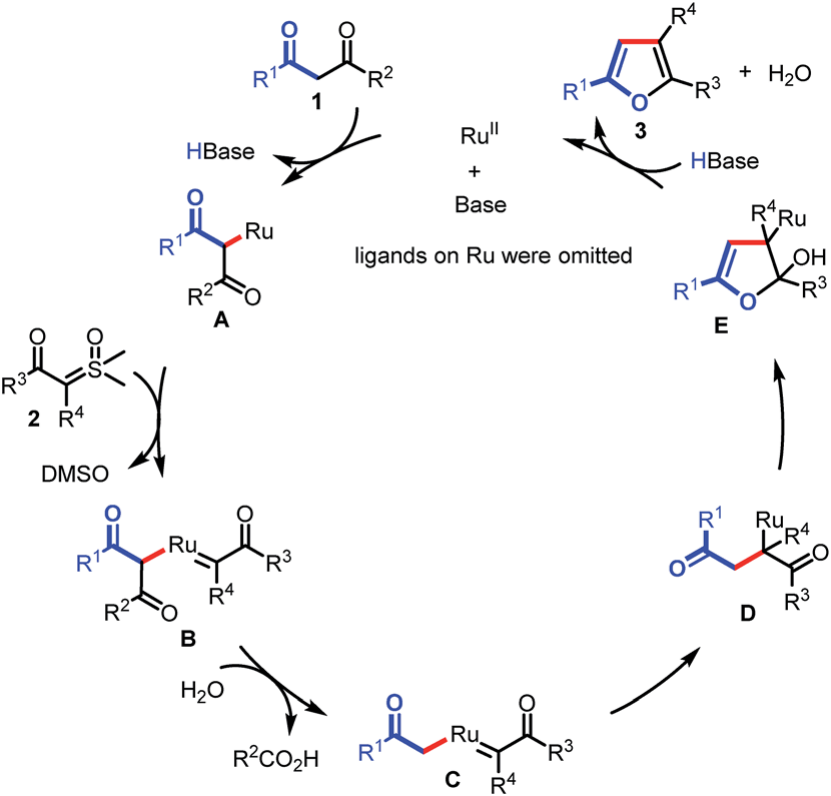
Plausible catalytic cycle.

On the basis of these results, we proposed that the reaction would proceed as shown in Scheme 5. The transformation begins with the generation of Ru complex **A** under basic conditions, which is subsequently captured by sulfoxonium ylide to form Ru carbene complex **B**. Then, C–C bond activation occurs in the presence of H_2_O, giving Ru complex **C**. Migratory insertion of **C** affords intermediate **D**. Finally, intramolecular annulation of **D** results in intermediate **E**, followed by β-O elimination to furnish the furan products (**3**) and regenerate the Ru(ii) catalyst.

## Conclusions

In conclusion, we have demonstrated the first example of Ru(ii)-catalyzed chemoselective deacylative annulation of 1,3-diones with sulfoxonium ylides. A series of substituted furans have been synthesized in reasonable yields by this novel method. This protocol which uses unstrained C–C(CO) bonds as nucleophiles in transition metal-catalyzed cross coupling should be expected to find wide applications. More work to better understand the mechanistic information on this strategy is currently underway.

## Conflicts of interest

There are no conflicts to declare.

## Supplementary Material

Supplementary information

Crystal structure data
