# Supplementary material for: Ruthenium(ii)-catalyzed chemoselective deacylative annulation of 1,3-diones with sulfoxonium ylides via C–C bond activation
Source: Chem Sci. 2019 Aug 12;10(39):9104–8. doi: 10.1039/c9sc03245b (PMC6889838; doi:10.1039/c9sc03245b)
Supplement: Supplementary file 1 [file SC-010-C9SC03245B-s001.pdf]

Electronic Supplementary Material (ESI) for Chemical Science.
This journal is © The Royal Society of Chemistry 2019

Ruthenium(II)-catalyzed chemoselective deacylative annulation of 1,3-diones with sulfoxonium ylides *via* C–C bond activation

Si Wen, Weiwei Lv, Dan Ba, Jing Liu, and Guolin Cheng*
College of Materials Science & Engineering, Huaqiao University, Xiamen 361021, China.
E-mail: glcheng@hqu.edu.cn

Table of Contents

1. General Information S2
2. Experimental Section S3
2.1 Optimization of the Reaction Conditions S3
2.2 Procedure for the Synthesis of 2 S6
2.3 Procedure for the Synthesis of 3 S7
3. Procedure for Gram-Scale Experiment of 3aa S21
4. Procedure for Gram-Scale Experiment of 3ad S22
5. X-Ray Crystallographic Data of 3ak S23
6. ^{1}H and ^{13}C NMR Spectra S25

1. General Information

All the solvents were used without further purification. the other commercial chemicals were used without further purification. All reactions were performed under an inert atmosphere of nitrogen in flame-dried glassware, unless otherwise stated. Analytical thin-layer chromatography was performed on 0.25 mm silica gel, 60-F254. Visualization was carried out with UV light and Vogel's permanganate. Preparative TLC was performed on 1.0 mm silica gel. ^{1}H NMR spectra were recorded on a Bruker DRX-500 instrument (500 MHz). ^{13}C NMR spectra were recorded on a Bruker DRX-500 instrument (126 MHz) and were fully decoupled by broad band proton decoupling. High-resolution mass spectra (HRMS) were recorded on an Agilent 1290 mass spectrometer using ESI-TOF (electrospray ionization time-of-flight). NMR spectra were recorded in $CDCl_3$. ^{1}H NMR spectra were referenced to residual $CHCl_3$ at 7.26 ppm, and ^{13}C NMR spectra were referenced to the central peak of $CDCl_3$ at 77.0 ppm. Chemical shifts (δ) are reported in ppm, and coupling constants (*J*) are reported in hertz (Hz). Multiplicities are reported using the following abbreviations: s = singlet, d = doublet, t = triplet, q = quartet, m = multiplet.

2. Experimental Section

2.1 Optimization of the Reaction Conditions

2.1.1 Table S1 Optimization of the Reaction Conditions[a]

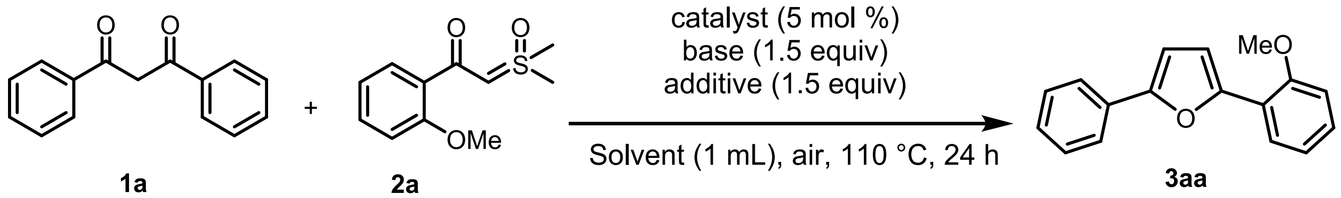

Entry	Catalyst	Solvent	Base	Additive	Yield (%)[b]
1	$[RuCl_2(p\text{-cymene})]_2$	HFIP	Na_3PO_4	$MesCO_2H$	23
2	$[RuCl_2(p\text{-cymene})]_2$	DCE	Na_3PO_4	$MesCO_2H$	15
3	$[RuCl_2(p\text{-cymene})]_2$	^{i}PrOH	Na_3PO_4	$MesCO_2H$	26
4	$[RuCl_2(p\text{-cymene})]_2$	DMF	Na_3PO_4	$MesCO_2H$	30
5	$[RuCl_2(p\text{-cymene})]_2$	CH_3CN	Na_3PO_4	$MesCO_2H$	25
6	$[RuCl_2(p\text{-cymene})]_2$	toluene	Na_3PO_4	$MesCO_2H$	35
7	$[RuCl_2(p\text{-cymene})]_2$	toluene	$NaHCO_3$	$MesCO_2H$	24
8	$[RuCl_2(p\text{-cymene})]_2$	toluene	K_2CO_3	$MesCO_2H$	20
9	$[RuCl_2(p\text{-cymene})]_2$	toluene	$KHCO_3$	$MesCO_2H$	22
10	$[RuCl_2(p\text{-cymene})]_2$	toluene	Cs_2CO_3	$MesCO_2H$	25
11	$[RuCl_2(p\text{-cymene})]_2$	toluene	NaOAc	$MesCO_2H$	30
12	$[RuCl_2(p\text{-cymene})]_2$	toluene	NaOH	$MesCO_2H$	26
13	$[RuCl_2(p\text{-cymene})]_2$	toluene	KOAc	$MesCO_2H$	15
14	$[RuCl_2(p\text{-cymene})]_2$	toluene	KOH	$MesCO_2H$	15
15	$[RuCl_2(p\text{-cymene})]_2$	toluene	K_2HPO_4	$MesCO_2H$	52
16	$[RuCl_2(p\text{-cymene})]_2$	toluene	K_3PO_4	$MesCO_2H$	15
17	$[RuCl_2(p\text{-cymene})]_2$	toluene	KH_2PO_4	$MesCO_2H$	40
18	$[RuCl_2(p\text{-cymene})]_2$	toluene	HCOONa	$MesCO_2H$	25
19	$[RuCl_2(p\text{-cymene})]_2$	toluene	CH_3ONa	$MesCO_2H$	25
20	$[RuCl_2(p\text{-cymene})]_2$	toluene	Na_2CO_3	$MesCO_2H$	30
21	$[RuCl_2(p\text{-cymene})]_2$	toluene	NaH_2PO_4	$MesCO_2H$	25
22	$[RuCl_2(p\text{-cymene})]_2$	toluene	^{t}BuOK	$MesCO_2H$	10
23	$[RuCl_2(p\text{-cymene})]_2$	toluene	^{t}BuONa	$MesCO_2H$	20

24	$[RuCl_2(p\text{-cymene})]_2$	toluene	LiOH	$MesCO_2H$	15
25	$[RuCl_2(p\text{-cymene})]_2$	toluene	CH_3OK	$MesCO_2H$	10
26	$[RuCl_2(p\text{-cymene})]_2$	toluene	Li_2CO_3	$MesCO_2H$	10
27	$[RuCl_2(p\text{-cymene})]_2$	toluene	CsOAc	$MesCO_2H$	12
28	$[RuCl_2(p\text{-cymene})]_2$	toluene	^{t}BuOLi	$MesCO_2H$	72
29	$[RuCl_2(p\text{-cymene})]_2$	toluene	-	$MesCO_2H$	10
30	$[RuCl_2(p\text{-cymene})]_2$	toluene	^{t}BuOLi	-	trace
31	$[RuCl_2(p\text{-cymene})]_2$	toluene	^{t}BuOLi	AcOH	20
32	$[RuCl_2(p\text{-cymene})]_2$	toluene	^{t}BuOLi	Ac-Gly-OH	10
33	$[RuCl_2(p\text{-cymene})]_2$	toluene	^{t}BuOLi	Val-Boc-OH	10
34	$[RuCl_2(p\text{-cymene})]_2$	toluene	$MesCO_2Li$	-	71
35	$[RuCl_2(p\text{-cymene})]_2$	DCE	$MesCO_2Li$	-	20
36	$[RuCl_2(p\text{-cymene})]_2$	NMP	$MesCO_2Li$	-	trace
37	$[RuCl_2(p\text{-cymene})]_2$	1,4-dioxane	$MesCO_2Li$	-	20
38	$[RuCl_2(p\text{-cymene})]_2$	THF	$MesCO_2Li$	-	58
39	$[RuCl_2(p\text{-cymene})]_2$	HFIP	$MesCO_2Li$	-	10
40	$[RuCl_2(p\text{-cymene})]_2$	^{t}AmOH	$MesCO_2Li$	-	15
41	$[RuCl_2(p\text{-cymene})]_2$	DME	$MesCO_2Li$	-	62
42	$[RuCl_2(p\text{-cymene})]_2$	DMSO	$MesCO_2Li$	-	N.R.
43	$[RuCl_2(p\text{-cymene})]_2$	DMF	$MesCO_2Li$	-	10
44	$[RuCl_2(p\text{-cymene})]_2$	^{i}PrOH	$MesCO_2Li$	-	15
45	$[RuCl_2(p\text{-cymene})]_2$	TMBE	$MesCO_2Li$	-	60
46	$[RuCl_2(p\text{-cymene})]_2$	CH_3CN	$MesCO_2Li$	-	10
47[c]	$[RuCl_2(p\text{-cymene})]_2$	toluene	$MesCO_2Li$	-	12

48[d]	$[RuCl_2(p\text{-cymene})]_2$	toluene	$MesCO_2Li$	-	66
49[e]	$[RuCl_2(p\text{-cymene})]_2$	toluene	$MesCO_2Li$	-	78
50[f]	$[RuCl_2(p\text{-cymene})]_2$	toluene	$MesCO_2Li$	-	40
51[g]	$[RuCl_2(p\text{-cymene})]_2$	toluene	$MesCO_2Li$	-	30
52	$Pd(OAc)_2$	toluene	$MesCO_2Li$	-	0
53	$[Cp^*RhCl_2]_2$	toluene	$MesCO_2Li$	-	0
54	$Cu(OAc)_2$	toluene	$MesCO_2Li$	-	0
55	AgOAc	toluene	$MesCO_2Li$	-	0

[a] **1a** (0.1 mmol), **2a** (0.2 mmol), solvent (1 mL), base (1.5 equiv), catalyst (5 mol%), additive (1.5 equiv) at 110 °C under air for 24 h. [b] The yields were determined by ^1H NMR analysis of the crude product using CH_2Br_2 as the internal standard. [c] At 80°C. [d] At 100°C. [e] At 120°C. [f] At 130°C. [g] At 140°C.

2.1.2 Table S2 Optimization of the Reaction Conditions[a]

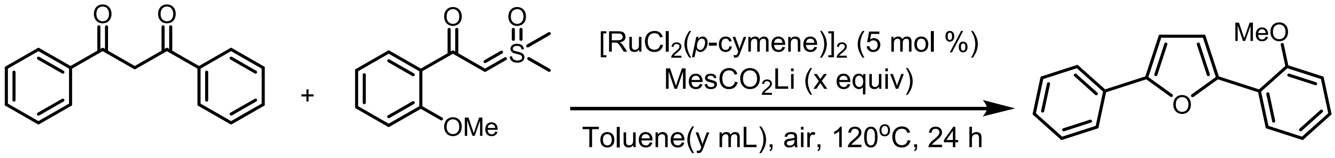

1a **2a** **3aa**

Entry	Solvent	Base	Yield (%)[b]
1	toluene	$MesCO_2Li$ (1.0 equiv)	66
2	toluene	$MesCO_2Li$ (2.0 equiv)	72
3	toluene (0.5 mL)	$MesCO_2Li$	72
4	toluene (2 mL)	$MesCO_2Li$	85
5	toluene (3 mL)	$MesCO_2Li$	66

[a] **1a** (0.1 mmol), **2a** (0.2 mmol), $MesCO_2Li$, $[RuCl_2(p\text{-cymene})]_2$ (5 mol%), in toluene at 120 °C for 24 h. [b] The yields were determined by ^1H NMR analysis of the crude product using CH_2Br_2 as the internal standard.

2.2 Procedure for the Synthesis of 2

Method 1: Under N_2, trimethylsulfoxonium iodide (3.3 g, 15 mmol, 3.0 equiv) was suspended in THF (50 mL) in a flame-dried 100 mL round bottom flask, ^{t}BuOK (1.7 g, 15 mmol, 3.0 equiv) was added and the mixture was stirred at room temperature for 2 hours. After, benzoyl chloride (5 mmol, 1.0 equiv) was added. The mixture was stirred at room temperature for 3 hours and then filtered through a plug of celite beofre all volatiles were removed under vacuum. Purification by flash chromatography (DCM/MeOH = 50 :1) afforded products[1].

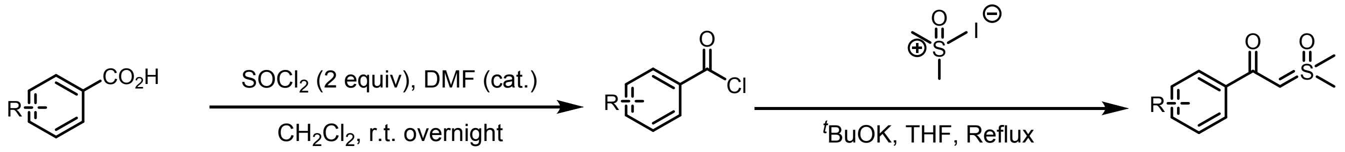

Method 2: Sulfurous dichloride (10 mmol, 2.0 equiv) was added to a mixture of the carboxylic acid (5 mmol, 1.0 equiv), *N,N*-dimethylformamide 3 drops in CH_2Cl_2 (30 mL) under N_2 atmosphere at 0 °C. The mixture was stirred at at room temperature overnight. Then, the excess of sulfurous dichloride and CH_2Cl_2 were removed in vacuo, obtain crude benzoyl chloride[2].

Under N_2, trimethylsulfoxonium iodide (3.3 g, 15 mmol, 3.0 equiv) was suspended in THF (50 mL) in a flame-dried 100 mL round bottom flask, ^{t}BuOK (1.7 g, 15 mmol, 3.0 equiv) was added and the mixture was stirred at room temperature for 2 hours. After, crude benzoyl chloride (5 mmol, 1.0 equiv) was added. The mixture was stirred at room temperature for 3 hours and then filtered through a plug of celite beofre all volatiles were removed under vacuum. Purification by flash chromatography (DCM/MeOH = 50 :1) afforded products [1].

Method 3: **2az** was synthesized following a literature procedure[3]

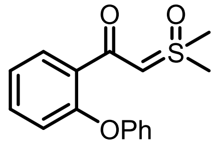

2ac

2-(dimethyl(oxo)-λ^6-sulfanylidene)-1-(2-phenoxyphenyl)ethan-1-one (**2ac**) as cream solid. ^{1}H NMR (500 MHz, Chloroform-d) δ 7.88 (d, J = 7.7 Hz, 1H), 7.32 (t, J = 7.7 Hz, 1H), 7.27 (t, J = 7.8 Hz, 2H), 7.18 (t, J = 7.5 Hz, 1H), 7.01 (t, J = 7.4 Hz, 1H), 6.91 (t, J = 8.5 Hz, 3H), 5.20 (s, 1H), 3.25 (s, 6H); ^{13}C NMR (126 MHz, Chloroform-d) δ 179.3, 157.3, 153.3, 131.7, 131.0, 129.7, 129.3, 123.7, 122.2, 120.15, 117.2, 73.9, 41.1; HRMS (ESI-TOF) m/z: calcd for $C_{16}H_{17}O_3S^+$: 289.0893 $(M + H)^+$, found: 289.0891.

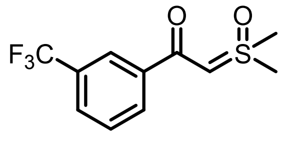

2al

2-(dimethyl(oxo)-λ⁶-sulfanylidene)-1-(3-(trifluoromethyl)phenyl)ethan-1-one (**2al**) as beige solid. ^{1}H NMR (500 MHz, Chloroform-d) δ 8.06 (s, 1H), 7.98 – 7.92 (m, 1H), 7.66 (t, J = 6.2 Hz, 1H), 7.48 (q, J = 7.3 Hz, 1H), 5.19 (d, J = 2.7 Hz, 1H), 3.54 (d, J = 3.6 Hz, 6H); ^{13}C NMR (126 MHz, Chloroform-d) δ 180.2, 139.4, 130.1 (q, J = 32.3 Hz), 129.6, 128.5, 126.9 (q, J = 3.7 Hz), 123.8 (q, J = 272.4 Hz), 123.2 (q, J = 3.9 Hz), 70.2, 41.6; HRMS (ESI-TOF) m/z: calcd for $C_{11}H_{12}F_3O_2S^+$: 265.0505 $(M + H)^+$, found: 265.0507.

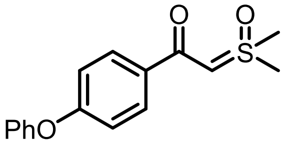

2ao

2-(dimethyl(oxo)-λ⁶-sulfanylidene)-1-(4-phenoxyphenyl)ethan-1-one (**2ao**) as white solid. ^{1}H NMR (500 MHz, Chloroform-d) δ 7.77 (d, J = 8.8 Hz, 2H), 7.35 (t, J = 8.0 Hz, 2H), 7.13 (t, J = 7.5 Hz, 1H), 7.05 – 7.01 (m, 2H), 6.99 – 6.94 (m, 2H), 5.00 (s, 1H), 3.49 (s, 6H); ^{13}C NMR (126 MHz, Chloroform-d) δ 181.3, 159.5, 156.1, 133.5, 129.7, 128.3, 123.7, 119.4, 117.5, 68.2, 42.1; HRMS (ESI-TOF) m/z: calcd for $C_{16}H_{17}O_3S^+$: 289.0893 $(M + H)^+$, found: 289.0895.

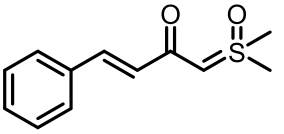

2ba

(***E***)**-1-(dimethyl(oxo)-λ⁶-sulfanylidene)-4-phenylbut-3-en-2-one** (**2ba**) as white solid. ^{1}H NMR (500 MHz, Chloroform-d) δ 7.54 – 7.47 (m, 2H), 7.46 – 7.41 (m, 1H), 7.39 – 7.27 (m, 3H), 6.63 – 6.55 (m, 1H), 4.59 (d, J = 1.7 Hz, 1H), 3.51 – 3.46 (m, 6H); ^{13}C NMR (126 MHz, Chloroform-d) δ 179.9, 136.4, 135.5, 129.0, 128.6, 127.6, 126.8 (d, J = 1.7 Hz), 71.8, 71.7, 42.3 (d, J = 4.5 Hz); HRMS (ESI-TOF) m/z: calcd for $C_{12}H_{15}O_2S^+$: 223.0787 $(M + H)^+$, found: 223.0789.

2.3 Procedure for the Synthesis of 3

1 was synthesized following a literature procedure[4].

2.3.1 Procedure for Synthesis of 3aa-3aq

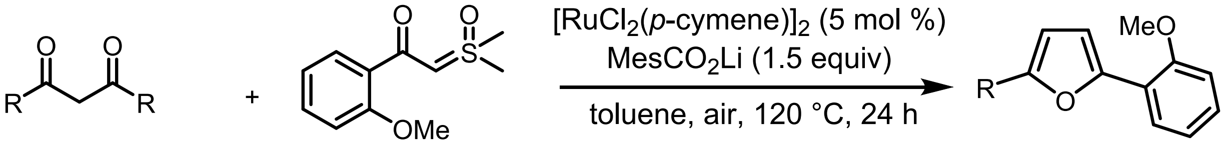

A dried 10 mL Schlenk tube was charged with **1,3-diphenylpropane-1,3-dione 1** (0.1 mmol, 1 equiv), **2-(dimethyl(oxo)-λ⁶-sulfanylidene)-1-(2-methoxyphenyl)ethan-1-one 2a** (45.3 mg, 0.2 mmol, 2 equiv), $[RuCl_2(p\text{-cymene})]_2$ (3.1 mg, 0.005 mmol, 5 mol %), $MesCO_2Li$ (25.5 mg, 0.15 mmol, 1.5 equiv), and toluene (2 mL). The reaction mixture was heated to 120 °C for 24 hours under vigorous

stirring. Upon completion, the reaction mixture was cooled to room temperature, diluted with ethyl acetate, and filtered through a pad of celite. The filtrate was concentrated under vacuum, and the resulting residue was purified by preparative thin layer chromatography (PTLC) with hexane to give the corresponding products.

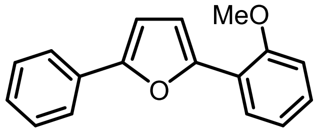

3aa

2-(2-methoxyphenyl)-5-phenylfuran （**3aa**） (20.4 mg, 82%) as a white solid. ^{1}H NMR (500 MHz, Chloroform-d) δ8.00 – 7.97 (m, 1H), 7.76 (d, J = 7.5 Hz, 2H), 7.40 (t, J = 7.8 Hz, 2H), 7.29 – 7.22 (m, 2H), 7.06 (d, J = 7.7 Hz, 1H), 7.03 (d, J = 3.4 Hz, 1H), 6.97 (d, J = 8.2 Hz, 1H), 6.76 (d, J = 3.4 Hz, 1H), 3.95 (s, 3H); ^{13}C NMR (126 MHz, Chloroform-d) δ 155.4, 152.2, 149.7, 130.9, 128.6, 128.0, 127.1, 125.8, 123.7, 120.7, 119.8, 112.2, 110.9, 107.4, 55.4; HRMS (ESI-TOF) m/z: calcd for $C_{17}H_{15}O_2^+$: 251.1067 $(M + H)^+$, found: 251.1068.

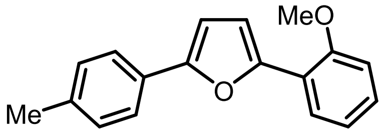

3ab

2-(2-methoxyphenyl)-5-(*p*-tolyl)furan (**3ab**) (16.9 mg, 64%) was prepared from typical procedure (hexane) as yellowish solid. ^{1}H NMR (500 MHz, Chloroform-d) δ 8.00 – 7.96 (m, 1H), 7.65 (d, J = 8.1 Hz, 2H), 7.26 – 7.22 (m, 1H), 7.20 (d, J = 7.9 Hz, 2H), 7.05 (t, J = 7.6 Hz, 1H), 7.02 (d, J = 3.4 Hz, 1H), 6.96 (d, J = 8.2 Hz, 1H), 6.70 (d, J = 3.4 Hz, 1H), 3.95 (s, 3H), 2.37 (s, 3H); ^{13}C NMR (126 MHz, Chloroform-d) δ 155.4, 152.5, 149.3, 137.0, 129.3, 128.2, 127.8, 125.7, 123.7, 120.7, 119.9, 112.2, 110.9, 106.6, 55.4, 21.3; HRMS (ESI-TOF) m/z: calcd for $C_{18}H_{17}O_2^+$: 265.1223 $(M + H)^+$, found: 265.1227.

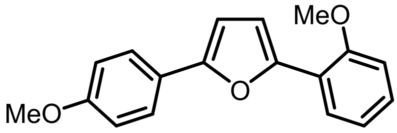

3ac

2-(2-methoxyphenyl)-5-(4-methoxyphenyl)furan (**3ac**) (13.5 mg, 48%) was prepared from typical procedure (hexane) as white solid. ^{1}H NMR (500 MHz, Chloroform-d) δ7.97 – 7.95 (m, 1H), 7.72 – 7.65 (m, 2H), 7.25 – 7.21 (m, 1H), 7.06 – 7.02 (m, 1H), 7.01 (d, J = 3.4 Hz, 1H), 6.97 – 6.92 (m, 3H), 6.62 (d, J = 3.4 Hz, 1H), 3.94 (s, 3H), 3.83 (s, 3H); ^{13}C NMR (126 MHz, Chloroform-d) δ 158.9, 155.3, 152.3, 149.1, 127.7, 125.6, 125.2, 124.0, 120.7, 119.9, 114.1, 112.2, 110.9, 105.8, 55.3 (d, J = 3.4 Hz); HRMS (ESI-TOF) m/z: calcd for $C_{18}H_{17}O_3^+$: 281.1172 $(M + H)^+$, found: 281.1177.

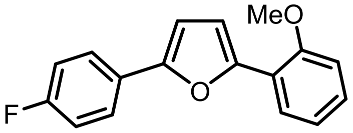

3ad

2-(4-fluorophenyl)-5-(2-methoxyphenyl)furan (**3ad**) (20.1 mg, 75%) was prepared from typical procedure (hexane) as yellowish solid. ^1H NMR (500 MHz, Chloroform-d) δ 7.97 – 7.93 (m, 1H), 7.74 – 7.67 (m, 2H), 7.27 – 7.22 (m, 1H), 7.11 – 7.07 (m, 2H), 7.07 – 7.03 (m, 1H), 7.01 (d, J = 3.4 Hz, 1H), 6.96 (d, J = 8.3 Hz, 1H), 6.68 (d, J = 3.4 Hz, 1H), 3.95 (s, 3H); ^{13}C NMR (126 MHz, Chloroform-d) δ162.0 (d, *J* = 246.9 Hz), 155.4, 151.4, 149.8, 128.0, 127.3 (d, *J* = 3.3 Hz), 125.7, 125.4 (d, *J* = 7.9 Hz), 120.7, 119.7, 115. 7 (d, *J* = 21.9 Hz), 112.2, 111.0, 107.0 (d, *J* = 1.7 Hz), 55.4; HRMS (ESI-TOF) m/z: calcd for $C_{17}H_{14}FO_2^+$: 269.0972 $(M + H)^+$, found: 269.0977.

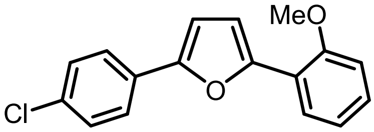

3ae

2-(4-chlorophenyl)-5-(2-methoxyphenyl)furan (**3ae**) (17 mg, 60%) was prepared from typical procedure (hexane) as colorless solid. ^1H NMR (500 MHz, Chloroform-d) δ 7.98 – 7.92 (m, 1H), 7.69 – 7.64 (m, 2H), 7.37 – 7.33 (m, 2H), 7.27 – 7.23 (m, 1H), 7.07 – 7.03 (m, 1H), 7.02 (d, J = 3.4 Hz, 1H), 6.97 (d, J = 8.2 Hz, 1H), 6.74 (d, J = 3.5 Hz, 1H), 3.95 (s, 3H); ^{13}C NMR (126 MHz, Chloroform-d) δ 155.5, 151.1, 150.1, 132.7, 129.4, 128.8, 128.2, 125.8, 124.9, 120.7, 119.5, 112.3, 111.0, 107.8, 55.4; HRMS (ESI-TOF) m/z: calcd for $C_{17}H_{14}ClO_2^+$: 285.0677 $(M + H)^+$, found: 285.0679.

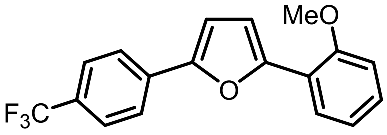

3af

2-(2-methoxyphenyl)-5-(4-(trifluoromethyl)phenyl)furan (**3af**) (24.2 mg, 76%) was prepared from typical procedure (hexane) as white solid. ^1H NMR (500 MHz, Chloroform-d) δ 8.00 – 7.94 (m, 1H), 7.85 – 7.79 (m, 2H), 7.63 (d, J = 8.2 Hz, 2H), 7.31 – 7.26 (m, 1H), 7.09 – 7.06 (m, 1H), 7.05 (d, J = 3.4 Hz, 1H), 6.98 (d, J = 8.2 Hz, 1H), 6.86 (d, J = 3.5 Hz, 1H), 3.95 (s, 3H); ^{13}C NMR (126 MHz, Chloroform-d) δ 155.7, 150.9, 150.7, 133.9, 128.6 (q, J = 32.3 Hz), 128.5, 125.9, 125.7 (q, J = 3.9 Hz), 124.2 (q, J = 271.7 Hz), 123.6, 123.1, 120.8, 119.3, 112.4, 111.0, 109.4, 55.4; HRMS (ESI-TOF) m/z: calcd for $C_{18}H_{14}F_3O_2^+$: 319.0940 $(M + H)^+$, found: 319.0944.

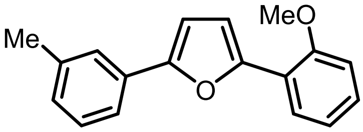

3ag

2-(2-methoxyphenyl)-5-(*m*-tolyl)furan (**3ag**) (16.7 mg, 63%) was prepared from typical procedure (hexane) as yellow solid. ^{1}H NMR (500 MHz, Chloroform-d) δ 8.02 – 7.97 (m, 1H), 7.56 (d, J = 11.4 Hz, 2H), 7.28 (t, J = 7.6 Hz, 1H), 7.26 – 7.21 (m, 1H), 7.09 – 7.05 (m, 2H), 7.02 (d, J = 3.5 Hz, 1H), 6.96 (d, J = 8.3 Hz, 1H), 6.75 – 6.73 (m, 1H), 3.94 (s, 3H), 2.40 (s, 3H). ^{13}C NMR (126 MHz, Chloroform-d) δ 155.4, 152.4, 149.6, 138.2, 130.8, 128.6, 128.0, 127.9, 125.8, 124.3, 121.0, 120.7, 119.8, 112.2, 111.0, 107.3, 55.3, 21.5. HRMS (ESI-TOF) m/z: calcd for $C_{18}H_{17}O_2^+$: 265.1223 (M + H)$^+$, found: 265.1227.

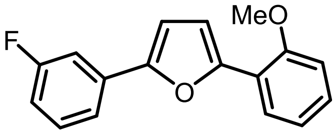

3ah

2-(3-fluorophenyl)-5-(2-methoxyphenyl)furan (**3ah**) (20.3 mg, 76%) was prepared from typical procedure (hexane) as colorless solid. ^{1}H NMR (500 MHz, Chloroform-d) δ 8.00 – 7.93 (m, 1H), 7.53 – 7.49 (m, 1H), 7.47 – 7.40 (m, 1H), 7.37 – 7.31 (m, 1H), 7.30 – 7.22 (m, 1H), 7.09 – 7.04 (m, 1H), 7.03 (d, J = 3.5 Hz, 1H), 6.96 (d, J = 8.5 Hz, 1H), 6.97 – 6.90 (m, 1H), 6.77 (d, J = 3.5 Hz, 1H), 3.95 (s, 3H); ^{13}C NMR (126 MHz, Chloroform-d) δ 163.2 (d, J = 244.9 Hz), 155.6, 151.0 (d, J = 3.1 Hz), 150.3, 133.0 (d, *J* = 8.5 Hz), 130.2 (d, *J* = 8.6 Hz), 128.3, 125.9, 120.8, 119.5, 119.3 (d, J = 2.7 Hz), 113.8 (d, *J* = 21.5 Hz), 112.2, 111.0, 110.5 (d, *J* = 23.5 Hz), 108.4, 55.4; HRMS (ESI-TOF) m/z: calcd for $C_{17}H_{14}FO_2^+$: 269.0972 (M + H)$^+$, found: 269.0975.

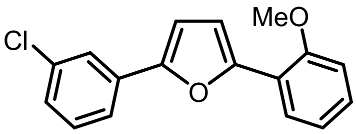

3ai

2-(3-chlorophenyl)-5-(2-methoxyphenyl)furan (**3ai**) (20 mg, 70%) was prepared from typical procedure (hexane) as cream solid. ^{1}H NMR (500 MHz, Chloroform-d) δ 7.99 – 7.95 (m, 1H), 7.72 (t, J = 1.8 Hz, 1H), 7.60 (d, J = 7.8 Hz, 1H), 7.31 (t, J = 7.9 Hz, 1H), 7.28 – 7.23 (m, 1H), 7.23 – 7.19 (m, 1H), 7.06 (t, J = 7.5 Hz, 1H), 7.03 (d, J = 3.5 Hz, 1H), 6.97 (d, J = 8.0 Hz, 1H), 6.77 (d, J = 3.4 Hz, 1H), 3.95 (s, 3H); ^{13}C NMR (126 MHz, Chloroform-d) δ 155.6, 150.7, 150.4, 134.7, 132.5, 129.9, 128.3, 127.0, 125.9, 123.6, 121.7, 120.8, 119.4, 112.3, 111.0, 108.5, 55.4; HRMS (ESI-TOF) m/z: calcd for $C_{17}H_{14}ClO_2^+$: 285.0677 (M + H)$^+$, found: 285.0678.

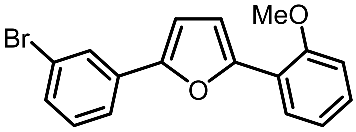

3aj

2-(3-bromophenyl)-5-(2-methoxyphenyl)furan (**3aj**) (12.1 mg, 37%) was prepared from typical procedure (hexane) as colorless solid. ^{1}H NMR (500 MHz, Chloroform-d) δ 7.99 – 7.95 (m, 1H), 7.88 (t, J = 1.8 Hz, 1H), 7.68 – 7.63 (m, 1H), 7.38 – 7.35 (m, 1H), 7.26 (q, J = 7.9, 7.3 Hz, 2H), 7.09 – 7.05 (m,

1H), 7.03 (d, J = 3.5 Hz, 1H), 6.97 (d, J = 8.3 Hz, 1H), 6.77 (d, *J* = 3.5 Hz, 1H), 3.96 (s, 3H); ^{13}C NMR (126 MHz, Chloroform-d) δ 155.6, 150.5, 150.4, 132.8, 130.2, 129.9, 128.3, 126.5, 125.9, 122.9, 122.2, 120.8, 119.4, 112.3, 111.0, 108.5, 55.4; HRMS (ESI-TOF) m/z: calcd for $C_{17}H_{14}BrO_2^+$: 329.0172 (M + H)$^+$, found: 329.0175.

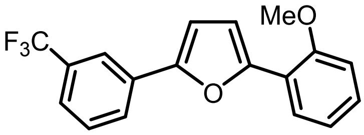

3ak

2-(2-methoxyphenyl)-5-(3-(trifluoromethyl)phenyl)furan (**3ak**) (25 mg, 79%) was prepared from typical procedure (hexane) as colorless solid. ^{1}H NMR (500 MHz, Chloroform-d) δ 8.01 – 7.94 (m, 2H), 7.92 – 7.86 (m, 1H), 7.52 – 7.47 (m, 2H), 7.29 – 7.25 (m, 1H), 7.09 – 7.06 (m, 1H), 7.05 (d, J = 3.5 Hz, 1H), 6.97 (d, J = 8.2 Hz, 1H), 6.83 (d, J = 3.5 Hz, 1H), 3.95 (s, 3H); ^{13}C NMR (126 MHz, Chloroform-d) δ 155.6, 150.64, 150.61, 131.5, 131.2 (q, J = 32.3 Hz), 129.1, 128.4, 126.6, 125.9, 124.0 (q, J = 272.4 Hz), 123.5 (q, J = 3.9 Hz), 120.8, 120.3 (q, J = 4.0 Hz), 119.4, 112.3, 111.0, 108.7, 55.4; HRMS (ESI-TOF) m/z: calcd for $C_{18}H_{14}F_3O_2^+$: 319.0940 (M + H)$^+$, found: 319.0943.

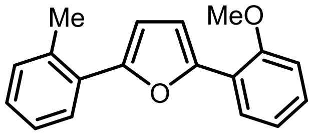

3al

2-(2-methoxyphenyl)-5-(*o*-tolyl)furan (**3al**) (13.9 mg, 53%) was prepared from typical procedure (hexane) as white solid. ^{1}H NMR (500 MHz, Chloroform-d) δ 7.97 – 7.94 (m, 1H), 7.83 – 7.77 (m, 1H), 7.29 – 7.24 (m, 3H), 7.24 – 7.18 (m, 1H), 7.06 – 7.03 (m, 2H), 6.98 (d, J = 8.2 Hz, 1H), 6.66 (d, J = 3.5 Hz, 1H), 3.96 (s, 3H), 2.58 (s, 3H); ^{13}C NMR (126 MHz, Chloroform-d) δ 155.5, 152.0, 149.4, 134.4, 131.2, 130.2, 128.0, 127.3, 126.9, 126.0, 125.8, 120.8, 119.8, 111.9, 110.9, 110.8, 55.4, 22.1; HRMS (ESI-TOF) m/z: calcd for $C_{18}H_{17}O_2^+$: 265.1223 (M + H)$^+$, found: 265.1227.

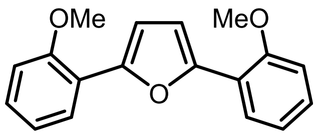

3am

2,5-bis(2-methoxyphenyl)furan (**3am**) (8.9 mg, 32%) was prepared from typical procedure (hexane) as white solid. ^{1}H NMR (500 MHz, Chloroform-d) δ 8.02 – 7.98 (m, 2H), 7.27 – 7.21 (m, 2H), 7.09 – 7.02 (m, 4H), 6.97 (d, J = 8.2 Hz, 2H), 3.96 (s, 6H); ^{13}C NMR (126 MHz, Chloroform-d) δ 155.5, 148.7, 127.8, 125.9, 120.7, 119.9, 112.4, 111.0, 55.4; HRMS (ESI-TOF) m/z: calcd for $C_{18}H_{17}O_3^+$: 281.1172 (M + H)$^+$, found: 281.1176.

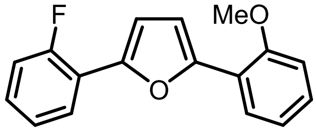

3an

2-(2-fluorophenyl)-5-(2-methoxyphenyl)furan (**3an**) (15 mg, 56%) was prepared from typical procedure (hexane) as cream solid. ^{1}H NMR (500 MHz, Chloroform-d) δ 7.99 – 7.97 (m, 1H), 7.97 – 7.92 (m, 1H), 7.30 – 7.24 (m, 1H), 7.24 – 7.18 (m, 2H), 7.14 – 7.09 (m, 1H), 7.09 – 7.02 (m, 2H), 6.97 (d, J = 8.3 Hz, 1H), 6.95 (t, J = 3.7 Hz, 1H), 3.96 (s, 3H); ^{13}C NMR (126 MHz, Chloroform-d) δ 158.7 (d, J = 250.7 Hz), 155.6, 149.6, 146.4 (d, J = 3.0 Hz), 128.2, 128.0 (d, J = 8.2 Hz), 125.92, 125.91 (d, J = 4.2 Hz), 124.3 (d, J = 3.5 Hz), 120.7, 119.6, 119.2 (d, J = 12.1 Hz), 125.9 (d, J = 21.6 Hz), 112.5 (d, J = 11.8 Hz),, 112.4 (d, J = 1.7 Hz), 111.0, 55.4; HRMS (ESI-TOF) m/z: calcd for $C_{17}H_{14}FO_2^+$: 269.0972 $(M + H)^+$, found: 269.0974.

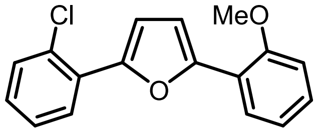

3ao

2-(2-chlorophenyl)-5-(2-methoxyphenyl)furan (**3ao**) (17.4 mg, 61%) was prepared from typical procedure (hexane) as orange solid. ^{1}H NMR (500 MHz, Chloroform-d) δ 8.02 – 7.94 (m, 2H), 7.48 – 7.41 (m, 1H), 7.38 – 7.30 (m, 1H), 7.29 – 7.26 (m, 1H), 7.26 – 7.23 (m, 1H), 7.21 – 7.17 (m, 1H), 7.10 – 7.02 (m, 2H), 6.98 (d, J = 8.2 Hz, 1H), 3.97 (s, 3H); ^{13}C NMR (126 MHz, Chloroform-d) δ 155.7, 149.8, 148.5, 130.8, 130.0, 129.2, 128.3, 127.8, 127.7, 126.8, 126.0, 120.7, 119.5, 113.3, 112.2, 111.0, 55.4. HRMS (ESI-TOF) m/z: calcd for $C_{17}H_{14}ClO_2^+$: 285.0677 $(M + H)^+$, found: 285.0678.

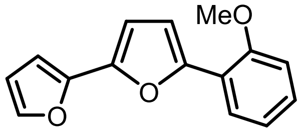

3ap

5-(2-methoxyphenyl)-2,2'-bifuran (**3ap**) (9.5 mg, 40%) was prepared from typical procedure (hexane) as black solid. ^{1}H NMR (500 MHz, Chloroform-d) δ 7.97 – 7.90 (m, 1H), 7.43 (d, J = 2.0 Hz, 1H), 7.28 – 7.22 (m, 1H), 7.07 – 7.02 (m, 1H), 7.01 (d, J = 3.3 Hz, 1H), 6.96 (d, J = 8.4 Hz, 1H), 6.68 – 6.61 (m, 2H), 6.51 – 6.45 (m, 1H), 3.94 (s, 3H); ^{13}C NMR (126 MHz, Chloroform-d) δ 155.4, 149.5, 146.8, 145.0, 141.7, 128.1, 125.8, 120.7, 119.5, 111.8, 111.4, 110.9, 107.4, 105.0, 55.4; HRMS (ESI-TOF) m/z: calcd for $C_{15}H_{13}O_3^+$: 241.0859 $(M + H)^+$, found: 241.0860.

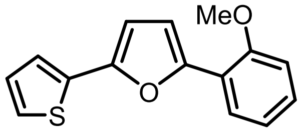

3aq

2-(2-methoxyphenyl)-5-(thiophen-2-yl)furan (**3aq**) (15.4 mg, 60%) was prepared from typical procedure (hexane) as dark brown solid. ^{1}H NMR (500 MHz, Chloroform-d) δ 7.95 – 7.91 (m, 1H), 7.33 – 7.31 (m, 1H), 7.26 – 7.24 (m, 1H), 7.23 – 7.21 (m, 1H), 7.07 – 7.03 (m, 2H), 7.00 (d, J = 3.5 Hz, 1H), 6.96 (d, J = 8.2 Hz, 1H), 6.60 (d, J = 3.4 Hz, 1H), 3.95 (s, 3H); ^{13}C NMR (126 MHz, Chloroform-d) δ 155.4, 149.3, 147.9, 134.0, 128.0, 127.6, 125.7, 123.9, 122.4, 120.7, 119.5, 112.1, 110.9, 107.3, 55.4; HRMS (ESI-TOF) m/z: calcd for $C_{15}H_{13}O_2S^+$: 257.0631 (M + H)$^+$, found: 257.0635.

2.3.2 Procedure for Synthesis of 3ba-3bz

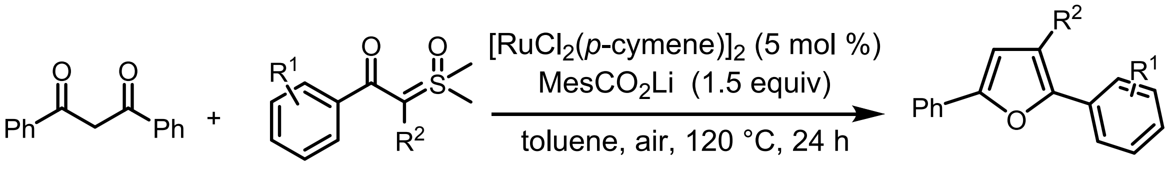

A dried 10 mL Schlenk tube was charged with **1,3-diphenylpropane-1,3-dione 1a** (22.4 mg, 0.1 mmol, 1 equiv), **sulfoxonium ylide 2** (0.2 mmol, 2 equiv), $[RuCl_2(p\text{-cymene})]_2$ (3.1 mg, 0.005 mmol, 5 mol %), $MesCO_2Li$ (25.5 mg, 0.15 mmol, 1.5 equiv), and toluene (2 mL). The reaction mixture was heated to 120 °C for 24 hours under vigorous stirring. Upon completion, the reaction mixture was cooled to room temperature, diluted with ethyl acetate, and filtered through a pad of celite. The filtrate was concentrated under vacuum, and the resulting residue was purified by preparative thin layer chromatography (PTLC) with hexane to give the corresponding products.

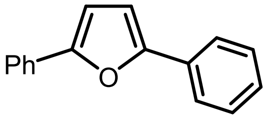

3ba

2,5-diphenylfuran (**3ba**) (9.2 mg, 42%) was prepared from typical procedure (hexane) as beige solid. ^{1}H NMR (500 MHz, Chloroform-d) δ 7.79 – 7.74 (m, 4H), 7.42 (t, J = 7.8 Hz, 4H), 7.30 – 7.26 (m, 2H), 6.75 (s, 2H); ^{13}C NMR (126 MHz, Chloroform-d) δ 153.3, 130.7, 128.7, 127.3, 123.7, 107.2; HRMS (ESI-TOF) m/z: calcd for $C_{16}H_{13}O^+$: 221.0961 (M + H)$^+$, found: 221.0957.

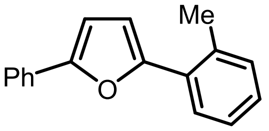

3bb

2-phenyl-5-(*o*-tolyl)furan (**3bb**) (17 mg, 73%) was prepared from typical procedure (hexane) as yellow oil. ^{1}H NMR (500 MHz, Chloroform-d) δ 7.81 (d, J = 7.8 Hz, 1H), 7.78 – 7.75 (m, 2H), 7.42 (t, J = 7.8 Hz, 2H), 7.31 (d, J = 6.7 Hz, 1H), 7.28 (d, J = 7.4 Hz, 2H), 7.25 – 7.21 (m, 1H), 6.79 (d, J = 3.5 Hz, 1H), 6.66 (d, J = 3.4 Hz, 1H), 2.59 (s, 3H); ^{13}C NMR (126 MHz, Chloroform-d) δ 153.1, 153.0,

134.4, 131.3, 130.8, 130.0, 128.7, 127.4, 127.3, 126.8, 126.0, 123.7, 110.6, 106.9, 22.1; HRMS (ESI-TOF) m/z: calcd for $C_{17}H_{15}O^{+}$: 235.1117 $(M + H)^{+}$, found: 235.1117.

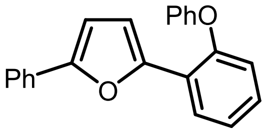

3bc

2-(2-phenoxyphenyl)-5-phenylfuran (**3bc**) (16.4 mg, 53%) was prepared from typical procedure (hexane) as yellow oil. ^{1}H NMR (500 MHz, Chloroform-d) δ 8.13 – 8.08 (m, 1H), 7.79 – 7.74 (m, 2H), 7.43 (t, J = 7.8 Hz, 2H), 7.41 – 7.36 (m, 2H), 7.32 – 7.28 (m, 1H), 7.28 – 7.23 (m, 2H), 7.17 – 7.12 (m, 1H), 7.10 – 7.06 (m, 2H), 7.02 (d, J = 3.5 Hz, 1H), 7.01 – 6.98 (m, 1H), 6.75 (d, J = 3.5 Hz, 1H); ^{13}C NMR (126 MHz, Chloroform-d) δ 156.9, 152.7, 152.4, 149.0, 130.7, 129.8, 128.7, 128.0, 127.3, 126.2, 123.9, 123.8, 123.2, 122.8, 119.8, 118.3, 112.5, 107.5; HRMS (ESI-TOF) m/z: calcd for $C_{22}H_{17}O_{2}^{+}$: 313.1223 $(M + H)^{+}$, found: 313.1226.

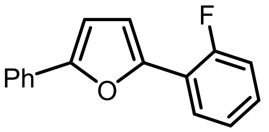

3bd

2-(2-fluorophenyl)-5-phenylfuran (**3bd**) (14.2 mg, 60%) was prepared from typical procedure (hexane) as yellow oil. ^{1}H NMR (500 MHz, Chloroform-d) δ 8.00 – 7.91 (m, 1H), 7.80 – 7.74 (m, 2H), 7.43 (t, J = 7.8 Hz, 2H), 7.32 – 7.28 (m, 1H), 7.26 – 7.21 (m, 2H), 7.17 – 7.11 (m, 1H), 6.95 (t, J = 3.6 Hz, 1H), 6.79 (d, J = 3.5 Hz, 1H); ^{13}C NMR (126 MHz, Chloroform-d) δ 158.7 (d, J = 250.7 Hz), 153.2, 147.4 (d, J = 3.1 Hz), 130.5, 128.7, 128.2 (d, J = 8.4Hz), 127.5, 125.8 (d, J = 3.2 Hz), 124.3 (d, J = 3.5 Hz), 123.9, 119.0 (d, J = 12.0 Hz), 115.9 (d, J = 21.4 Hz), 112.4 (d, J = 12.1 Hz), 107.5 (d, J = 1.8 Hz); HRMS (ESI-TOF) m/z: calcd for $C_{16}H_{12}FO^{+}$: 239.0867 $(M + H)^{+}$, found: 239.0866.

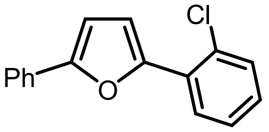

3be

2-(2-chlorophenyl)-5-phenylfuran (**3be**) (10.6 mg, 42%) was prepared from typical procedure (hexane) as yellow white solid. ^{1}H NMR (500 MHz, Chloroform-d) δ8.01 – 7.97 (m, 1H), 7.79 – 7.75 (m, 2H), 7.47 – 7.45 (m, 1H), 7.42 (t, J = 7.8 Hz, 2H), 7.38 – 7.33 (m, 1H), 7.32 – 7.28 (m, 1H), 7.24 (d, J = 3.5 Hz, 1H), 7.23 – 7.19 (m, 1H), 6.80 (d, J = 3.6 Hz, 1H); ^{13}C NMR (126 MHz, Chloroform-d) δ 153.3, 149.5, 130.8, 130.5, 130.0, 129.1, 128.7, 127.9, 127.7, 127.6, 126.9, 123.9, 113.2, 107.2; HRMS (ESI-TOF) m/z: calcd for $C_{16}H_{12}ClO^{+}$: 255.0571 $(M + H)^{+}$, found: 255.0572.

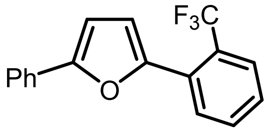

3bf

2-phenyl-5-(2-(trifluoromethyl)phenyl)furan (**3bf**) (13.5 mg, 47%) was prepared from typical procedure (hexane) as yellow oil. ^{1}H NMR (500 MHz, Chloroform-d) δ 7.84 (d, J = 7.9 Hz, 1H), 7.79 (d, J = 8.1 Hz, 1H), 7.79 – 7.73 (m, 2H), 7.60 (t, J = 7.6 Hz, 1H), 7.46 – 7.38 (m, 3H), 7.30 (t, J = 7.4 Hz, 1H), 6.82 (d, J = 3.5 Hz, 1H), 6.78 (d, J = 3.5 Hz, 1H); ^{13}C NMR (126 MHz, Chloroform-d) δ154.5, 150.0, 131.7, 130.5, 129.6, 129.5 (d, *J* = 2.1 Hz), 128.7, 127.8, 127.5, 127.3, 126.2 (d, *J* = 31.3 Hz), 124.1 (q, J = 273.3 Hz), 123.9, 112.2 (q, *J* = 3.1 Hz), 107.0; HRMS (ESI-TOF) m/z: calcd for $C_{17}H_{12}F_3O^+$: 289.0835 $(M + H)^+$, found: 289.0838.

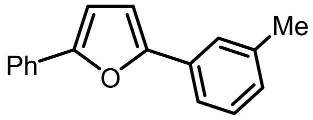

3bg

2-phenyl-5-(*m*-tolyl)furan (**3bg**) (12.1 mg, 52%) was prepared from typical procedure (hexane) as cream solid. ^{1}H NMR (500 MHz, Chloroform-d) δ 7.79 – 7.73 (m, 2H), 7.60 – 7.53 (m, 2H), 7.42 (t, J = 7.8 Hz, 2H), 7.34 – 7.24 (m, 2H), 7.10 (d, J = 7.5 Hz, 1H), 6.74 (q, J = 3.4 Hz, 2H), 2.42 (s, 3H); ^{13}C NMR (126 MHz, Chloroform-d) δ 153.5, 153.2, 138.3, 130.8, 130.7, 128.7, 128.6, 128.2, 127.3, 124.3, 123.7, 120.9, 107.2, 107.1, 21.5; HRMS (ESI-TOF) m/z: calcd for $C_{17}H_{15}O^+$: 235.1117 $(M + H)^+$, found: 235.1120.

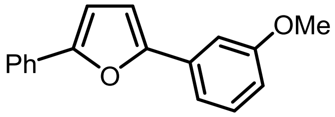

3bh

2-(3-methoxyphenyl)-5-phenylfuran (**3bh**) (9.4 mg, 38%) was prepared from typical procedure (hexane) as yellow oil. ^{1}H NMR (500 MHz, Chloroform-d) δ7.77 – 7.74 (m, 2H), 7.44 – 7.39 (m, 2H), 7.37 – 7.32 (m, 2H), 7.32 – 7.26 (m, 2H), 6.86 – 6.82 (m, 1H), 6.75 (s, 2H), 3.89 (s, 3H); ^{13}C NMR (126 MHz, Chloroform-d) δ 159.9, 153.4, 153.1, 132.0, 130.7, 129.8, 128.7, 127.4, 123.7, 116.4, 112.9, 109.2, 107.6, 107.2, 55.3; HRMS (ESI-TOF) m/z: calcd for $C_{17}H_{15}O_2^+$: 251.1067 $(M + H)^+$, found: 251.1070.

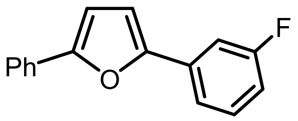

3bi

2-(3-fluorophenyl)-5-phenylfuran (**3bi**) (8.8 mg, 37%) was prepared from typical procedure (hexane) as cream solid. ^{1}H NMR (500 MHz, Chloroform-d) δ 7.79 – 7.72 (m, 2H), 7.51 (d, J = 7.8 Hz, 1H), 7.48 – 7.39 (m, 3H), 7.39 – 7.34 (m, 1H), 7.33 – 7.27 (m, 1H), 7.00 – 6.92 (m, 1H), 6.78 – 6.74 (m, 2H); ^{13}C

NMR (126 MHz, Chloroform-d) δ163.2 (d, J = 245.1 Hz), 153.8, 152.0 (d, J = 3.1 Hz), 132.8 (d, J = 8.5 Hz), 130.5, 130.3 (d, J = 8.5 Hz), 128.7, 127.6, 123.8, 119.3 (d, J = 2.9 Hz), 114.0 (d, J = 21.5 Hz), 110.5 (d, J = 23.5 Hz), 108.3, 107.3; HRMS (ESI-TOF) m/z: calcd for $C_{16}H_{12}FO^+$: 239.0867 $(M + H)^+$, found: 239.0868.

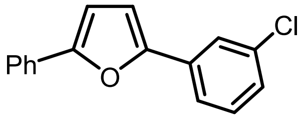

3bj

2-(3-chlorophenyl)-5-phenylfuran (**3bj**) (11.9 mg, 47%) was prepared from typical procedure (hexane) as white solid. ^{1}H NMR (500 MHz, Chloroform-d) δ7.77 – 7.74 (m, 2H), 7.73 (t, J = 1.9 Hz, 1H), 7.61 (m, 1H), 7.45 – 7.40 (m, 2H), 7.33 (t, J = 7.9 Hz, 1H), 7.31 – 7.28 (m, 1H), 7.25 – 7.22 (m, 1H), 6.78 – 6.74 (m, 2H); ^{13}C NMR (126 MHz, Chloroform-d) δ 153.9, 151.8, 134.7, 132.4, 130.4, 130.0, 128.7, 127.6, 127.1, 123.8, 123.6, 121.7, 108.3, 107.3; HRMS (ESI-TOF) m/z: calcd for $C_{16}H_{12}ClO^+$: 255.0571 $(M + H)^+$, found: 255.0573.

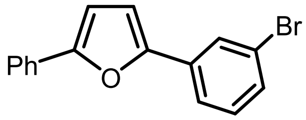

3bk

2-(3-bromophenyl)-5-phenylfuran (**3bk**) (16.8 mg, 56%) was prepared from typical procedure (hexane) as cream solid. ^{1}H NMR (500 MHz, Chloroform-d) δ 7.88 (t, J = 1.8 Hz, 1H), 7.79 – 7.72 (m, 2H), 7.67 – 7.64 (m, 1H), 7.45 – 7.40 (m, 2H), 7.40 – 7.37 (m, 1H), 7.32 – 7.28 (m, 1H), 7.27 – 7.25 (m, 1H), 6.77 – 6.74 (m, 2H); ^{13}C NMR (126 MHz, Chloroform-d) δ 154.0, 151.6, 132.6, 130.4, 130.2, 130.1, 128.7, 127.6, 126.5, 123.8, 122.9, 122.1, 108.3, 107.2; HRMS (ESI-TOF) m/z: calcd for $C_{16}H_{12}BrO^+$: 299.0066 $(M + H)^+$, found: 299.0065.

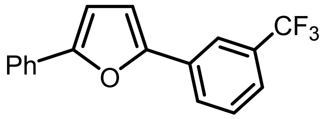

3bl

2-phenyl-5-(3-(trifluoromethyl)phenyl)furan (**3bl**) (18.1 mg, 63%) was prepared from typical procedure (hexane) as yellowish solid. ^{1}H NMR (500 MHz, Chloroform-d) δ 7.97 (s, 1H), 7.93 – 7.87 (m, 1H), 7.77 (d, J = 7.4 Hz, 2H), 7.57 – 7.48 (m, 2H), 7.44 (t, J = 7.8 Hz, 2H), 7.31 (t, J = 7.5 Hz, 1H), 6.83 (d, J = 3.5 Hz, 1H), 6.77 (d, J = 3.5 Hz, 1H); ^{13}C NMR (126 MHz, Chloroform-d) δ154.2, 151.7, 131.4, 131.2 (q, J = 32.3 Hz), 130.3, 129.2, 128.8, 127.7, 126.6, 124.1 (q, J = 272.4 Hz), 123.9, 123.7 (d, J = 4.9 Hz), 120.3 (d, J = 4.0 Hz), 108.6, 107.3; HRMS (ESI-TOF) m/z: calcd for $C_{17}H_{12}F_3O^+$: 289.0835 $(M + H)^+$, found: 289.0836.

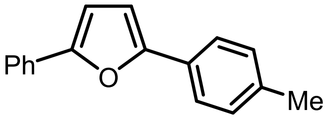

3bm

2-phenyl-5-(*p*-tolyl)furan (**3bm**) (11.9 mg, 51%) was prepared from typical procedure (hexane) as pale yellow solid. ^{1}H NMR (500 MHz, Chloroform-d) δ 7.64 – 7.60 (m, 2H), 7.52 (d, J = 8.1 Hz, 2H), 7.27 (t, J = 7.7 Hz, 2H), 7.16 – 7.11 (m, 1H), 7.09 (d, J = 7.9 Hz, 2H), 6.62 – 6.59 (m, 1H), 6.57 – 6.53 (m, 1H), 2.25 (s, 3H); ^{13}C NMR (126 MHz, Chloroform-d) δ 153.6, 152.9, 137.2, 130.8, 129.4, 128.7, 128.1, 127.2, 123.7, 123.6, 107.2, 106.5, 21.3; HRMS (ESI-TOF) m/z: calcd for $C_{17}H_{15}O^{+}$: 235.1117 $(M + H)^{+}$, found: 235.1121.

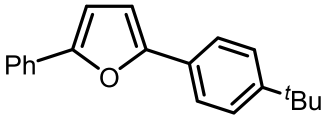

3bn

2-(4-(*tert*-butyl)phenyl)-5-phenylfuran (**3bn**) (14.4 mg, 52%) was prepared from typical procedure (hexane) as white solid. ^{1}H NMR (500 MHz, Chloroform-d) δ 7.77 – 7.74 (m, 2H), 7.71 – 7.67 (m, 2H), 7.46 – 7.43 (m, 2H), 7.42 (t, J = 7.9 Hz, 2H), 7.30 – 7.25 (m, 1H), 6.76 – 6.73 (m, 1H), 6.71 – 6.68 (m, 1H), 1.36 (s, 9H); ^{13}C NMR (126 MHz, Chloroform-d) δ 153.5, 153.0, 150.4, 130.9, 128.7, 128.1, 127.2, 125.6, 123.6, 123.5, 107.2, 106.6, 34.6, 31.3; HRMS (ESI-TOF) m/z: calcd for $C_{20}H_{21}O^{+}$: 277.1587 $(M + H)^{+}$, found: 277.1586.

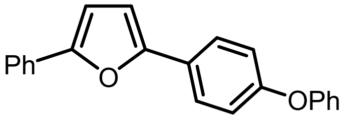

3bo

2-(4-phenoxyphenyl)-5-phenylfuran (**3bo**) (14.1 mg, 45%) was prepared from typical procedure (hexane) as yellowish solid. ^{1}H NMR (500 MHz, Chloroform-d) δ 7.78 – 7.69 (m, 4H), 7.41 (t, J = 7.8 Hz, 2H), 7.39 – 7.35 (m, 2H), 7.30 – 7.25 (m, 1H), 7.14 (t, J = 7.4 Hz, 1H), 7.10 – 7.03 (m, 4H), 6.74 (d, J = 3.5 Hz, 1H), 6.67 (d, J = 3.4 Hz, 1H); ^{13}C NMR (126 MHz, Chloroform-d) δ 157.0, 156.6, 153.1, 153.0, 130.7, 129.8, 128.7, 127.2, 126.2, 125.2, 123.6, 123.4, 119.1, 118.9, 107.2, 106.5; HRMS (ESI-TOF) m/z: calcd for $C_{22}H_{17}O_{2}^{+}$: 313.1223 $(M + H)^{+}$, found: 313.1228.

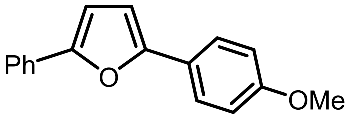

3bp

2-(4-methoxyphenyl)-5-phenylfuran (**3bp**) (13.8 mg, 55%) was prepared from typical procedure (hexane) as white solid. ^{1}H NMR (500 MHz, Chloroform-d) δ7.75 – 7.72 (m, 2H), 7.71 – 7.67 (m, 2H), 7.40 (t, J = 7.8 Hz, 2H), 7.30 – 7.22 (m, 1H), 6.97 – 6.94 (m, 2H), 6.72 (d, J = 3.4 Hz, 1H), 6.61 (d, J = 3.4 Hz, 1H), 3.85 (s, 3H); ^{13}C NMR (126 MHz, Chloroform-d) δ 159.0, 153.4, 152.6, 130.9, 128.6,

127.1, 125.2, 123.9, 123.5, 114.1, 107.2, 105.6, 55.3; HRMS (ESI-TOF) m/z: calcd for $C_{17}H_{15}O_2^+$: 251.1067 $(M + H)^+$, found: 251.1070.

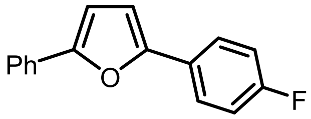

3bq

2-(4-fluorophenyl)-5-phenylfuran (**3bq**) (13.8 mg, 58%) was prepared from typical procedure (hexane) as cream solid. ^{1}H NMR (500 MHz, Chloroform-d) δ 7.79 – 7.68 (m, 4H), 7.42 (t, J = 7.8 Hz, 2H), 7.32 – 7.25 (m, 1H), 7.14 – 7.08 (m, 2H), 6.75 – 6.72 (m, 1H), 6.68 – 6.66 (m, 1H); ^{13}C NMR (126 MHz, Chloroform-d) δ 162.1 (d, J = 247.1 Hz), 153.3, 152.5, 130.6, 128.7, 127.4, 127.3, 127.1 (d, J = 3.5 Hz), 125.4 (d, J = 7.9 Hz), 123.7, 123.6, 115.7 (d, J = 21.9 Hz), 107.2, 106.8 (d, J = 1.6 Hz); HRMS (ESI-TOF) m/z: calcd for $C_{16}H_{12}FO^+$: 239.0867 $(M + H)^+$, found: 239.0869.

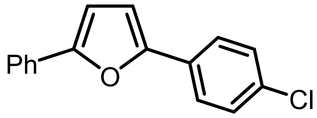

3br

2-(4-chlorophenyl)-5-phenylfuran (**3br**) (12.9 mg, 51%) was prepared from typical procedure (hexane) as white solid. ^{1}H NMR (500 MHz, Chloroform-d) δ 7.79 – 7.71 (m, 2H), 7.70 – 7.62 (m, 2H), 7.42 (t, J = 7.7 Hz, 2H), 7.37 (d, J = 8.6 Hz, 2H), 7.29 (t, J = 7.4 Hz, 1H), 6.74 (q, J = 3.5 Hz, 2H); ^{13}C NMR (126 MHz, Chloroform-d) δ 153.6, 152.2, 132.9, 130.5, 129.2, 128.9, 128.7, 127.5, 124.9, 123.7, 107.7, 107.3; HRMS (ESI-TOF) m/z: calcd for $C_{16}H_{12}ClO^+$: 255.0571 $(M + H)^+$, found: 255.0570.

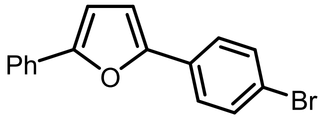

3bs

2-(4-bromophenyl)-5-phenylfuran (**3bs**) (13.2 mg, 44%) was prepared from typical procedure (hexane) as white solid. ^{1}H NMR (500 MHz, Chloroform-d) δ 7.77 – 7.71 (m, 2H), 7.62 – 7.59 (m, 2H), 7.54 – 7.51 (m, 2H), 7.42 (t, J = 7.8 Hz, 2H), 7.31 – 7.27 (m, 1H), 6.75 – 6.72 (m, 2H); ^{13}C NMR (126 MHz, Chloroform-d) δ 153.7, 152.2, 131.8, 130.5, 129.6, 128.7, 127.5, 125.1, 123.7, 121.0, 107.8, 107.3; HRMS (ESI-TOF) m/z: calcd for $C_{16}H_{12}BrO^+$: 299.0066 $(M + H)^+$, found: 299.0067.

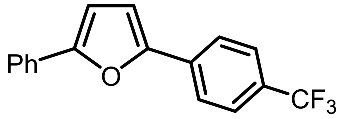

3bt

2-phenyl-5-(4-(trifluoromethyl)phenyl)furan (**3bt**) (15.2 mg, 53%) was prepared from typical procedure (hexane) as light yellow solid. ^{1}H NMR (500 MHz, Chloroform-d) δ 7.83 (d, J = 8.1 Hz, 2H), 7.79 – 7.73 (m, 2H), 7.65 (d, J = 8.2 Hz, 2H), 7.43 (t, J = 7.8 Hz, 2H), 7.34 – 7.29 (m, 1H), 6.86 (d, J = 3.5 Hz, 1H), 6.77 (d, J = 3.5 Hz, 1H); ^{13}C NMR (126 MHz, Chloroform-d) δ154.4, 151.7, 133.8, 130.3,

128.81 (q, *J* = 31.5 Hz), 128.78, 127.8, 125.7 (q, *J* = 3.9 Hz), 124.2 (q, *J* = 271.8 Hz), 123.9, 123.6, 109.2, 107.4; HRMS (ESI-TOF) m/z: calcd for $C_{17}H_{12}F_3O^+$: 289.0835 $(M + H)^+$, found: 289.0839.

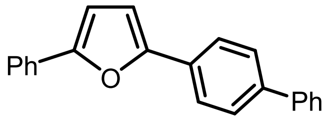

3bu

2-([1,1'-biphenyl]-4-yl)-5-phenylfuran (**3bu**) (17.7 mg, 60%) was prepared from typical procedure (hexane) as colorless solid. ^1H NMR (500 MHz, Chloroform-d) δ 7.86 – 7.82 (m, 2H), 7.81 – 7.75 (m, 2H), 7.70 – 7.62 (m, 4H), 7.47 (t, J = 7.7 Hz, 2H), 7.43 (t, J = 7.8 Hz, 2H), 7.37 (t, J = 7.4 Hz, 1H), 7.29 (t, J = 7.4 Hz, 1H), 6.81 – 6.76 (m, 2H); ^{13}C NMR (126 MHz, Chloroform-d) δ 153.4, 153.1, 140.6, 139.9, 130.7, 129.7, 128.8, 128.7, 127.4, 126.9, 124.1, 123.7, 107.4, 107.3; HRMS (ESI-TOF) m/z: calcd for $C_{22}H_{17}O^+$: 297.1274 $(M + H)^+$, found: 297.1274.

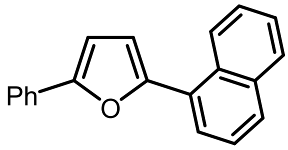

3bv

2-(naphthalen-1-yl)-5-phenylfuran (**3bv**) (9.2 mg, 34%) was prepared from typical procedure (hexane) as yellow oil. ^1H NMR (500 MHz, Chloroform-d) δ 8.54 (d, J = 8.6 Hz, 1H), 7.93 – 7.90 (m, 1H), 7.86 (d, J = 8.3 Hz, 1H), 7.85 – 7.83 (m, 1H), 7.83 – 7.77 (m, 2H), 7.60 – 7.56 (m, 1H), 7.56 – 7.52 (m, 2H), 7.46 – 7.41 (m, 2H), 7.32 – 7.28 (m, 1H), 6.87 (d, J = 3.4 Hz, 1H), 6.84 (d, J = 3.4 Hz, 1H); ^{13}C NMR (126 MHz, Chloroform-d) δ 153.8, 152.9, 134.0, 130.8, 130.2, 128.7, 128.6, 128.5, 128.4, 127.4, 126.6, 126.0, 125.9, 125.5, 125.3, 123.7, 111.4, 106.9; HRMS (ESI-TOF) m/z: calcd for $C_{20}H_{15}O^+$: 271.1117 $(M + H)^+$, found: 271.1115.

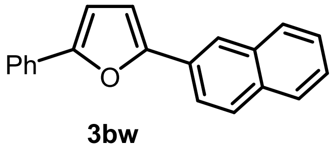

3bw

2-(naphthalen-2-yl)-5-phenylfuran (**3bw**) (12.1 mg, 45%) was prepared from typical procedure (hexane) as yellow solid. ^1H NMR (500 MHz, Chloroform-d) δ 8.23 (s, 1H), 7.91 (d, J = 8.0 Hz, 1H), 7.87 (d, J = 8.6 Hz, 1H), 7.87 – 7.79 (m, 4H), 7.53 – 7.47 (m, 2H), 7.47 – 7.43 (m, 2H), 7.33 – 7.29 (m, 1H), 6.87 (d, J = 3.5 Hz, 1H), 6.80 (d, J = 3.5 Hz, 1H); ^{13}C NMR (126 MHz, Chloroform-d) δ 153.6, 153.4, 133.6, 132.7, 130.7, 128.7, 128.4, 128.1, 128.0, 127.8, 127.4, 126.5, 125.9, 123.8, 122.2, 121.9, 107.9, 107.4; HRMS (ESI-TOF) m/z: calcd for $C_{20}H_{15}O^+$: 271.1117 $(M + H)^+$, found: 271.1118.

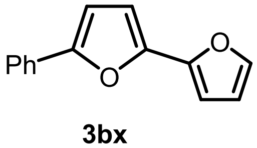

3bx

5-phenyl-2,2'-bifuran (**3bx**) (11.4 mg, 54%) was prepared from typical procedure (hexane) as reddish black oil. ^{1}H NMR (500 MHz, Chloroform-d) δ 7.75 – 7.69 (m, 2H), 7.44 (s, 1H), 7.40 (t, J = 7.8 Hz, 2H), 7.31 – 7.24 (m, 1H), 6.72 (d, J = 3.5 Hz, 1H), 6.65 – 6.63 (m, 2H), 6.52 – 6.46 (m, 1H); ^{13}C NMR (126 MHz, Chloroform-d) δ 153.1, 146.5, 146.0, 141.8, 130.4, 128.7, 127.4, 123.7, 111.5, 107.2, 106.8, 105.2; HRMS (ESI-TOF) m/z: calcd for $C_{14}H_{11}O_2^+$: 211.0754 (M + H)$^+$, found: 211.0759.

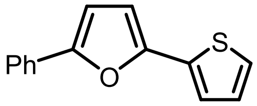

3by

2-phenyl-5-(thiophen-2-yl)furan (**3by**) (12.5 mg, 55%) was prepared from typical procedure (hexane) as black solid. ^{1}H NMR (500 MHz, Chloroform-d) δ 7.76 – 7.69 (m, 2H), 7.41 (t, J = 7.8 Hz, 2H), 7.34 – 7.31 (m, 1H), 7.30 – 7.26 (m, 1H), 7.26 – 7.23 (m, 1H), 7.10 – 7.04 (m, 1H), 6.71 (d, J = 3.4 Hz, 1H), 6.59 (d, J = 3.5 Hz, 1H). ^{13}C NMR (126 MHz, Chloroform-d) δ 152.9, 148.9, 133.7, 130.5, 128.7, 127.7, 127.4, 124.1, 123.7, 122.5, 107.2, 107.1; HRMS (ESI-TOF) m/z: calcd for $C_{14}H_{11}OS^+$: 227.0525 (M + H)$^+$, found: 227.0529.

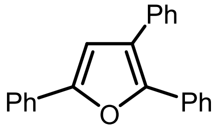

3bz

2,3,5-triphenylfuran (**3bz**) (7.9 mg, 27%) as a white solid. ^{1}H NMR (500 MHz, Chloroform-d) δ 7.80 – 7.73 (m, 2H), 7.63 – 7.59 (m, 2H), 7.49 – 7.46 (m, 2H), 7.44 – 7.37 (m, 4H), 7.35 – 7.28 (m, 4H), 7.28 – 7.23 (m, 1H), 6.82 (s, 1H); ^{13}C NMR (126 MHz, Chloroform-d) δ 152.5, 147.9, 134.3, 131.1, 130.5, 128.7, 128.7, 128.7, 128.4, 127.5, 127.5, 127.3, 126.1, 124.5, 123.8, 109.4; HRMS (ESI-TOF) m/z: calcd for $C_{22}H_{17}O^+$: 297.1274 (M + H)$^+$, found: 297.1275.

2.3.3 Procedure for Synthesis of 3ca

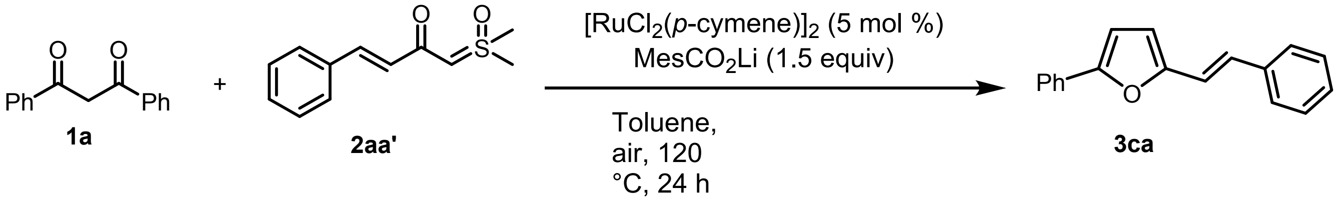

A dried 10 mL Schlenk tube was charged with **1,3-diphenylpropane-1,3-dione 1a** (22.4 mg, 0.1 mmol, 1 equiv), ***(E)*-1-(dimethyl(oxo)-λ^6-sulfanylidene)-4-phenylbut-3-en-2-one 2aa'** (44.5mg, 0.2 mmol), [RuCl$_2$(*p*-cymene)]$_2$ (3.1 mg, 0.005 mmol, 5 mol %), MesCO$_2$Li (25.5 mg, 0.15 mmol, 1.5 equiv), and toluene (2 mL). The reaction mixture was heated to 120 °C for 24 hours under vigorous stirring. Upon completion, the reaction mixture was cooled to room temperature, diluted with ethyl acetate, and filtered through a pad of celite. The filtrate was concentrated under vacuum, and the resulting residue

was purified by preparative thin layer chromatography (PTLC) with hexane to give the corresponding products (**3ca**).

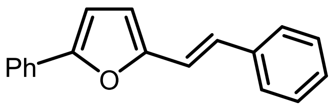

3ca

(*E*)-2-phenyl-5-styrylfuran (**3ca**) (6.7 mg, 27%) as a pale yellow solid. ^{1}H NMR (500 MHz, Chloroform-d) δ 7.77 – 7.71 (m, 2H), 7.50 (d, J = 7.3 Hz, 2H), 7.40 (t, J = 7.8 Hz, 2H), 7.36 (t, J = 7.7 Hz, 2H), 7.31 – 7.21 (m, 2H), 7.13 (d, J = 16.2 Hz, 1H), 6.92 (d, J = 16.3 Hz, 1H), 6.70 (d, J = 3.5 Hz, 1H), 6.44 (d, J = 3.5 Hz, 1H); ^{13}C NMR (126 MHz, Chloroform-d) δ 153.5, 152.8, 137.1, 130.6, 128.7, 127.5, 127.4, 126.9, 126.3, 123.8, 116.4, 111.1, 107.3; HRMS (ESI-TOF) m/z: calcd for $C_{18}H_{15}O^+$: 247.1117 $(M + H)^+$, found: 247.1115.

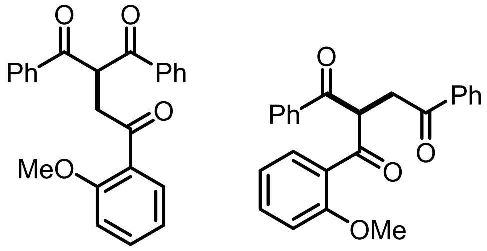

4a **5a**

^{1}H NMR (500 MHz, Chloroform-d) δ 8.10 – 8.05 (m, 2H), 8.05 – 7.98 (m, 3H), 7.96 – 7.90 (m, 2H), 7.62 – 7.55 (m, 3H), 7.53 – 7.42 (m, 6H), 7.02 (t, J = 7.6 Hz, 1H), 6.88 (d, J = 8.7 Hz, 1H), 6.18 – 6.09 (m, 1H), 4.03 – 3.97 (m, 1H), 3.86 (s, 0.5H), 3.80 (d, J = 6.4 Hz, 0.37H), 3.47 (s, 3H), 3.37 – 3.31 (m, 1H); ^{13}C NMR (126 MHz, Chloroform-d) δ 197.0, 196.8, 195.2, 158.6, 136.3, 135.6, 134.6, 133.3, 133.2, 131.5, 128.9, 128.8, 128.6, 128.6, 128.2, 125.6, 121.0, 111.4, 55.6, 54.9, 37.4; HRMS (ESI-TOF) m/z: calcd for $C_{24}H_{21}O_4^+$: 373.1434 $(M + H)^+$, found: 373.1436. (**4a**:**5a**=1:6)

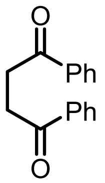

9

1,4-diphenylbutane-1,4-dione[5] (**9**) ^{1}H NMR (500 MHz, Chloroform-d) δ 8.07 – 8.03 (m, 4H), 7.61 – 7.56 (m, 2H), 7.49 (t, J = 7.7 Hz, 4H), 3.48 (s, 4H); ^{13}C NMR (126 MHz, Chloroform-d) δ 198.7, 136.7, 133.2, 128.6, 128.1, 32.6.

3.Procedure for Gram-Scale Experiment of 3aa

A dried 150 mL Schlenk tube was charged with **1,3-diphenylpropane-1,3-dione 1a** (1.0 g, 4.5 mmol, 1 equiv), **2-(dimethyl(oxo)-λ^6-sulfanylidene)-1-(2-methoxyphenyl)ethan-1-one 2a** (2.0 g, 9.0 mmol, 2

equiv), $[RuCl_2(p\text{-Cymene})]_2$ (137.8 mg, 0.225 mmol, 5 mol %), $MesCO_2Li$ (1.1g, 6.75 mmol, 1.5 equiv), and toluene (90 mL). The reaction mixture was heated to 120 °C for 48 hours under vigorous stirring. Upon completion, the reaction mixture was cooled to room temperature, diluted with ethyl acetate, and filtered through a pad of celite. The filtrate was concentrated under vacuum, and the resulting residue was purified by preparative thin layer chromatography (PTLC) with hexane to give the corresponding products **2-(2-methoxyphenyl)-5-phenylfuran** (**3aa**) (0.686 g, 61%) as a white solid.

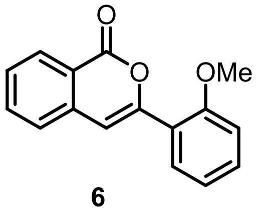

6

3-(2-methoxyphenyl)-*1H*-isochromen-1-one (**6**) (147.6 mg 13%) was also isolated as a yellow solid. ^{1}H NMR (500 MHz, Chloroform-d) δ 8.31 (d, J = 7.9 Hz, 1H), 8.01 – 7.96 (m, 1H), 7.75 – 7.67 (m, 1H), 7.53 – 7.46 (m, 2H), 7.41 – 7.37 (m, 2H), 7.08 (t, J = 7.6 Hz, 1H), 7.02 (d, J = 8.3 Hz, 1H), 3.97 (s, 3H). ^{13}C NMR (126 MHz, Chloroform-d) δ 162.7, 157.1, 150.4, 138.0, 134.6, 130.7, 129.4, 128.8, 128.0, 126.3, 120.8, 120.7, 120.6, 111.3, 107.0, 55.6; HRMS (ESI-TOF) m/z: calcd for $C_{16}H_{13}O_3^+$: 253.0859 $(M + H)^+$, found: 253.0857.

4.Procedure for Gram-Scale Experiment of 3ad

A dried 150 mL Schlenk tube was charged with **1,3-bis(4-fluorophenyl)propane-1,3-dione 1d** (1.3 g, 5.0 mmol, 1 equiv), **2-(dimethyl(oxo)-λ^6-sulfanylidene)-1-(2-methoxyphenyl)ethan-1-one 2a** (2.3 g, 10.0 mmol, 2 equiv), $[RuCl_2(p\text{-Cymene})]_2$ (153.1 mg, 0.25 mmol, 5 mol %), $MesCO_2Li$ (1.3g, 7.5 mmol, 1.5 equiv), and toluene (100 mL). The reaction mixture was heated to 120 °C for 48 hours under vigorous stirring. Upon completion, the reaction mixture was cooled to room temperature, diluted with ethyl acetate, and filtered through a pad of celite. The filtrate was concentrated under vacuum, and the resulting residue was purified by preparative thin layer chromatography (PTLC) with hexane to give the corresponding products **2-(4-fluorophenyl)-5-(2-methoxyphenyl)furan** (**3ad**) (0.890 g, 66%) as a white solid.

5. X-Ray Crystallographic Data of 3ak

Crystal structure and data of 2-(2-methoxyphenyl)-5-(3-(trifluoromethyl)phenyl)furan (**3ak**) (CCDC 1918495, Displacement ellipsoids are drawn at the 50% probability level.)

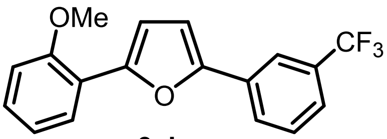

3ak

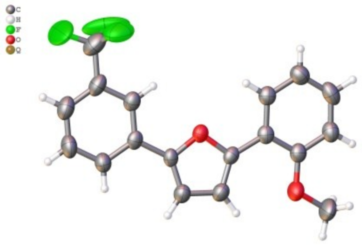

Table S3 Crystal data and structure refinement for 3ak.

Empirical formula	$C_{18}H_{13}F_3O_2$
Formula weight	318.28
Temperature/K	295.5(2)
Crystal system	monoclinic
Space group	$P2_1/n$
a/Å	9.501(3)
b/Å	16.731(5)
c/Å	9.633(2)
α/°	90.00
β/°	99.37(3)
γ/°	90.00
Volume/Å^3	1510.8(7)
Z	4
ρ_{calc}g/cm^3	1.346
μ/mm^{-1}	0.112
F(000)	632.0
Crystal size/mm^3	0.12 × 0.11 × 0.1
Radiation	MoKα (λ = 0.71073)
2Θ range for data collection/°	7.42 to 50
Index ranges	$-10 \le h \le 11, -19 \le k \le 19, -11 \le l \le 11$
Reflections collected	8734
Independent reflections	2659 [R_{int} = 0.1477, R_{sigma} = 0.1036]
Data/restraints/parameters	2659/34/237
Goodness-of-fit on F^2	0.971
Final R indexes [I>=2σ (I)]	R_1 = 0.0932, wR_2 = 0.2108
Final R indexes [all data]	R_1 = 0.1319, wR_2 = 0.2718
Largest diff. peak/hole/e Å^{-3}	0.43/-0.64

Reference

1 (*a*) R. M. P. Dias and A. C. B. Burtoloso, *Org. Lett.*, 2016, **18**, 3034; (*b*) A. M. Phelps, V. S. Chan, J. G. Napolitano, S. W. Krabbe, J. M. Schomaker and S. Shekhar, *J. Org. Chem.*, 2016, **81**, 4158; (*c*) M. Barday, C. Janot, N. R. Halcovitch, J. Muir and C. Aissa, *Angew. Chem. Int. Ed.*, 2017, **56**, 13117.

2 S.-S. Zhang, J.-Q. Wu, Y.-X. Lao, X.-G. Liu, Y. Liu, W.-X. Lv, D.-H. Tan, Y.-F. Zeng and H. Wang, *Org. Lett.*, 2014, **16**, 6412.

3 A. G. Talero, B. S. Martins and A. C. B. Burtoloso, *Org. Lett.*, 2018, **20**, 7206.

4 D. Lim, F. Fang, G. Zhou and D. M. Coltart, *Org. Lett.*, 2007, **9**, 4139.

5 Y. Peng, L. Luo, C.-S. Yan, J.-J. Zhang and Y.-W. Wang, *J. Org. Chem.*, 2013, **78**, 10960.

6. ^{1}H and ^{13}C NMR Spectra

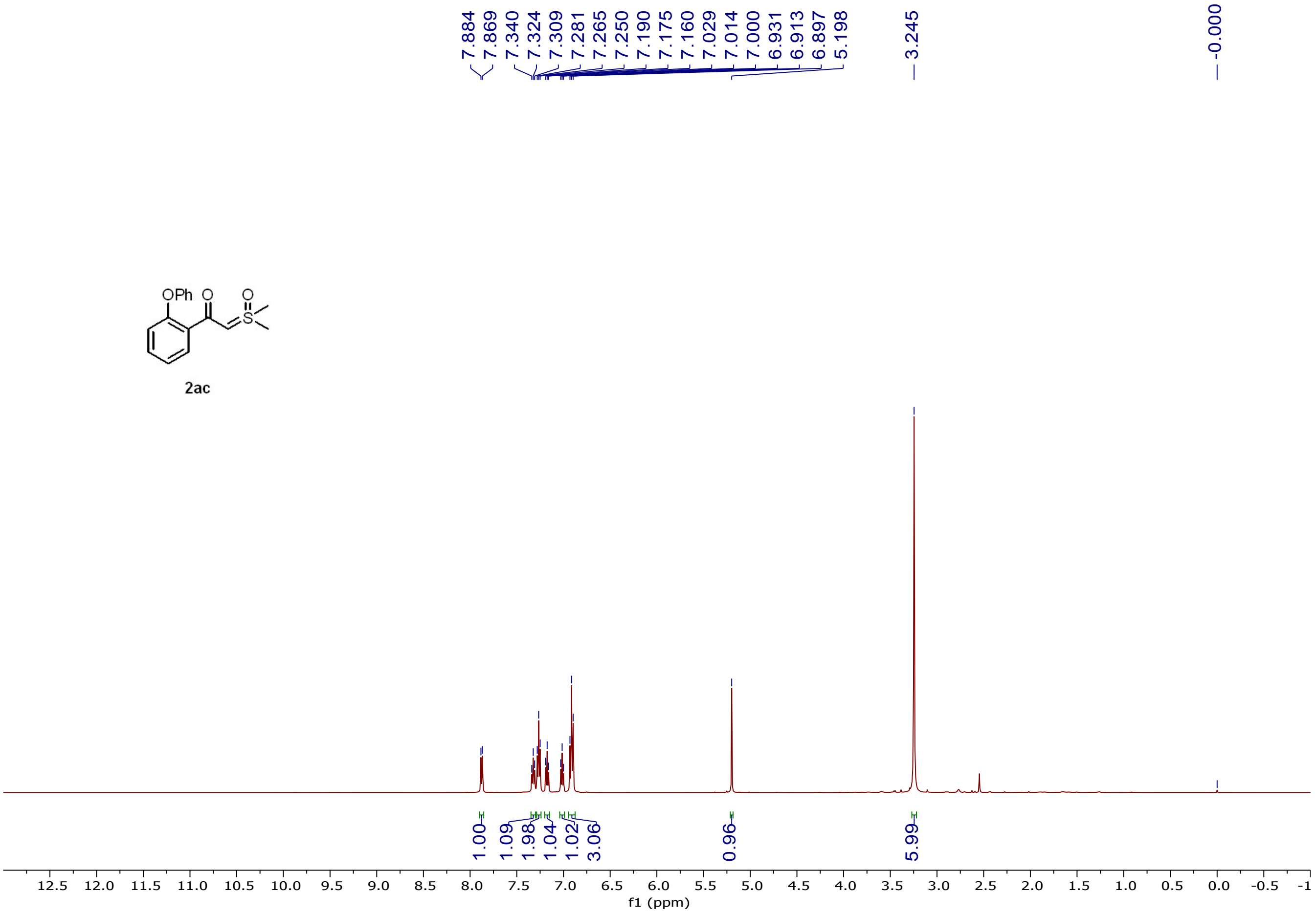

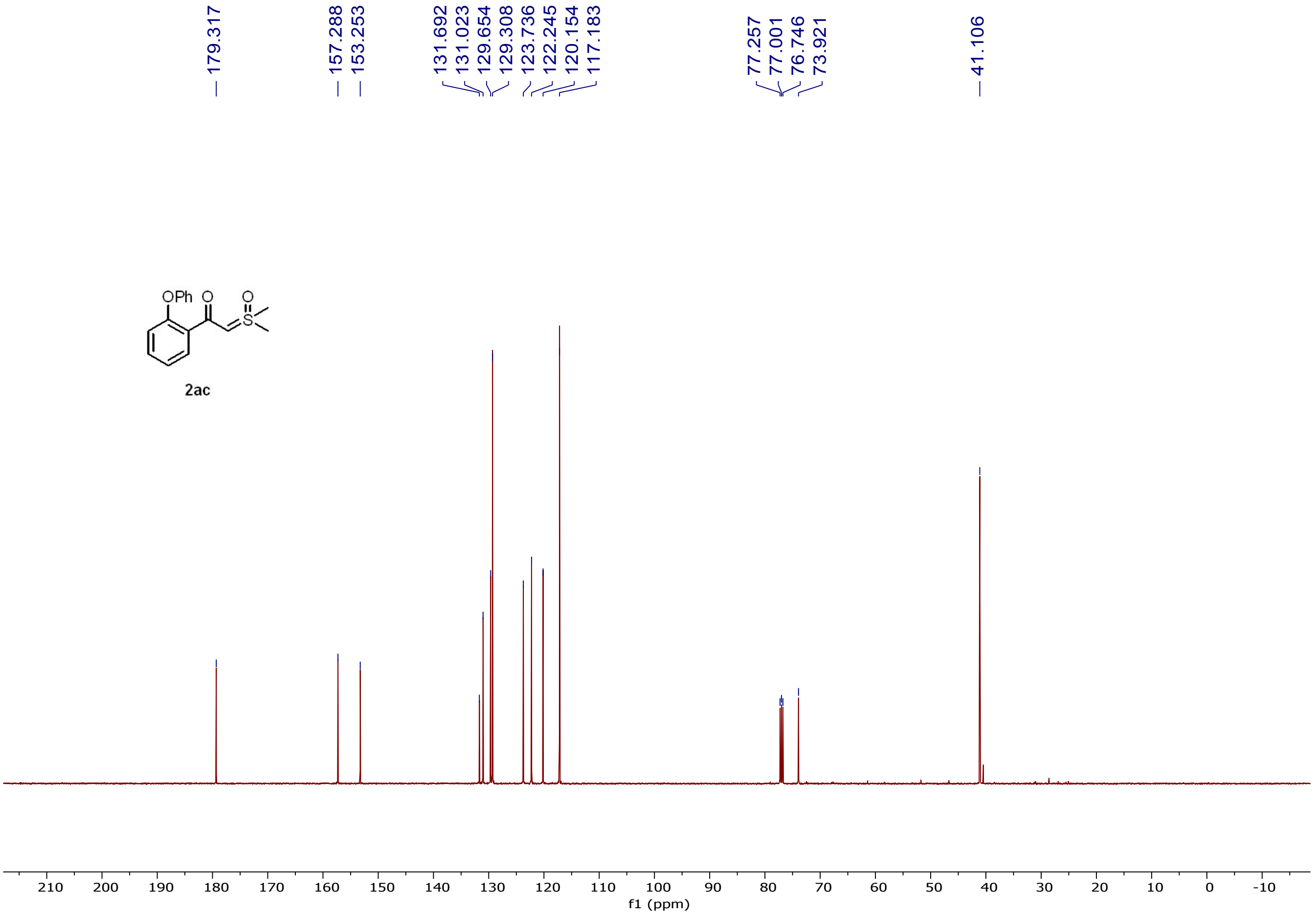

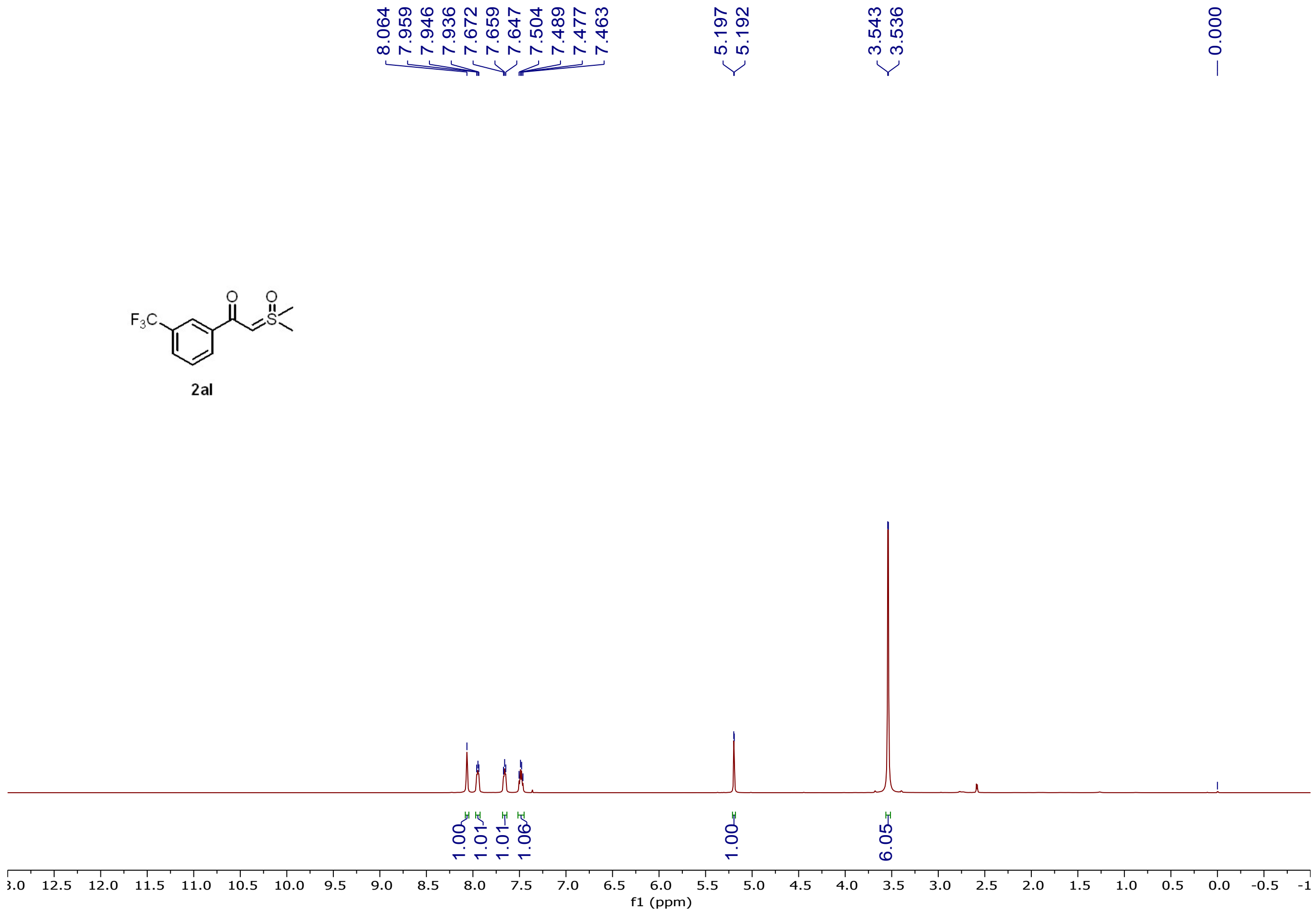

8.064
7.959
7.946
7.936
7.672
7.659
7.647
7.504
7.489
7.477
7.463
5.197
5.192
3.543
3.536
0.000
F3C
2al
1.00
1.01
1.01
1.06
1.00
6.05
f1 (ppm)

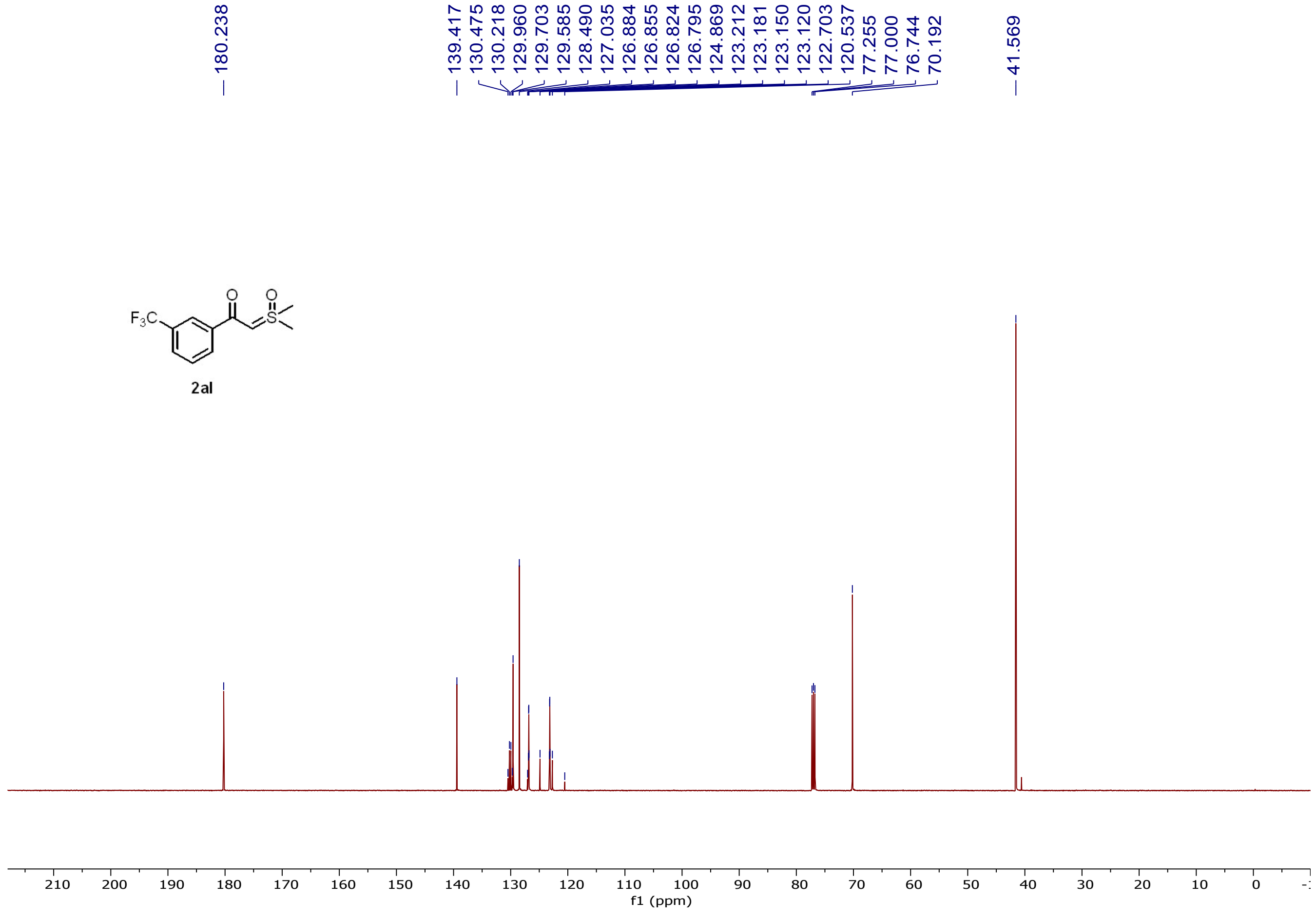

180.238
139.417
130.475
130.218
129.960
129.703
129.585
128.490
127.035
126.884
126.855
126.824
126.795
124.869
123.212
123.181
123.150
123.120
122.703
120.537
77.255
77.000
76.744
70.192
41.569
F3C
2al
f1 (ppm)

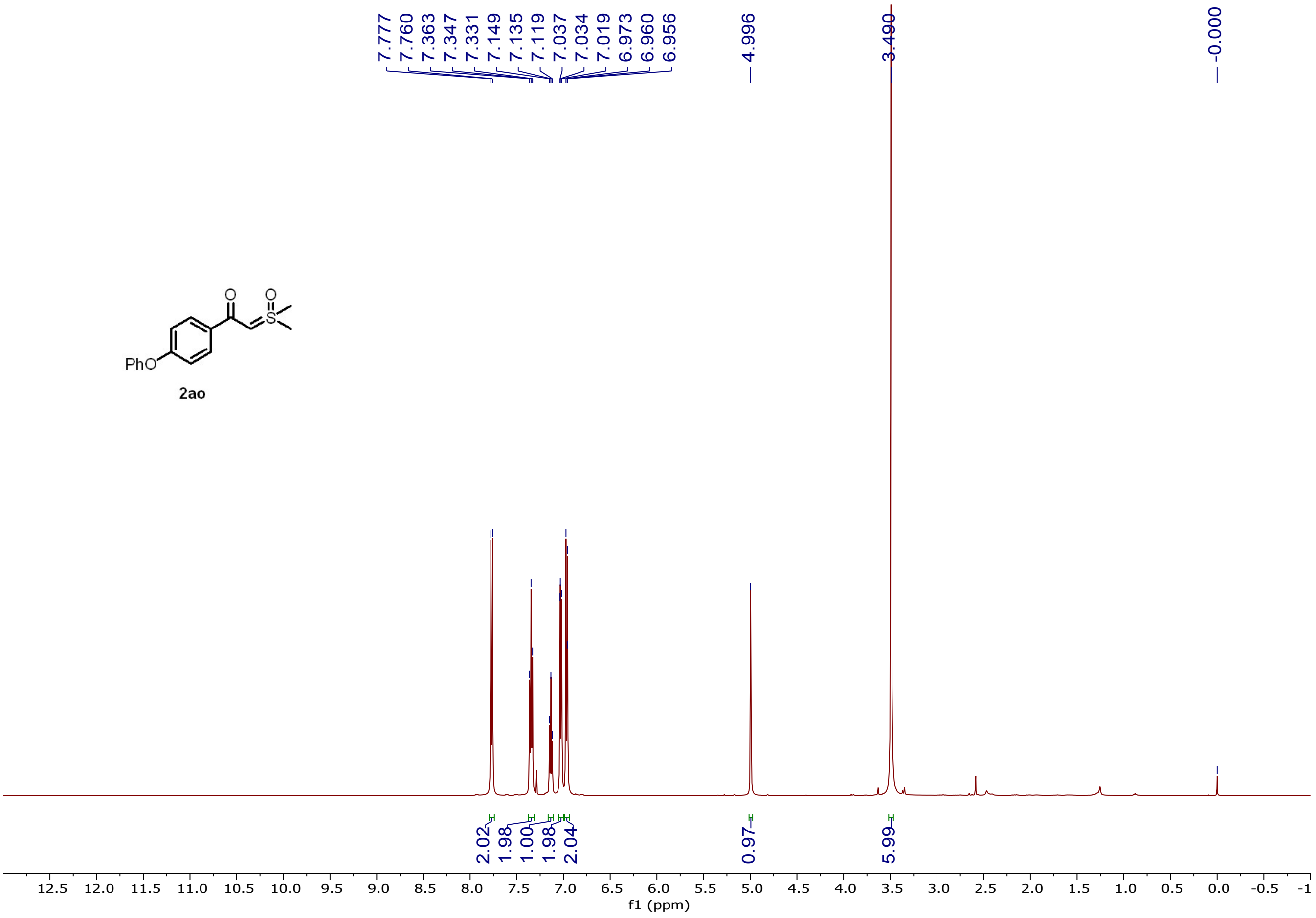
7.777
7.760
7.363
7.347
7.331
7.149
7.135
7.119
7.037
7.034
7.019
6.973
6.960
6.956
4.996
3.490
-0.000
PhO
2ao
2.02
1.98
1.00
1.98
2.04
0.97
5.99
f1 (ppm)

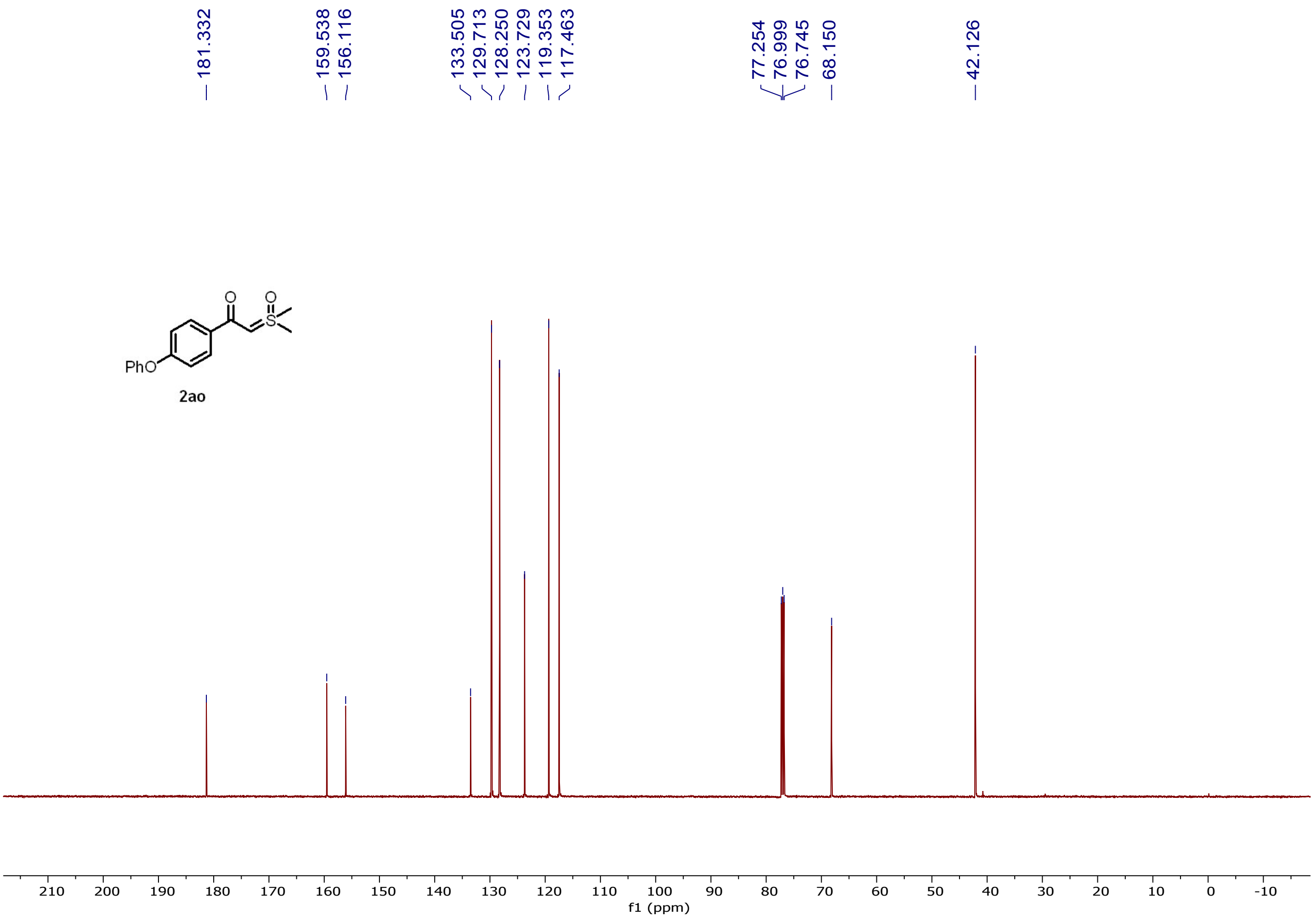
181.332
159.538
156.116
133.505
129.713
128.250
123.729
119.353
117.463
77.254
76.999
76.745
68.150
42.126
PhO
2ao
f1 (ppm)

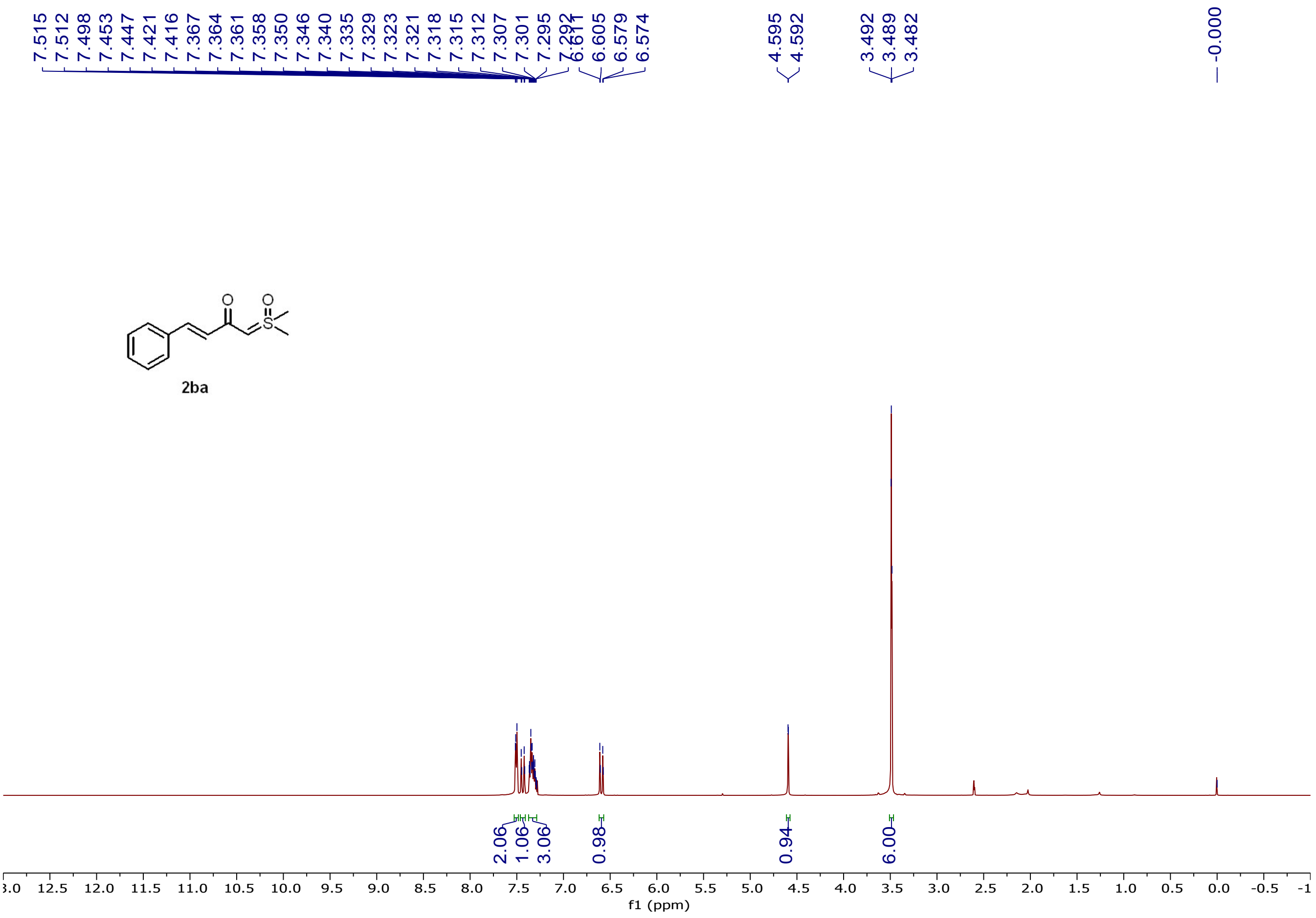
7.515 7.512 7.498 7.453 7.447 7.421 7.416 7.367 7.364 7.361 7.358 7.350 7.346 7.340 7.335 7.329 7.323 7.321 7.318 7.315 7.312 7.307 7.301 7.295 7.292 6.611 6.605 6.579 6.574
4.595 4.592
3.492 3.489 3.482
-0.000
2ba
2.06 1.06 3.06
0.98
0.94
6.00
f1 (ppm)

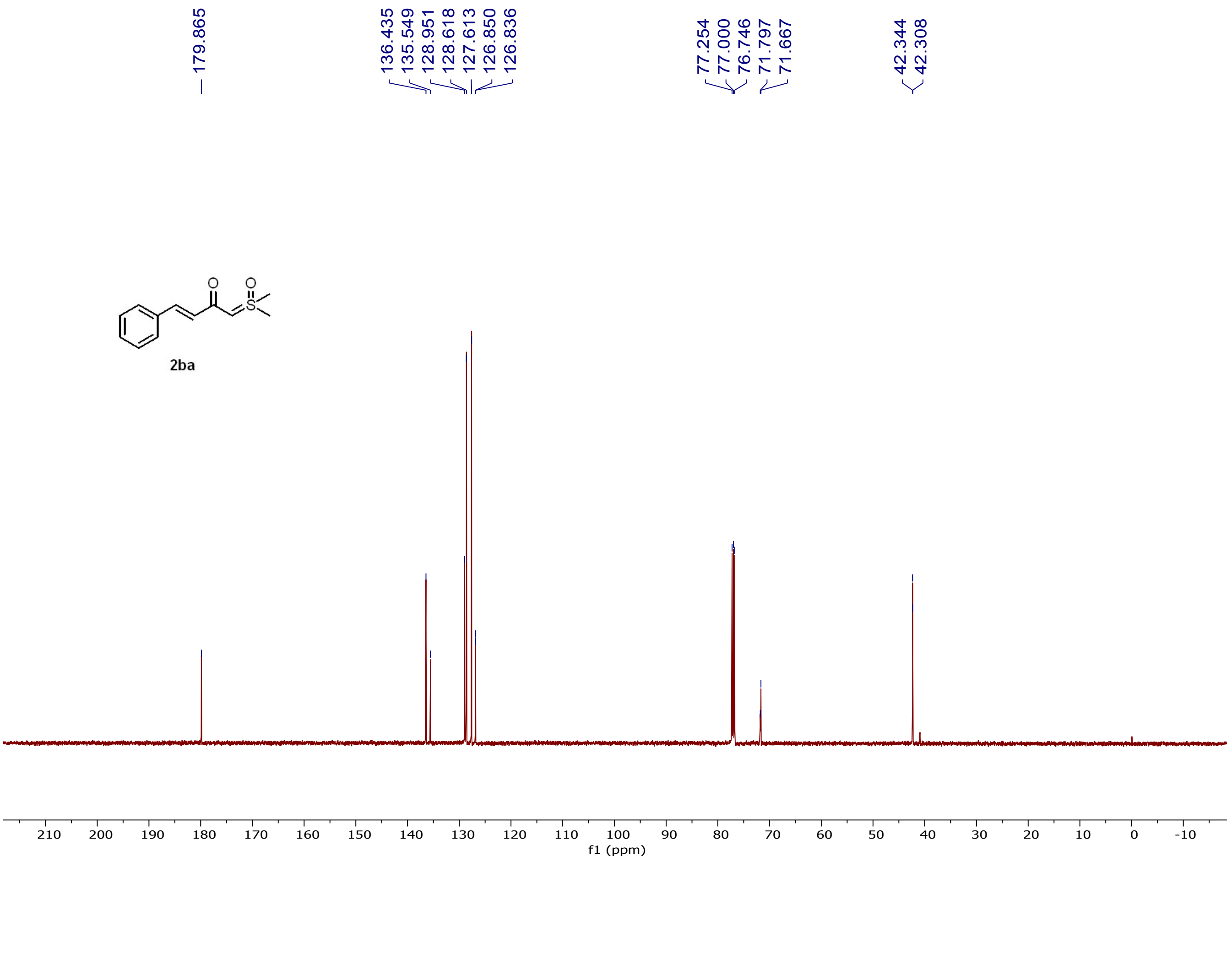
179.865
136.435 135.549 128.951 128.618 127.613 126.850 126.836
77.254 77.000 76.746 71.797 71.667
42.344 42.308
2ba
f1 (ppm)

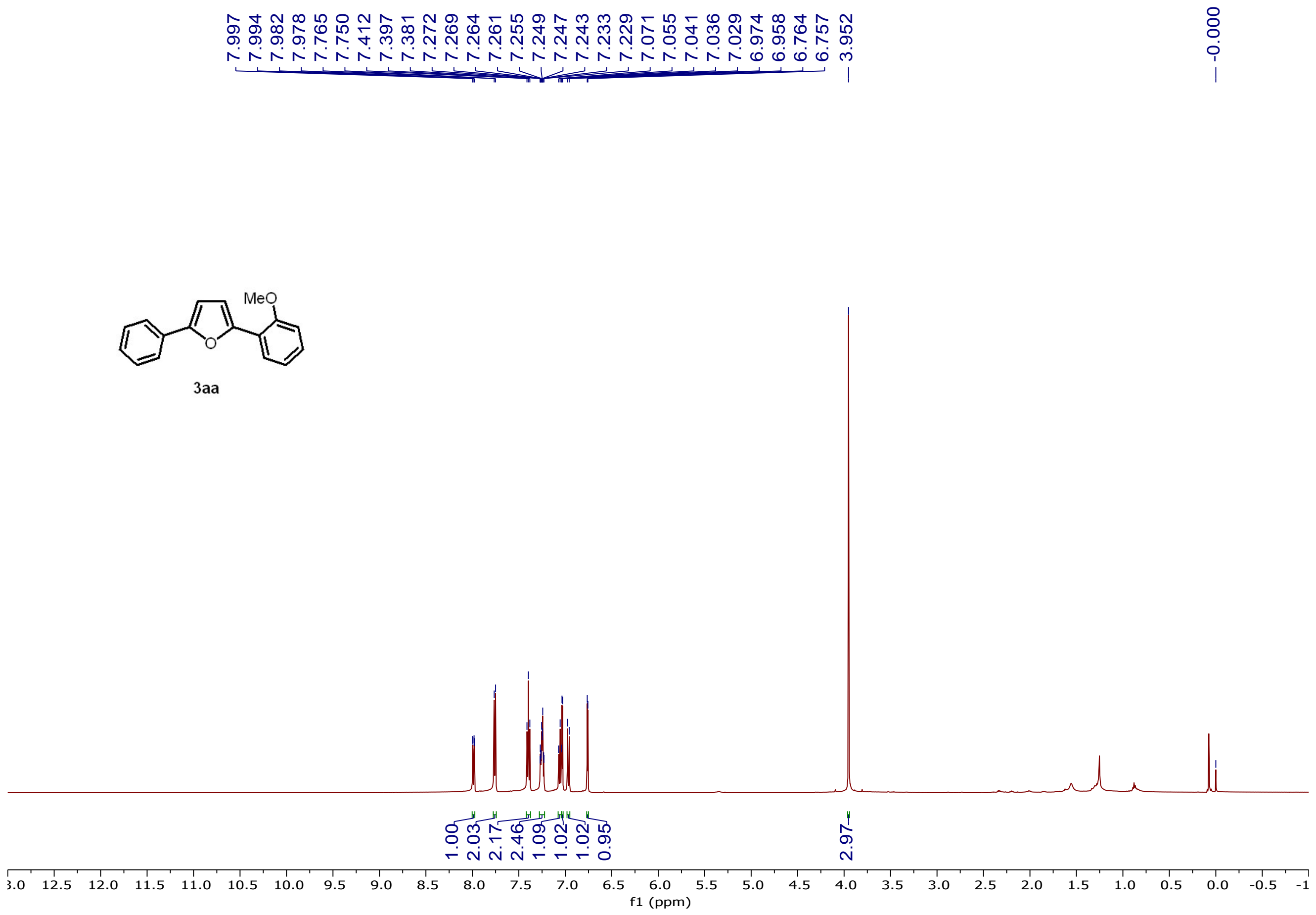
7.997 7.994 7.982 7.978 7.765 7.750 7.412 7.397 7.381 7.272 7.269 7.264 7.261 7.255 7.249 7.247 7.243 7.233 7.229 7.071 7.055 7.041 7.036 7.029 6.974 6.958 6.764 6.757 3.952
-0.000
MeO
3aa
1.00 2.03 2.17 2.46 1.09 1.02 1.02 0.95
2.97
f1 (ppm)

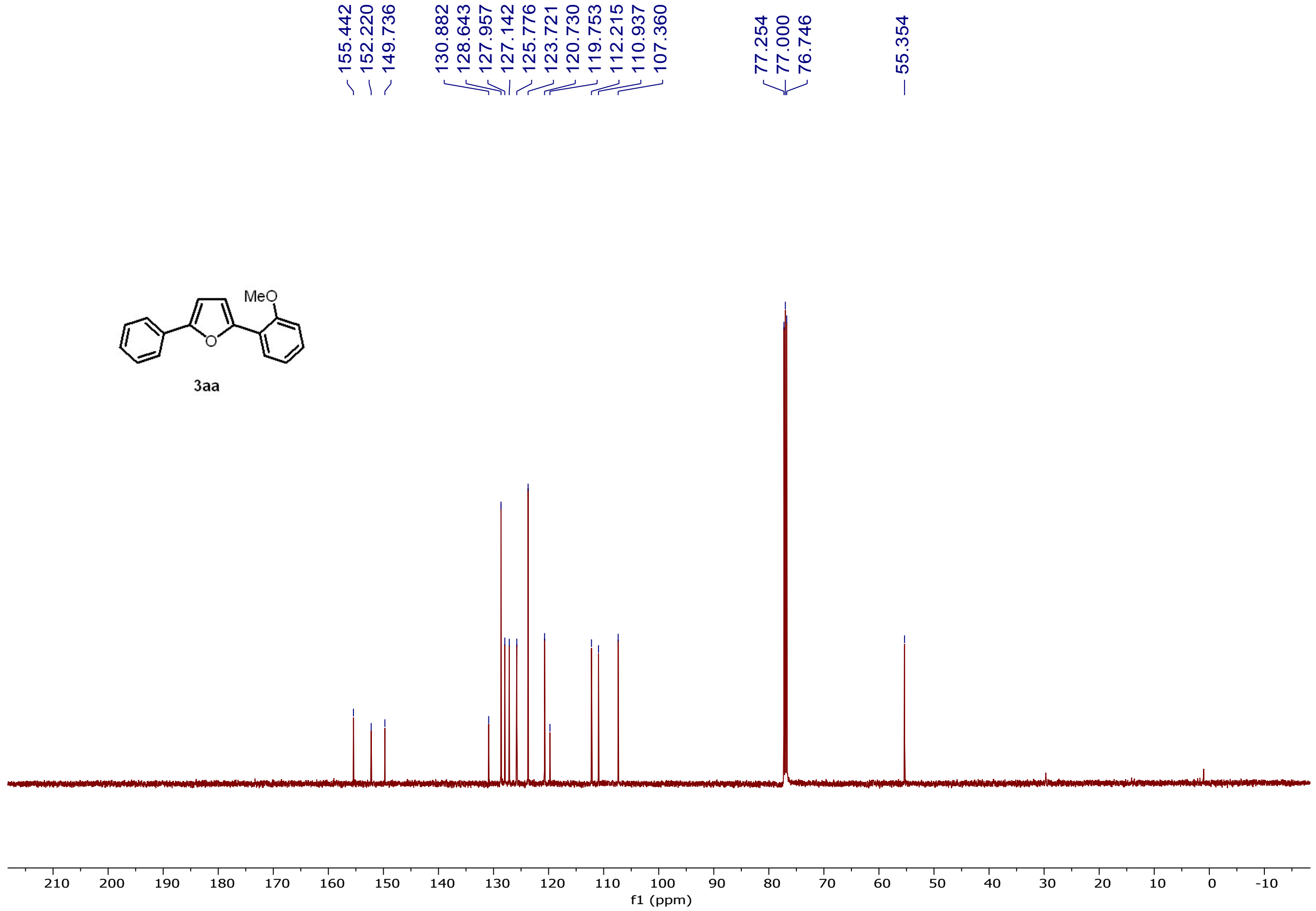
155.442 152.220 149.736
130.882 128.643 127.957 127.142 125.776 123.721 120.730 119.753 112.215 110.937 107.360
77.254 77.000 76.746
55.354
MeO
3aa
f1 (ppm)

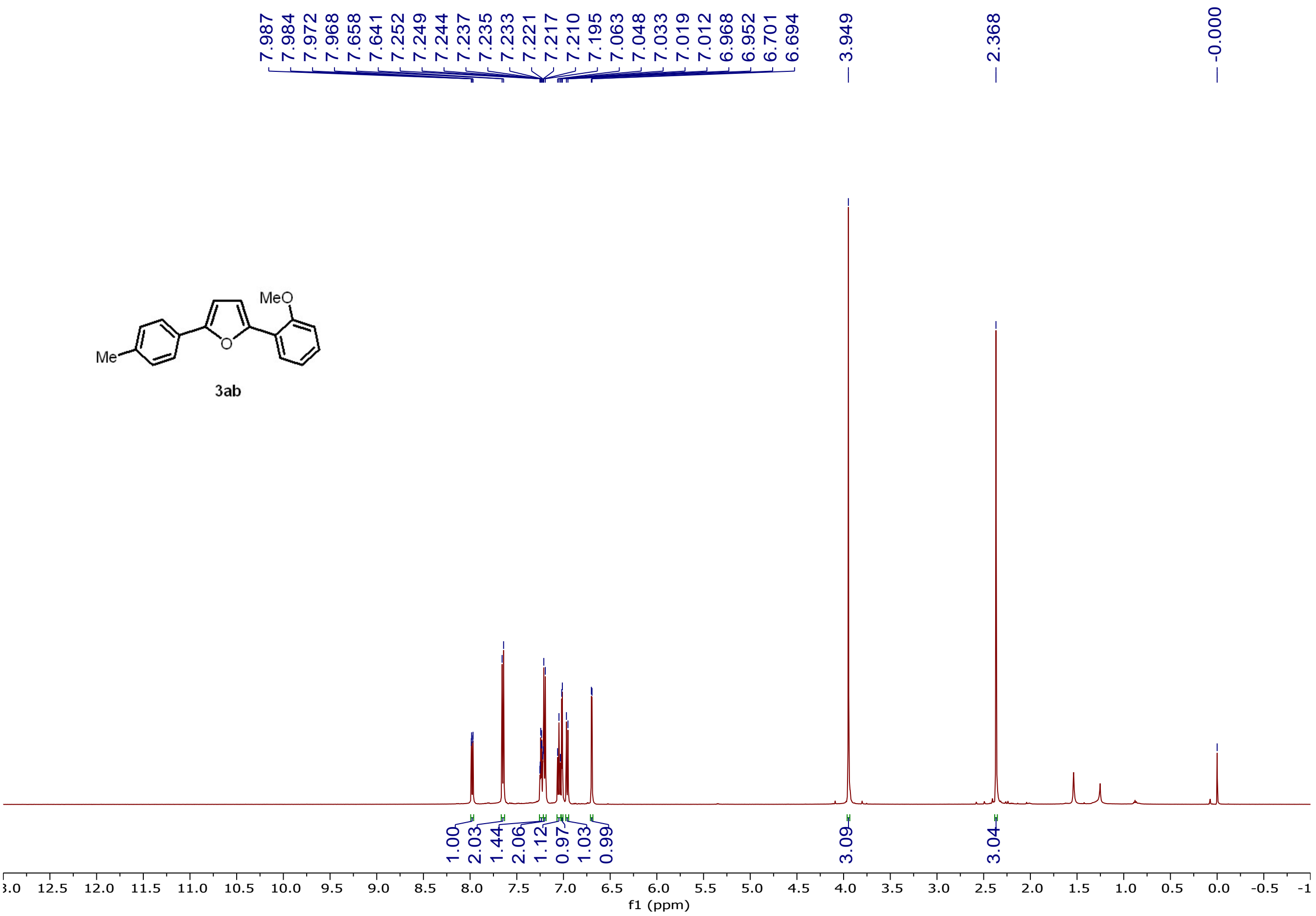

7.987
7.984
7.972
7.968
7.658
7.641
7.252
7.249
7.244
7.237
7.235
7.233
7.221
7.217
7.210
7.195
7.063
7.048
7.033
7.019
7.012
6.968
6.952
6.701
6.694
3.949
2.368
-0.000
MeO
Me
O
3ab
1.00
2.03
1.44
2.06
1.12
0.97
1.03
0.99
3.09
3.04
f1 (ppm)

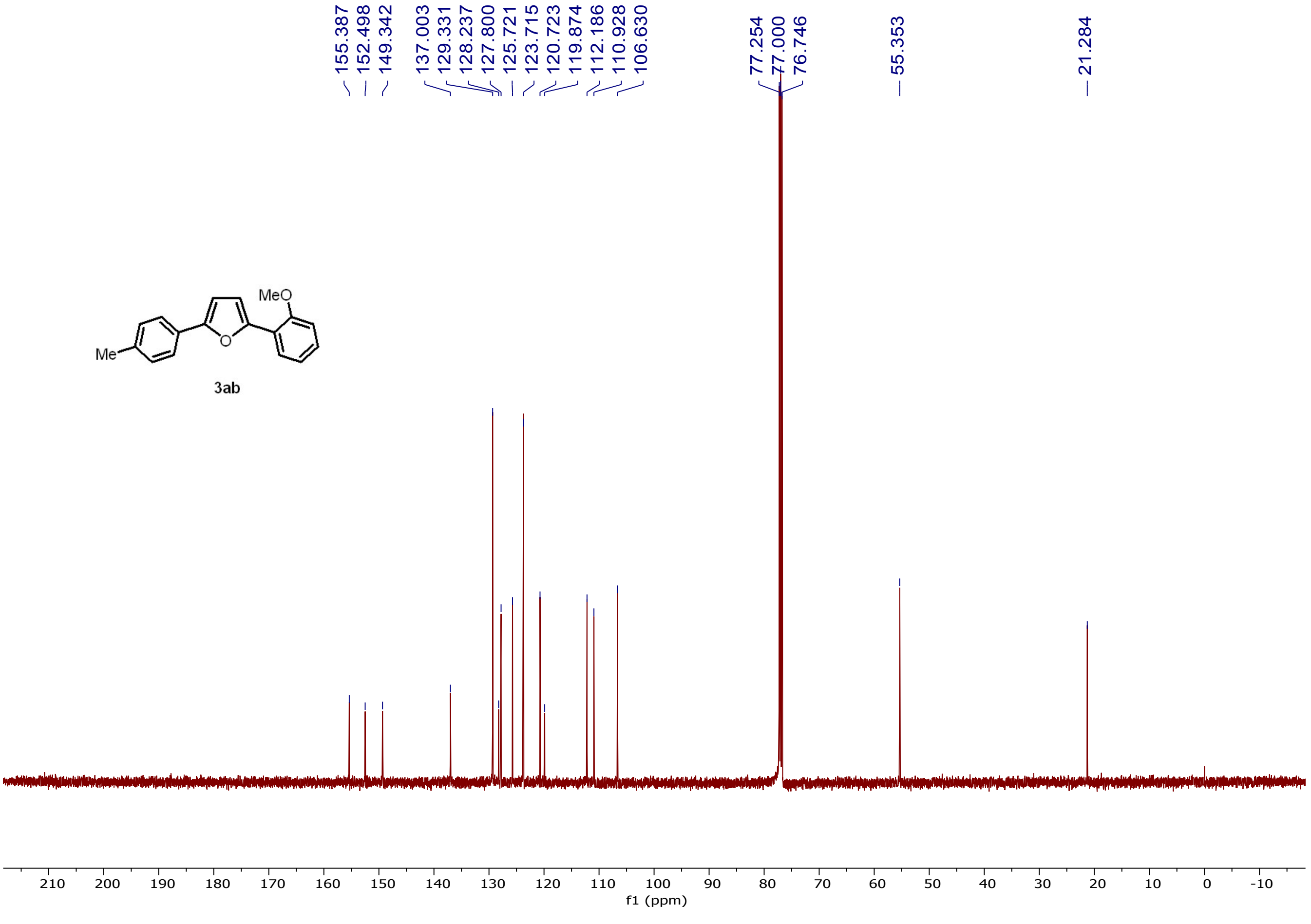

155.387
152.498
149.342
137.003
129.331
128.237
127.800
125.721
123.715
120.723
119.874
112.186
110.928
106.630
77.254
77.000
76.746
55.353
21.284
MeO
Me
O
3ab
f1 (ppm)

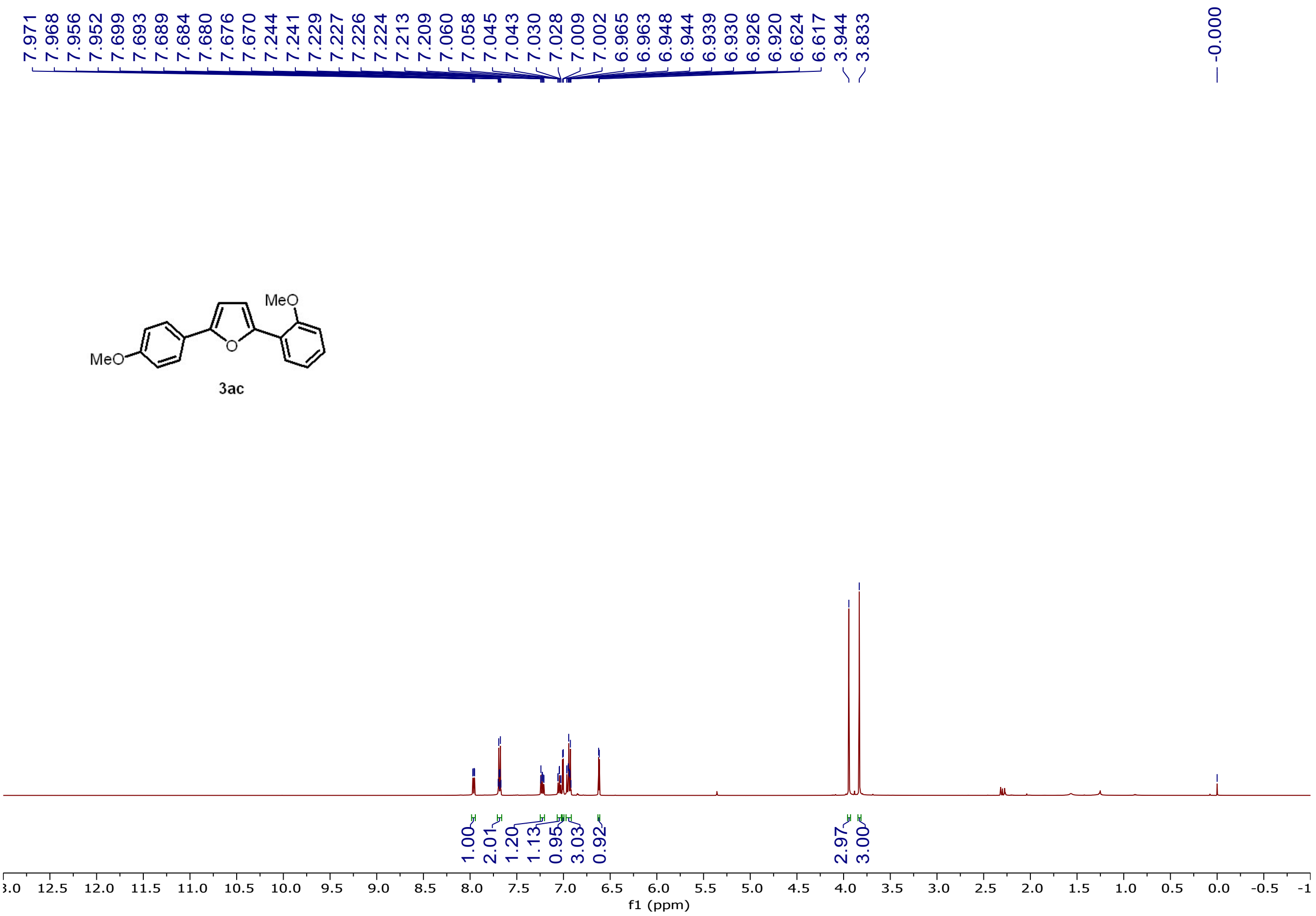
7.971 7.968 7.956 7.952 7.699 7.693 7.689 7.684 7.680 7.676 7.670 7.244 7.241 7.229 7.227 7.226 7.224 7.213 7.209 7.060 7.058 7.045 7.043 7.030 7.028 7.009 7.002 6.965 6.963 6.948 6.944 6.939 6.930 6.926 6.920 6.624 6.617 3.944 3.833
-0.000
MeO
MeO
O
3ac
1.00 2.01 1.20 1.13 0.95 3.03 0.92
2.97 3.00
f1 (ppm)

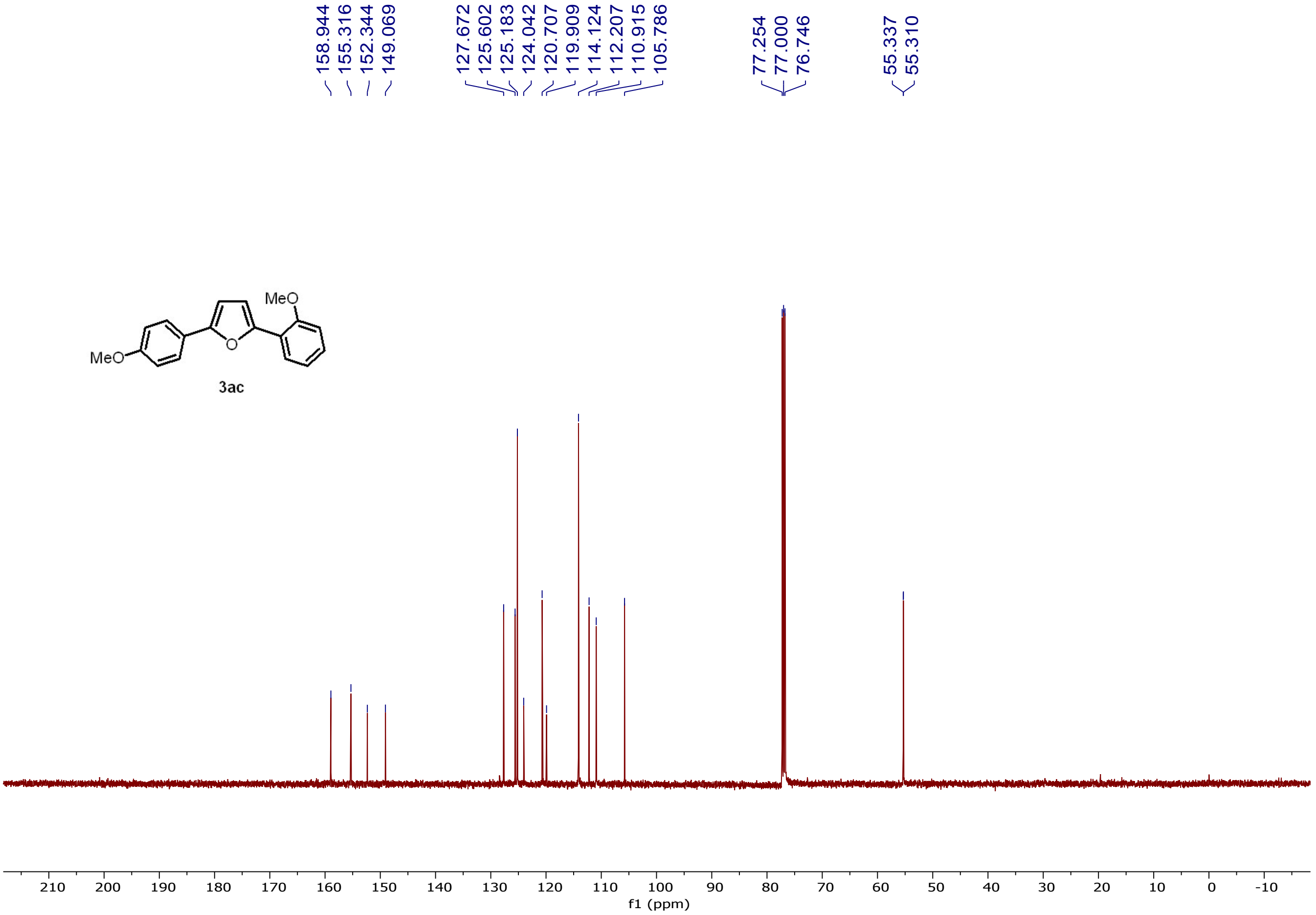
158.944 155.316 152.344 149.069
127.672 125.602 125.183 124.042 120.707 119.909 114.124 112.207 110.915 105.786
77.254 77.000 76.746
55.337 55.310
MeO
MeO
O
3ac
f1 (ppm)

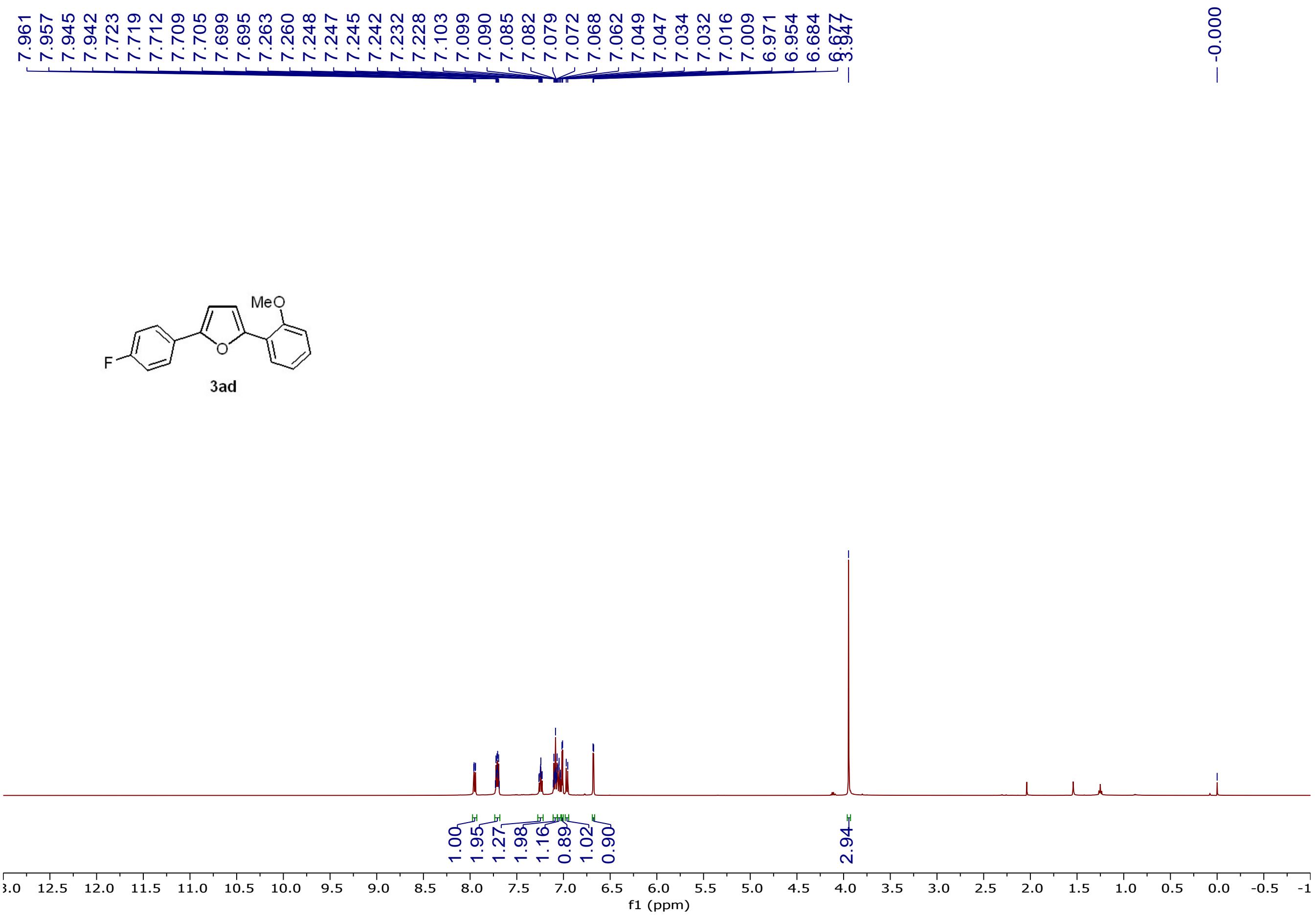
7.961 7.957 7.945 7.942 7.723 7.719 7.712 7.709 7.705 7.699 7.695 7.263 7.260 7.248 7.247 7.245 7.242 7.232 7.228 7.103 7.099 7.090 7.085 7.082 7.079 7.072 7.068 7.062 7.049 7.047 7.034 7.032 7.016 7.009 6.971 6.954 6.684 6.677 3.947
-0.000
MeO
F
O
3ad
1.00 1.95 1.27 1.98 1.16 0.89 1.02 0.90
2.94
f1 (ppm)

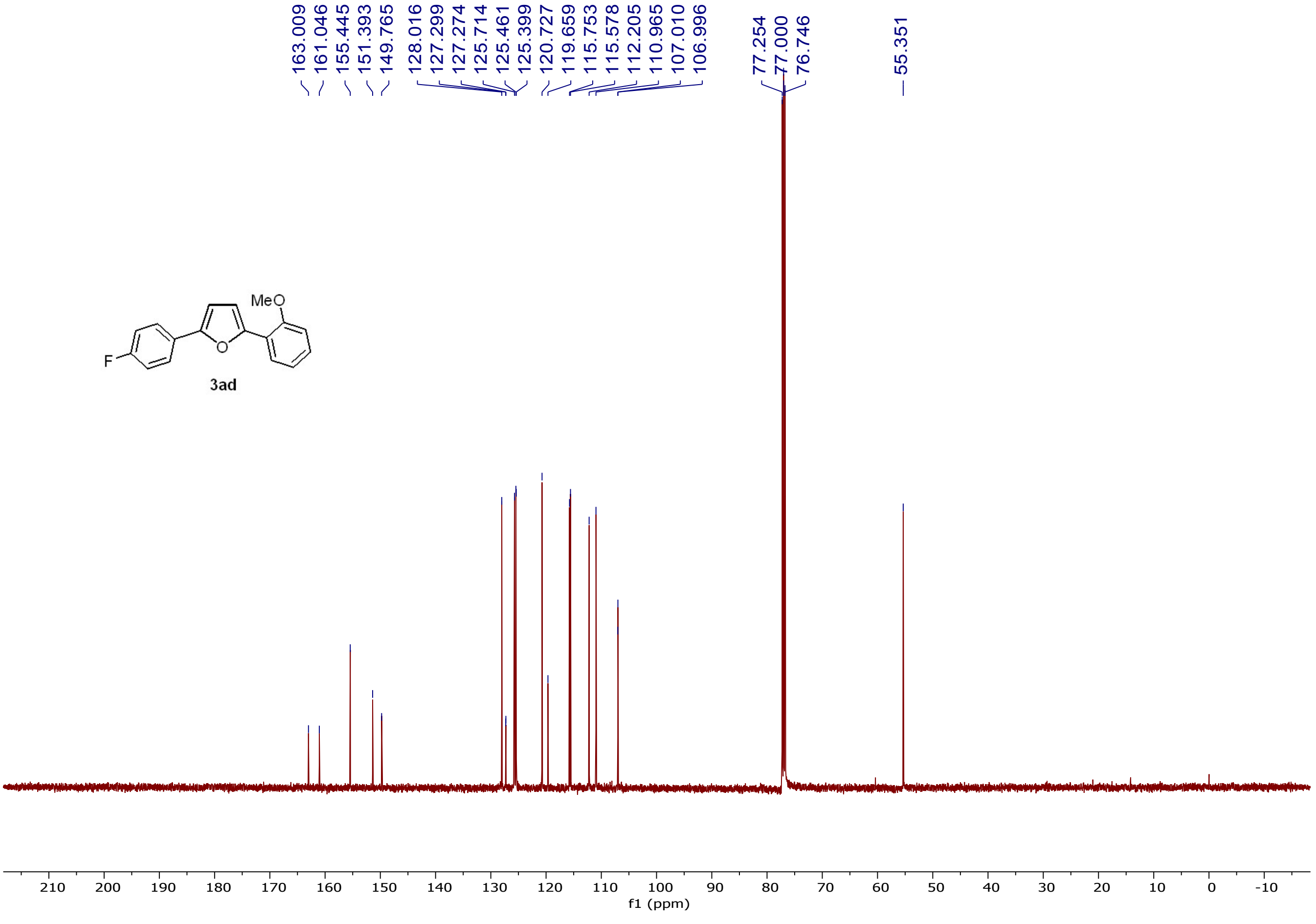
163.009 161.046 155.445 151.393 149.765 128.016 127.299 127.274 125.714 125.461 125.399 120.727 119.659 115.753 115.578 112.205 110.965 107.010 106.996
77.254 77.000 76.746
55.351
MeO
F
O
3ad
f1 (ppm)

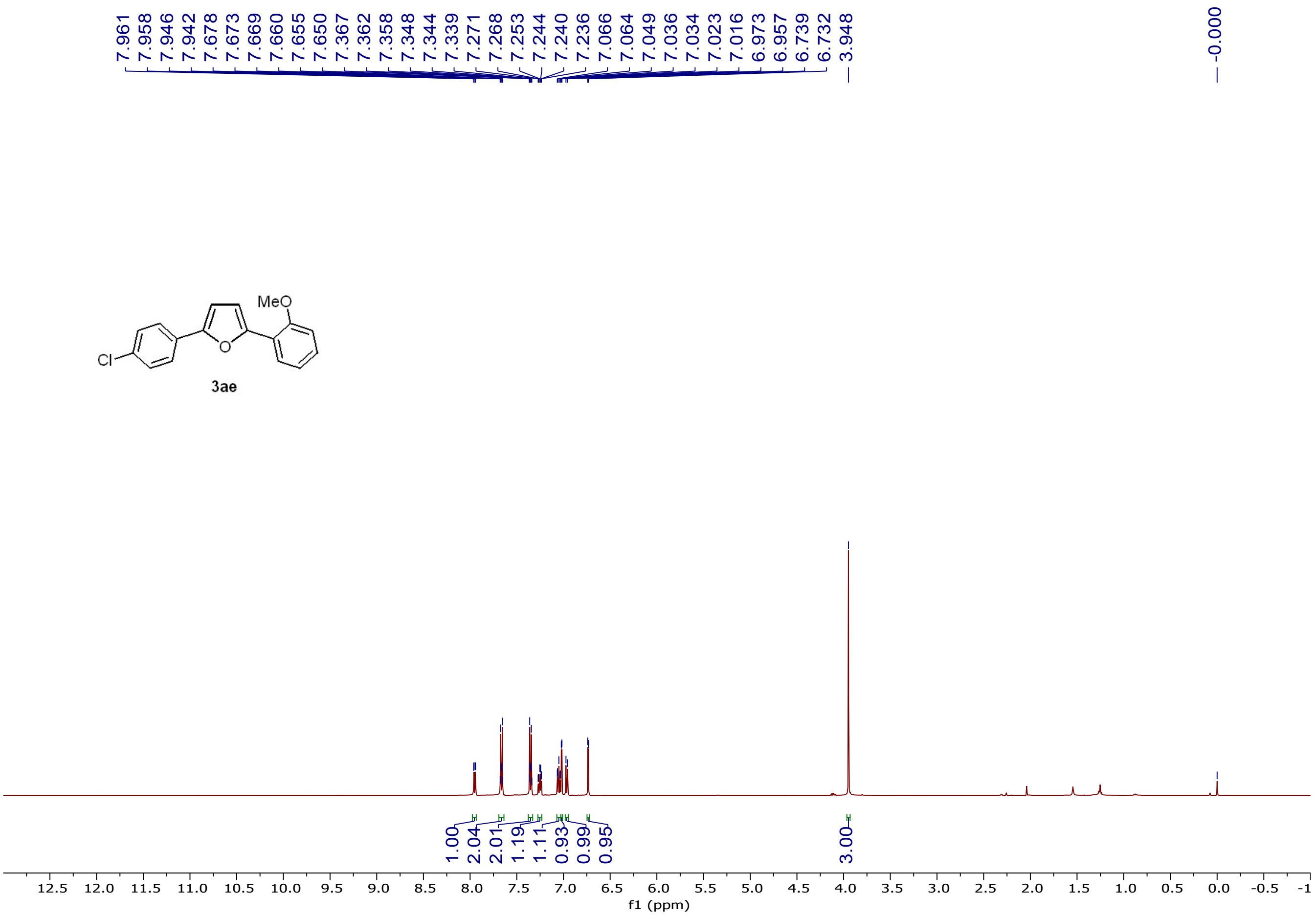
7.961
7.958
7.946
7.942
7.678
7.673
7.669
7.660
7.655
7.650
7.367
7.362
7.358
7.348
7.344
7.339
7.271
7.268
7.253
7.244
7.240
7.236
7.066
7.064
7.049
7.036
7.034
7.023
7.016
6.973
6.957
6.739
6.732
3.948
-0.000
MeO
Cl
O
3ae
1.00
2.04
2.01
1.19
1.11
0.93
0.99
0.95
3.00
f1 (ppm)

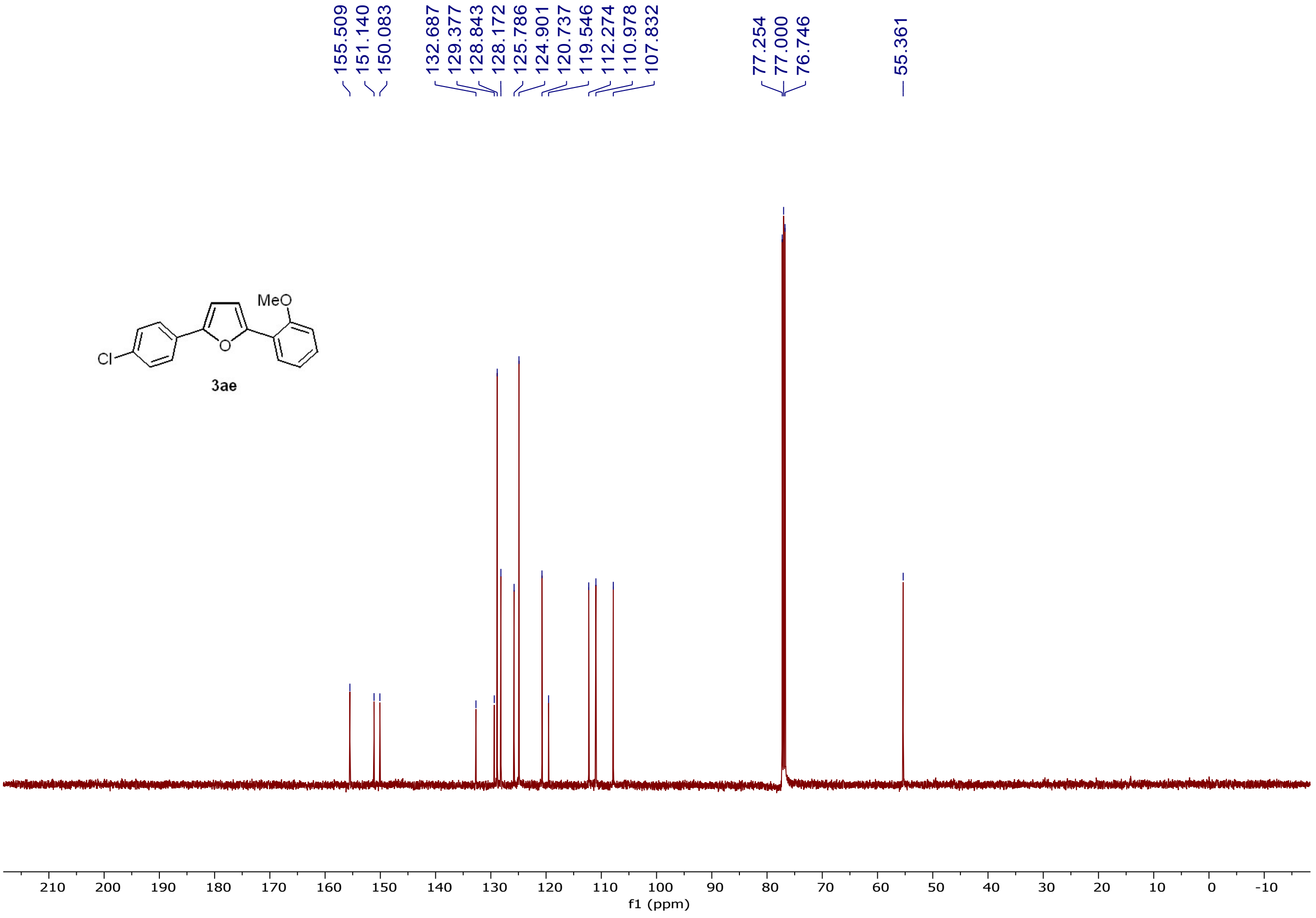
155.509
151.140
150.083
132.687
129.377
128.843
128.172
125.786
124.901
120.737
119.546
112.274
110.978
107.832
77.254
77.000
76.746
55.361
MeO
Cl
O
3ae
f1 (ppm)

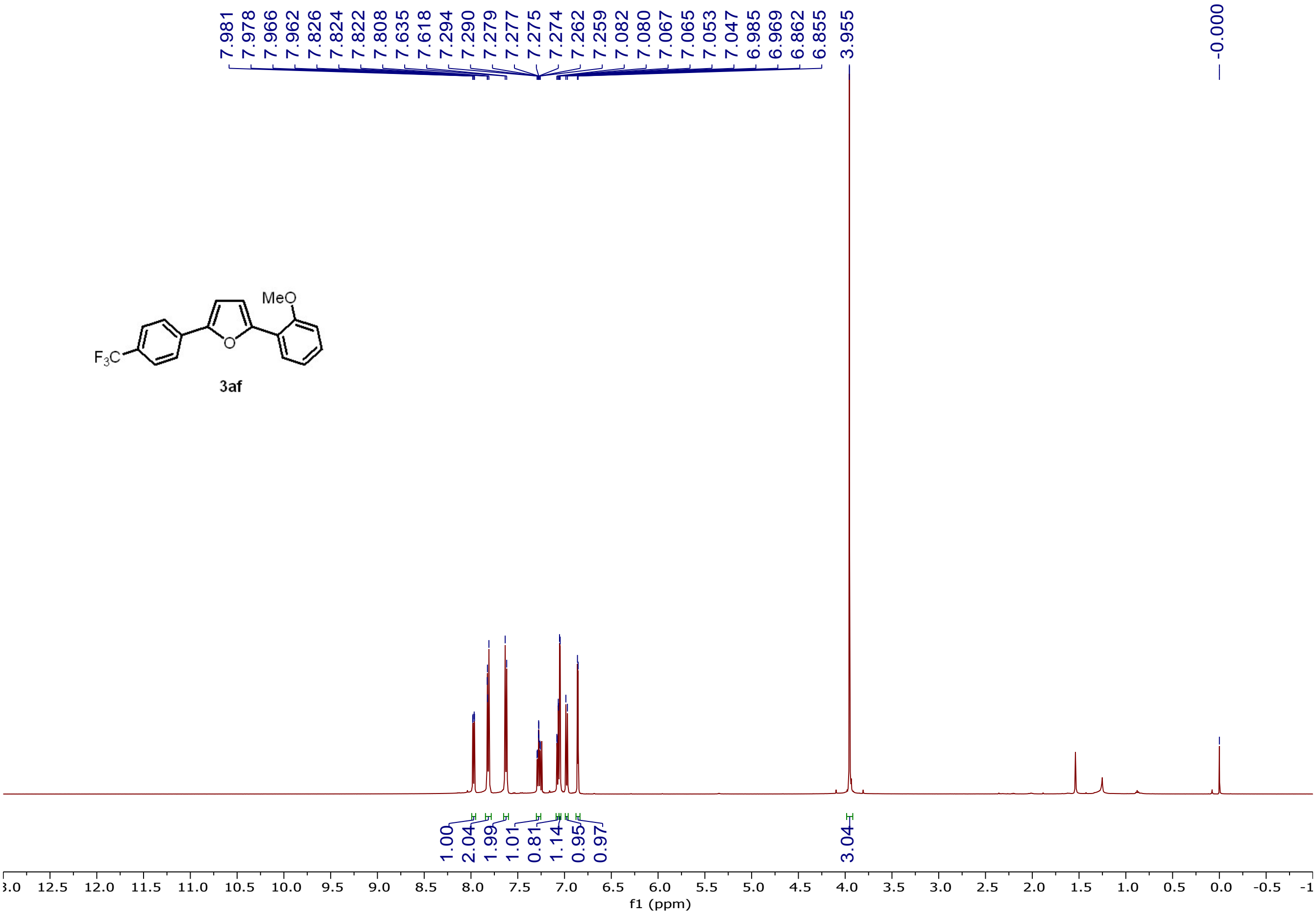

7.981
7.978
7.966
7.962
7.826
7.824
7.822
7.808
7.635
7.618
7.294
7.290
7.279
7.277
7.275
7.274
7.262
7.259
7.082
7.080
7.067
7.065
7.053
7.047
6.985
6.969
6.862
6.855
3.955
-0.000
MeO
F_3C
O
3af
1.00
2.04
1.99
1.01
0.81
1.14
0.95
0.97
3.04
f1 (ppm)

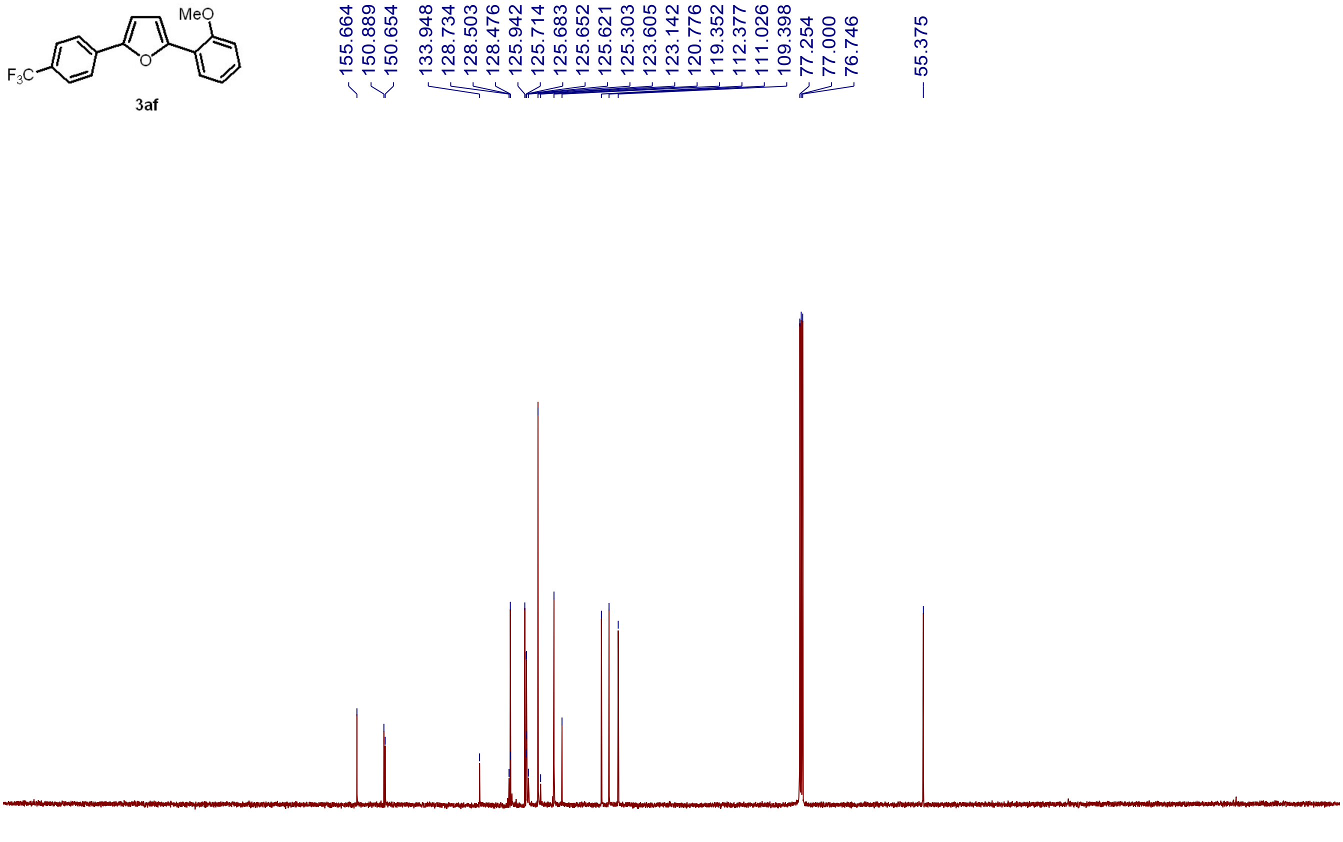

MeO
F_3C
O
3af
155.664
150.889
150.654
133.948
128.734
128.503
128.476
125.942
125.714
125.683
125.652
125.621
125.303
123.605
123.142
120.776
119.352
112.377
111.026
109.398
77.254
77.000
76.746
55.375
f1 (ppm)

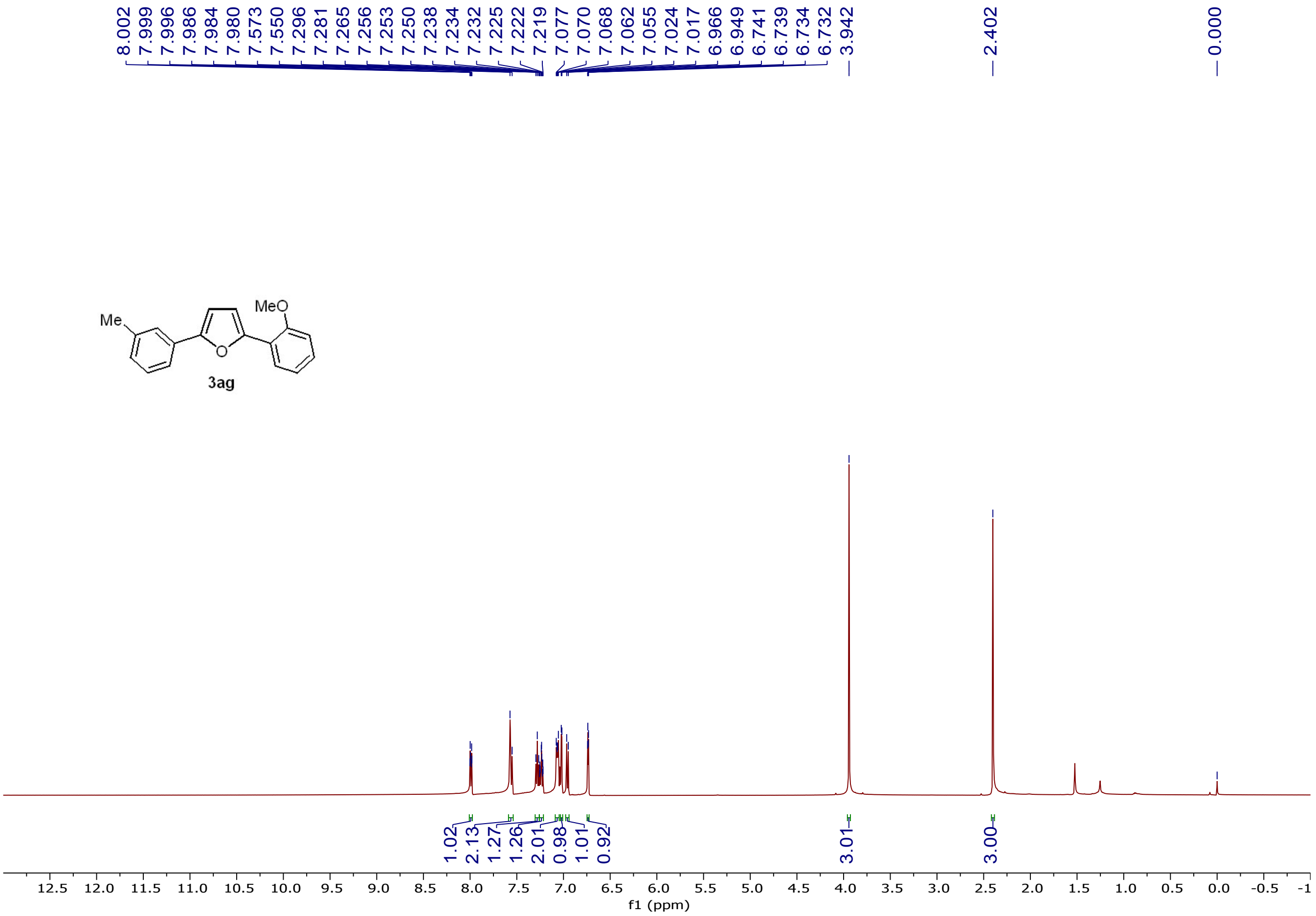
8.002
7.999
7.996
7.986
7.984
7.980
7.573
7.550
7.296
7.281
7.265
7.256
7.253
7.250
7.238
7.234
7.232
7.225
7.222
7.219
7.077
7.070
7.068
7.062
7.055
7.024
7.017
6.966
6.949
6.741
6.739
6.734
6.732
3.942
2.402
0.000
Me
MeO
O
3ag
1.02
2.13
1.27
1.26
2.01
0.98
1.01
0.92
3.01
3.00
f1 (ppm)

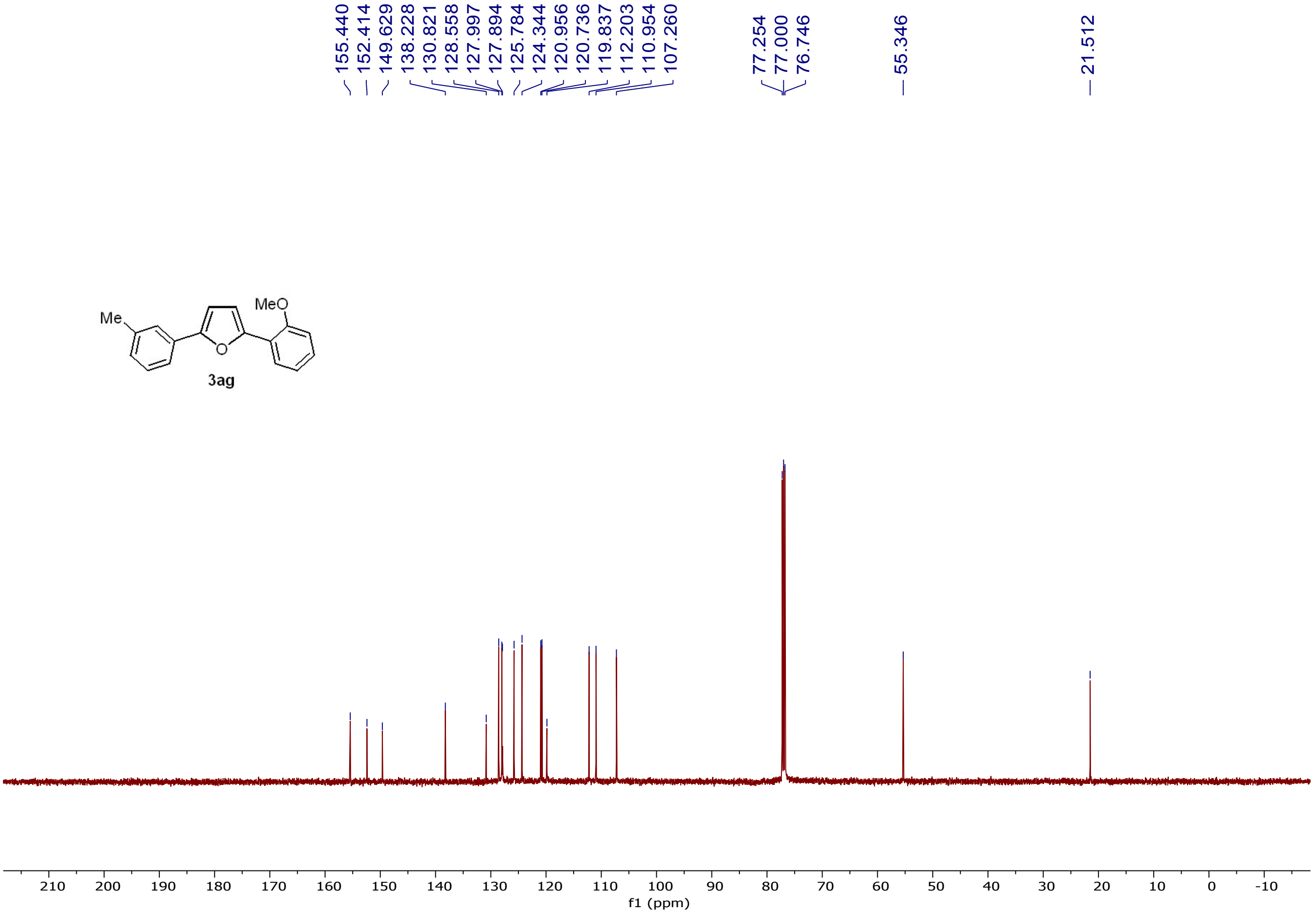
155.440
152.414
149.629
138.228
130.821
128.558
127.997
127.894
125.784
124.344
120.956
120.736
119.837
112.203
110.954
107.260
77.254
77.000
76.746
55.346
21.512
Me
MeO
O
3ag
f1 (ppm)

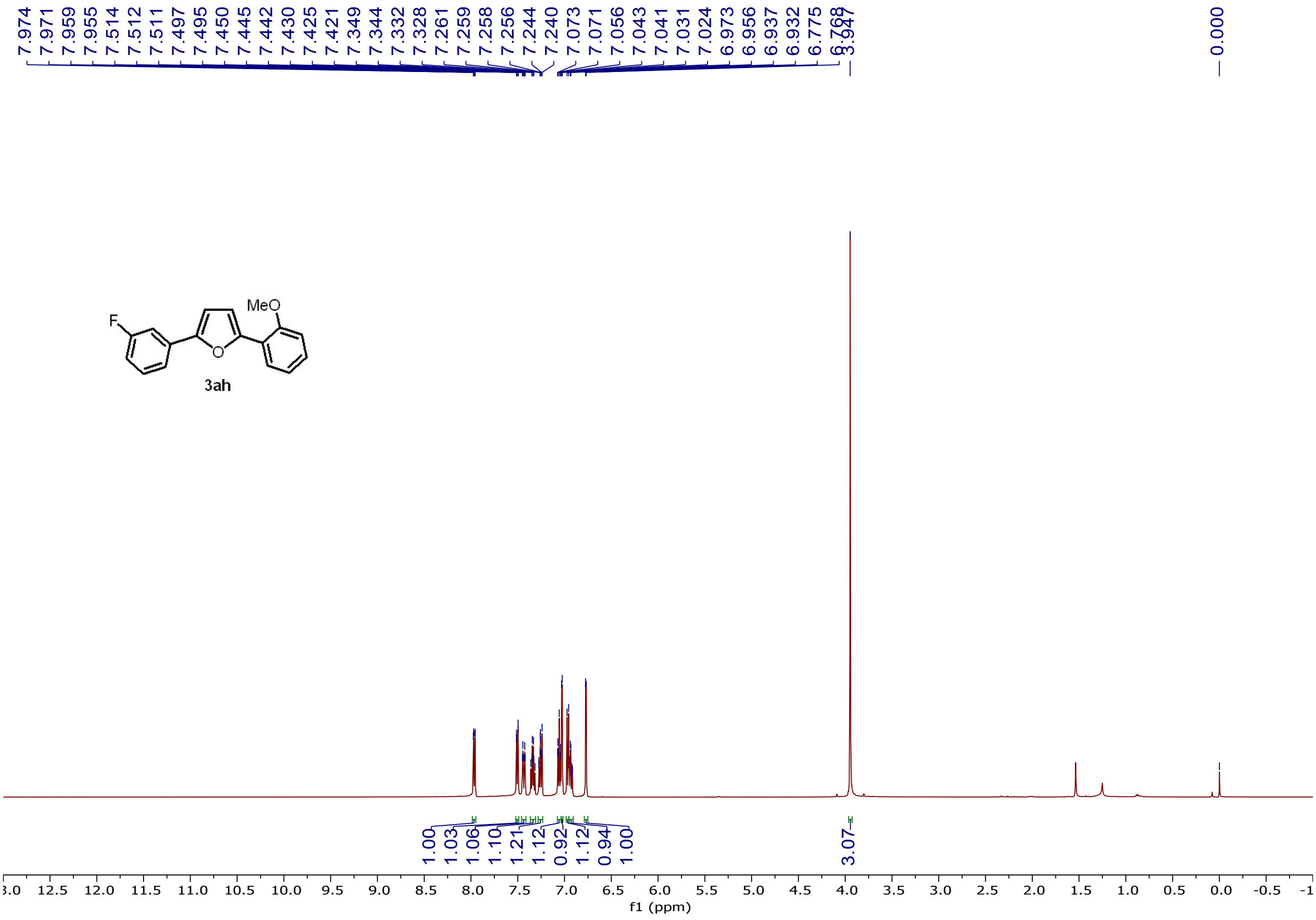
7.974
7.971
7.959
7.955
7.514
7.512
7.511
7.497
7.495
7.450
7.445
7.442
7.430
7.425
7.421
7.349
7.344
7.332
7.328
7.261
7.259
7.258
7.256
7.244
7.240
7.073
7.071
7.056
7.043
7.041
7.031
7.024
6.973
6.956
6.937
6.932
6.775
6.768
3.947
0.000
MeO
F
O
3ah
1.00
1.03
1.06
1.10
1.21
1.12
0.92
1.12
0.94
1.00
3.07
f1 (ppm)

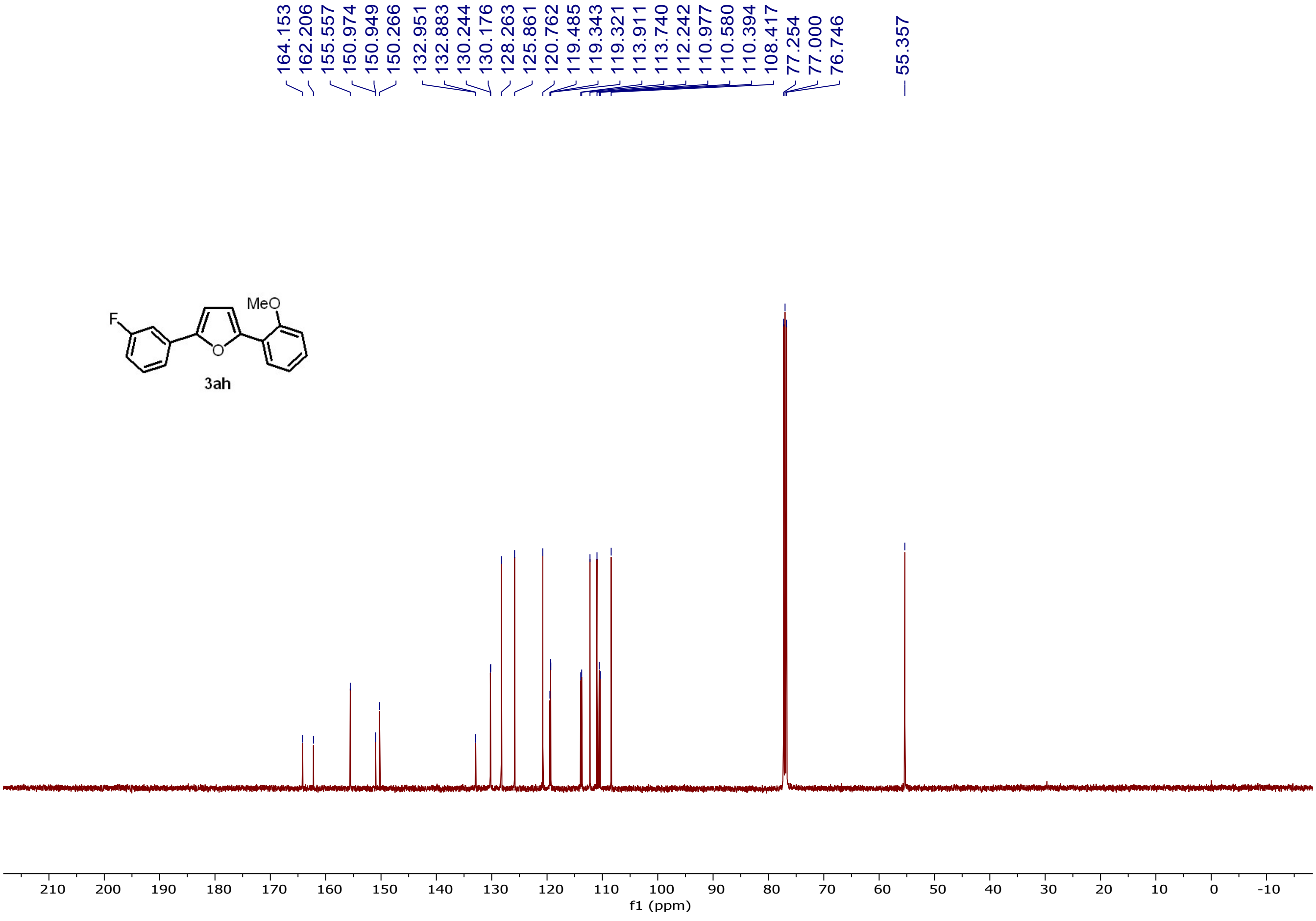
164.153
162.206
155.557
150.974
150.949
150.266
132.951
132.883
130.244
130.176
128.263
125.861
120.762
119.485
119.343
119.321
113.911
113.740
112.242
110.977
110.580
110.394
108.417
77.254
77.000
76.746
55.357
MeO
F
O
3ah
f1 (ppm)

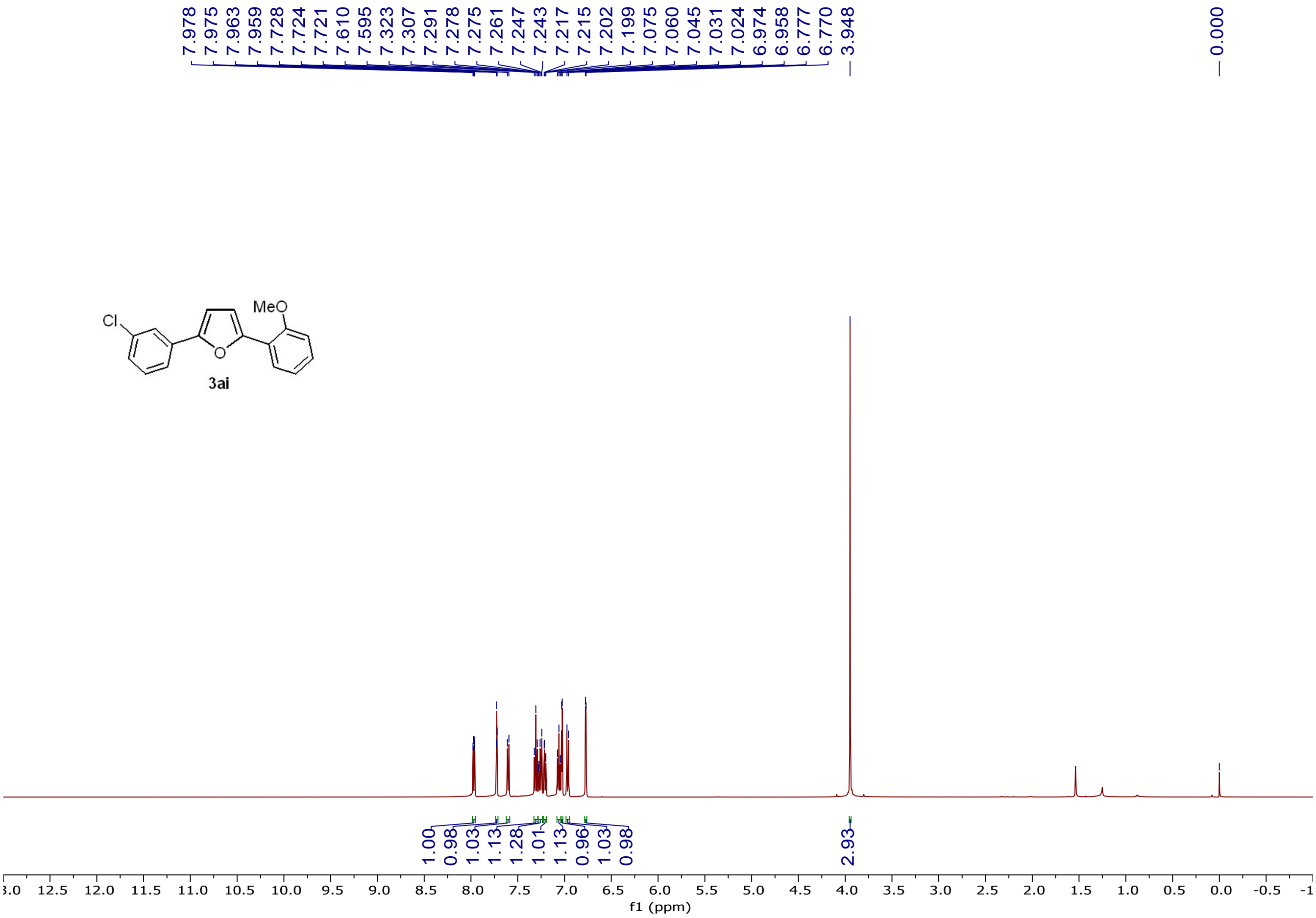
7.978
7.975
7.963
7.959
7.728
7.724
7.721
7.610
7.595
7.323
7.307
7.291
7.278
7.275
7.261
7.247
7.243
7.217
7.215
7.202
7.199
7.075
7.060
7.045
7.031
7.024
6.974
6.958
6.777
6.770
3.948
0.000
Cl
MeO
O
3ai
1.00
0.98
1.03
1.13
1.28
1.01
1.13
0.96
1.03
0.98
2.93
f1 (ppm)

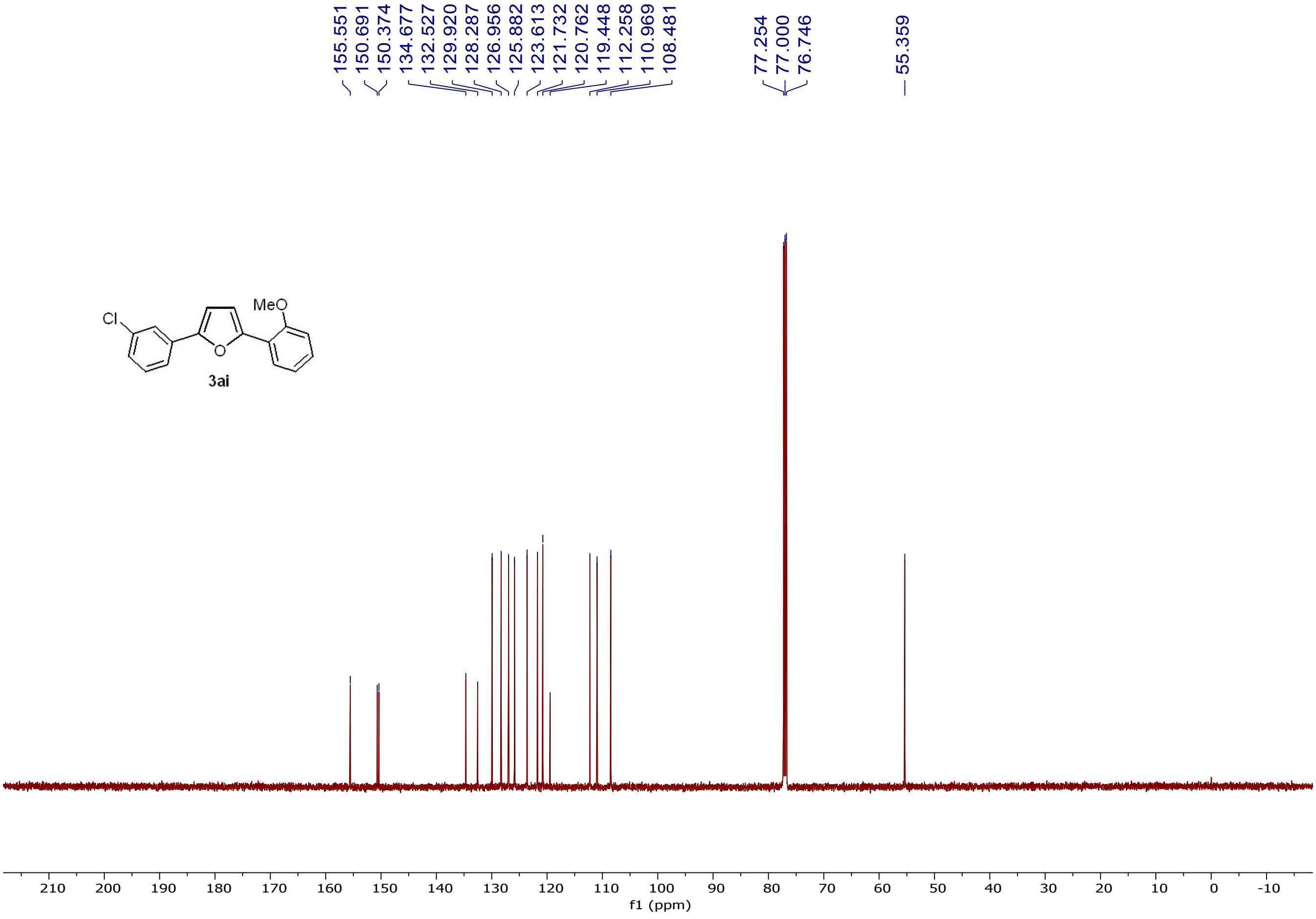
155.551
150.691
150.374
134.677
132.527
129.920
128.287
126.956
125.882
123.613
121.732
120.762
119.448
112.258
110.969
108.481
77.254
77.000
76.746
55.359
Cl
MeO
O
3ai
f1 (ppm)

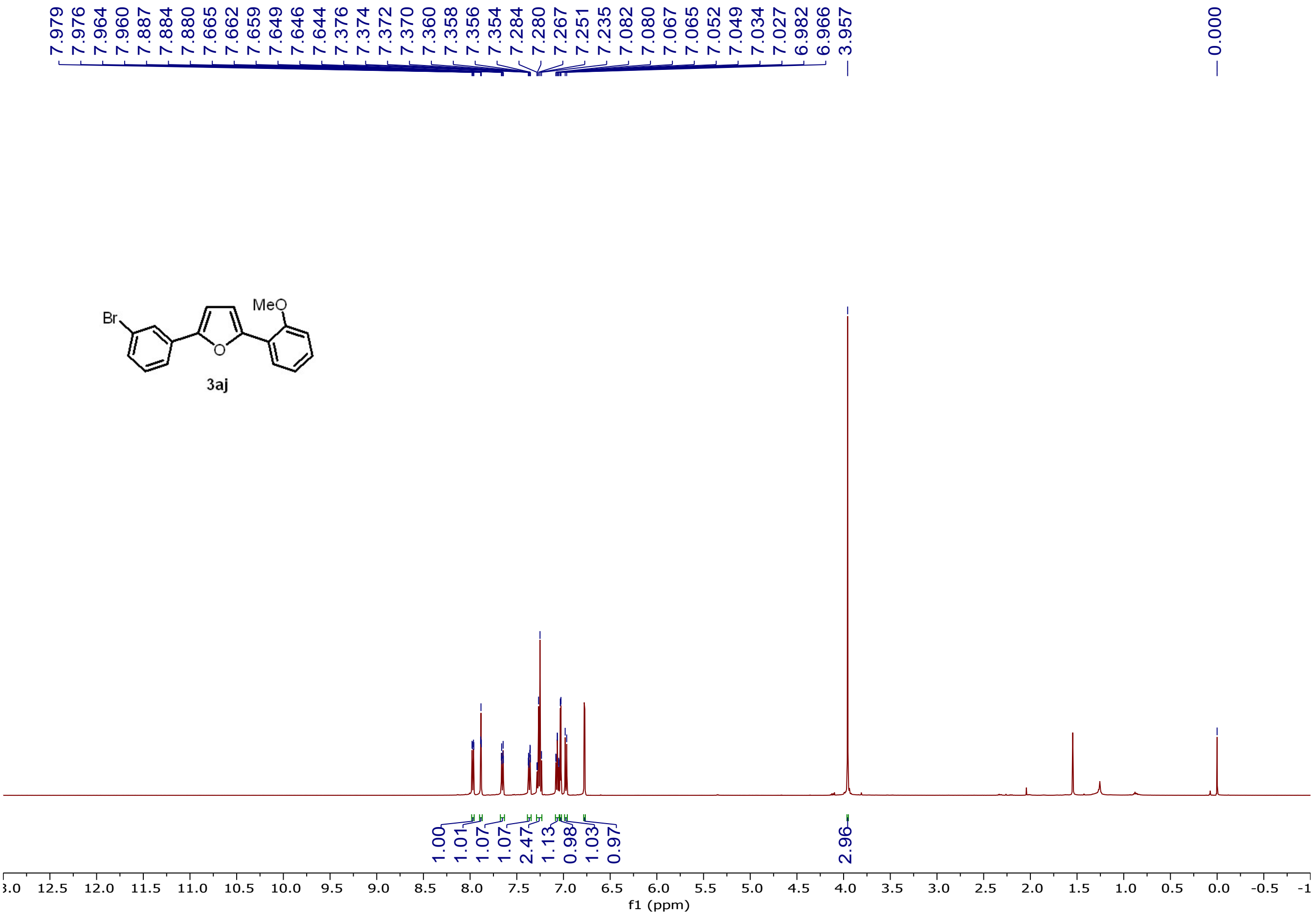

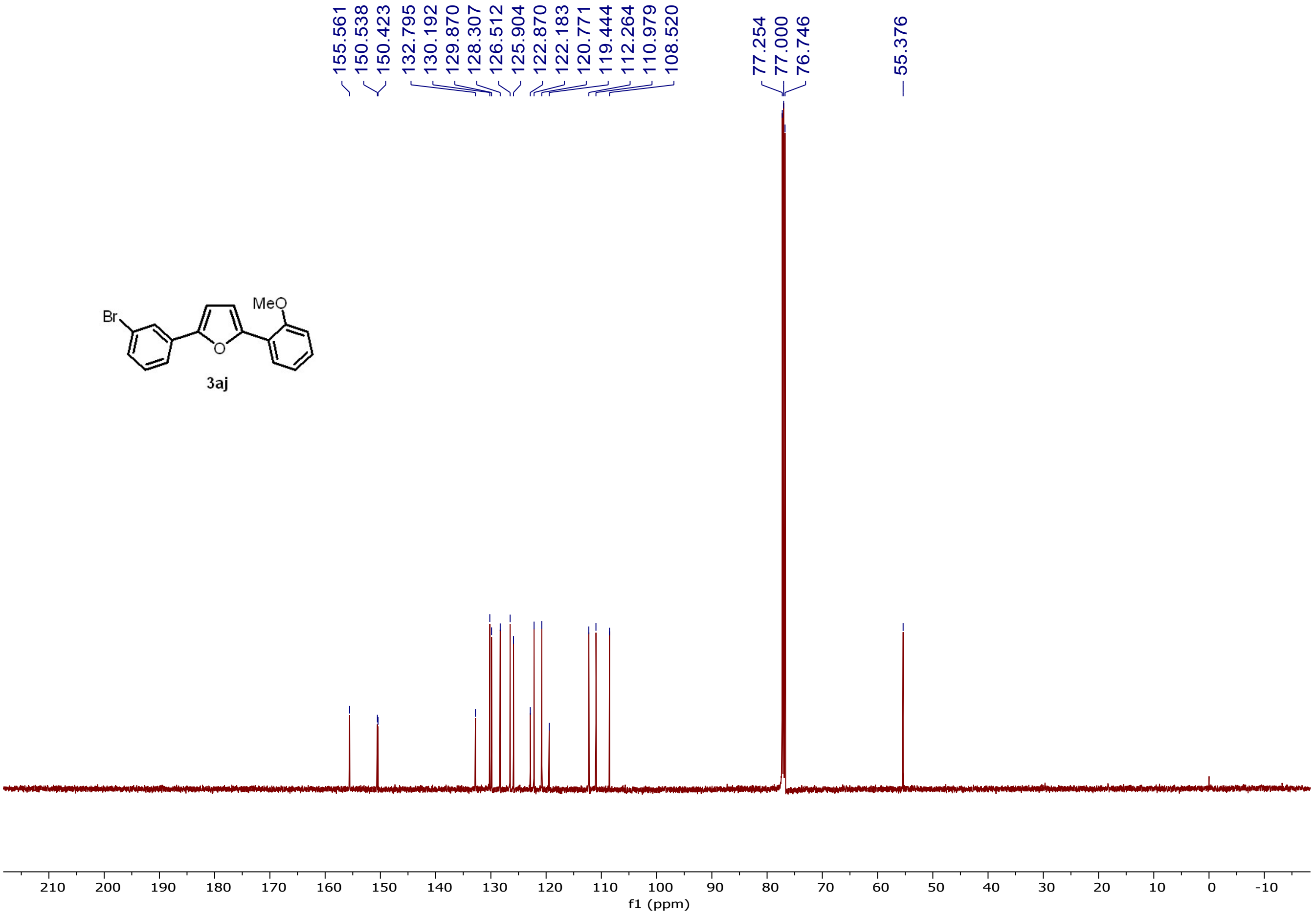

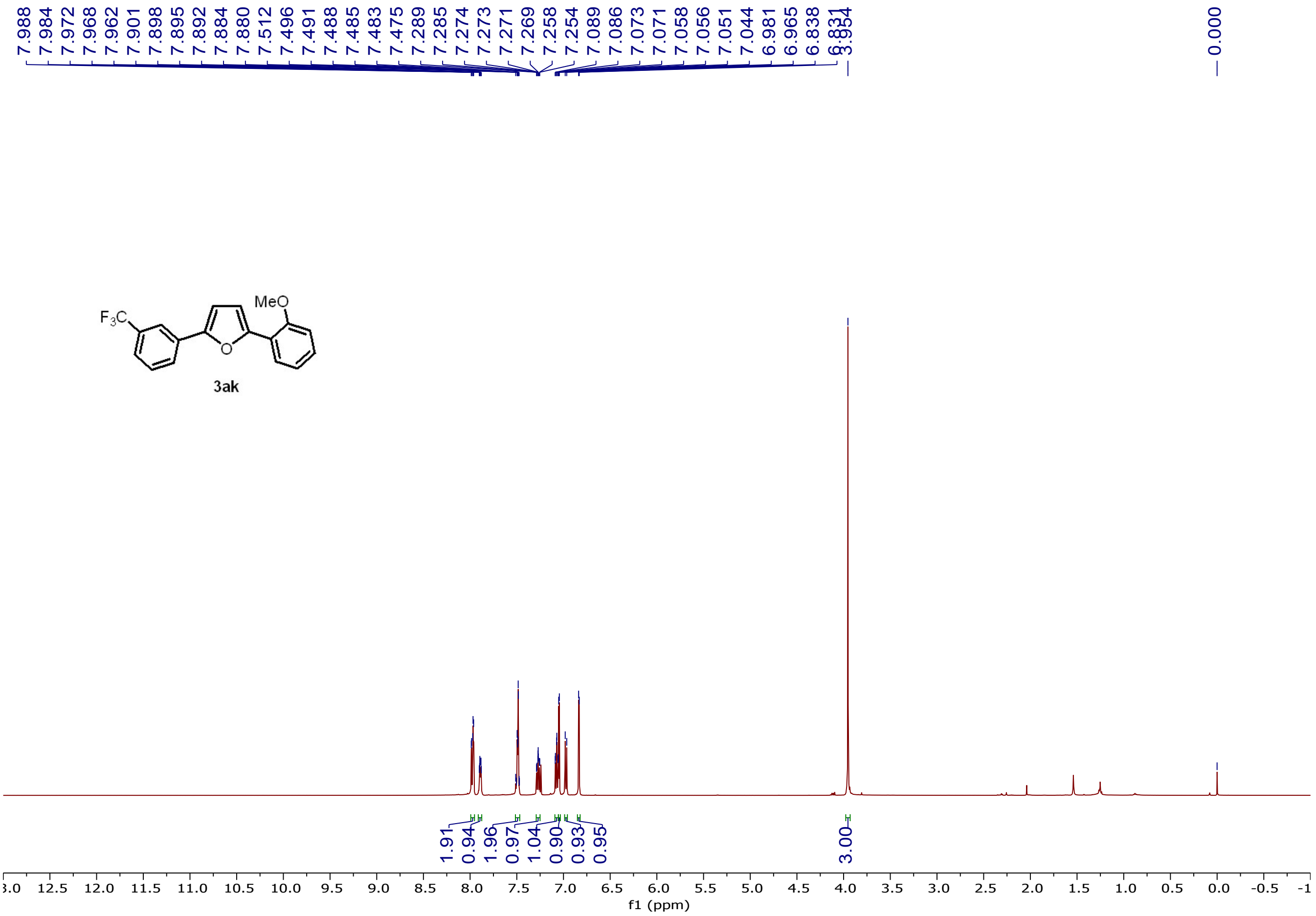
7.988 7.984 7.972 7.968 7.962 7.901 7.898 7.895 7.892 7.884 7.880 7.512 7.496 7.491 7.488 7.485 7.483 7.475 7.289 7.285 7.274 7.273 7.271 7.269 7.258 7.254 7.089 7.086 7.073 7.071 7.058 7.056 7.051 7.044 6.981 6.965 6.838 6.831 3.954
0.000
F_3C
MeO
O
3ak
1.91 0.94 1.96 0.97 1.04 0.90 0.93 0.95
3.00
f1 (ppm)

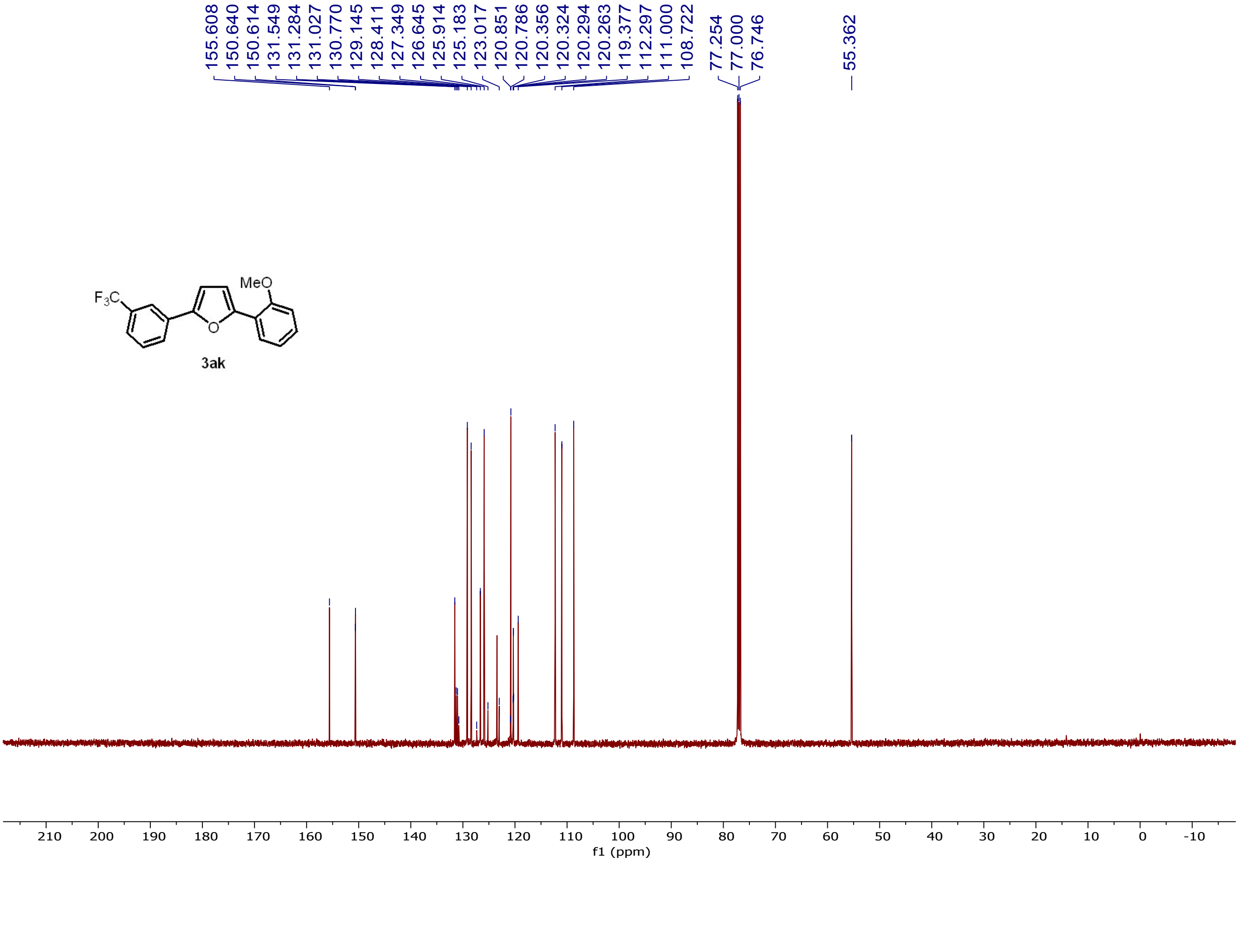
155.608 150.640 150.614 131.549 131.284 131.027 130.770 129.145 128.411 127.349 126.645 125.914 125.183 123.017 120.851 120.786 120.356 120.324 120.294 120.263 119.377 112.297 111.000 108.722
77.254 77.000 76.746
55.362
F_3C
MeO
O
3ak
f1 (ppm)

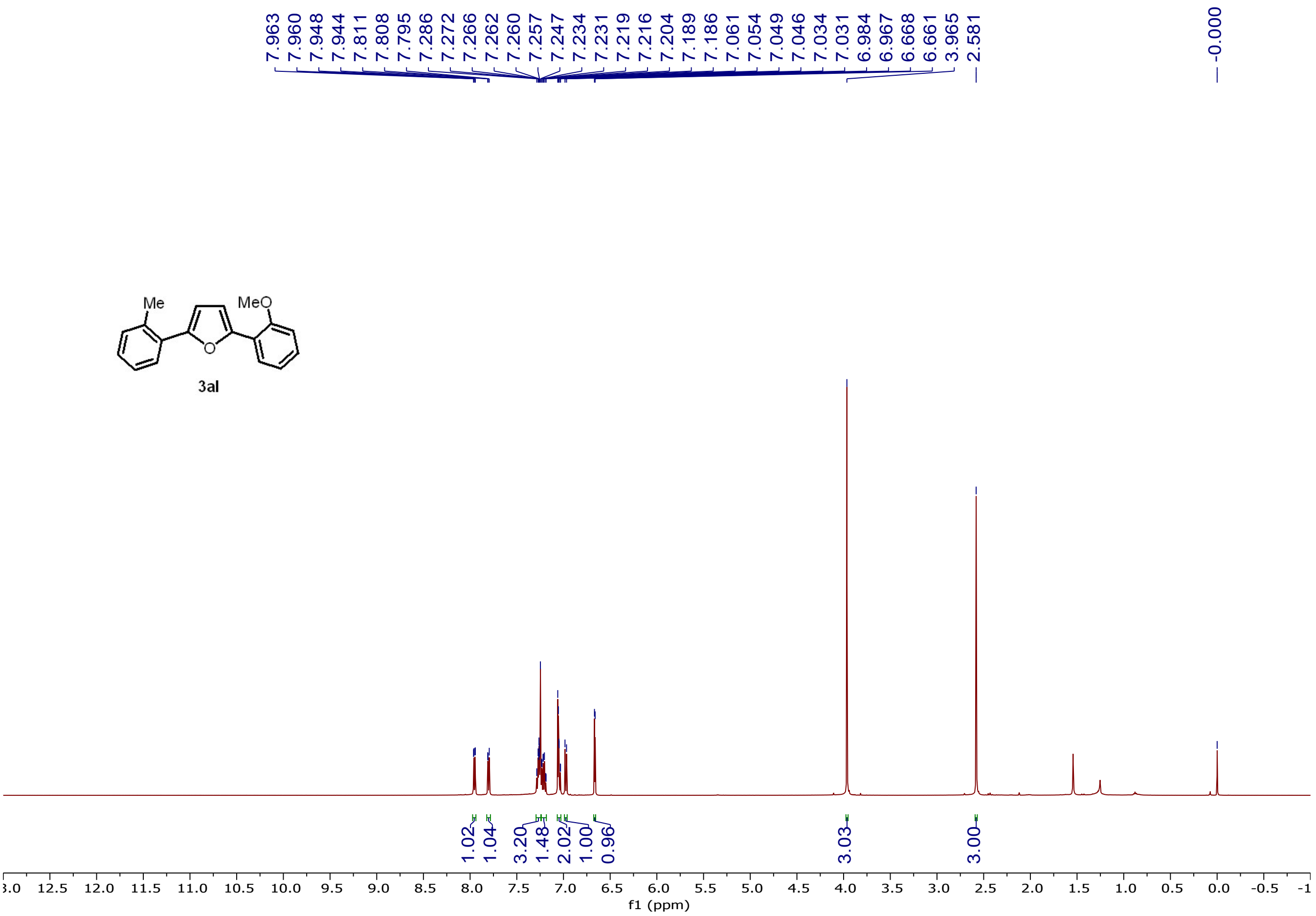
7.963 7.960 7.948 7.944 7.811 7.808 7.795 7.286 7.272 7.266 7.262 7.260 7.257 7.247 7.234 7.231 7.219 7.216 7.204 7.189 7.186 7.061 7.054 7.049 7.046 7.034 7.031 6.984 6.967 6.668 6.661 3.965 2.581
-0.000
Me
MeO
O
3al
1.02 1.04 3.20 1.48 2.02 1.00 0.96 3.03 3.00
f1 (ppm)

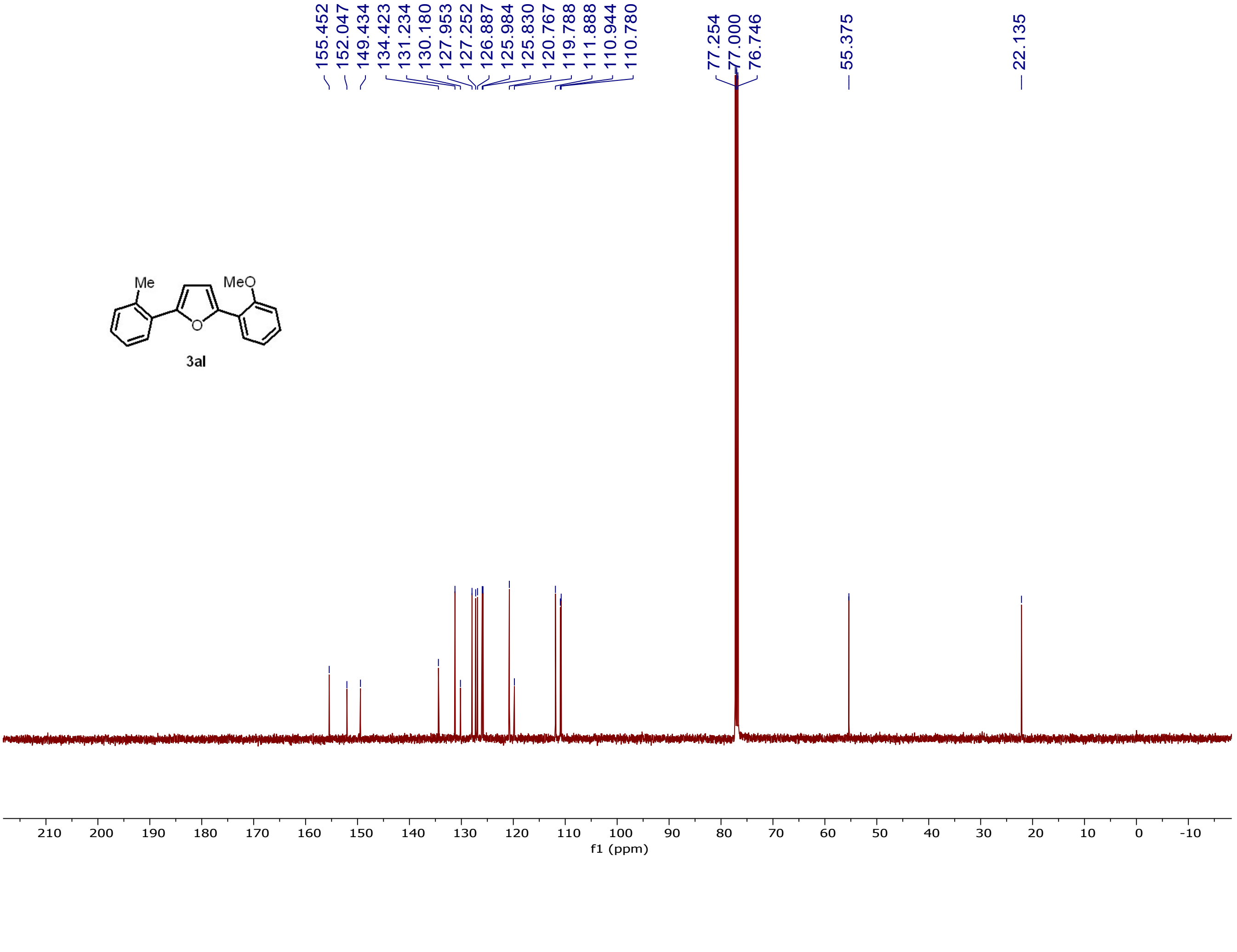
155.452 152.047 149.434 134.423 131.234 130.180 127.953 127.252 126.887 125.984 125.830 120.767 119.788 111.888 110.944 110.780
77.254 77.000 76.746
55.375
22.135
Me
MeO
O
3al
f1 (ppm)

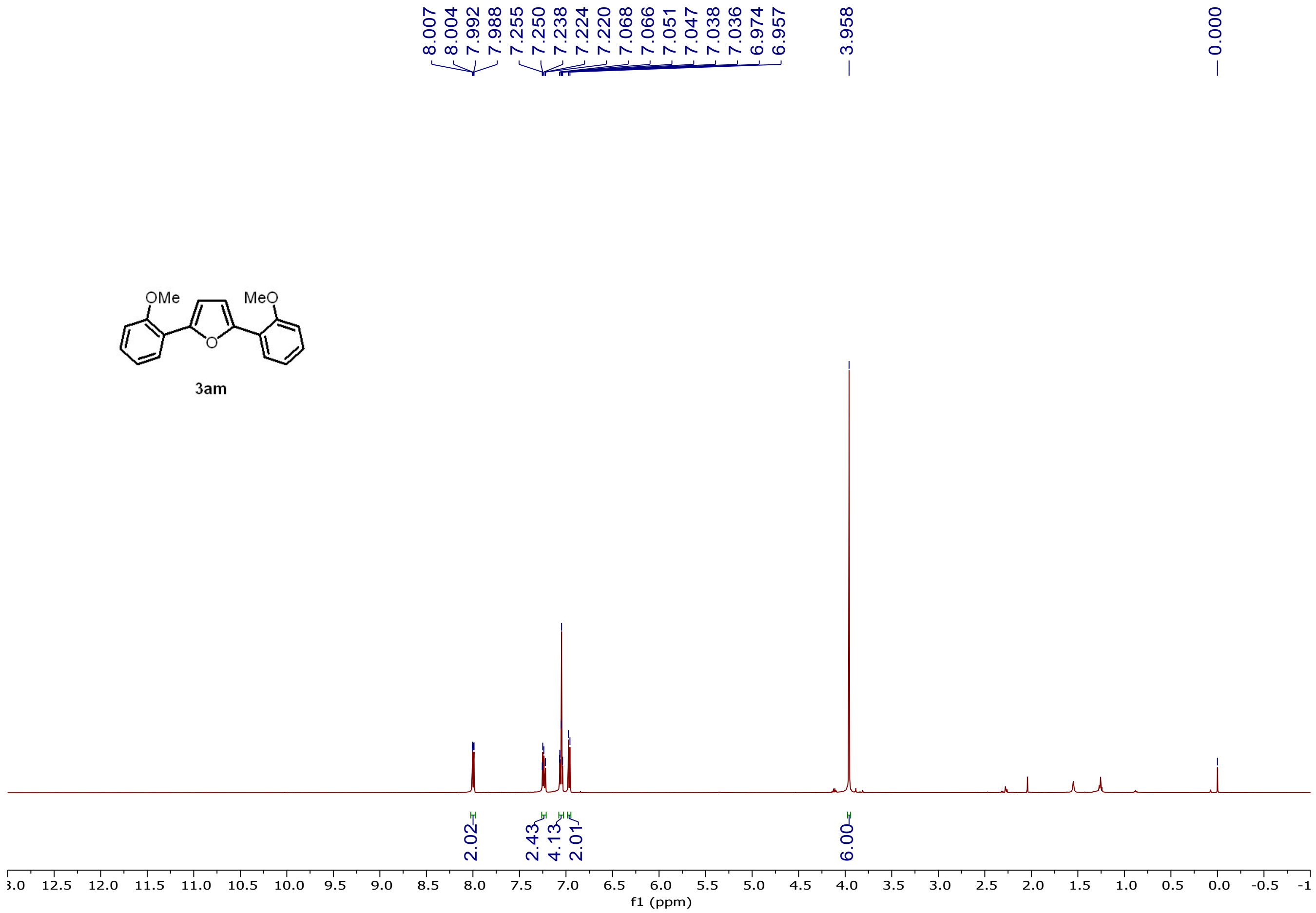
8.007
8.004
7.992
7.988
7.255
7.250
7.238
7.224
7.220
7.068
7.066
7.051
7.047
7.038
7.036
6.974
6.957
3.958
0.000
OMe
MeO
O
3am
2.02
2.43
4.13
2.01
6.00
f1 (ppm)

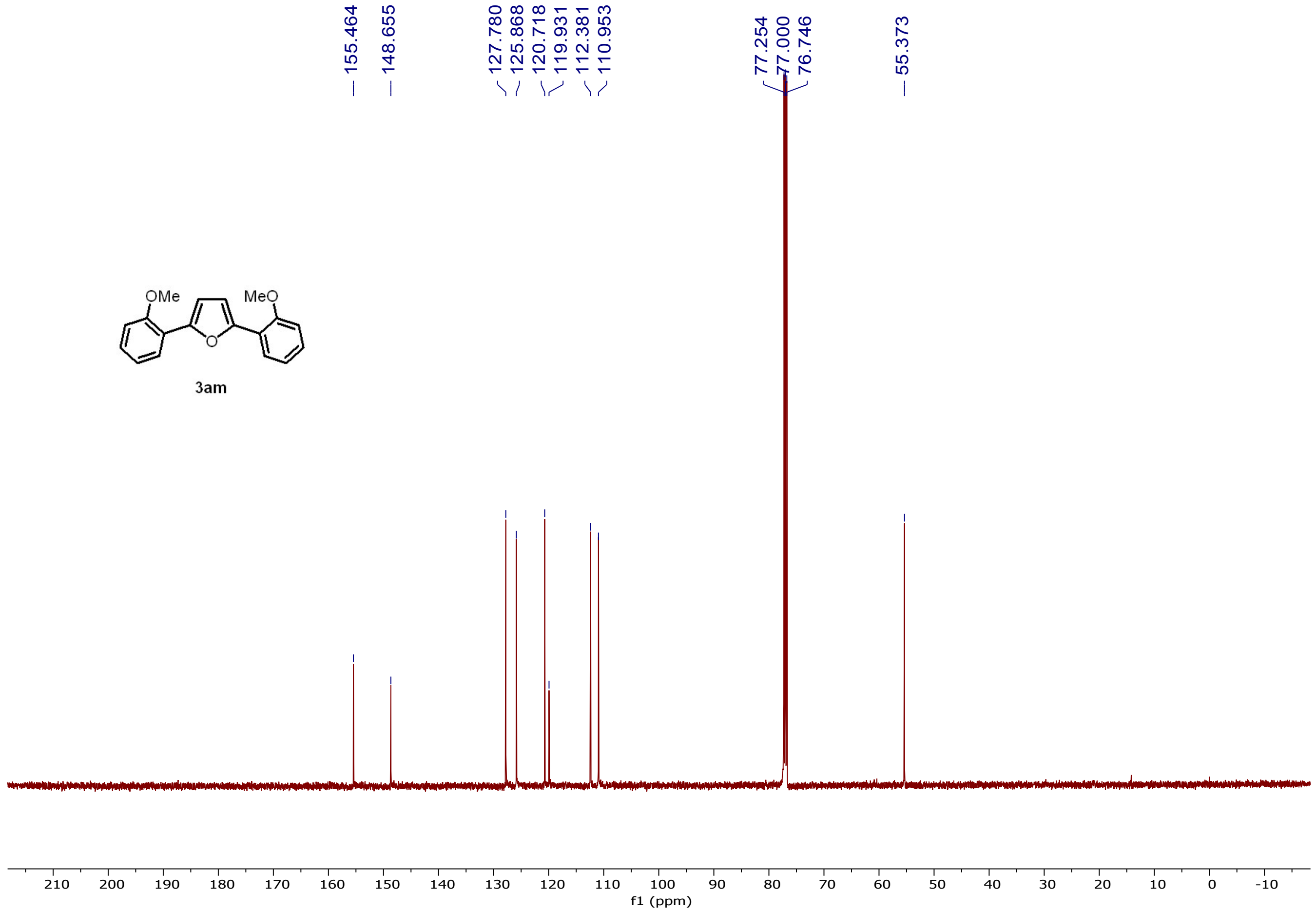
155.464
148.655
127.780
125.868
120.718
119.931
112.381
110.953
77.254
77.000
76.746
55.373
OMe
MeO
O
3am
f1 (ppm)

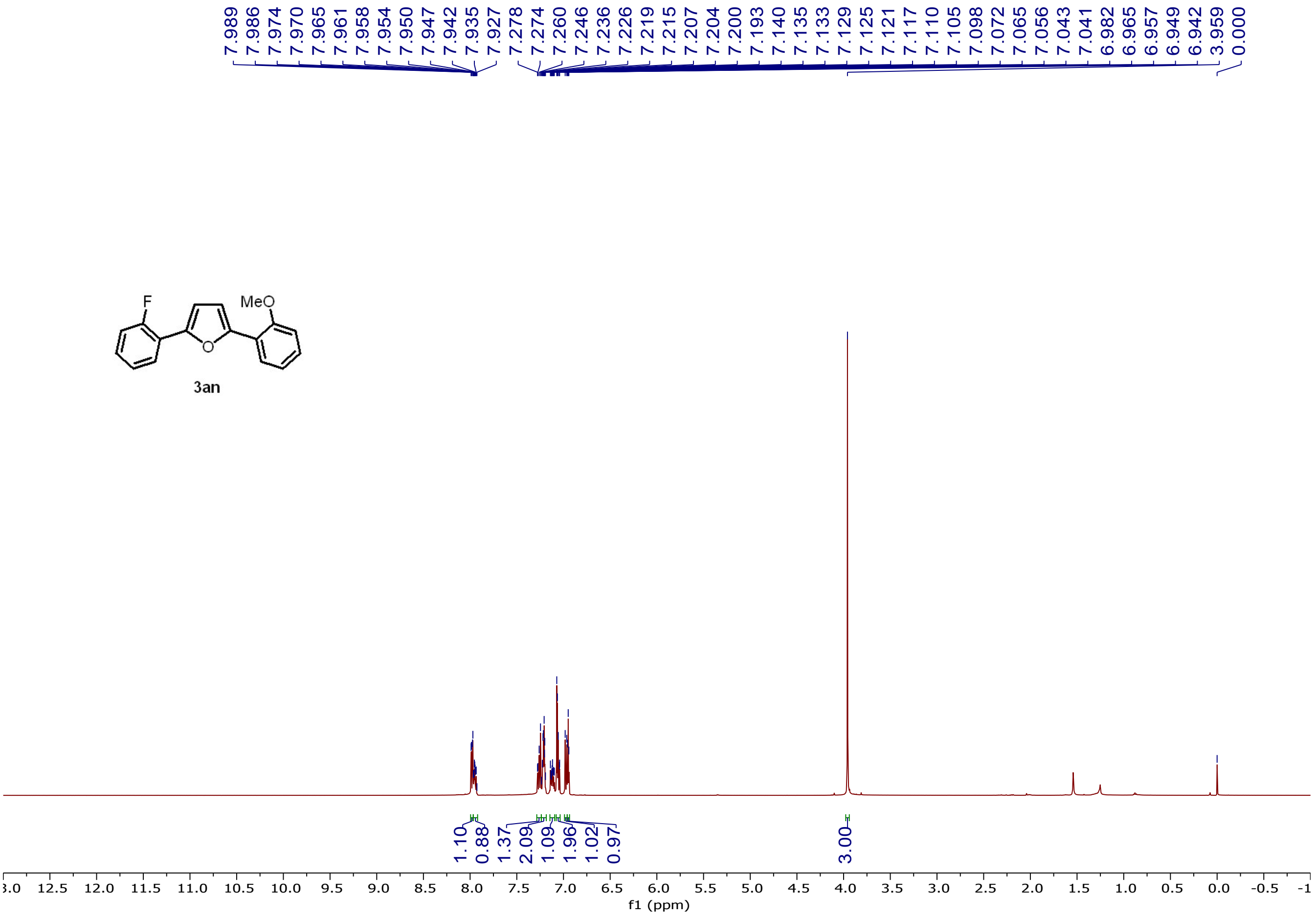
7.989 7.986 7.974 7.970 7.965 7.961 7.958 7.954 7.950 7.947 7.942 7.935 7.927 7.278 7.274 7.260 7.246 7.236 7.226 7.219 7.215 7.207 7.204 7.200 7.193 7.140 7.135 7.133 7.129 7.125 7.121 7.117 7.110 7.105 7.098 7.072 7.065 7.056 7.043 7.041 6.982 6.965 6.957 6.949 6.942 3.959 0.000
F
MeO
O
3an
1.10 0.88 1.37 2.09 1.09 1.96 1.02 0.97 3.00
f1 (ppm)

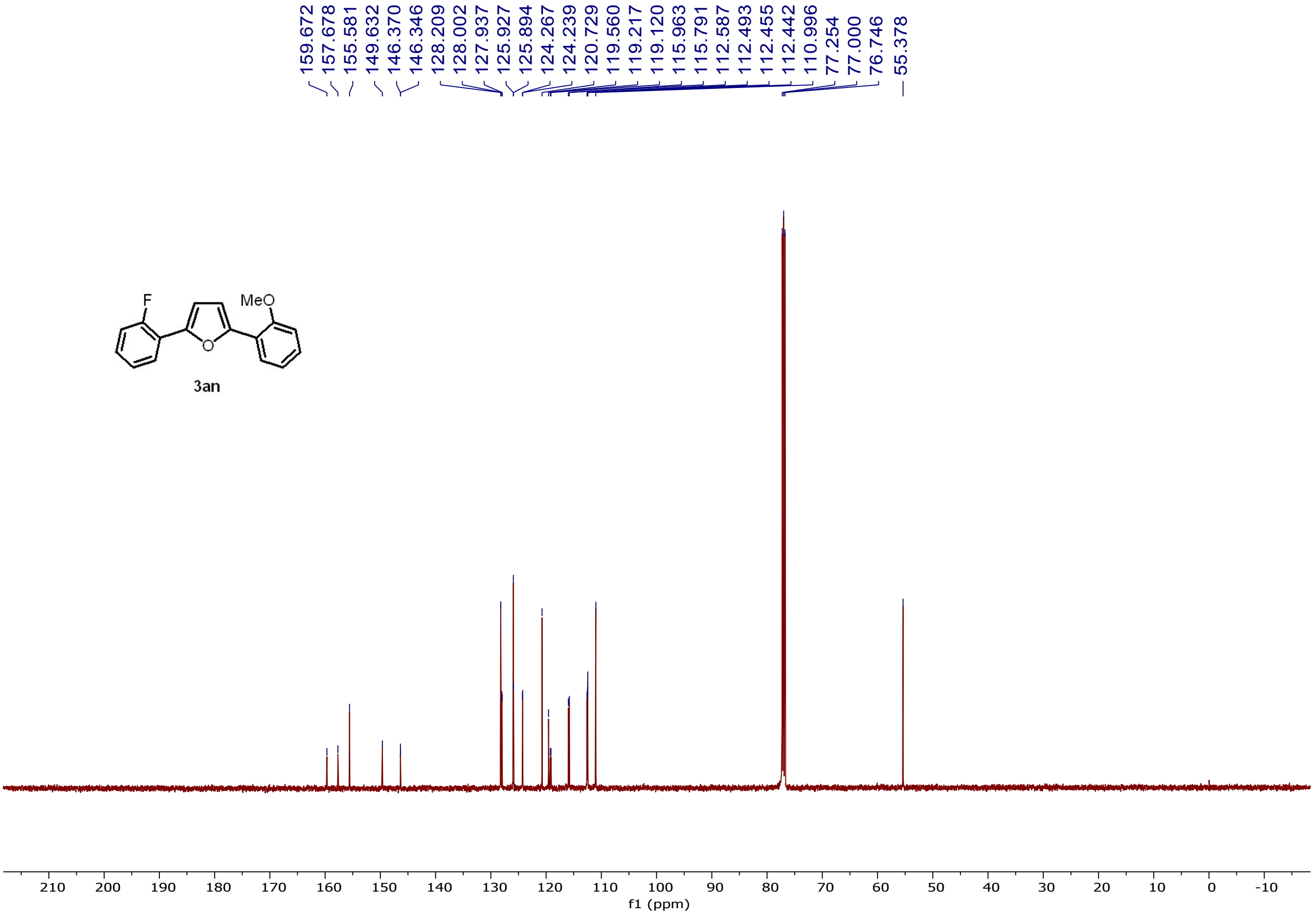
159.672 157.678 155.581 149.632 146.370 146.346 128.209 128.002 127.937 125.927 125.894 124.267 124.239 120.729 119.560 119.217 119.120 115.963 115.791 112.587 112.493 112.455 112.442 110.996 77.254 77.000 76.746 55.378
F
MeO
O
3an
f1 (ppm)

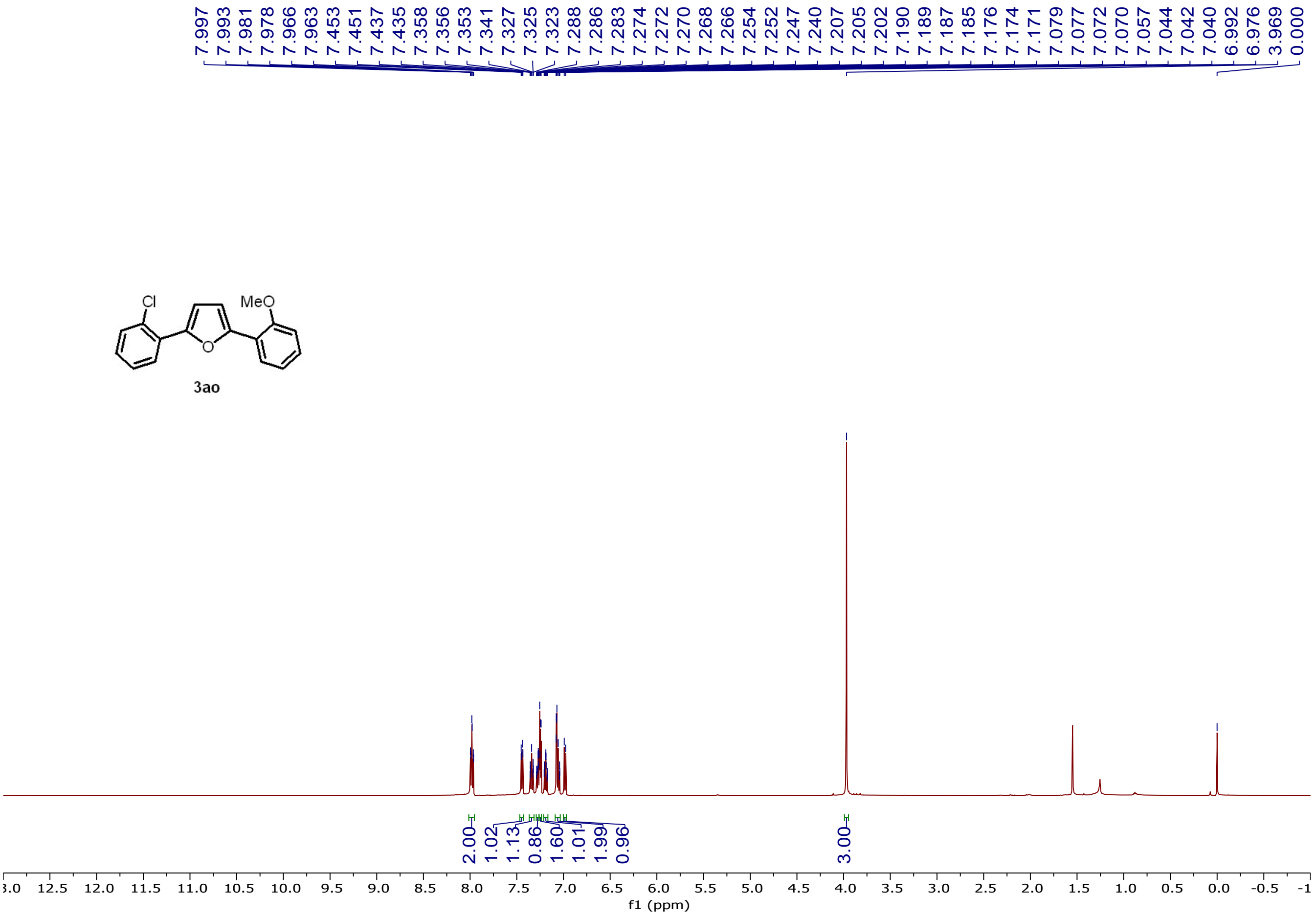
7.997 7.993 7.981 7.978 7.966 7.963 7.453 7.451 7.437 7.435 7.358 7.356 7.353 7.341 7.327 7.325 7.323 7.288 7.286 7.283 7.274 7.272 7.270 7.268 7.266 7.254 7.252 7.247 7.240 7.207 7.205 7.202 7.190 7.189 7.187 7.185 7.176 7.174 7.171 7.079 7.077 7.072 7.070 7.057 7.044 7.042 7.040 6.992 6.976 3.969 0.000
Cl
MeO
O
3ao
2.00 1.02 1.13 0.86 1.60 1.01 1.99 0.96 3.00
f1 (ppm)

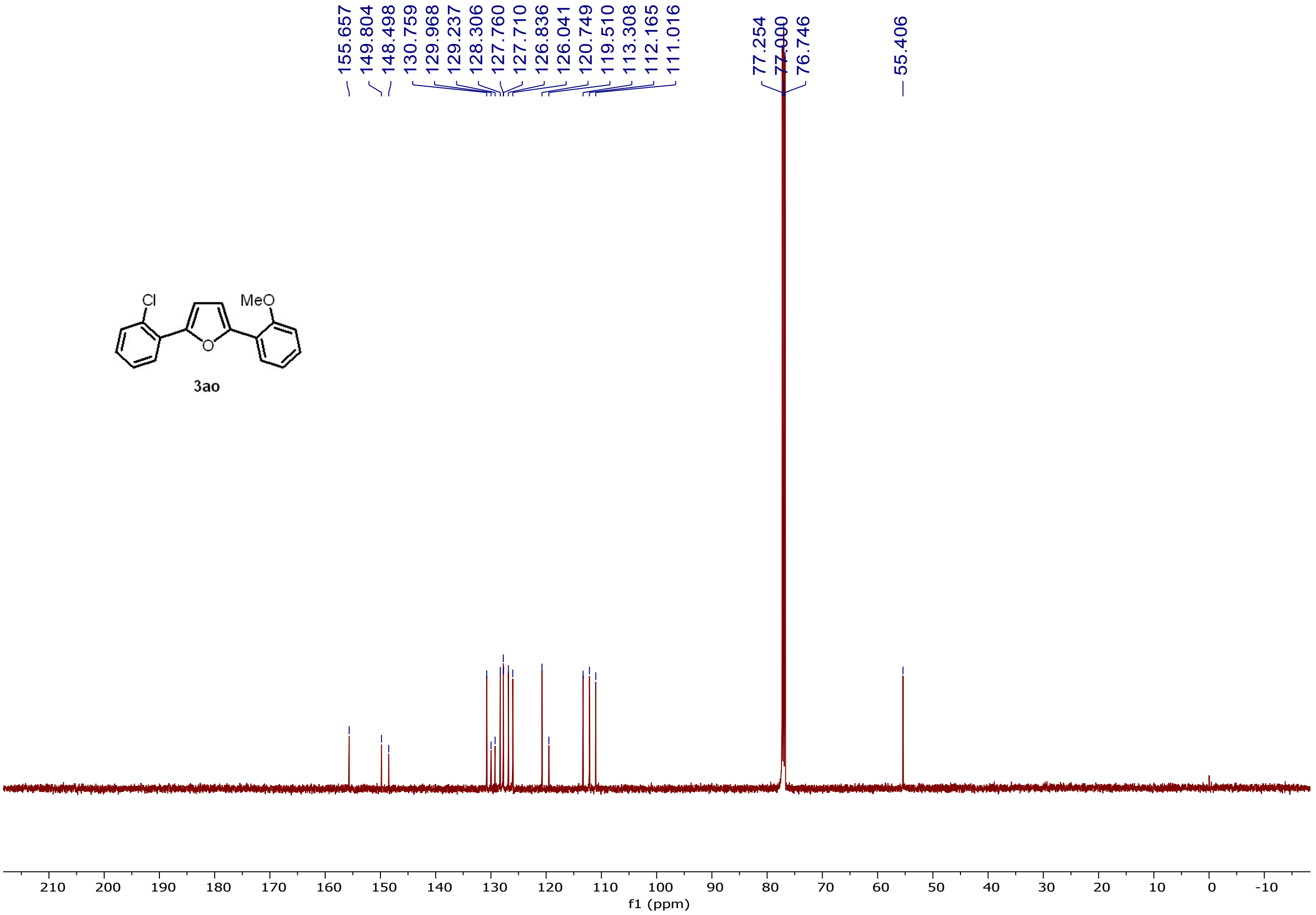
155.657 149.804 148.498 130.759 129.968 129.237 128.306 127.760 127.710 126.836 126.041 120.749 119.510 113.308 112.165 111.016 77.254 77.000 76.746 55.406
Cl
MeO
O
3ao
f1 (ppm)

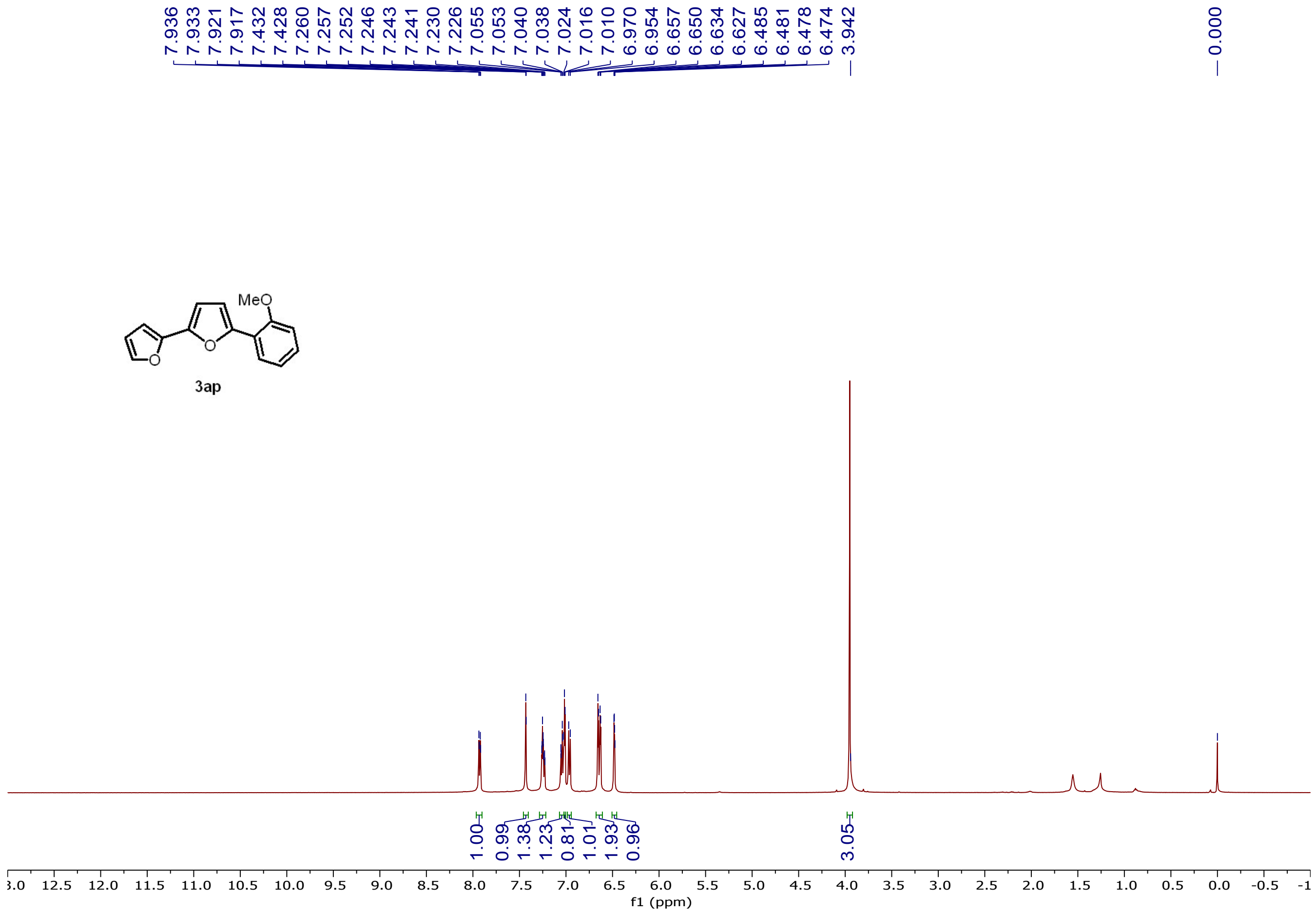
7.936 7.933 7.921 7.917 7.432 7.428 7.260 7.257 7.252 7.246 7.243 7.241 7.230 7.226 7.055 7.053 7.040 7.038 7.024 7.016 7.010 6.970 6.954 6.657 6.650 6.634 6.627 6.485 6.481 6.478 6.474 3.942
0.000
MeO
O
O
3ap
1.00 0.99 1.38 1.23 0.81 1.01 1.93 0.96
3.05
f1 (ppm)

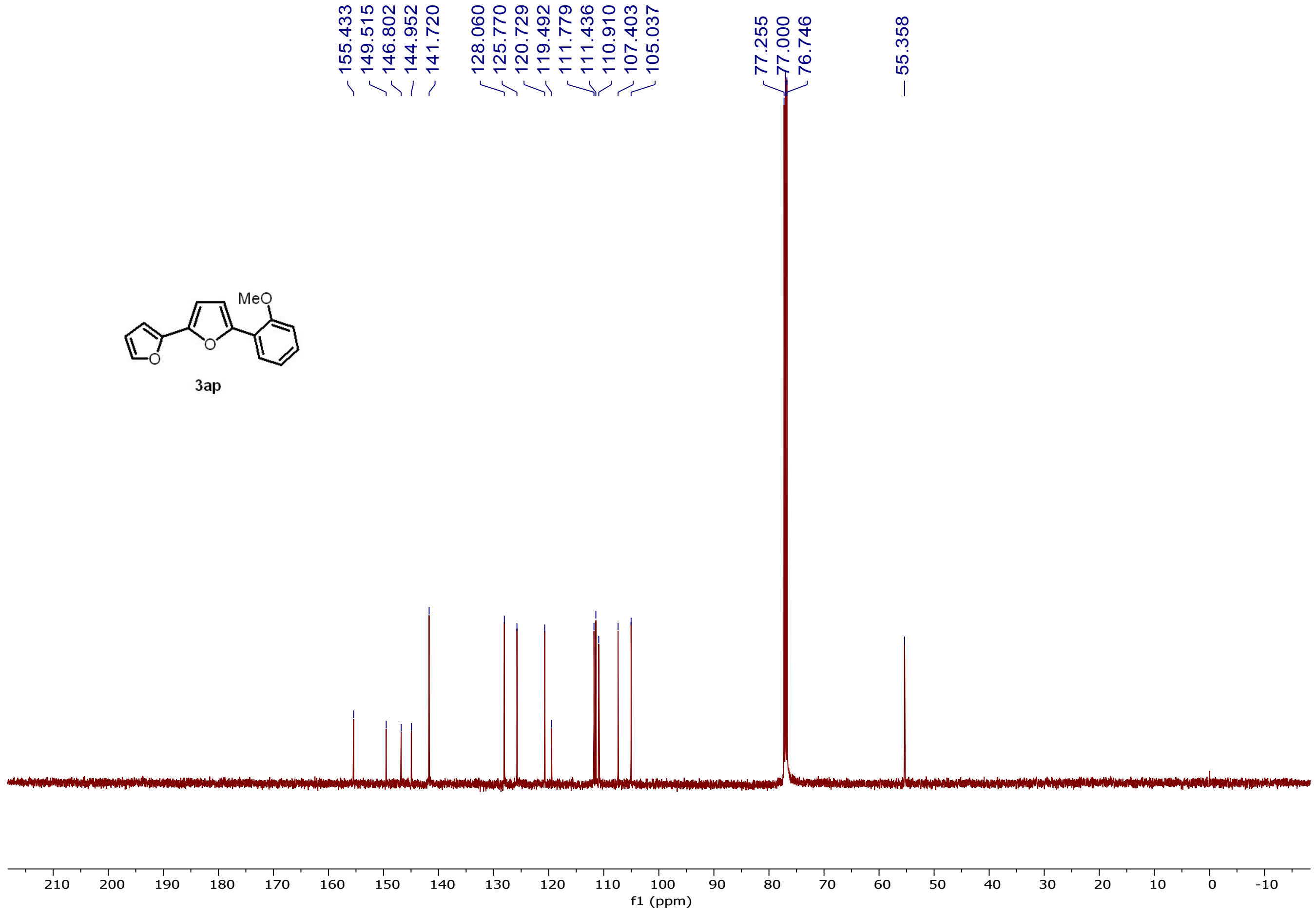
155.433 149.515 146.802 144.952 141.720
128.060 125.770 120.729 119.492 111.779 111.436 110.910 107.403 105.037
77.255 77.000 76.746
55.358
MeO
O
O
3ap
f1 (ppm)

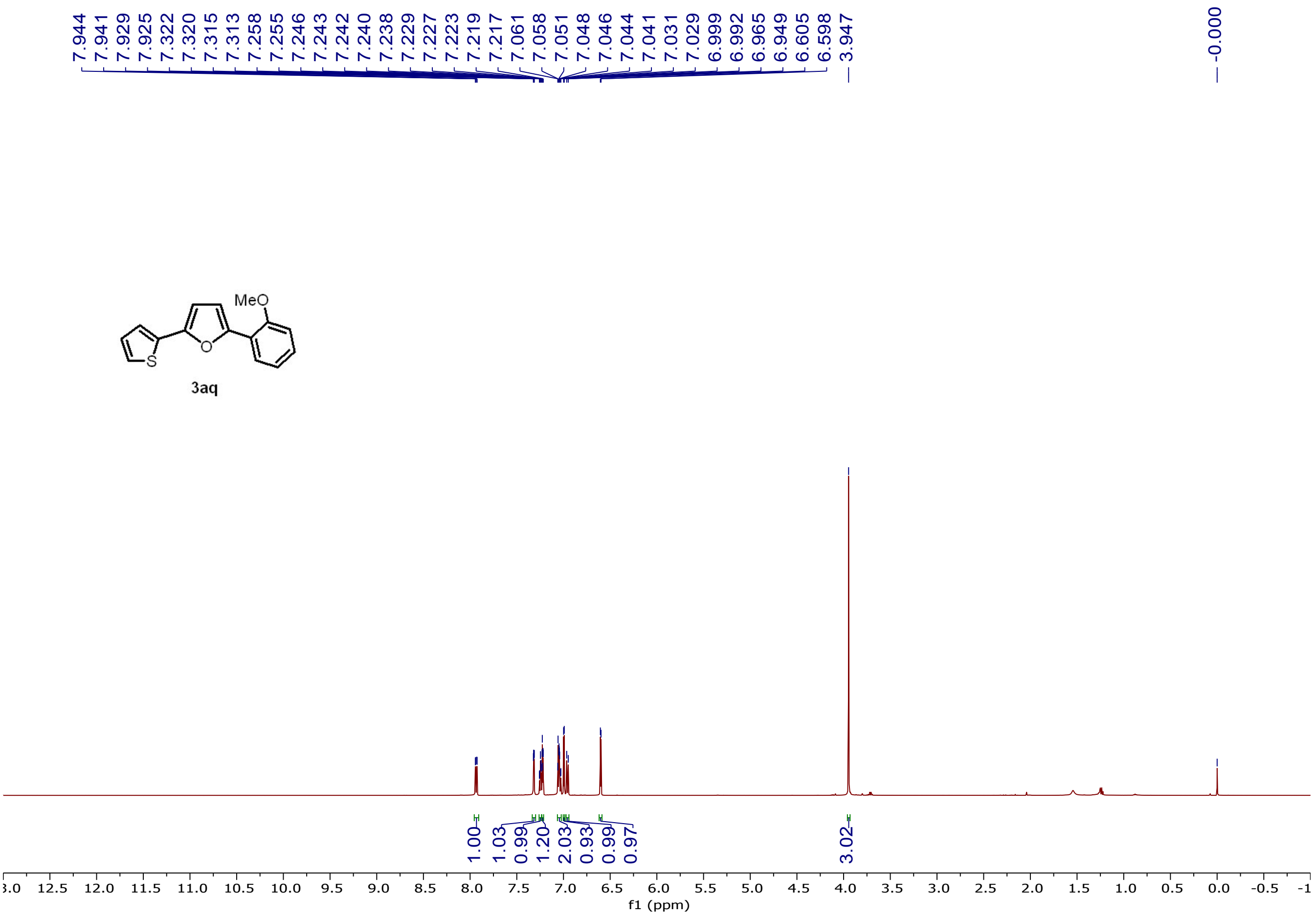
7.944 7.941 7.929 7.925 7.322 7.320 7.315 7.313 7.258 7.255 7.246 7.243 7.242 7.240 7.238 7.229 7.227 7.223 7.219 7.217 7.061 7.058 7.051 7.048 7.046 7.044 7.041 7.031 7.029 6.999 6.992 6.965 6.949 6.605 6.598 3.947
-0.000
MeO
O
S
3aq
1.00 1.03 0.99 1.20 2.03 0.93 0.99 0.97
3.02
f1 (ppm)

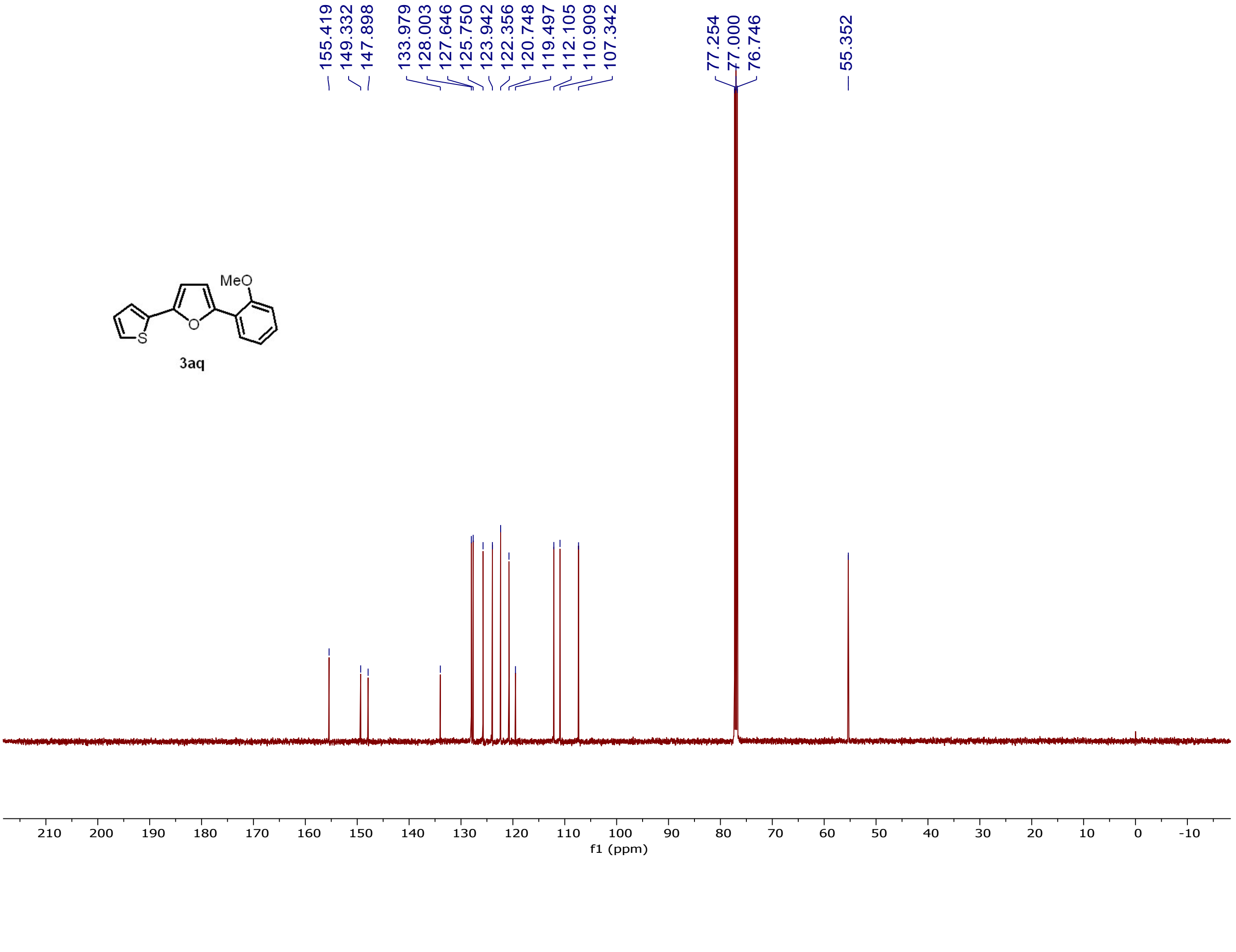
155.419 149.332 147.898
133.979 128.003 127.646 125.750 123.942 122.356 120.748 119.497 112.105 110.909 107.342
77.254 77.000 76.746
55.352
MeO
O
S
3aq
f1 (ppm)

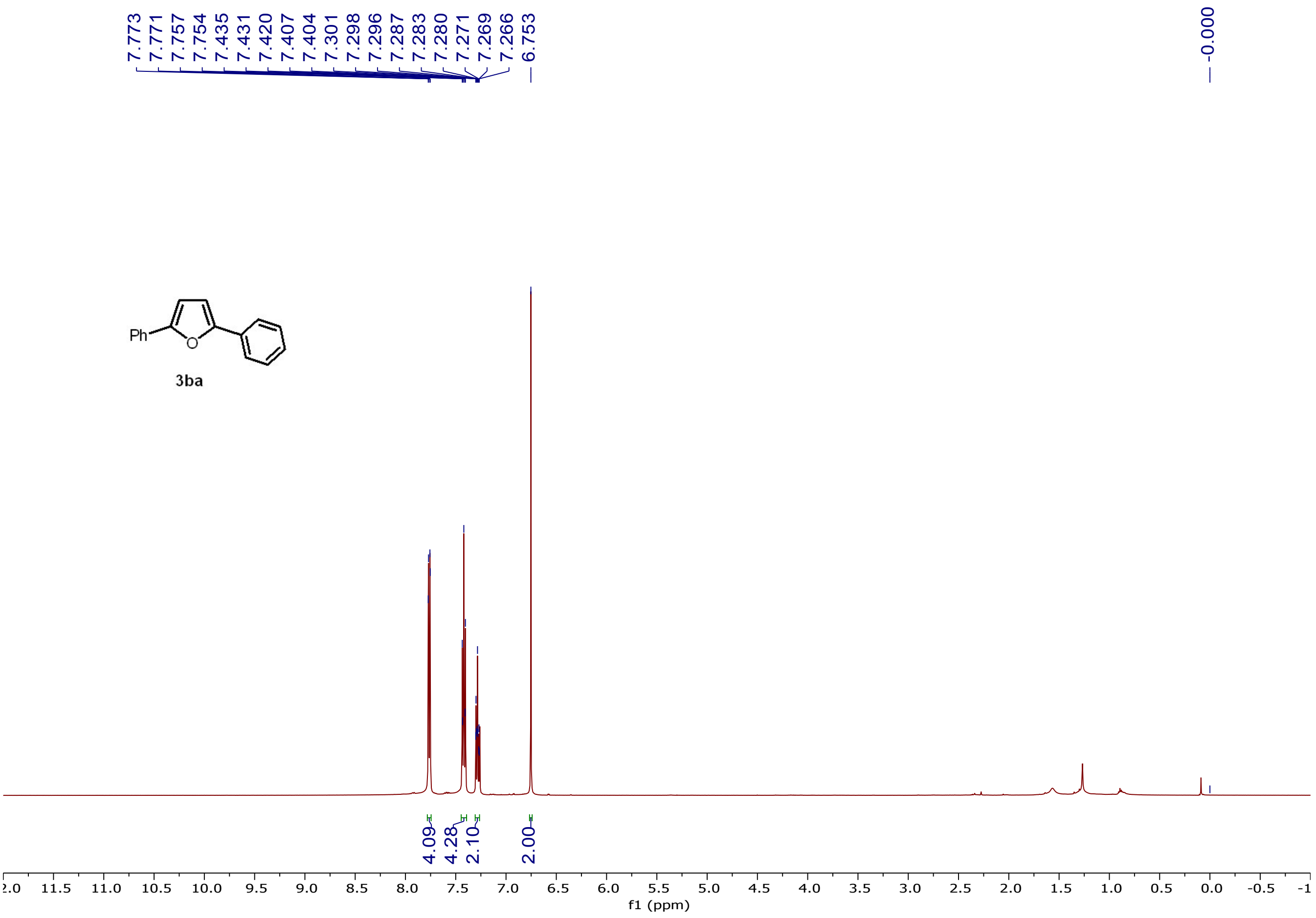
7.773
7.771
7.757
7.754
7.435
7.431
7.420
7.407
7.404
7.301
7.298
7.296
7.287
7.283
7.280
7.271
7.269
7.266
6.753
-0.000
Ph
O
3ba
4.09
4.28
2.10
2.00
f1 (ppm)

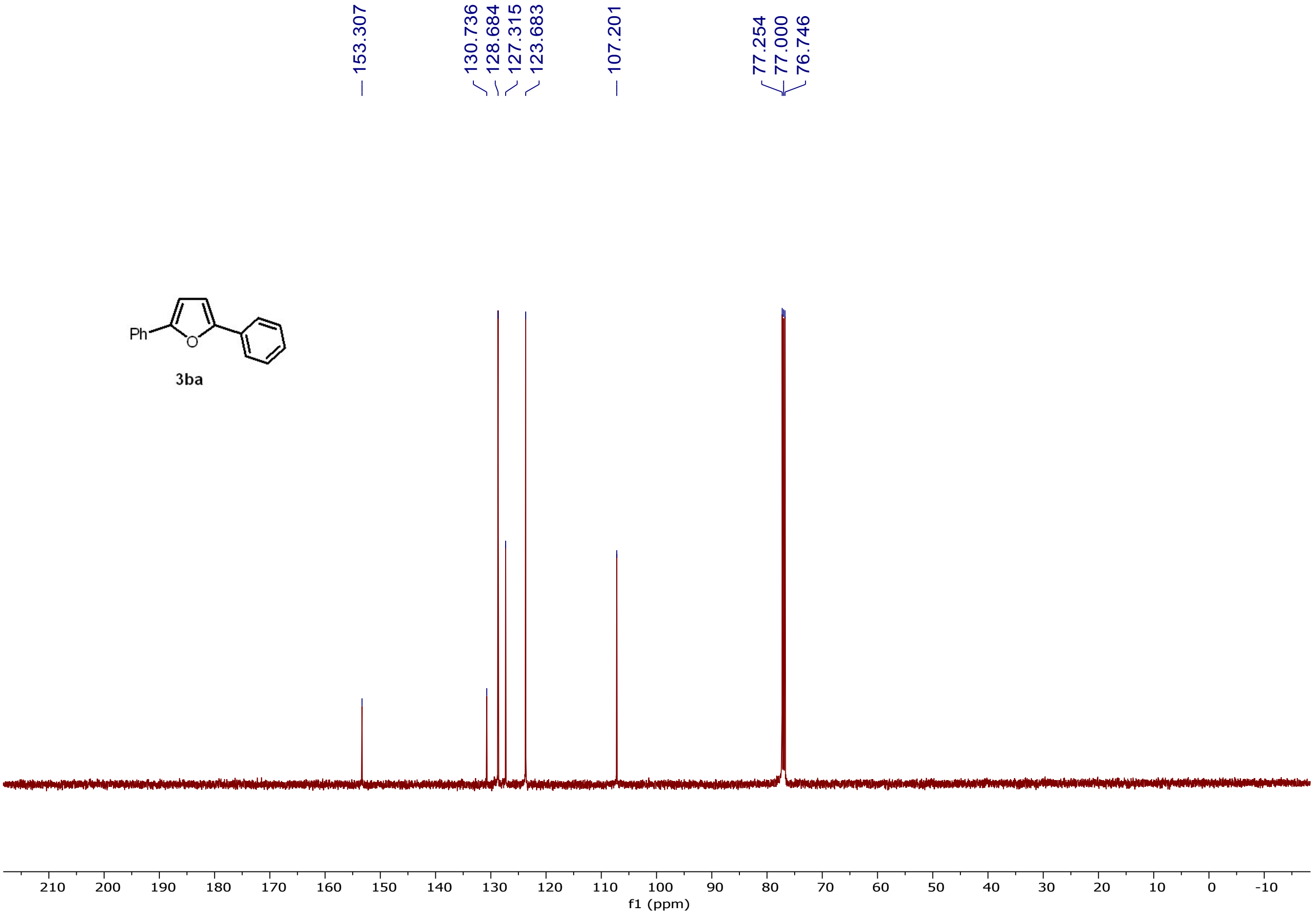
153.307
130.736
128.684
127.315
123.683
107.201
77.254
77.000
76.746
Ph
O
3ba
f1 (ppm)

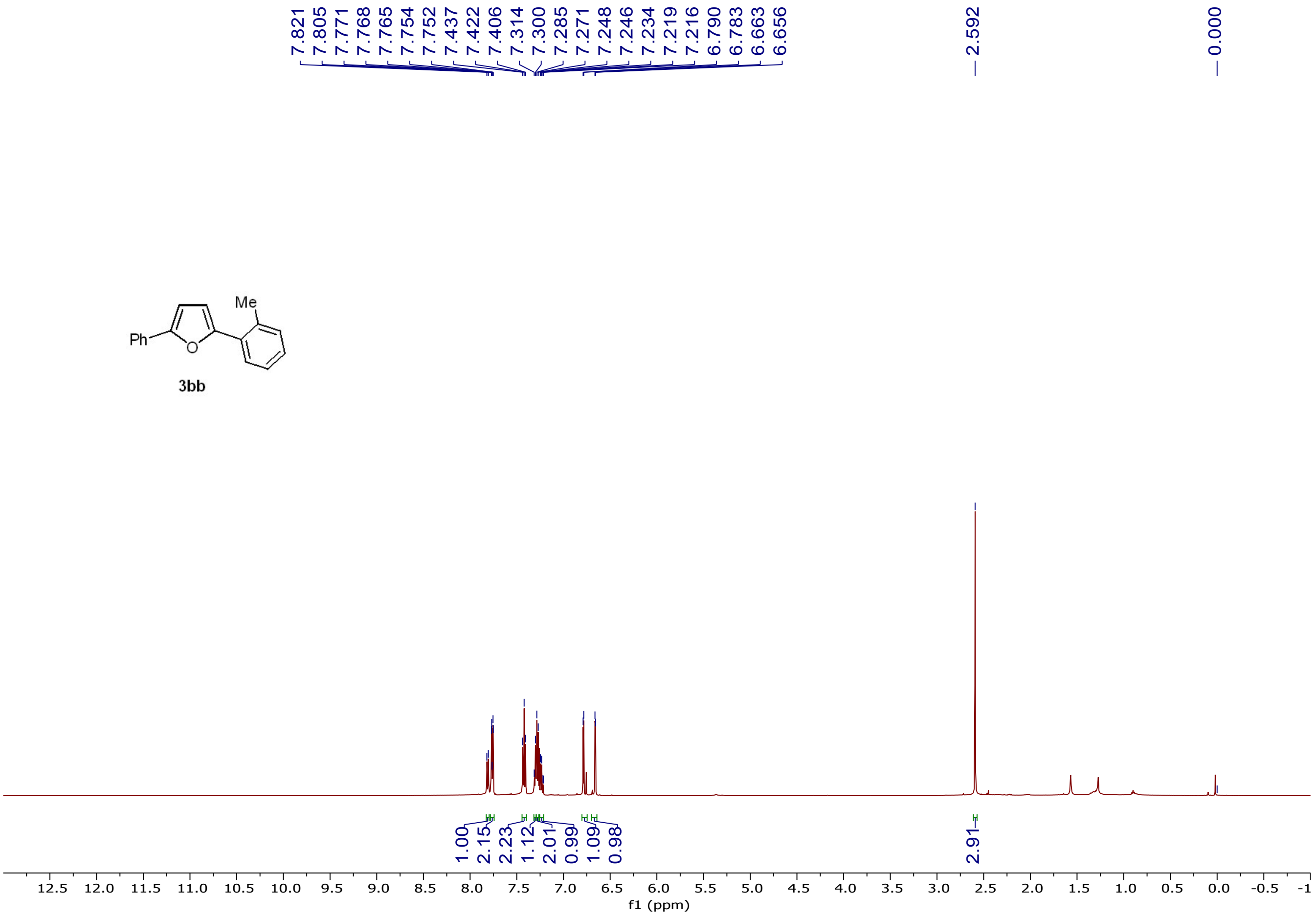
7.821
7.805
7.771
7.768
7.765
7.754
7.752
7.437
7.422
7.406
7.314
7.300
7.285
7.271
7.248
7.246
7.234
7.219
7.216
6.790
6.783
6.663
6.656
2.592
0.000
Ph
O
Me
3bb
1.00
2.15
2.23
1.12
2.01
0.99
1.09
0.98
2.91
f1 (ppm)

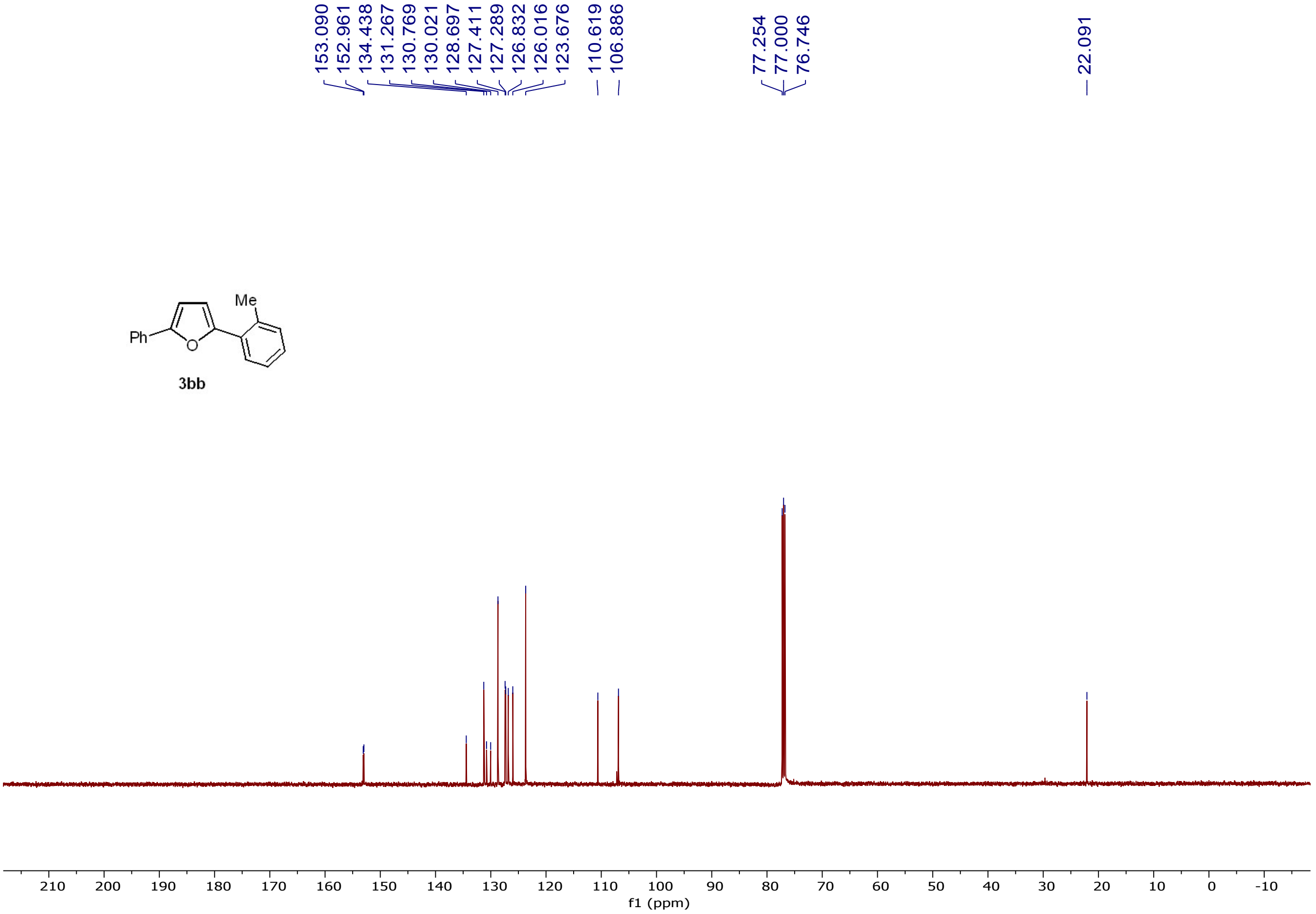
153.090
152.961
134.438
131.267
130.769
130.021
128.697
127.411
127.289
126.832
126.016
123.676
110.619
106.886
77.254
77.000
76.746
22.091
Ph
O
Me
3bb
f1 (ppm)

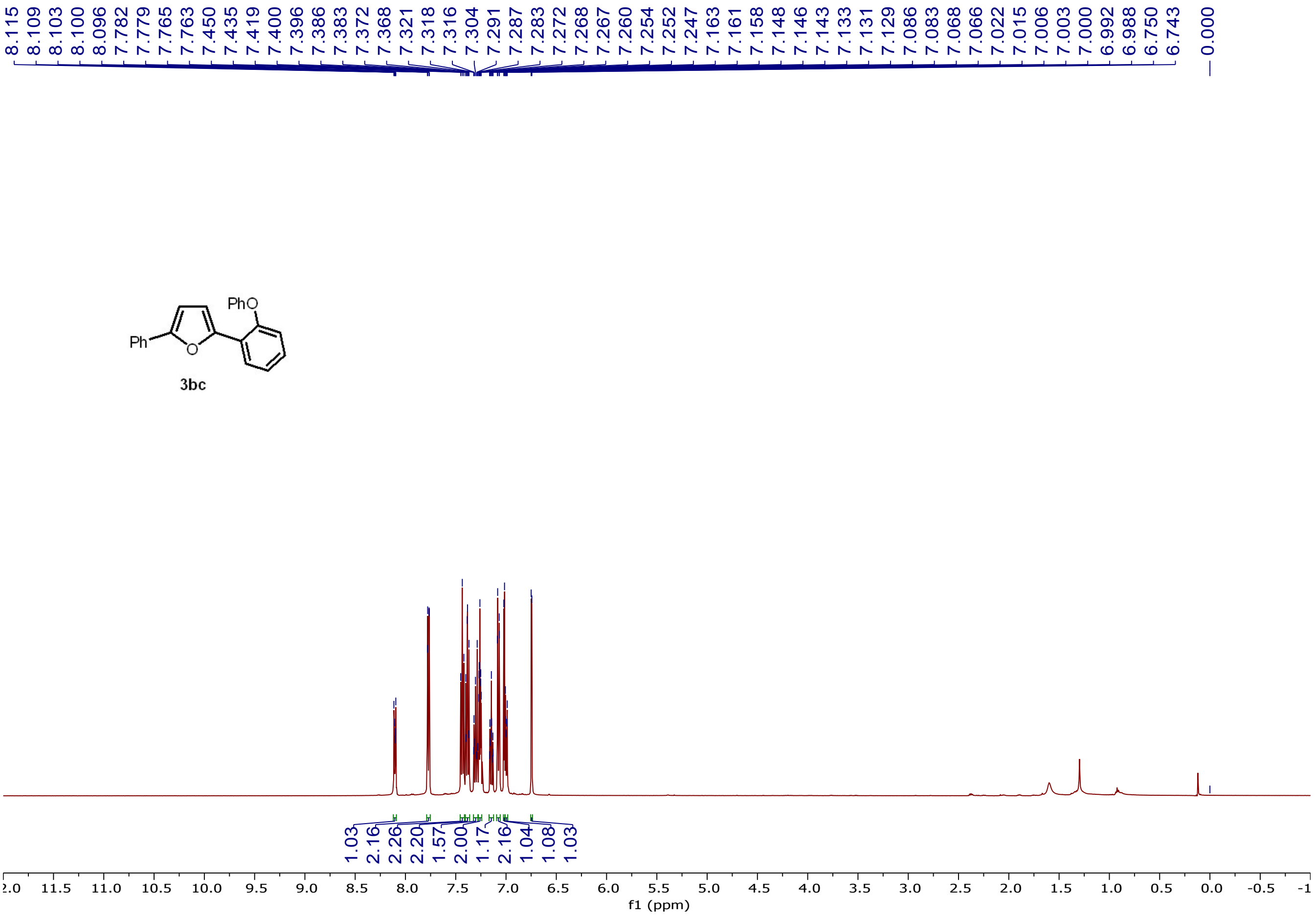
8.115 8.109 8.103 8.100 8.096 7.782 7.779 7.765 7.763 7.450 7.435 7.419 7.400 7.396 7.386 7.383 7.372 7.368 7.321 7.318 7.316 7.304 7.291 7.287 7.283 7.272 7.268 7.267 7.260 7.254 7.252 7.247 7.163 7.161 7.158 7.148 7.146 7.143 7.133 7.131 7.129 7.086 7.083 7.068 7.066 7.022 7.015 7.006 7.003 7.000 6.992 6.988 6.750 6.743
0.000
PhO
Ph
O
3bc
1.03 2.16 2.26 2.20 1.57 2.00 1.17 2.16 1.04 1.08 1.03
f1 (ppm)

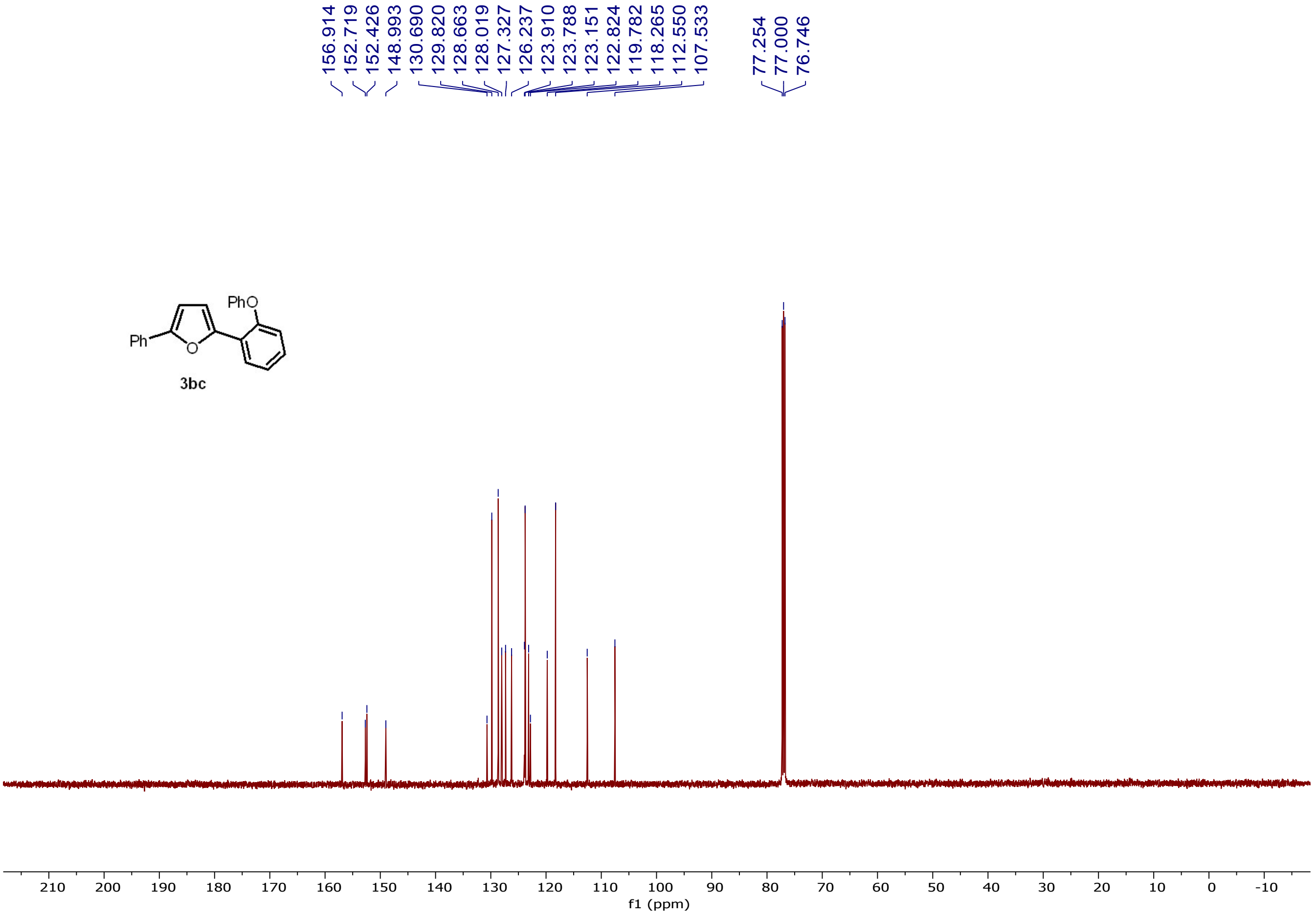
156.914 152.719 152.426 148.993 130.690 129.820 128.663 128.019 127.327 126.237 123.910 123.788 123.151 122.824 119.782 118.265 112.550 107.533
77.254 77.000 76.746
PhO
Ph
O
3bc
f1 (ppm)

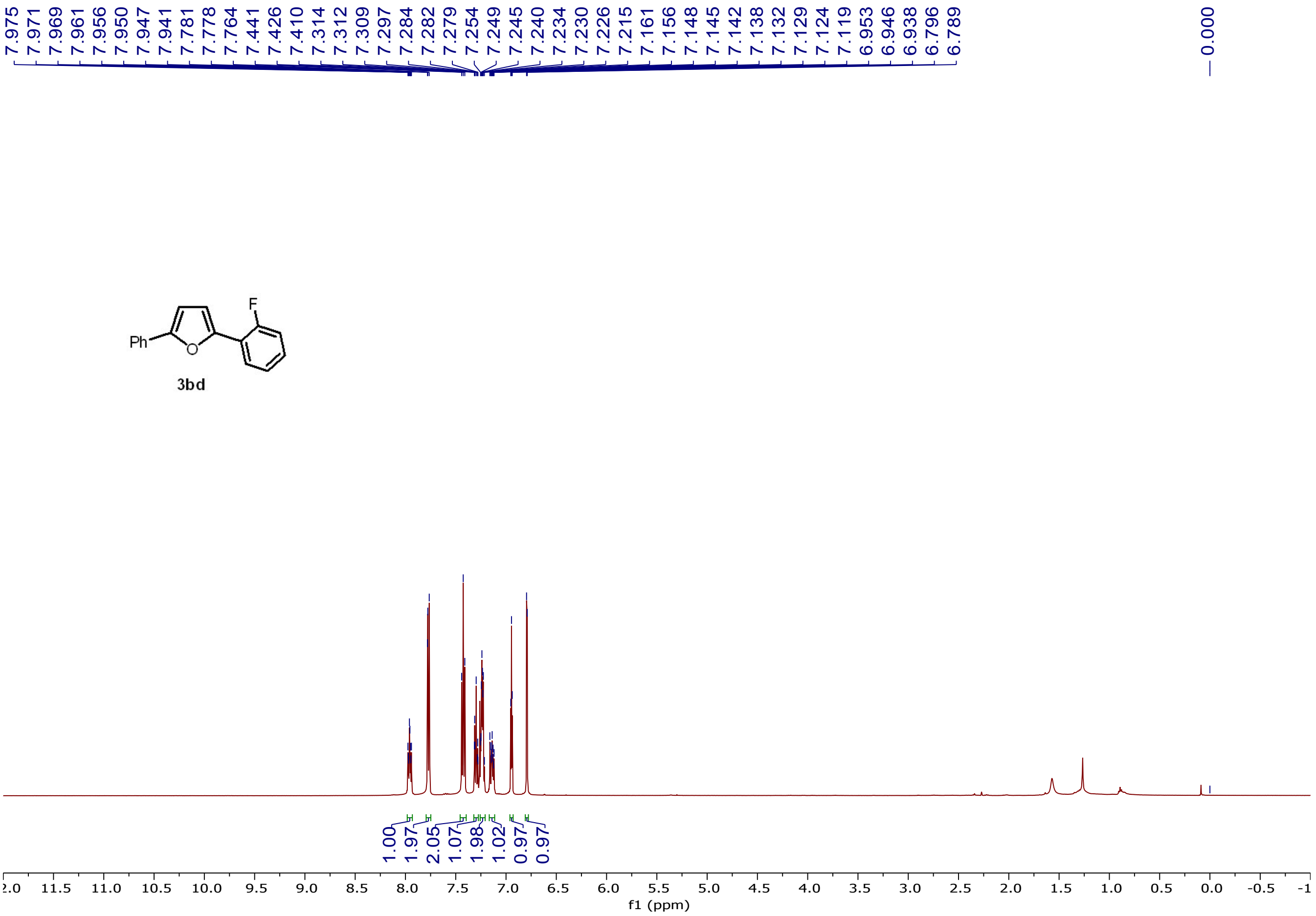
7.975 7.971 7.969 7.961 7.956 7.950 7.947 7.941 7.781 7.778 7.764 7.441 7.426 7.410 7.314 7.312 7.309 7.297 7.284 7.282 7.279 7.254 7.249 7.245 7.240 7.234 7.230 7.226 7.215 7.161 7.156 7.148 7.145 7.142 7.138 7.132 7.129 7.124 7.119 6.953 6.946 6.938 6.796 6.789
0.000
F
Ph
O
3bd
1.00 1.97 2.05 1.07 1.98 1.02 0.97 0.97
f1 (ppm)

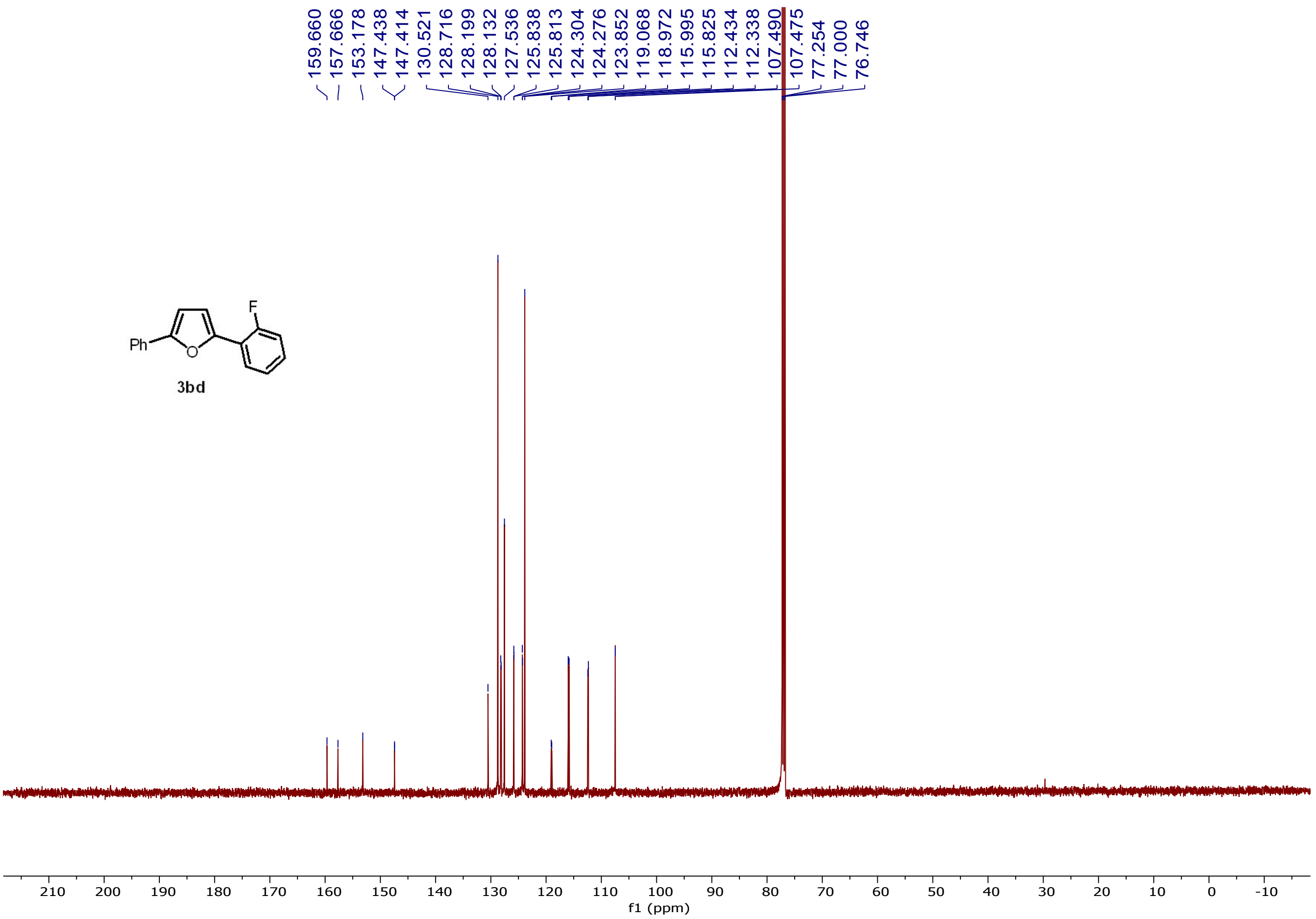
159.660 157.666 153.178 147.438 147.414 130.521 128.716 128.199 128.132 127.536 125.838 125.813 124.304 124.276 123.852 119.068 118.972 115.995 115.825 112.434 112.338 107.490 107.475 77.254 77.000 76.746
F
Ph
O
3bd
f1 (ppm)

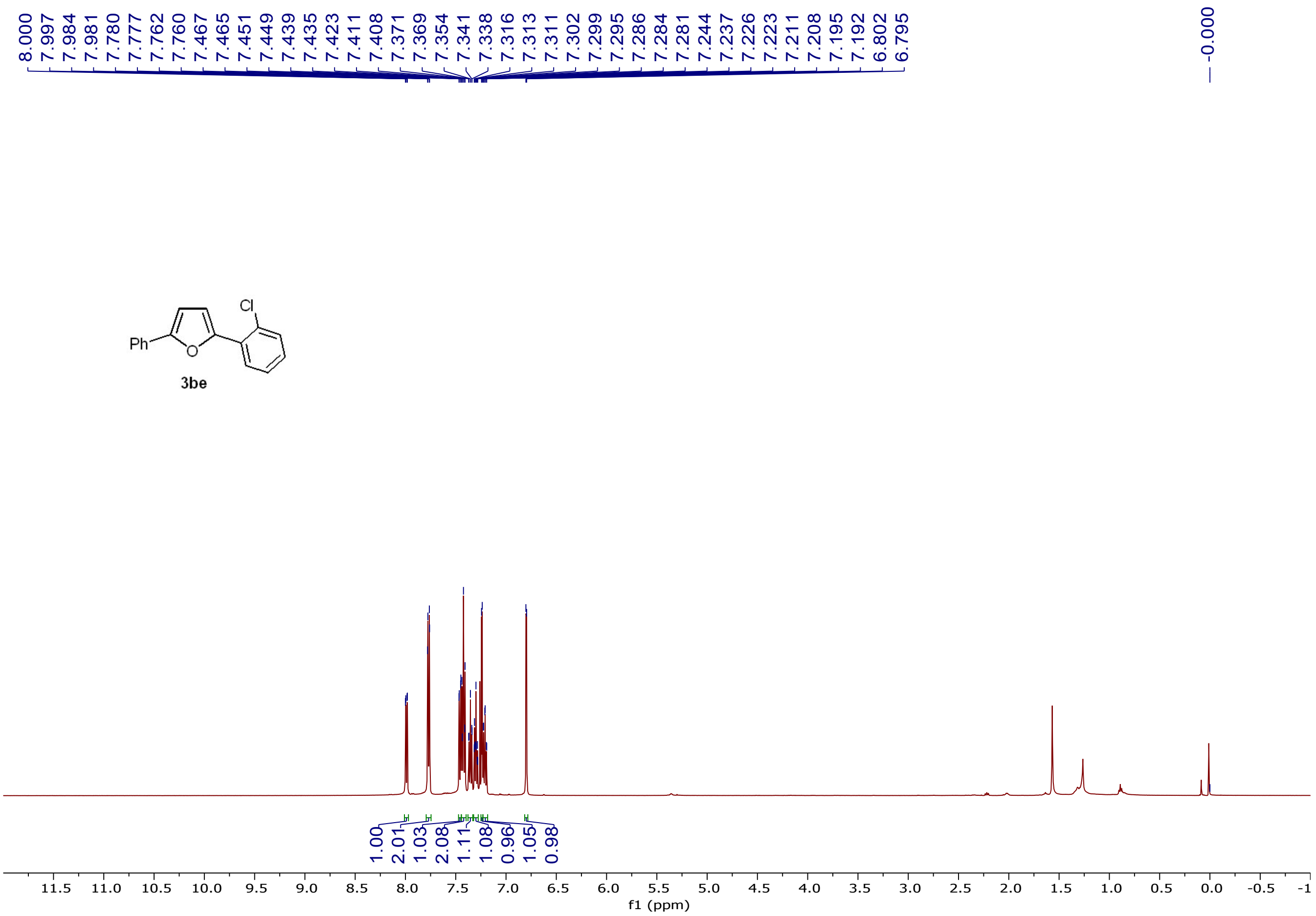
Ph
Cl
O
3be
f1 (ppm)

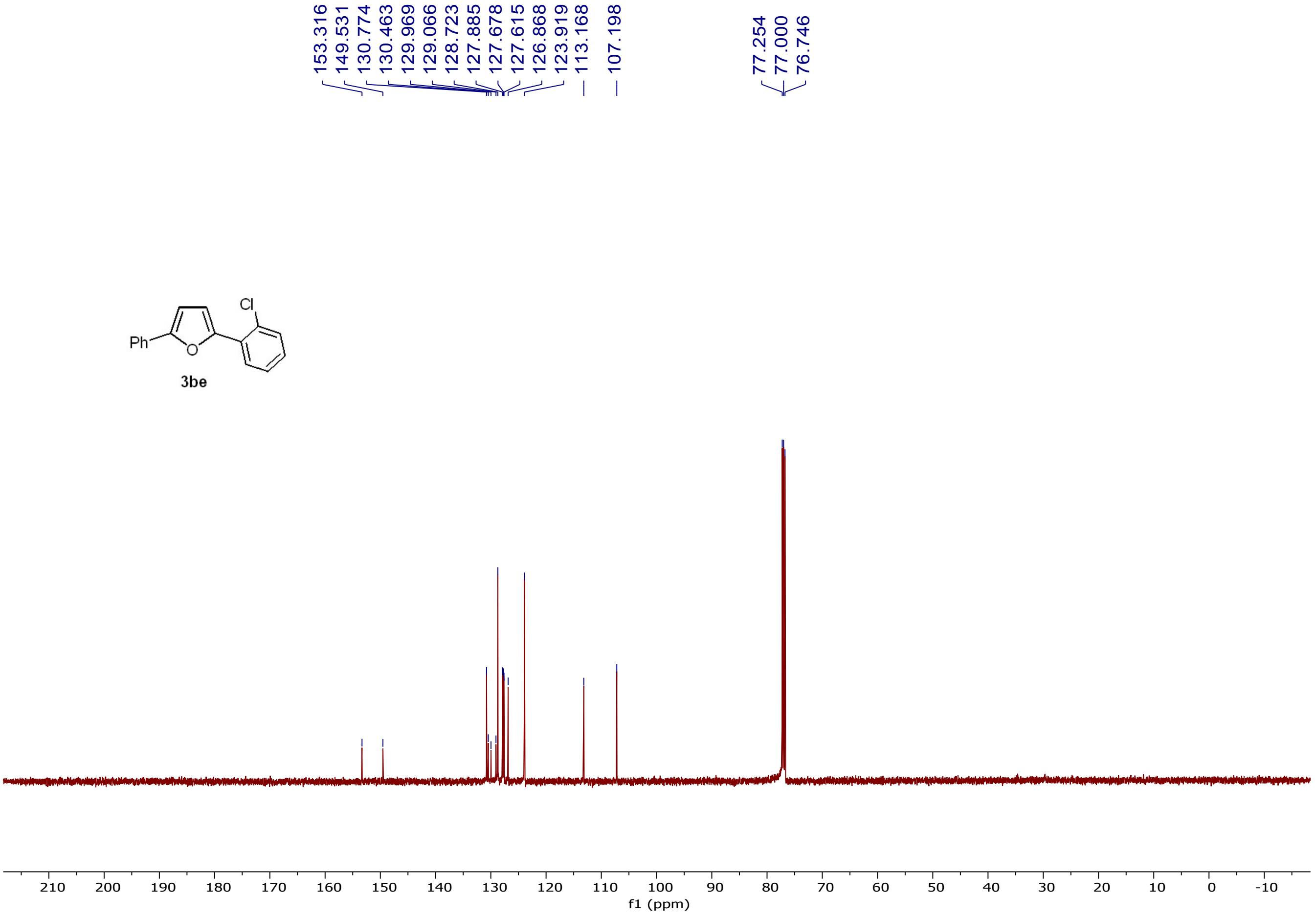
Ph
Cl
O
3be
f1 (ppm)

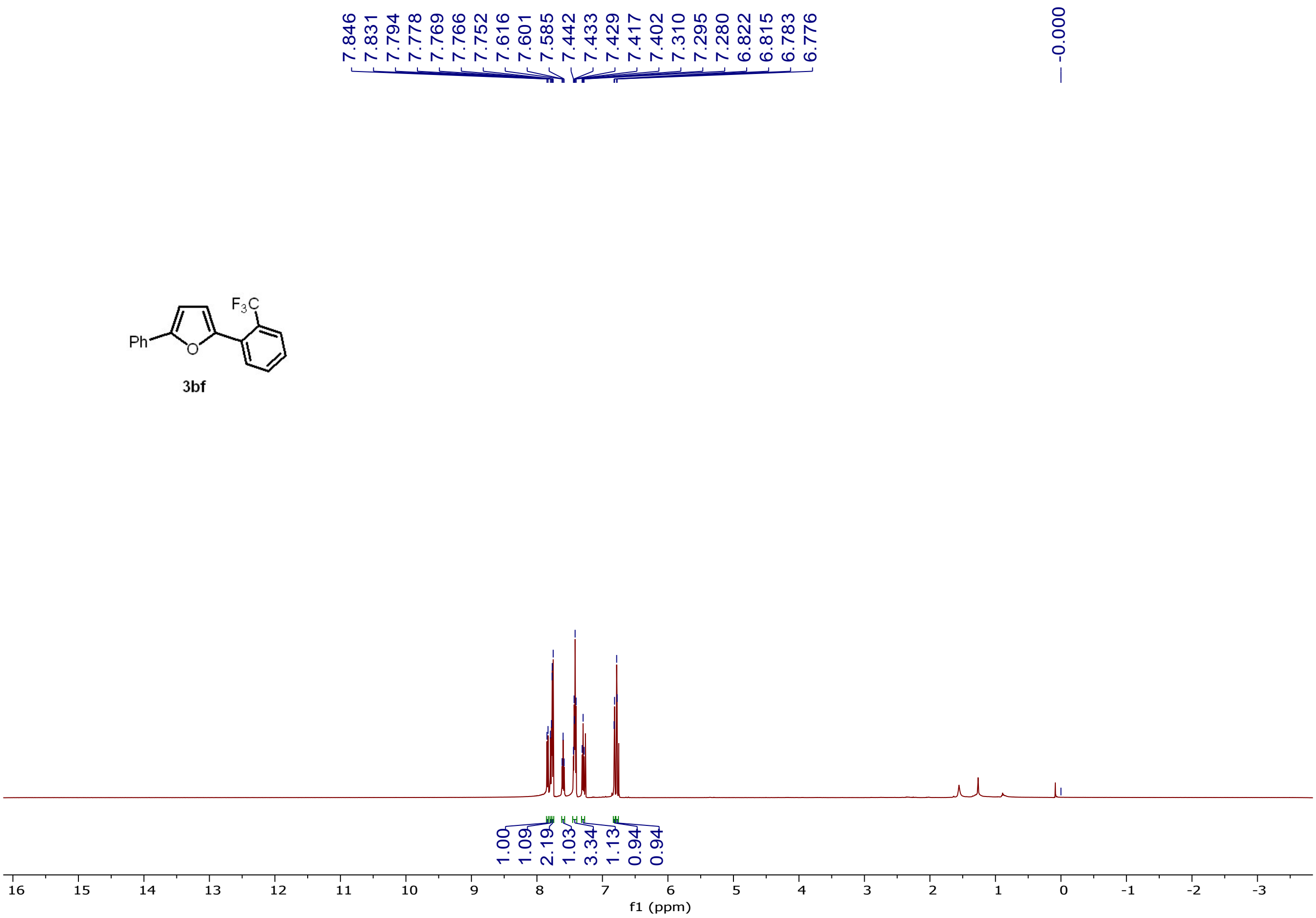
7.846
7.831
7.794
7.778
7.769
7.766
7.752
7.616
7.601
7.585
7.442
7.433
7.429
7.417
7.402
7.310
7.295
7.280
6.822
6.815
6.783
6.776
-0.000
1.00
1.09
2.19
1.03
3.34
1.13
0.94
0.94
f1 (ppm)
F3C
Ph
O

3bf

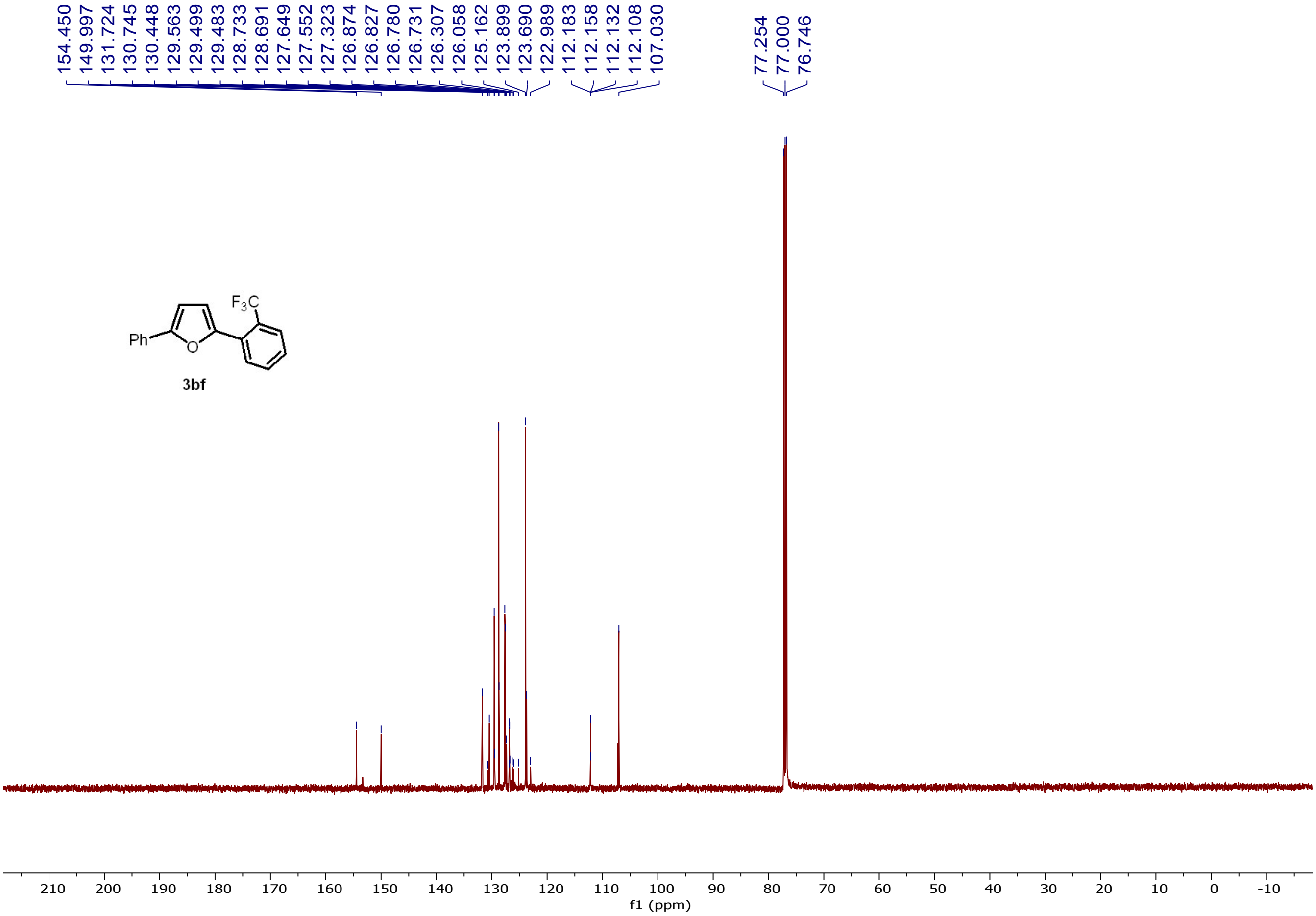
154.450
149.997
131.724
130.745
130.448
129.563
129.499
129.483
128.733
128.691
127.649
127.552
127.323
126.874
126.827
126.780
126.731
126.307
126.058
125.162
123.899
123.690
122.989
112.183
112.158
112.132
112.108
107.030
77.254
77.000
76.746
f1 (ppm)
F3C
Ph
O

3bf

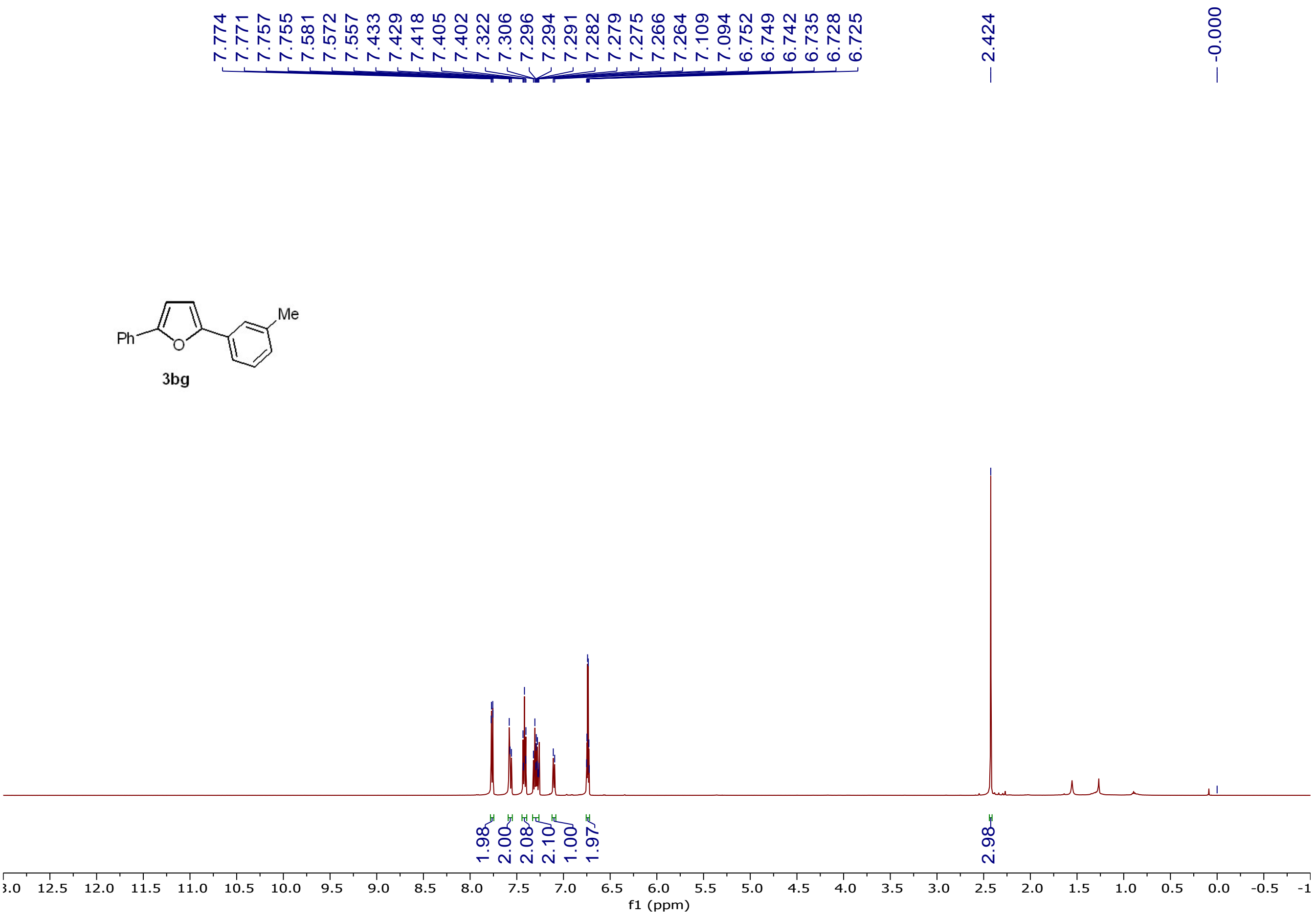
7.774 7.771 7.757 7.755 7.581 7.572 7.557 7.433 7.429 7.418 7.405 7.402 7.322 7.306 7.296 7.294 7.291 7.282 7.279 7.275 7.266 7.264 7.109 7.094 6.752 6.749 6.742 6.735 6.728 6.725
2.424
-0.000
Ph
O
Me
3bg
1.98 2.00 2.08 2.10 1.00 1.97
2.98
f1 (ppm)

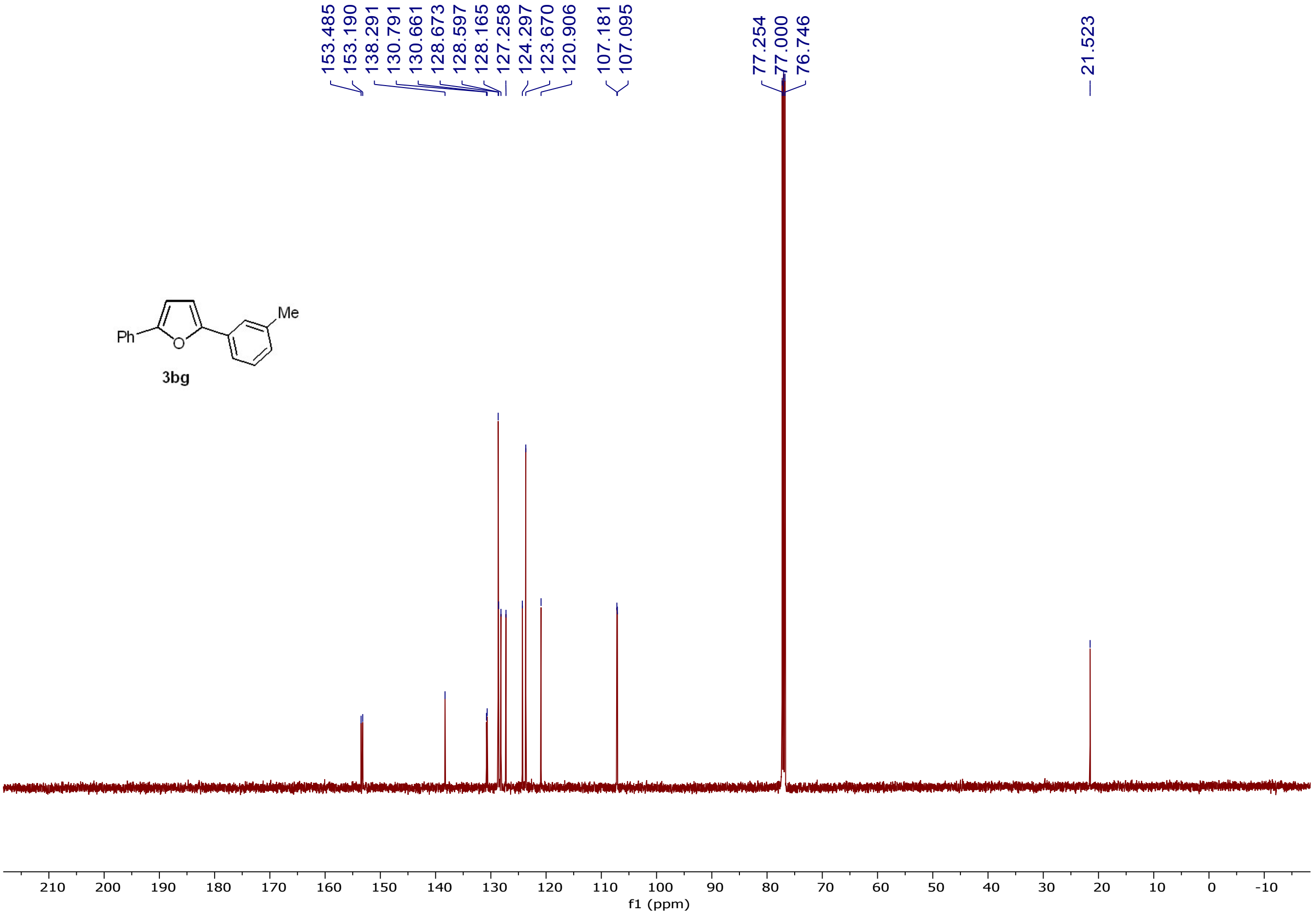
153.485 153.190 138.291 130.791 130.661 128.673 128.597 128.165 127.258 124.297 123.670 120.906 107.181 107.095
77.254 77.000 76.746
21.523
Ph
O
Me
3bg
f1 (ppm)

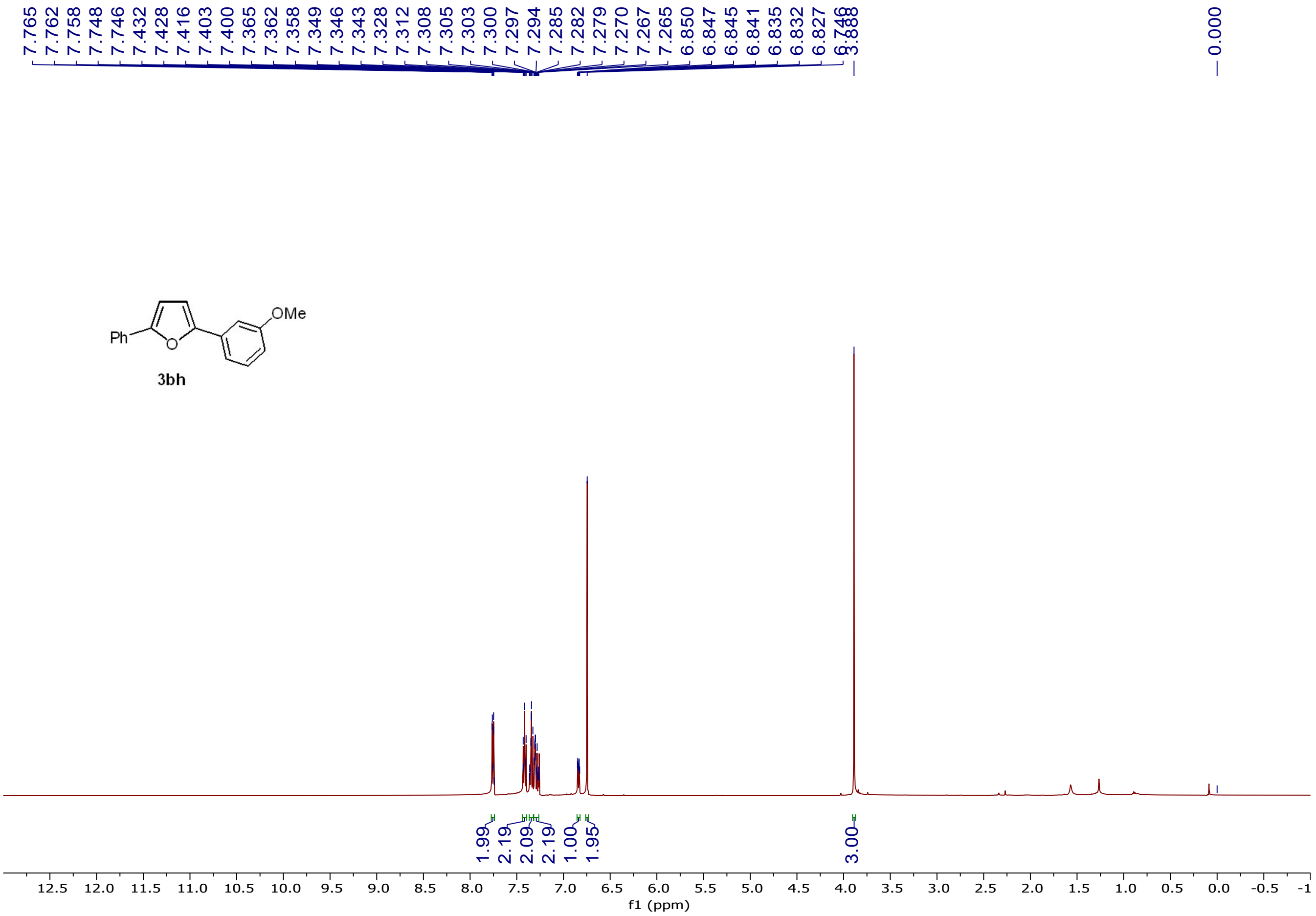
7.765 7.762 7.758 7.748 7.746 7.432 7.428 7.416 7.403 7.400 7.365 7.362 7.358 7.349 7.346 7.343 7.328 7.312 7.308 7.305 7.303 7.300 7.297 7.294 7.285 7.282 7.279 7.270 7.267 7.265 6.850 6.847 6.845 6.841 6.835 6.832 6.827 6.746 3.888
0.000
Ph
OMe
3bh
1.99 2.19 2.09 2.19 1.00 1.95
3.00
12.5 12.0 11.5 11.0 10.5 10.0 9.5 9.0 8.5 8.0 7.5 7.0 6.5 6.0 5.5 5.0 4.5 4.0 3.5 3.0 2.5 2.0 1.5 1.0 0.5 0.0 -0.5 -1
f1 (ppm)

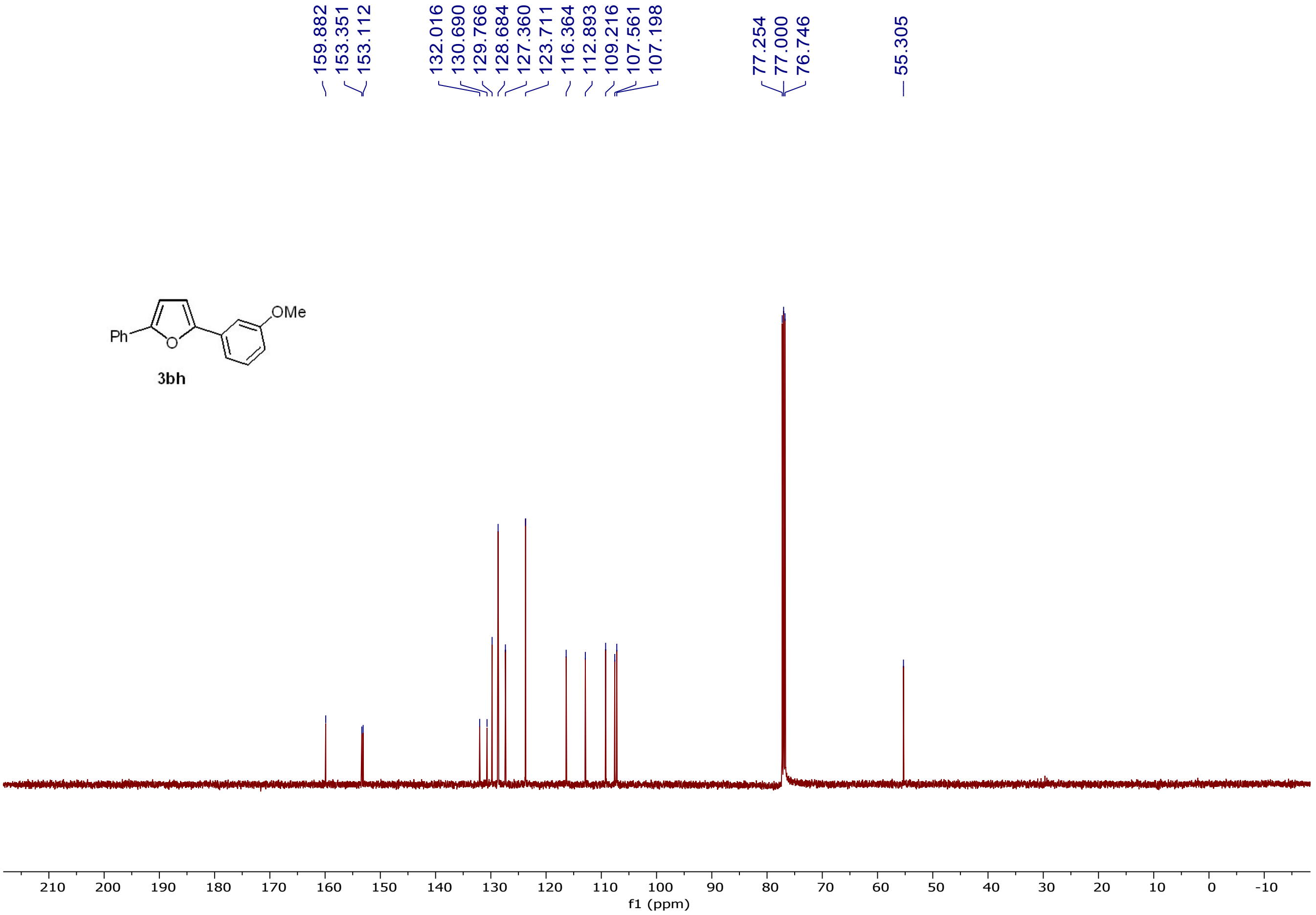
159.882 153.351 153.112
132.016 130.690 129.766 128.684 127.360 123.711 116.364 112.893 109.216 107.561 107.198
77.254 77.000 76.746
55.305
Ph
OMe
3bh
210 200 190 180 170 160 150 140 130 120 110 100 90 80 70 60 50 40 30 20 10 0 -10
f1 (ppm)

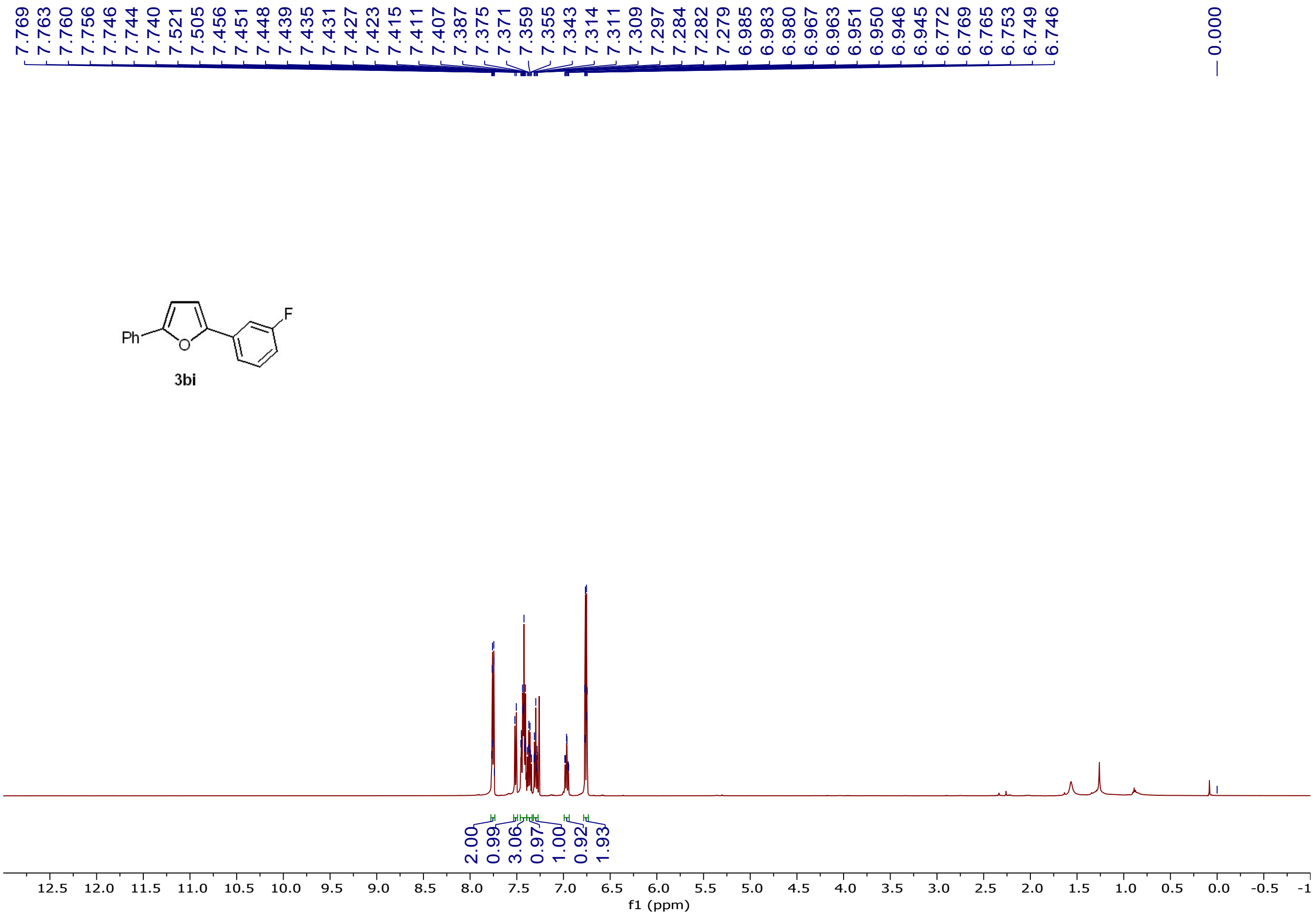
7.769 7.763 7.760 7.756 7.746 7.744 7.740 7.521 7.505 7.456 7.451 7.448 7.439 7.435 7.431 7.427 7.423 7.415 7.411 7.407 7.387 7.375 7.371 7.359 7.355 7.343 7.314 7.311 7.309 7.297 7.284 7.282 7.279 6.985 6.983 6.980 6.967 6.963 6.951 6.950 6.946 6.945 6.772 6.769 6.765 6.753 6.749 6.746
0.000
Ph
O
F
3bi
2.00 0.99 3.06 0.97 1.00 0.92 1.93
f1 (ppm)

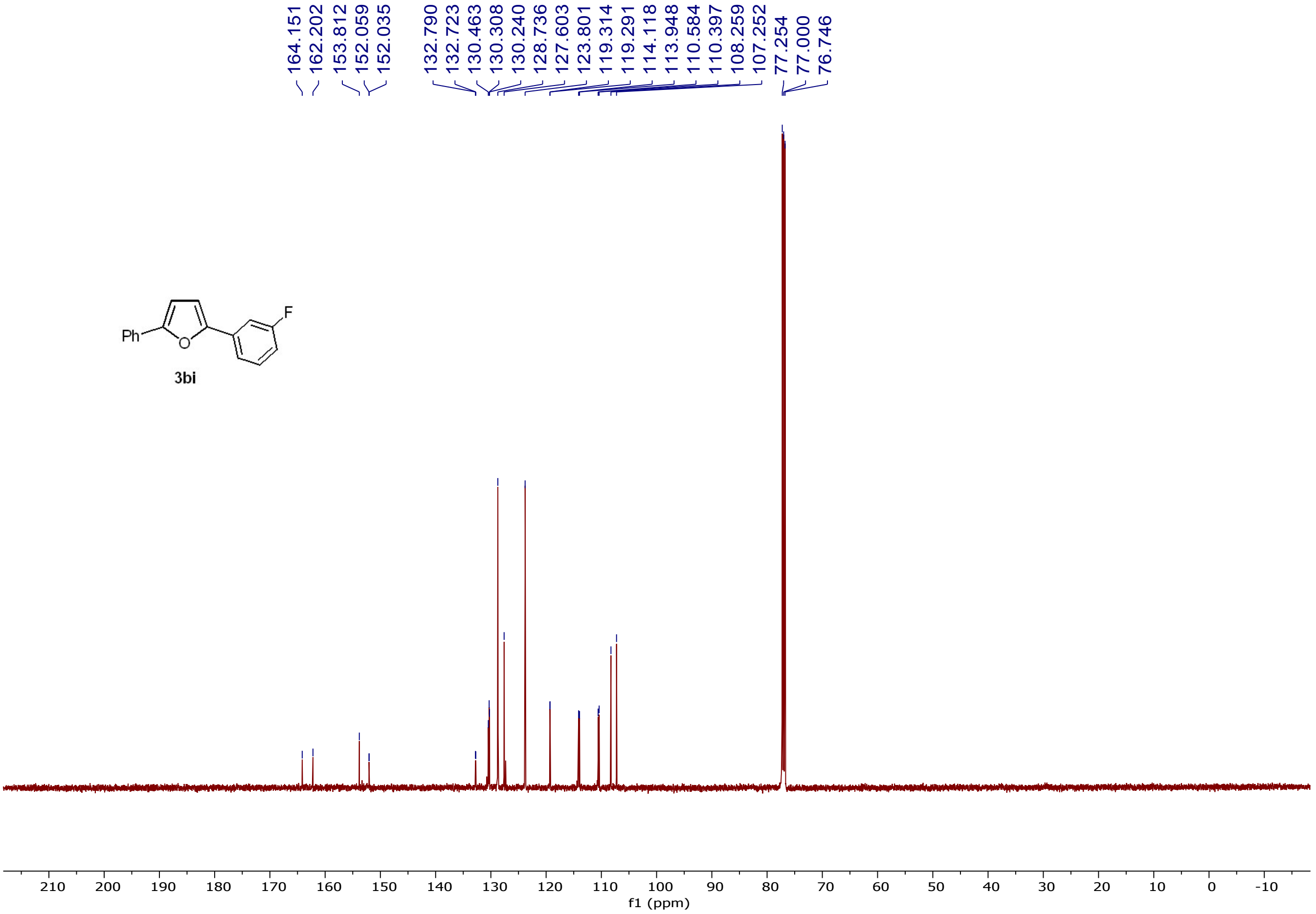
164.151 162.202 153.812 152.059 152.035
132.790 132.723 130.463 130.308 130.240 128.736 127.603 123.801 119.314 119.291 114.118 113.948 110.584 110.397 108.259 107.252 77.254 77.000 76.746
Ph
O
F
3bi
f1 (ppm)

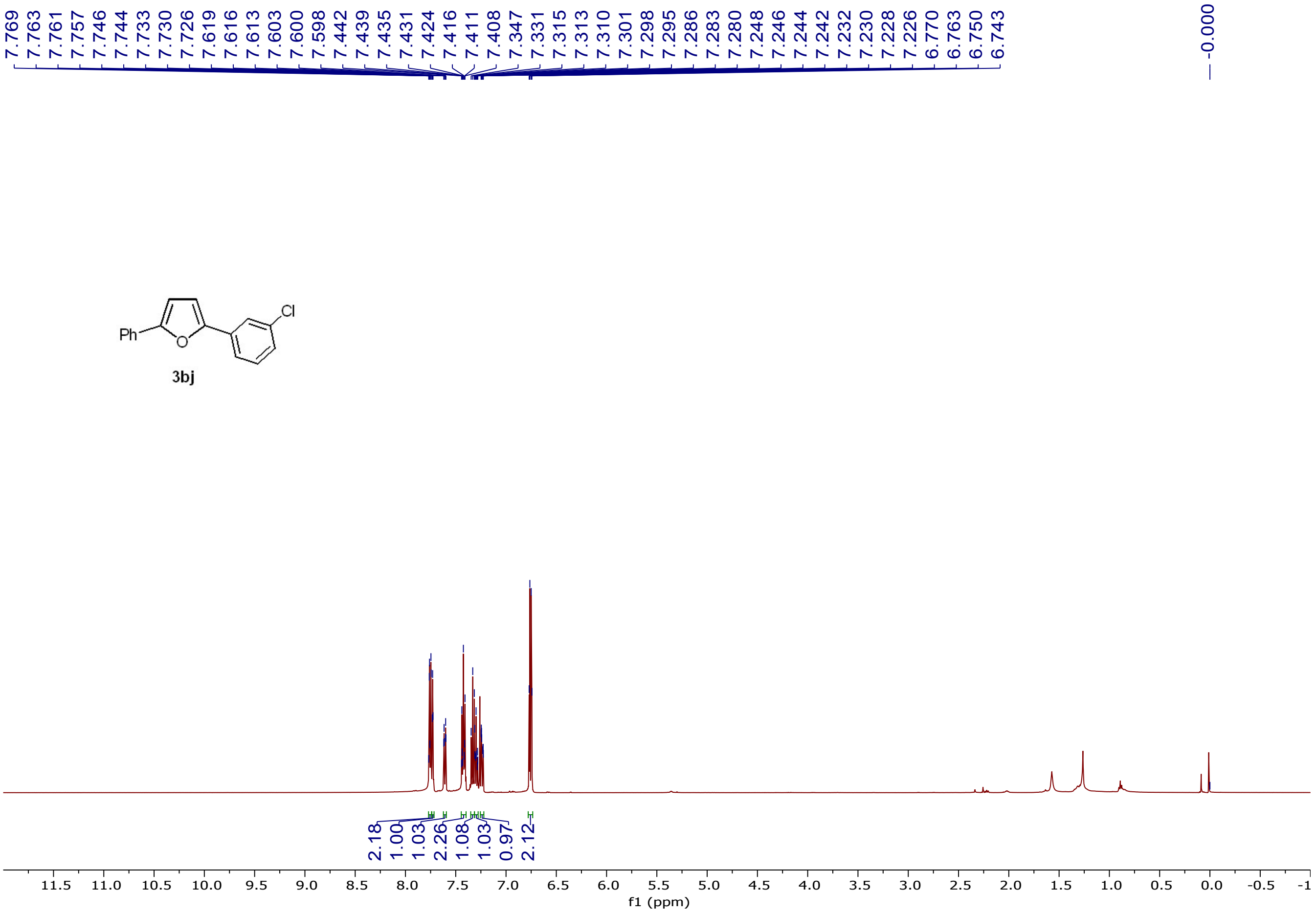
7.769
7.763
7.761
7.757
7.746
7.744
7.733
7.730
7.726
7.619
7.616
7.613
7.603
7.600
7.598
7.442
7.439
7.435
7.431
7.424
7.416
7.411
7.408
7.347
7.331
7.315
7.313
7.310
7.301
7.298
7.295
7.286
7.283
7.280
7.248
7.246
7.244
7.242
7.232
7.230
7.228
7.226
6.770
6.763
6.750
6.743
-0.000
Ph
O
Cl
3bj
2.18
1.00
1.03
2.26
1.08
1.03
0.97
2.12
f1 (ppm)

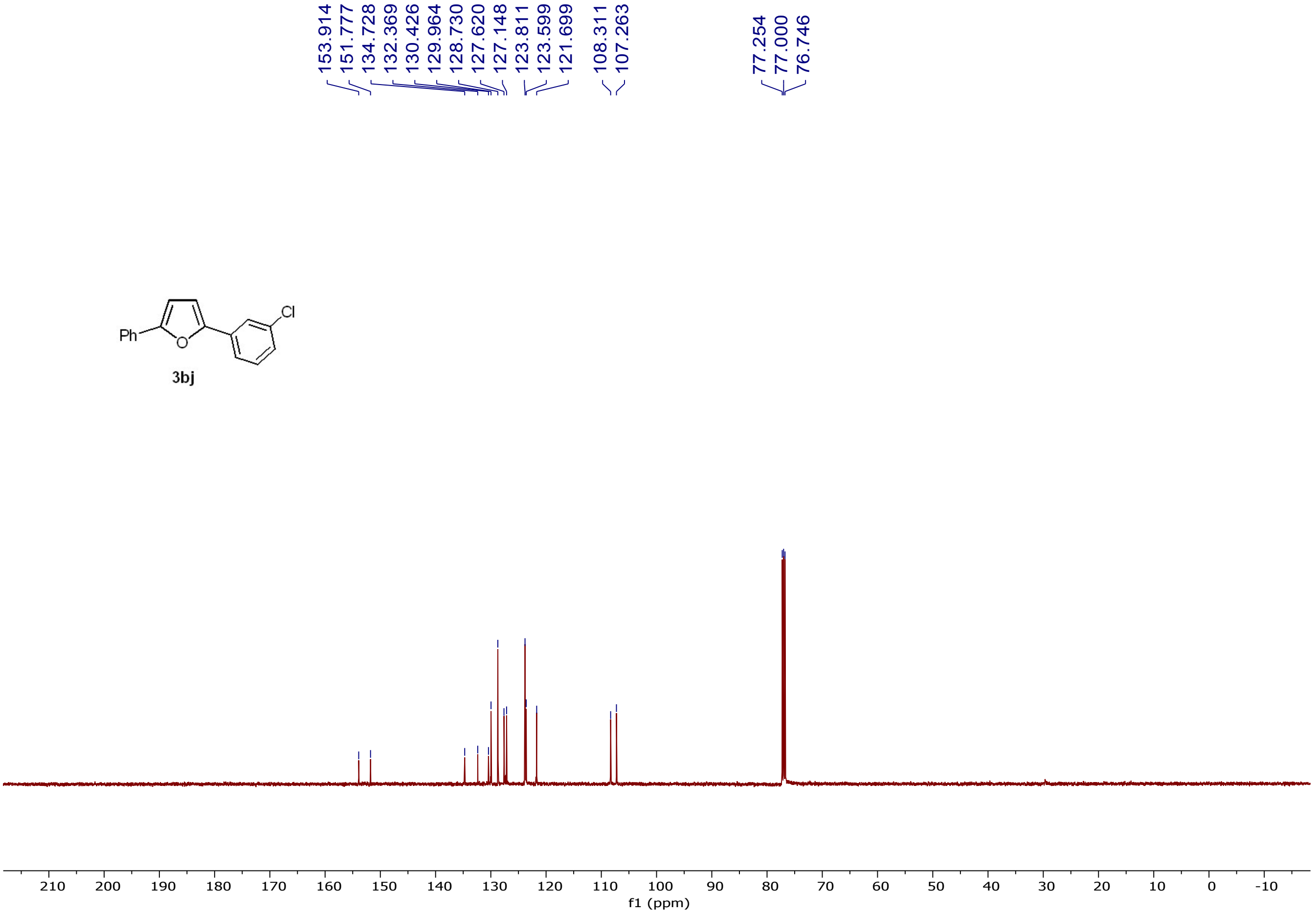
153.914
151.777
134.728
132.369
130.426
129.964
128.730
127.620
127.148
123.811
123.599
121.699
108.311
107.263
77.254
77.000
76.746
Ph
O
Cl
3bj
f1 (ppm)

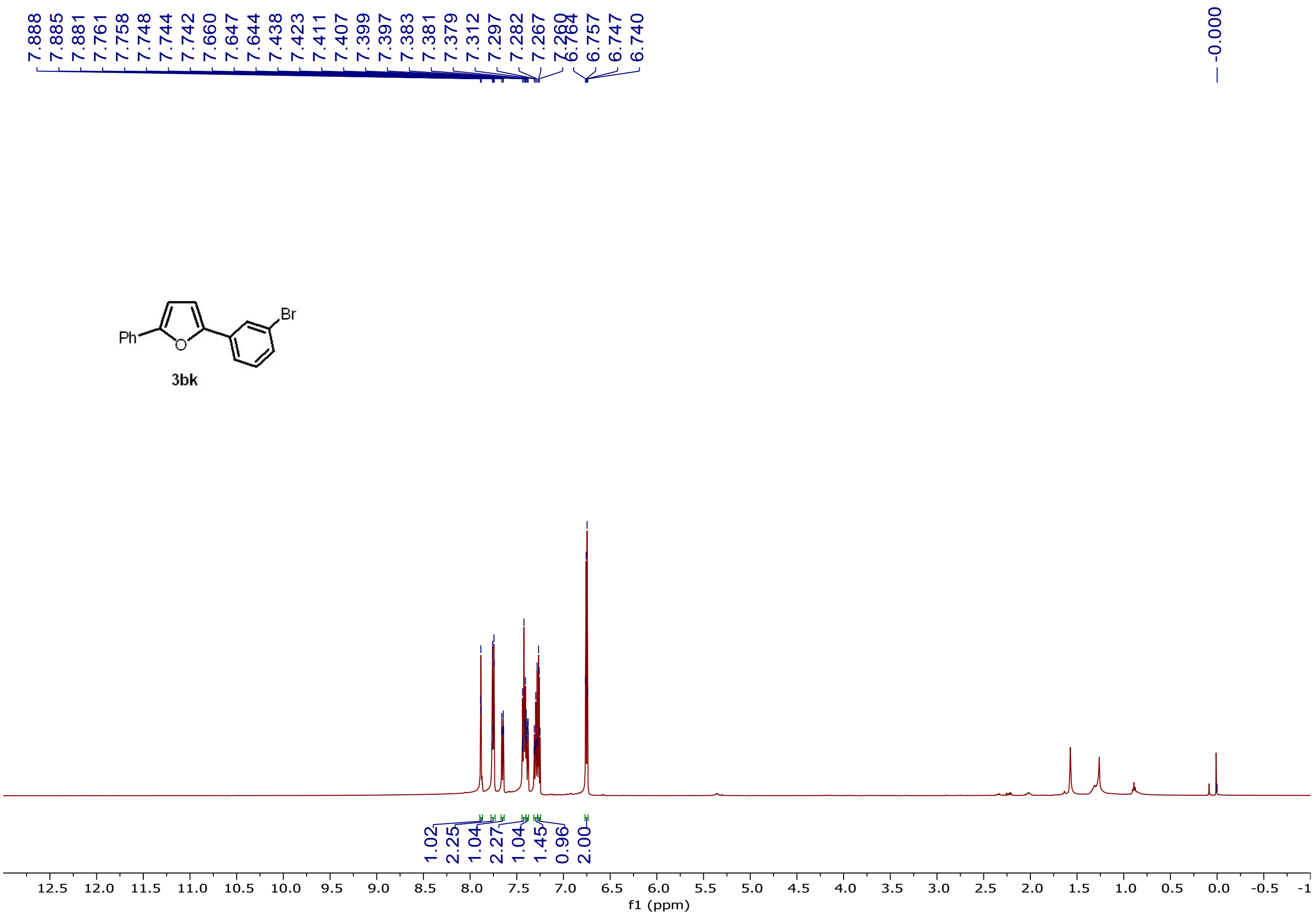
7.888 7.885 7.881 7.761 7.758 7.748 7.744 7.742 7.660 7.647 7.644 7.438 7.423 7.411 7.407 7.399 7.397 7.383 7.381 7.379 7.312 7.297 7.282 7.267 7.260 6.764 6.757 6.747 6.740
-0.000
Ph
O
Br
3bk
1.02 2.25 1.04 2.27 1.04 1.45 0.96 2.00
f1 (ppm)

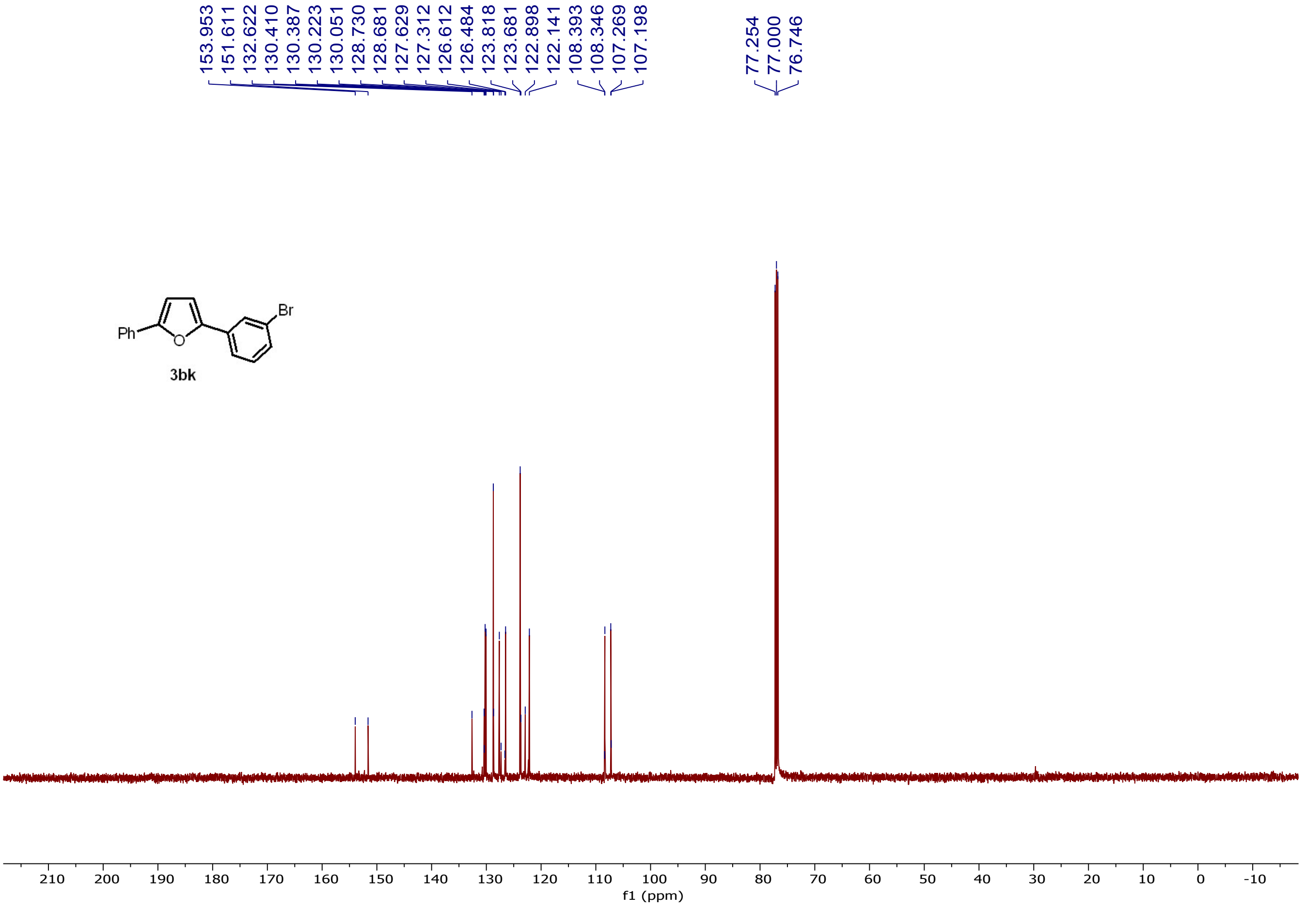
153.953 151.611 132.622 130.410 130.387 130.223 130.051 128.730 128.681 127.629 127.312 126.612 126.484 123.818 123.681 122.898 122.141 108.393 108.346 107.269 107.198
77.254 77.000 76.746
Ph
O
Br
3bk
f1 (ppm)

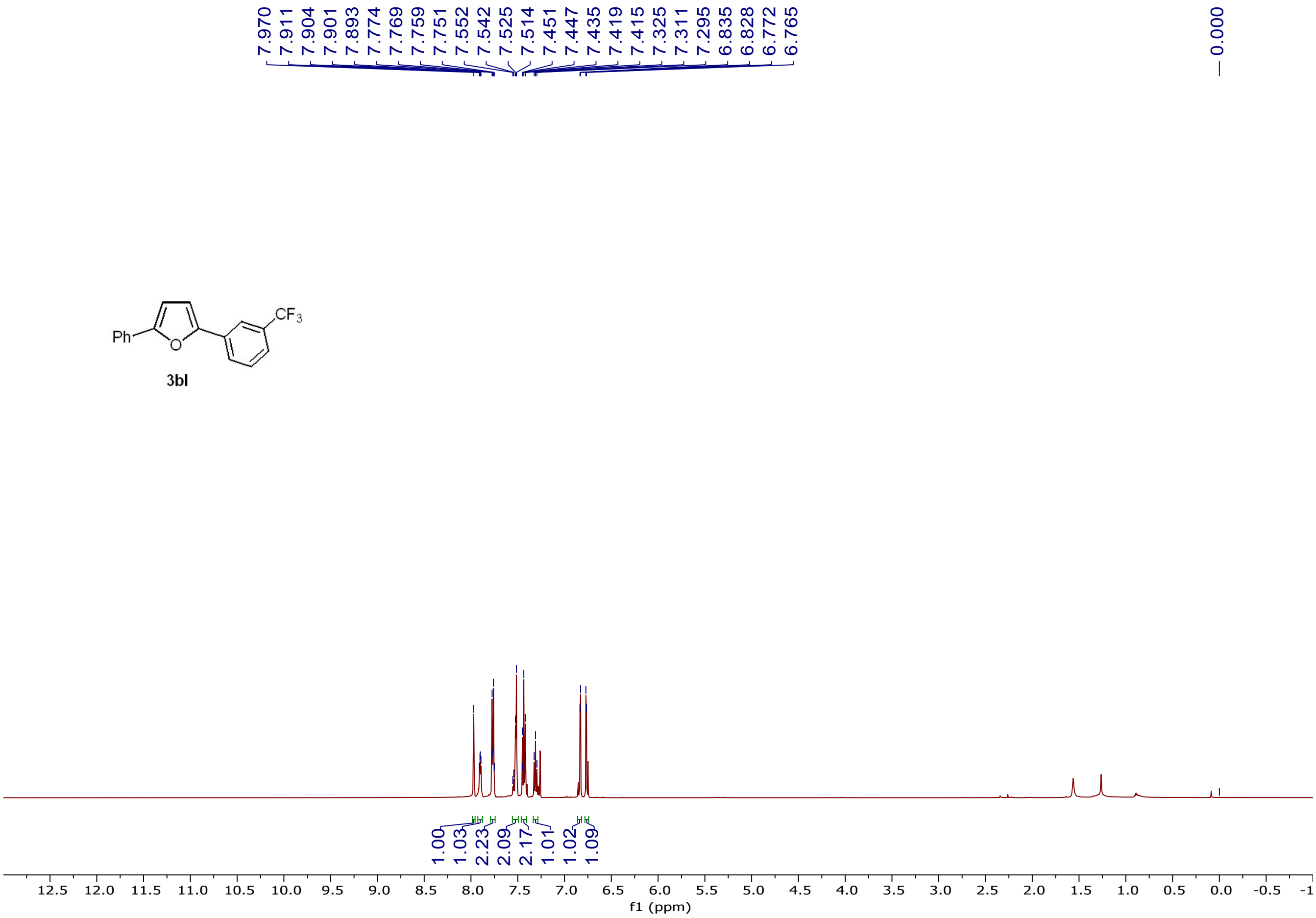
7.970
7.911
7.904
7.901
7.893
7.774
7.769
7.759
7.751
7.552
7.542
7.525
7.514
7.451
7.447
7.435
7.419
7.415
7.325
7.311
7.295
6.835
6.828
6.772
6.765
0.000
Ph
O
CF3
3bl
1.00
1.03
2.23
2.09
2.17
1.01
1.02
1.09
f1 (ppm)

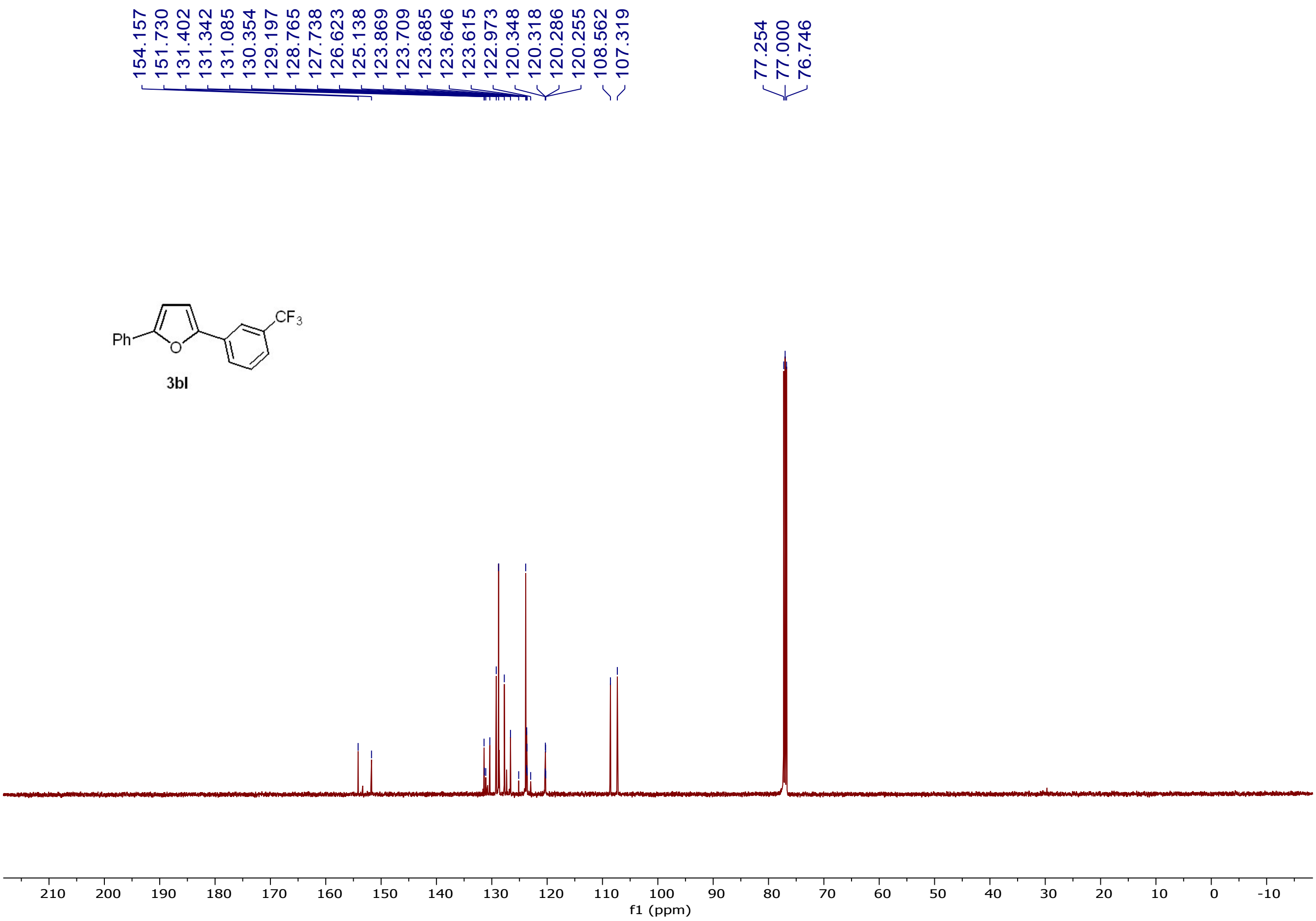
154.157
151.730
131.402
131.342
131.085
130.354
129.197
128.765
127.738
126.623
125.138
123.869
123.709
123.685
123.646
123.615
122.973
120.348
120.318
120.286
120.255
108.562
107.319
77.254
77.000
76.746
Ph
O
CF3
3bl
f1 (ppm)

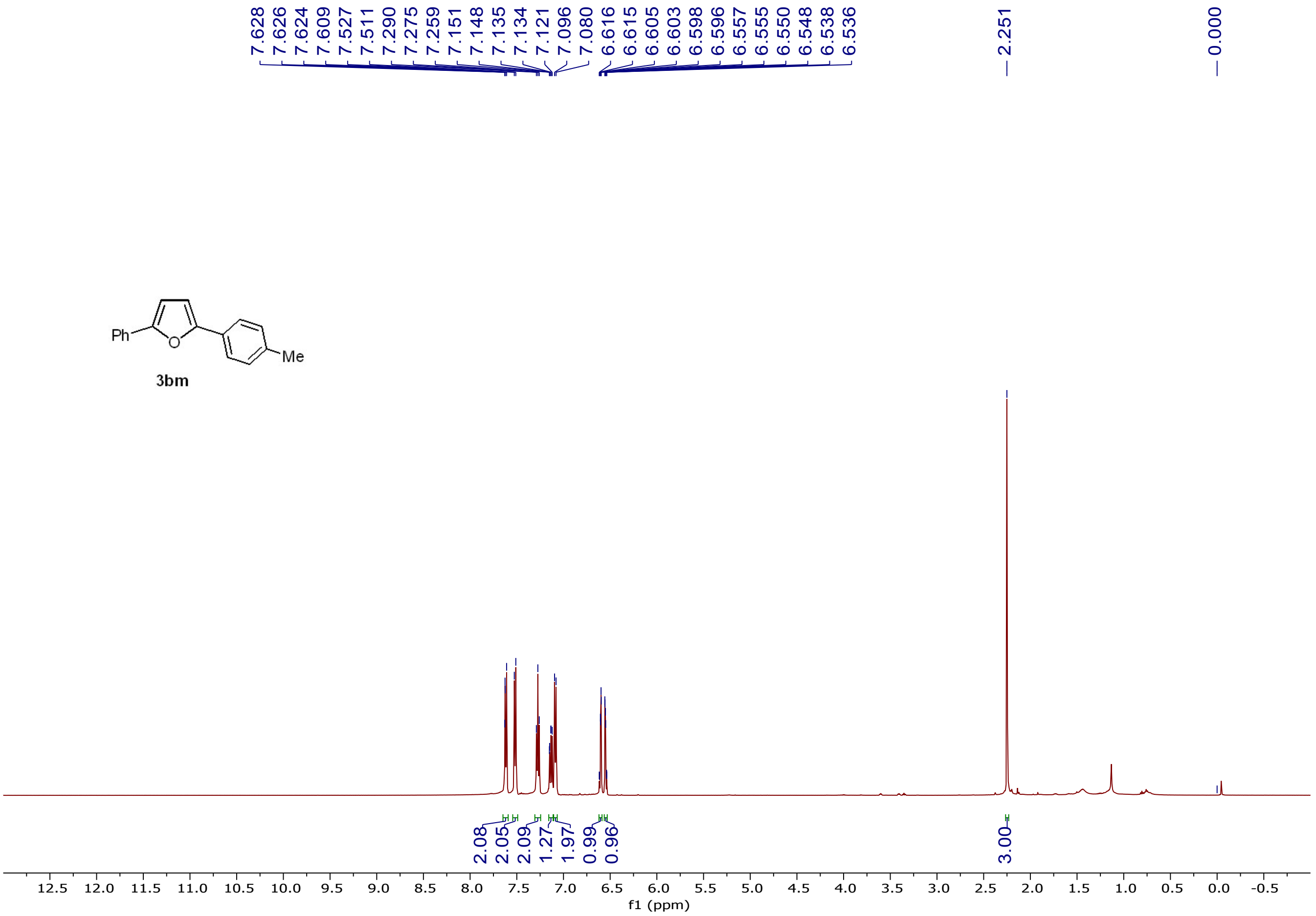
7.628
7.626
7.624
7.609
7.527
7.511
7.290
7.275
7.259
7.151
7.148
7.135
7.134
7.121
7.096
7.080
6.616
6.615
6.605
6.603
6.598
6.596
6.557
6.555
6.550
6.548
6.538
6.536
2.251
0.000
Ph
O
Me
3bm
2.08
2.05
2.09
1.27
1.97
0.99
0.96
3.00
f1 (ppm)

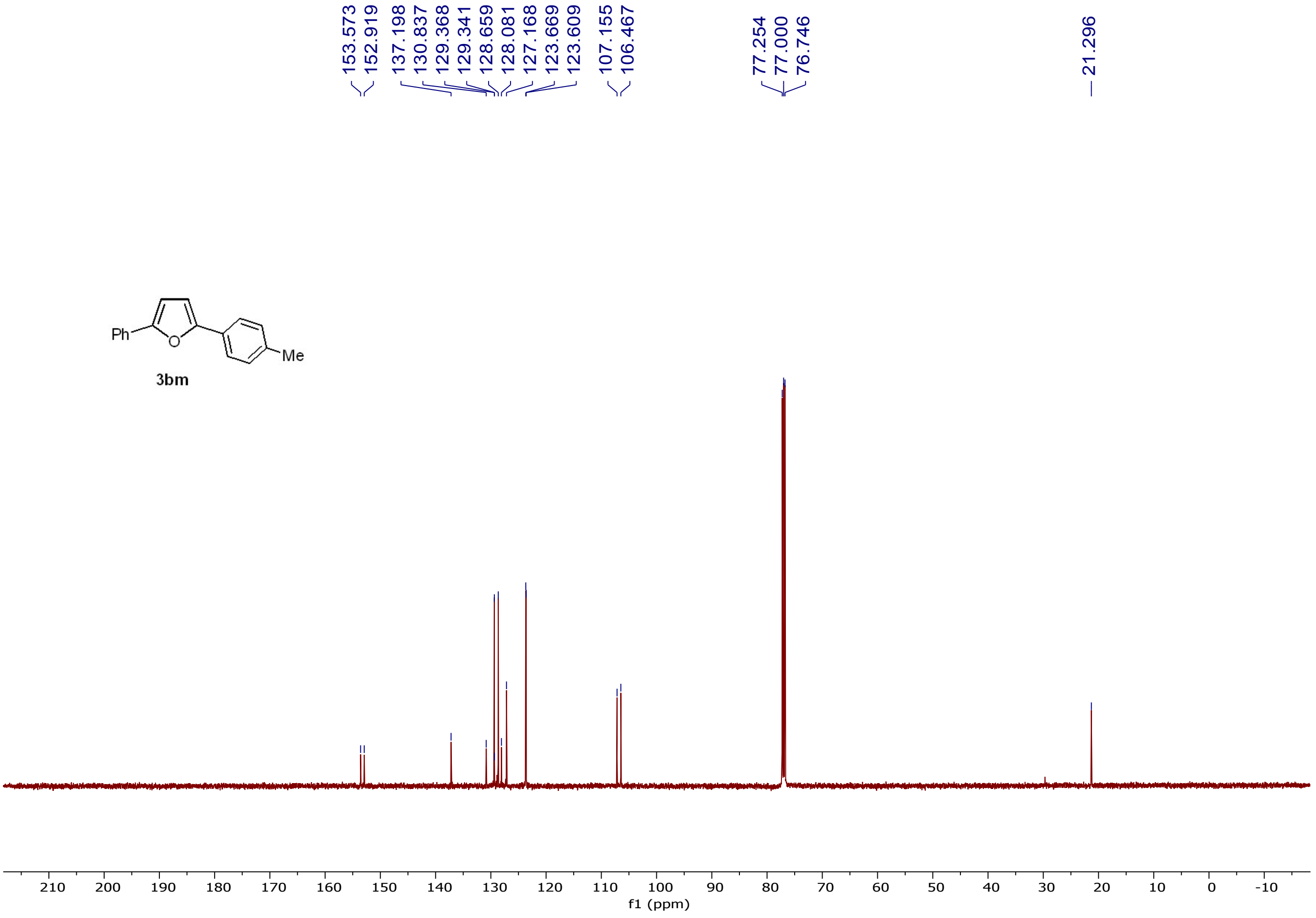
153.573
152.919
137.198
130.837
129.368
129.341
128.659
128.081
127.168
123.669
123.609
107.155
106.467
77.254
77.000
76.746
21.296
Ph
O
Me
3bm
f1 (ppm)

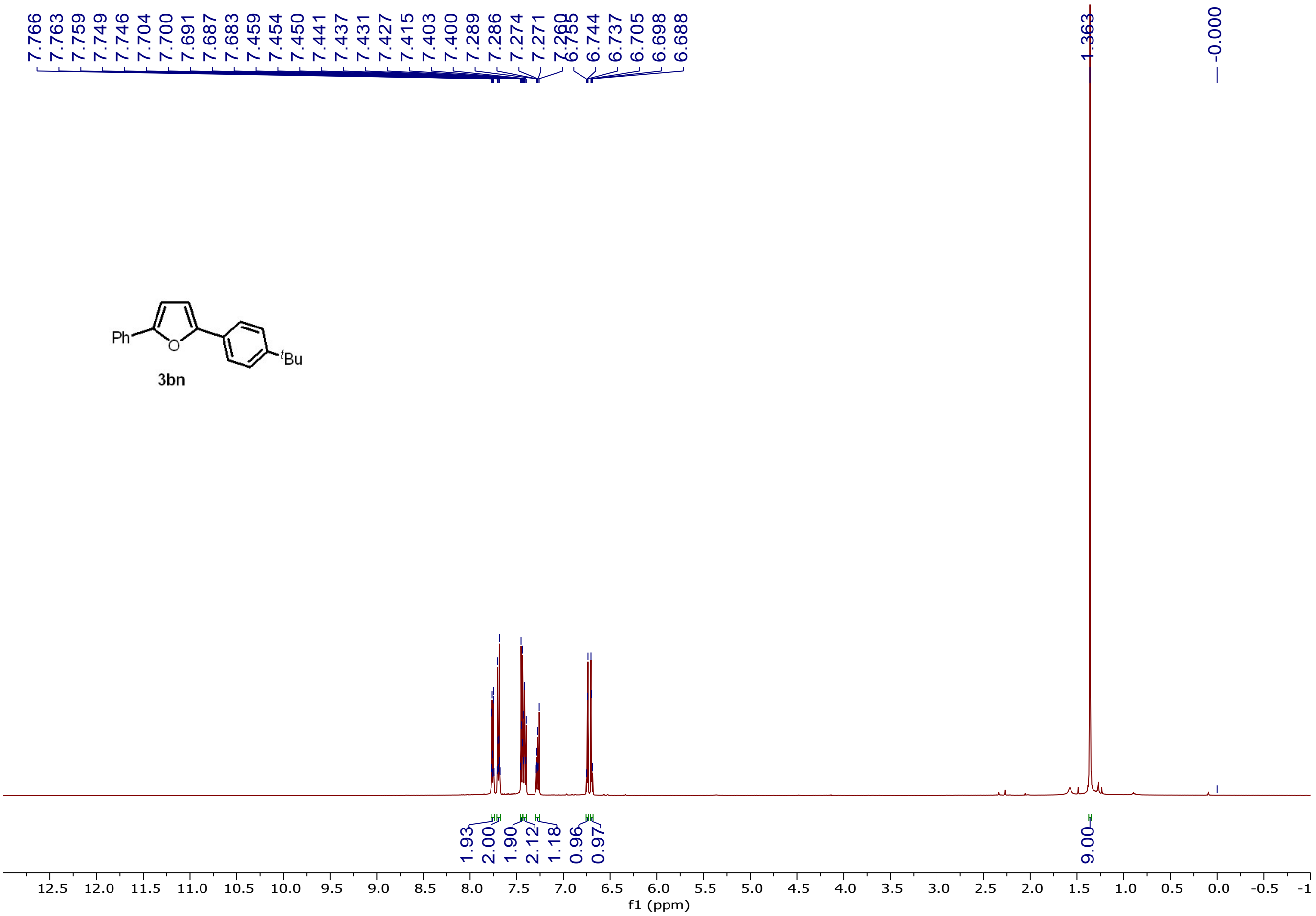
7.766
7.763
7.759
7.749
7.746
7.704
7.700
7.691
7.687
7.683
7.459
7.454
7.450
7.441
7.437
7.431
7.427
7.415
7.403
7.400
7.289
7.286
7.274
7.271
7.260
6.755
6.744
6.737
6.705
6.698
6.688
1.363
-0.000
Ph
O
tBu
3bn
1.93
2.00
1.90
2.12
1.18
0.96
0.97
9.00
12.5 12.0 11.5 11.0 10.5 10.0 9.5 9.0 8.5 8.0 7.5 7.0 6.5 6.0 5.5 5.0 4.5 4.0 3.5 3.0 2.5 2.0 1.5 1.0 0.5 0.0 -0.5 -1
f1 (ppm)

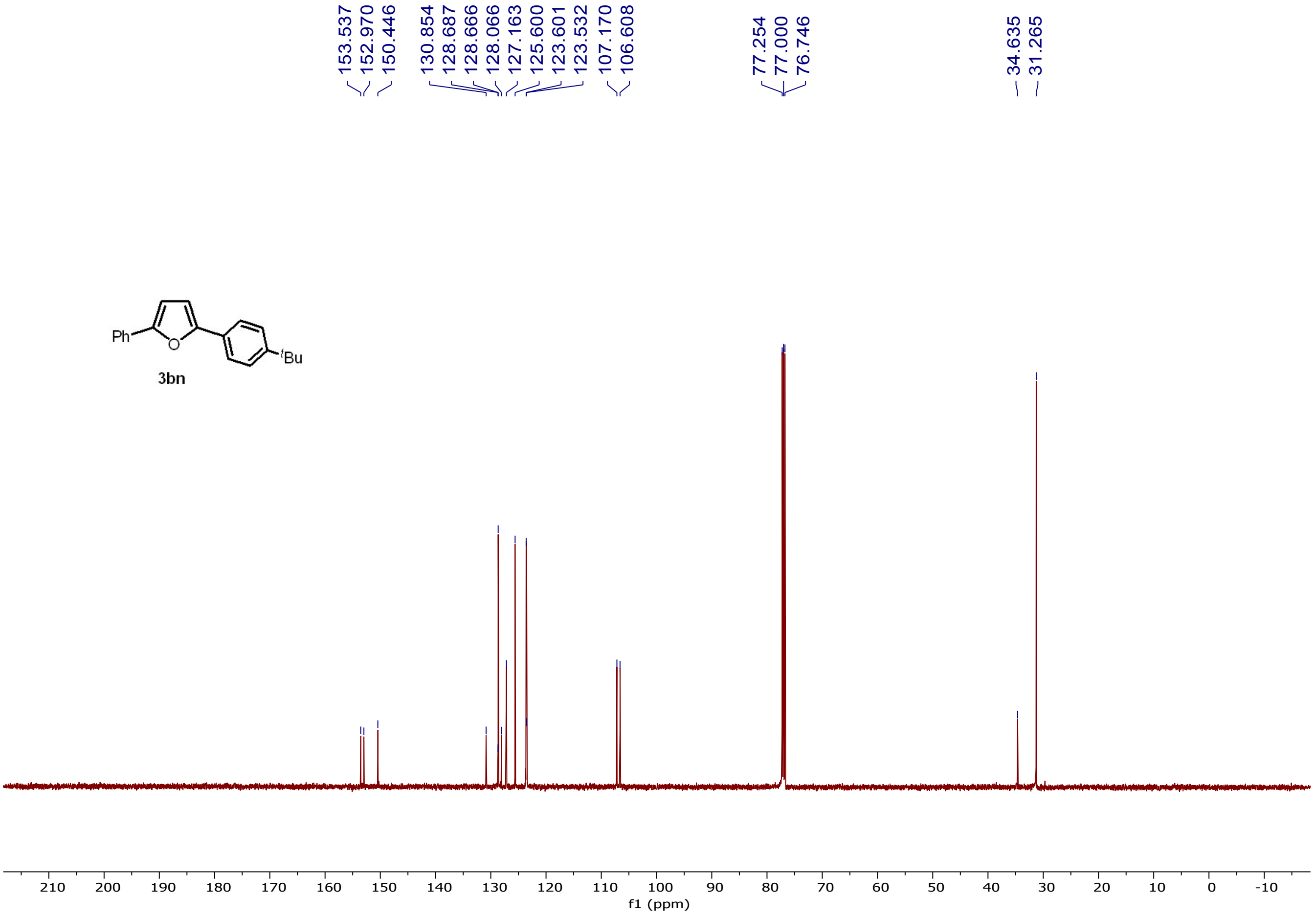
153.537
152.970
150.446
130.854
128.687
128.666
128.066
127.163
125.600
123.601
123.532
107.170
106.608
77.254
77.000
76.746
34.635
31.265
Ph
O
tBu
3bn
210 200 190 180 170 160 150 140 130 120 110 100 90 80 70 60 50 40 30 20 10 0 -10
f1 (ppm)

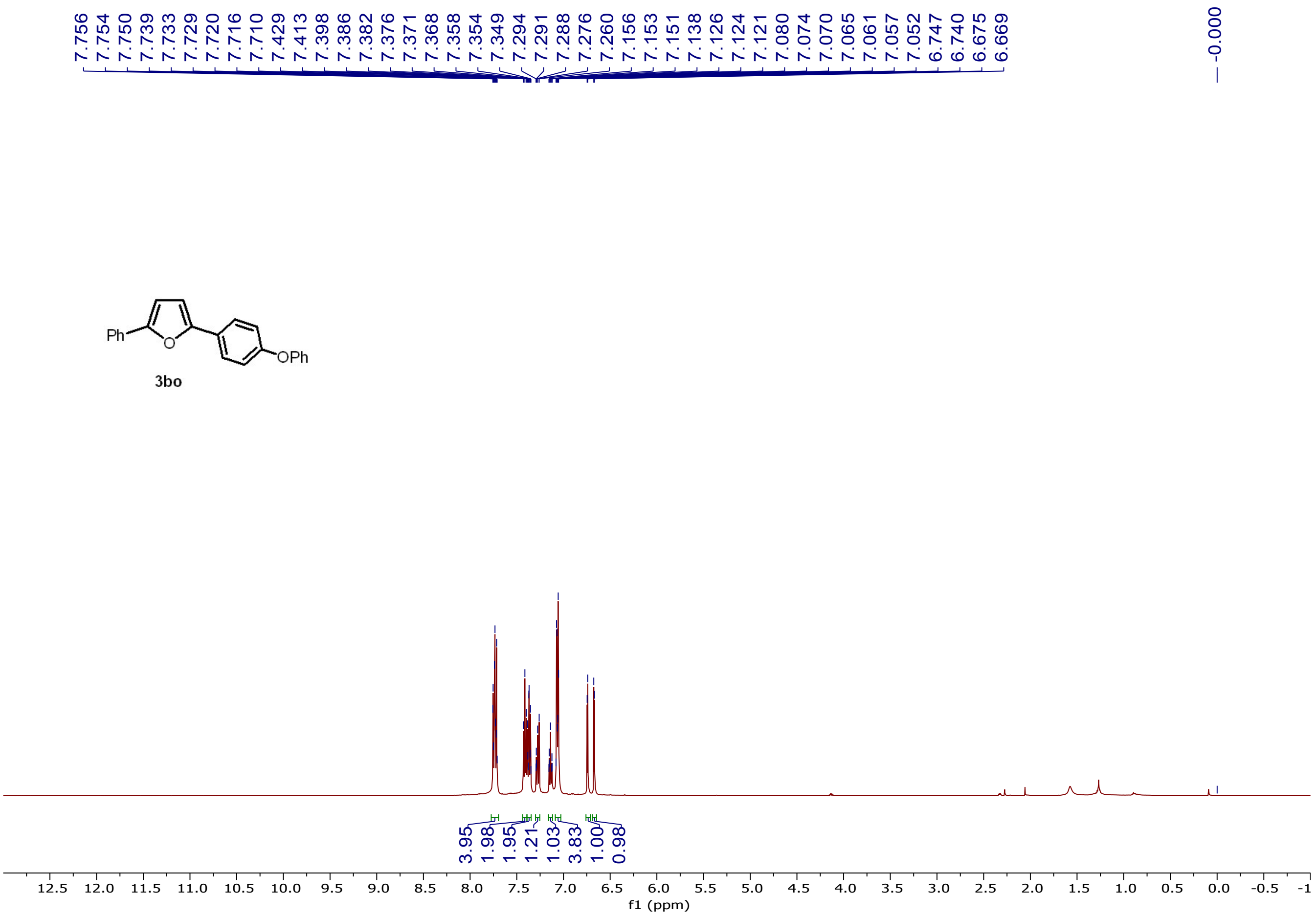
7.756 7.754 7.750 7.739 7.733 7.729 7.720 7.716 7.710 7.429 7.413 7.398 7.386 7.382 7.376 7.371 7.368 7.358 7.354 7.349 7.294 7.291 7.288 7.276 7.260 7.156 7.153 7.151 7.138 7.126 7.124 7.121 7.080 7.074 7.070 7.065 7.061 7.057 7.052 6.747 6.740 6.675 6.669
-0.000
Ph
O
OPh
3bo
3.95 1.98 1.95 1.21 1.03 3.83 1.00 0.98
f1 (ppm)

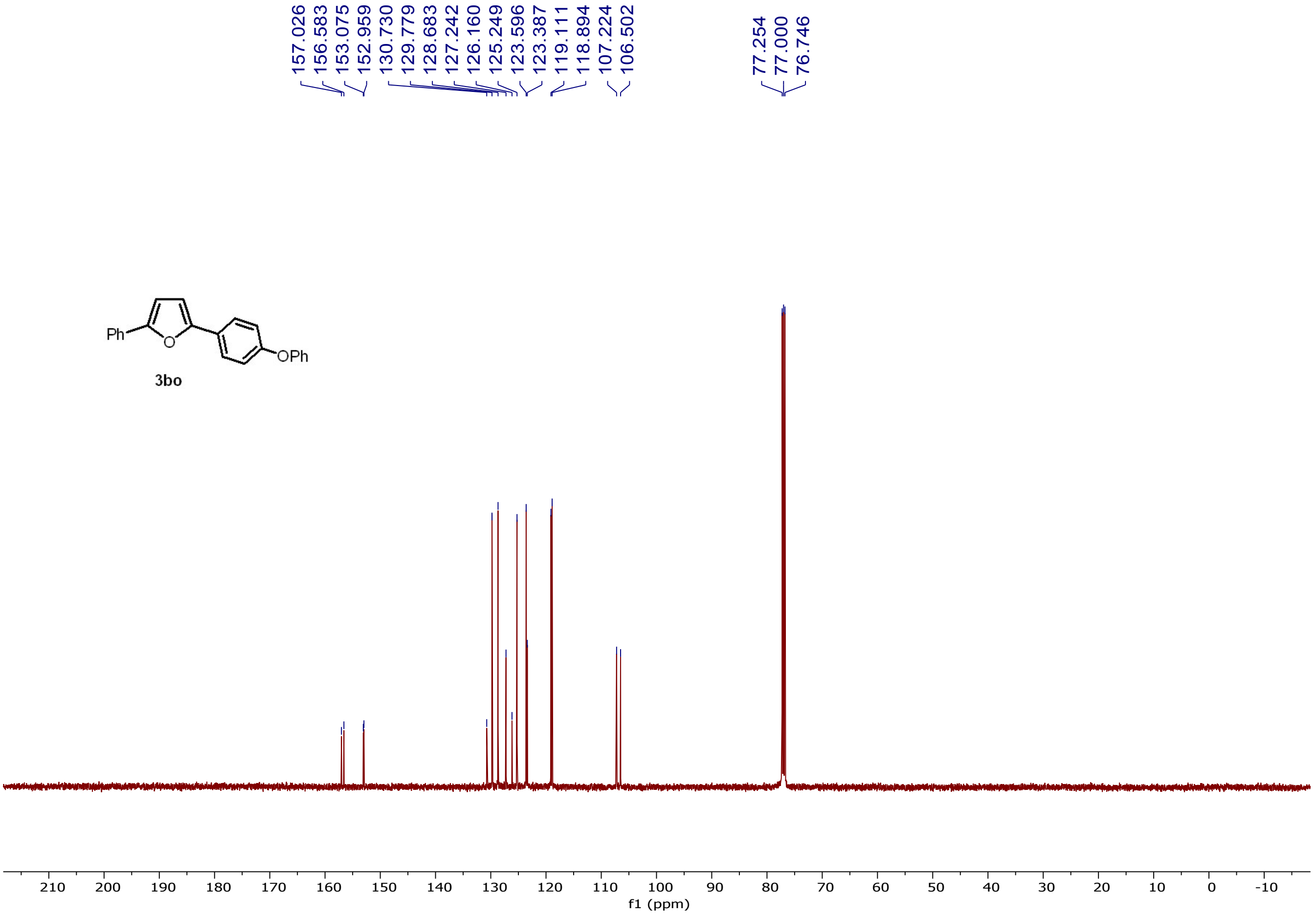
157.026 156.583 153.075 152.959 130.730 129.779 128.683 127.242 126.160 125.249 123.596 123.387 119.111 118.894 107.224 106.502
77.254 77.000 76.746
Ph
O
OPh
3bo
f1 (ppm)

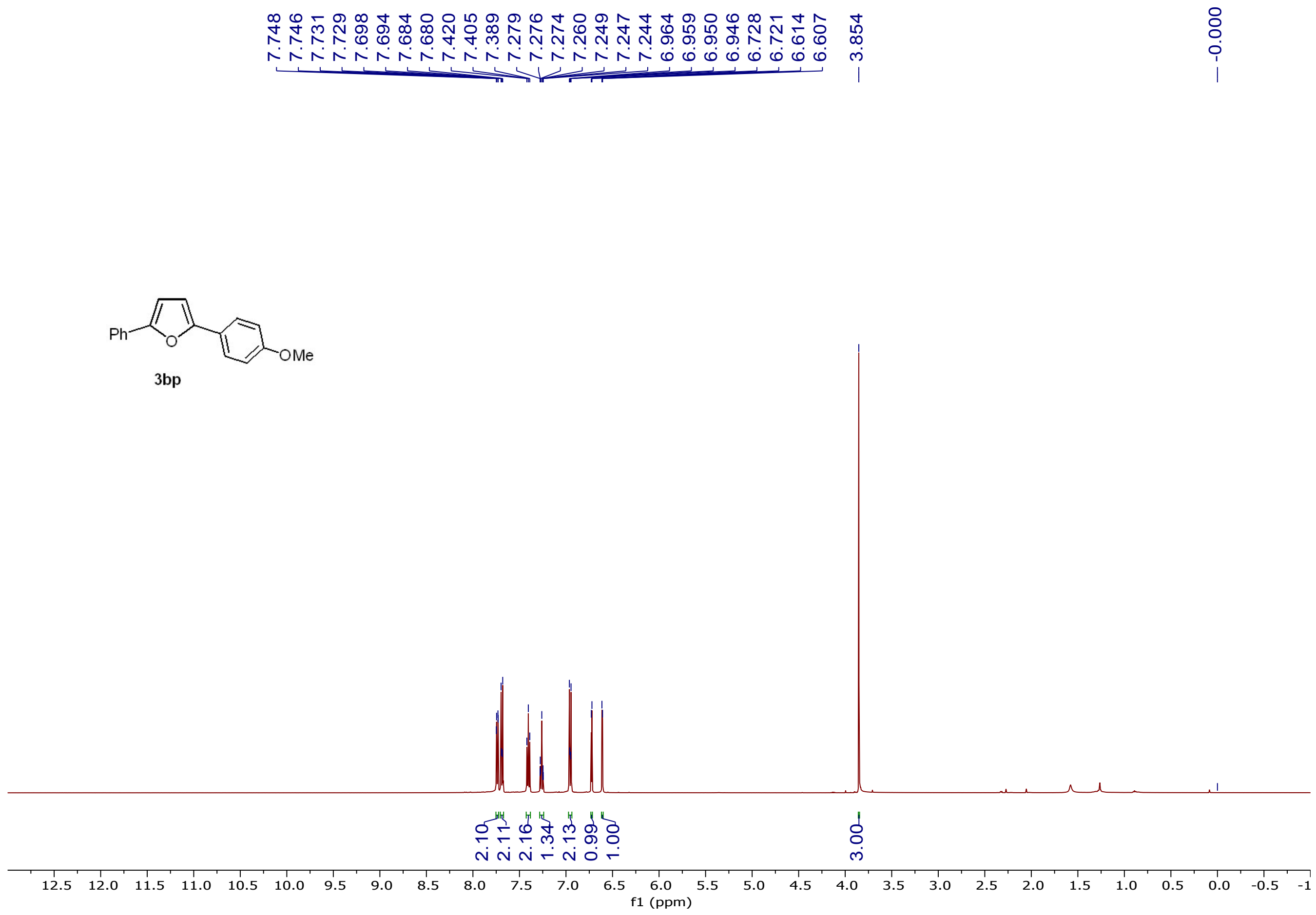

7.748
7.746
7.731
7.729
7.698
7.694
7.684
7.680
7.420
7.405
7.389
7.279
7.276
7.274
7.260
7.249
7.247
7.244
6.964
6.959
6.950
6.946
6.728
6.721
6.614
6.607
3.854
-0.000
Ph
O
OMe
3bp
2.10
2.11
2.16
1.34
2.13
0.99
1.00
3.00
12.5 12.0 11.5 11.0 10.5 10.0 9.5 9.0 8.5 8.0 7.5 7.0 6.5 6.0 5.5 5.0 4.5 4.0 3.5 3.0 2.5 2.0 1.5 1.0 0.5 0.0 -0.5 -1
f1 (ppm)

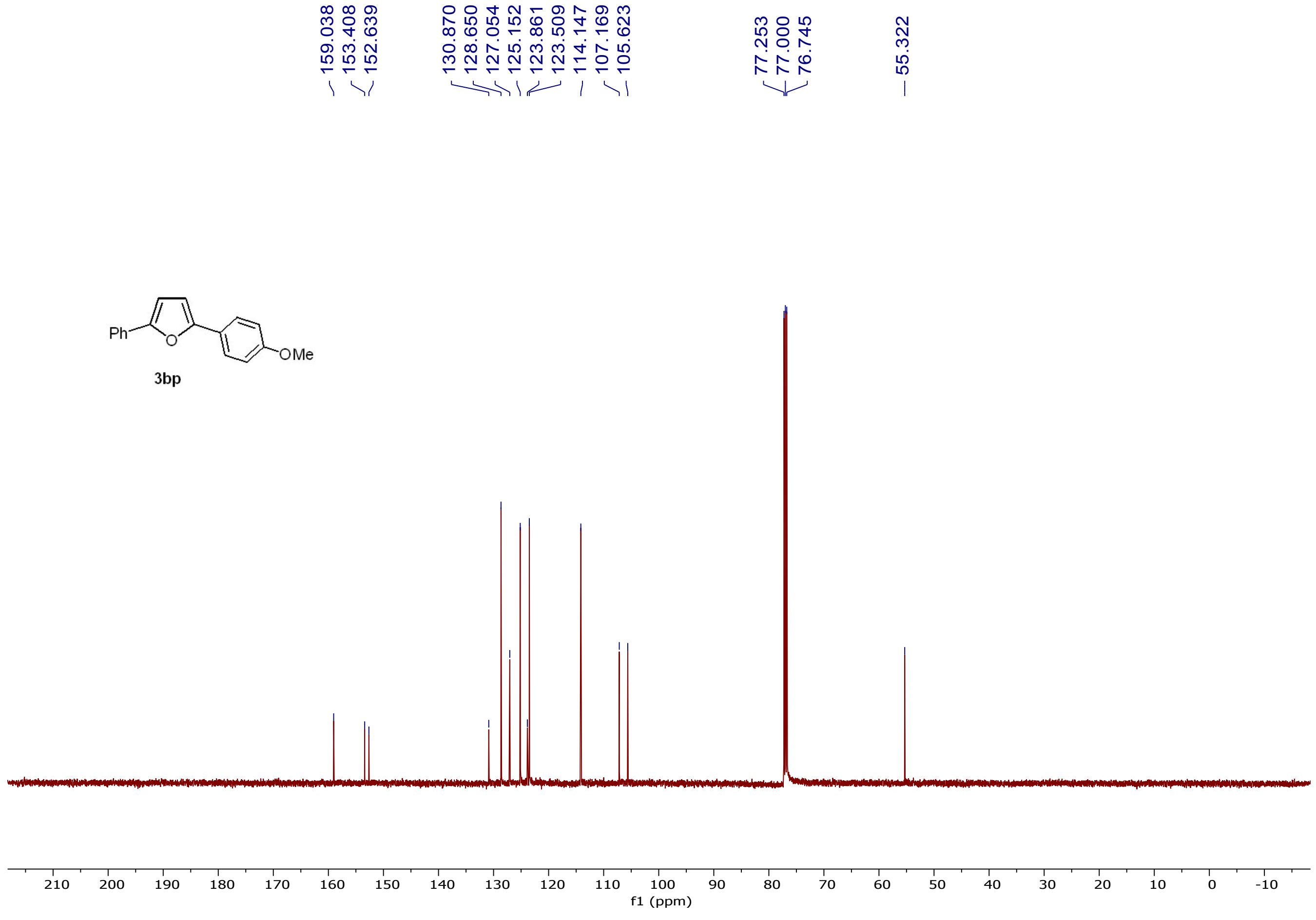

159.038
153.408
152.639
130.870
128.650
127.054
125.152
123.861
123.509
114.147
107.169
105.623
77.253
77.000
76.745
55.322
Ph
O
OMe
3bp
210 200 190 180 170 160 150 140 130 120 110 100 90 80 70 60 50 40 30 20 10 0 -10
f1 (ppm)

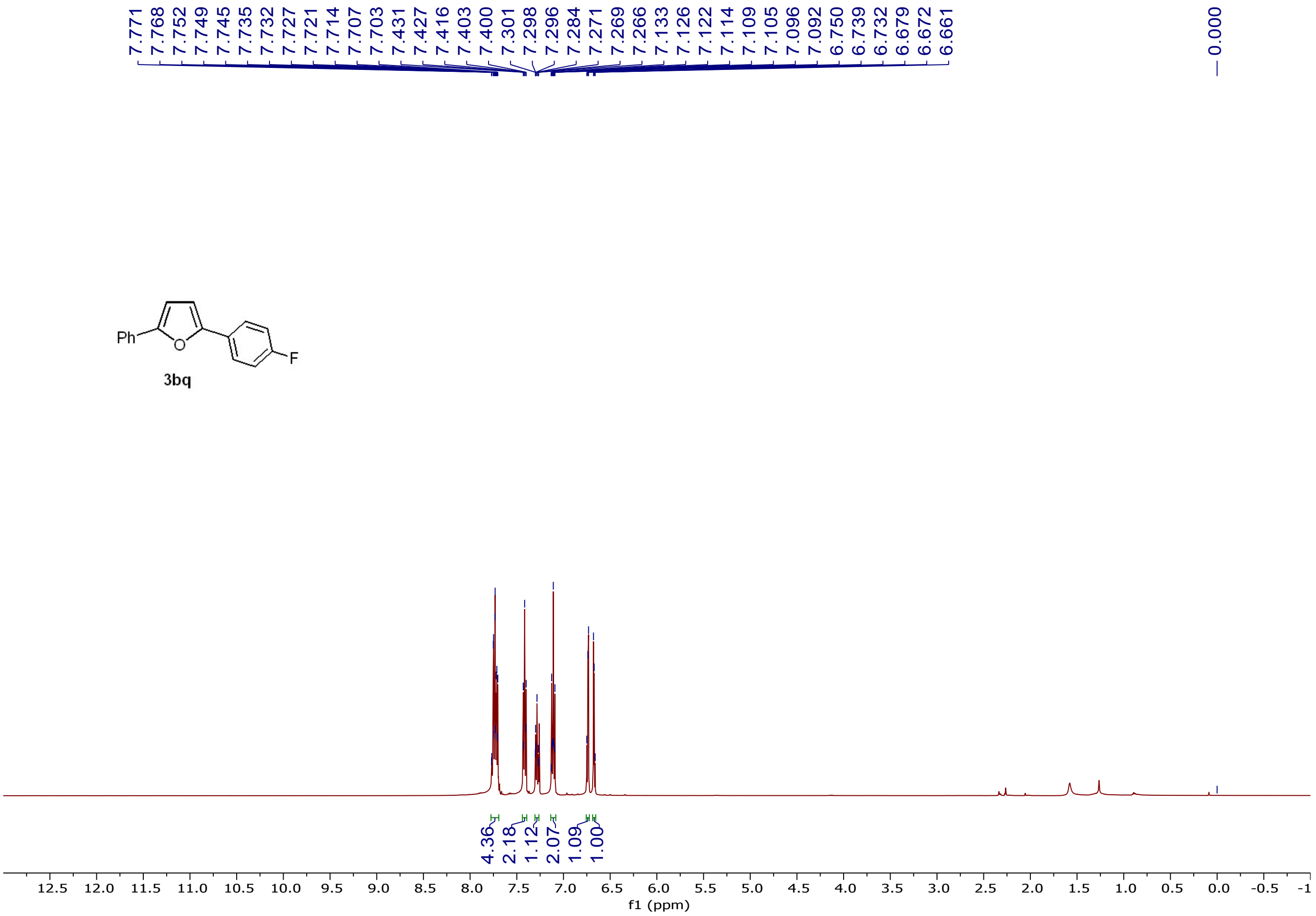
7.771
7.768
7.752
7.749
7.745
7.735
7.732
7.727
7.721
7.714
7.707
7.703
7.431
7.427
7.416
7.403
7.400
7.301
7.298
7.296
7.284
7.271
7.269
7.266
7.133
7.126
7.122
7.114
7.109
7.105
7.096
7.092
6.750
6.739
6.732
6.679
6.672
6.661
0.000
Ph
O
F
3bq
4.36
2.18
1.12
2.07
1.09
1.00
12.5 12.0 11.5 11.0 10.5 10.0 9.5 9.0 8.5 8.0 7.5 7.0 6.5 6.0 5.5 5.0 4.5 4.0 3.5 3.0 2.5 2.0 1.5 1.0 0.5 0.0 -0.5 -1
f1 (ppm)

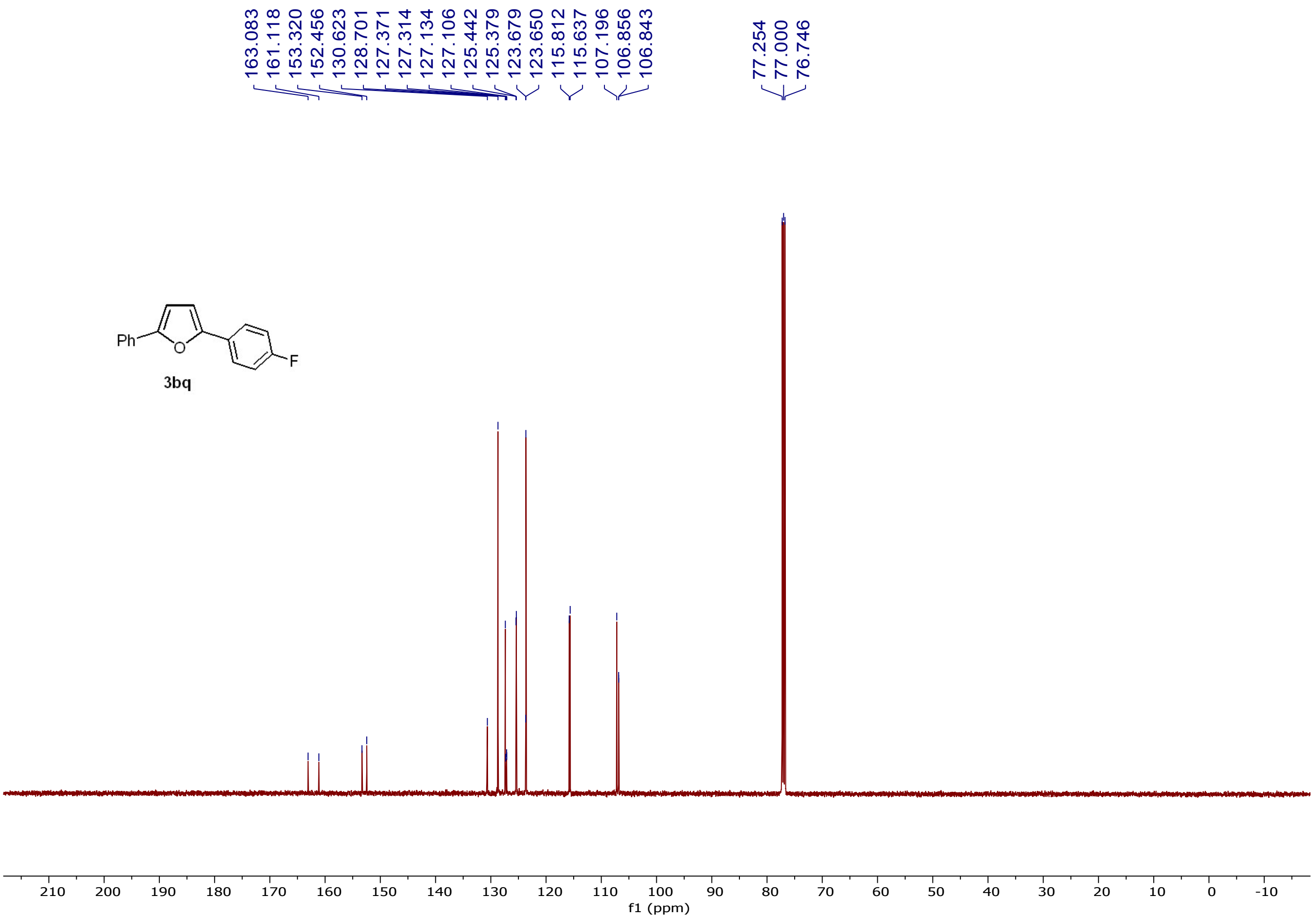
163.083
161.118
153.320
152.456
130.623
128.701
127.371
127.314
127.134
127.106
125.442
125.379
123.679
123.650
115.812
115.637
107.196
106.856
106.843
77.254
77.000
76.746
Ph
O
F
3bq
210 200 190 180 170 160 150 140 130 120 110 100 90 80 70 60 50 40 30 20 10 0 -10
f1 (ppm)

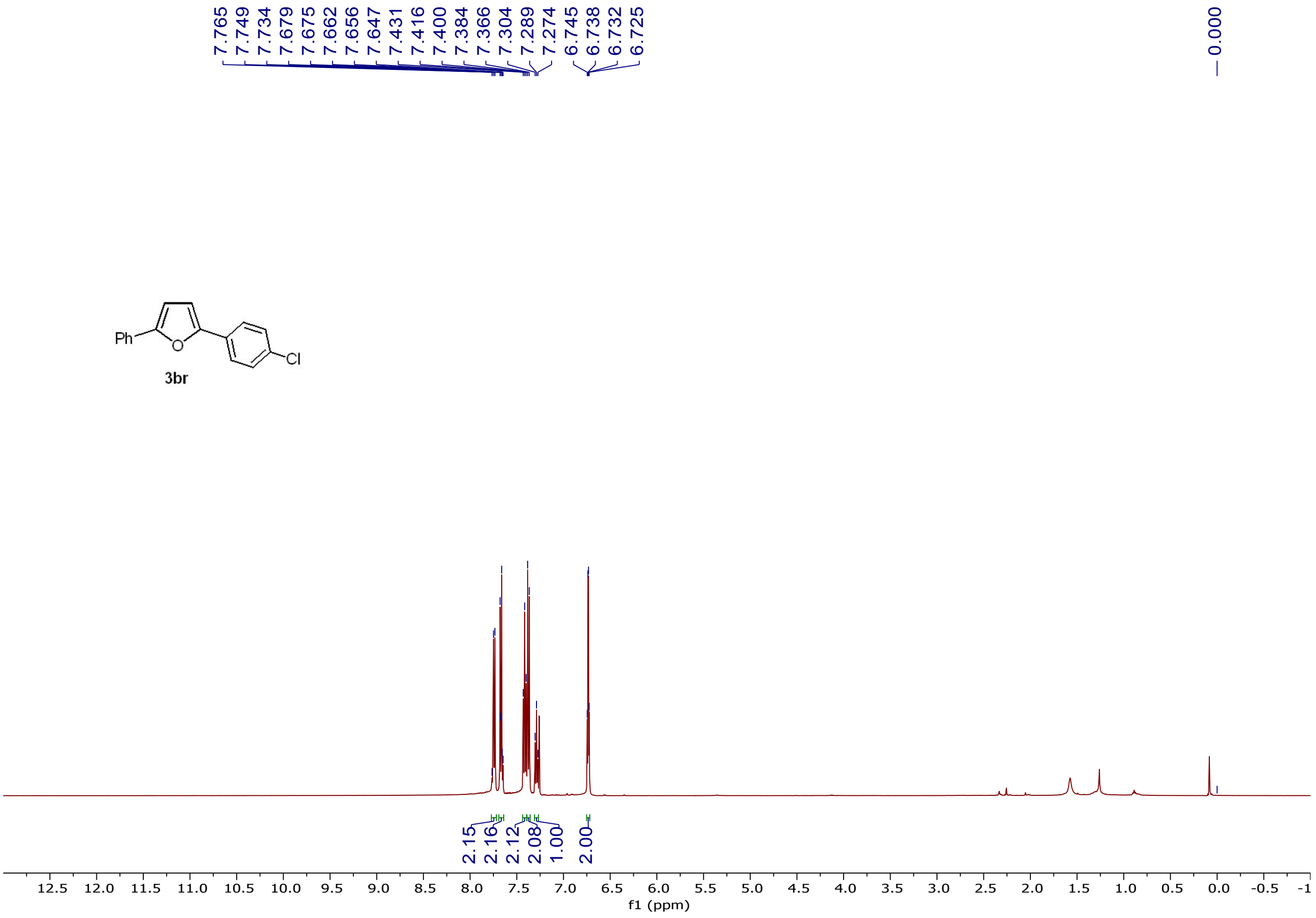
7.765
7.749
7.734
7.679
7.675
7.662
7.656
7.647
7.431
7.416
7.400
7.384
7.366
7.304
7.289
7.274
6.745
6.738
6.732
6.725
0.000
Ph
O
Cl
3br
2.15
2.16
2.12
2.08
1.00
2.00
12.5 12.0 11.5 11.0 10.5 10.0 9.5 9.0 8.5 8.0 7.5 7.0 6.5 6.0 5.5 5.0 4.5 4.0 3.5 3.0 2.5 2.0 1.5 1.0 0.5 0.0 -0.5 -1
f1 (ppm)

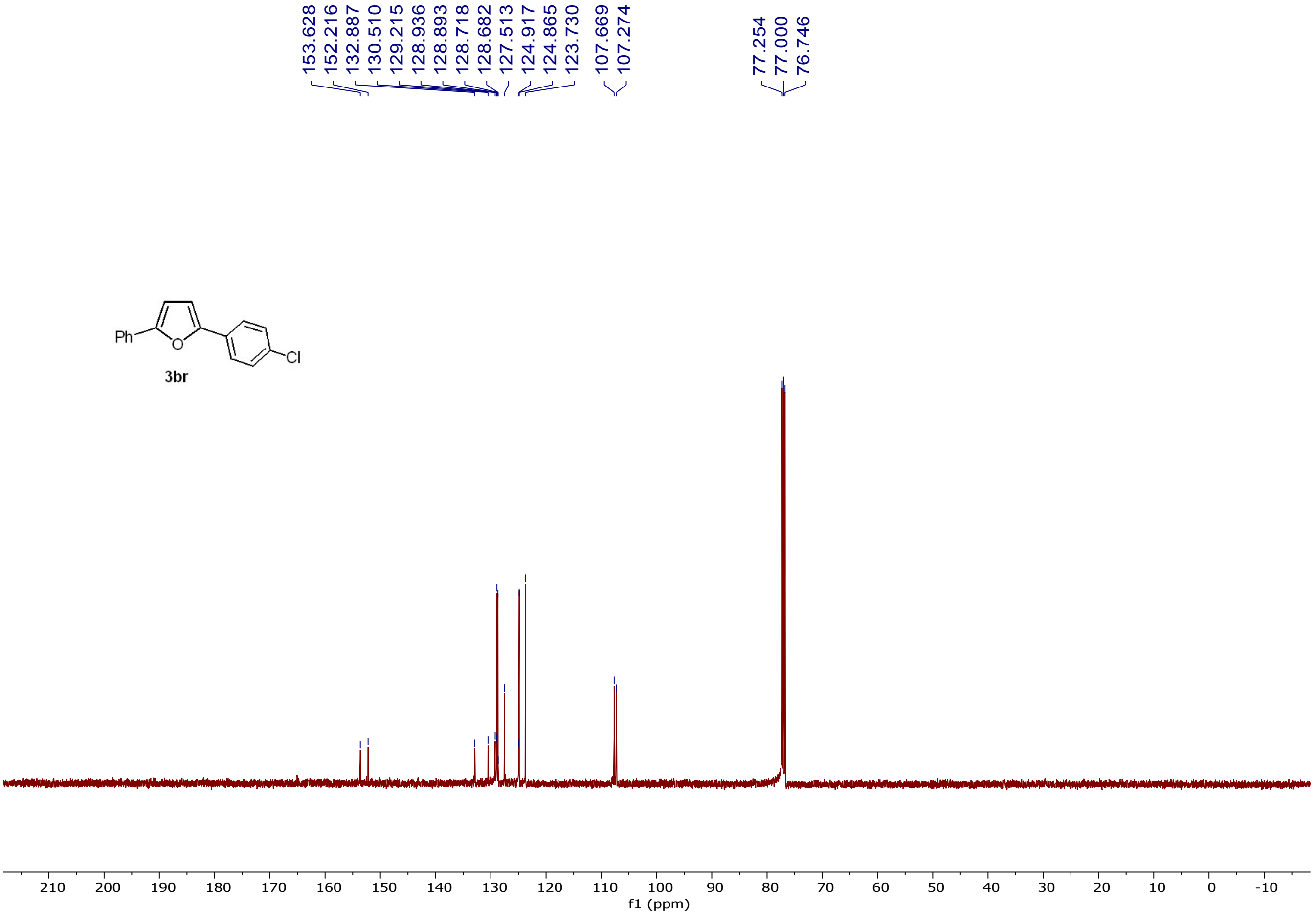
153.628
152.216
132.887
130.510
129.215
128.936
128.893
128.718
128.682
127.513
124.917
124.865
123.730
107.669
107.274
77.254
77.000
76.746
Ph
O
Cl
3br
210 200 190 180 170 160 150 140 130 120 110 100 90 80 70 60 50 40 30 20 10 0 -10
f1 (ppm)

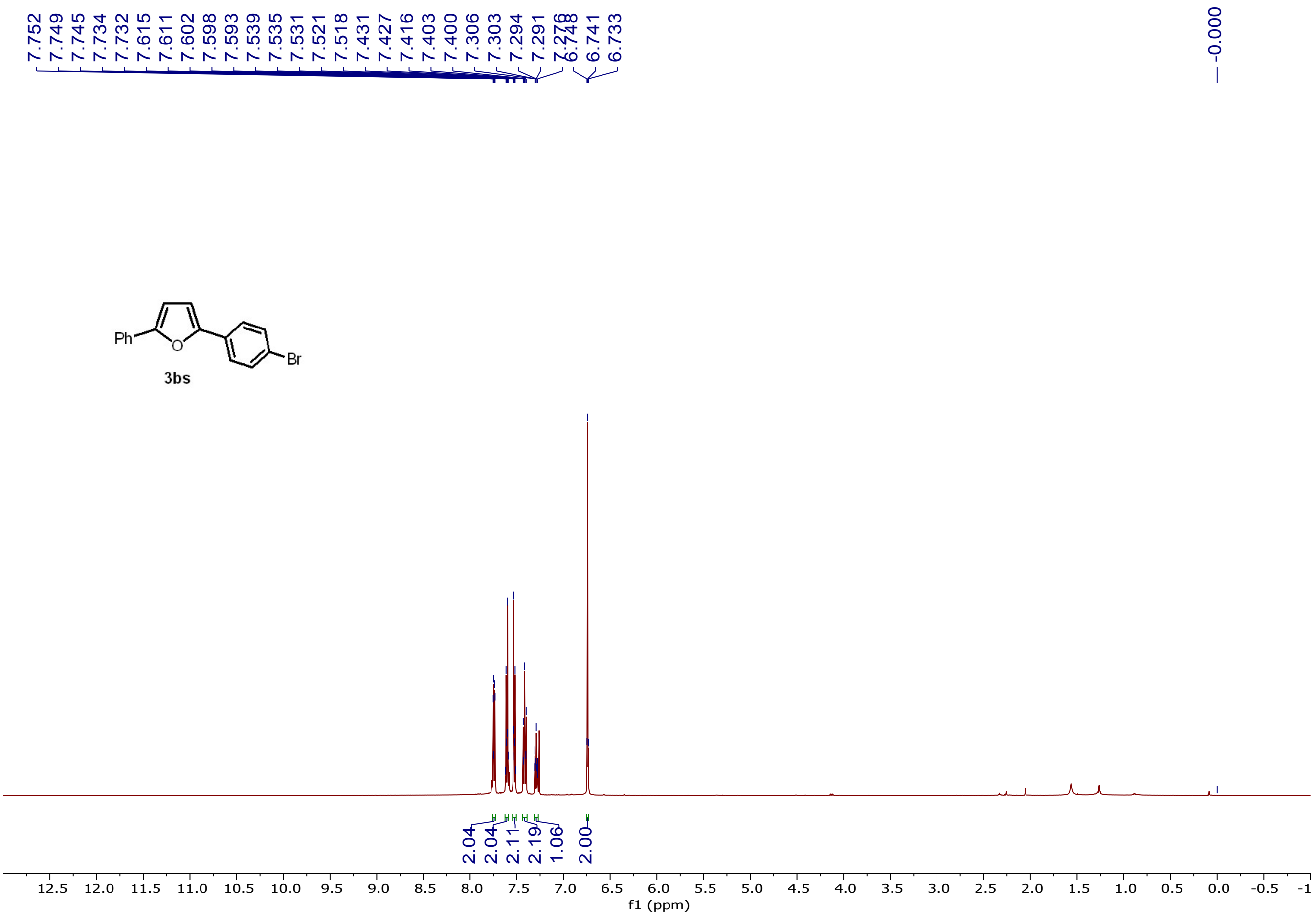
7.752
7.749
7.745
7.734
7.732
7.615
7.611
7.602
7.598
7.593
7.539
7.535
7.531
7.521
7.518
7.431
7.427
7.416
7.403
7.400
7.306
7.303
7.294
7.291
7.276
6.748
6.741
6.733
-0.000
Ph
O
Br
3bs
2.04
2.04
2.11
2.19
1.06
2.00
f1 (ppm)

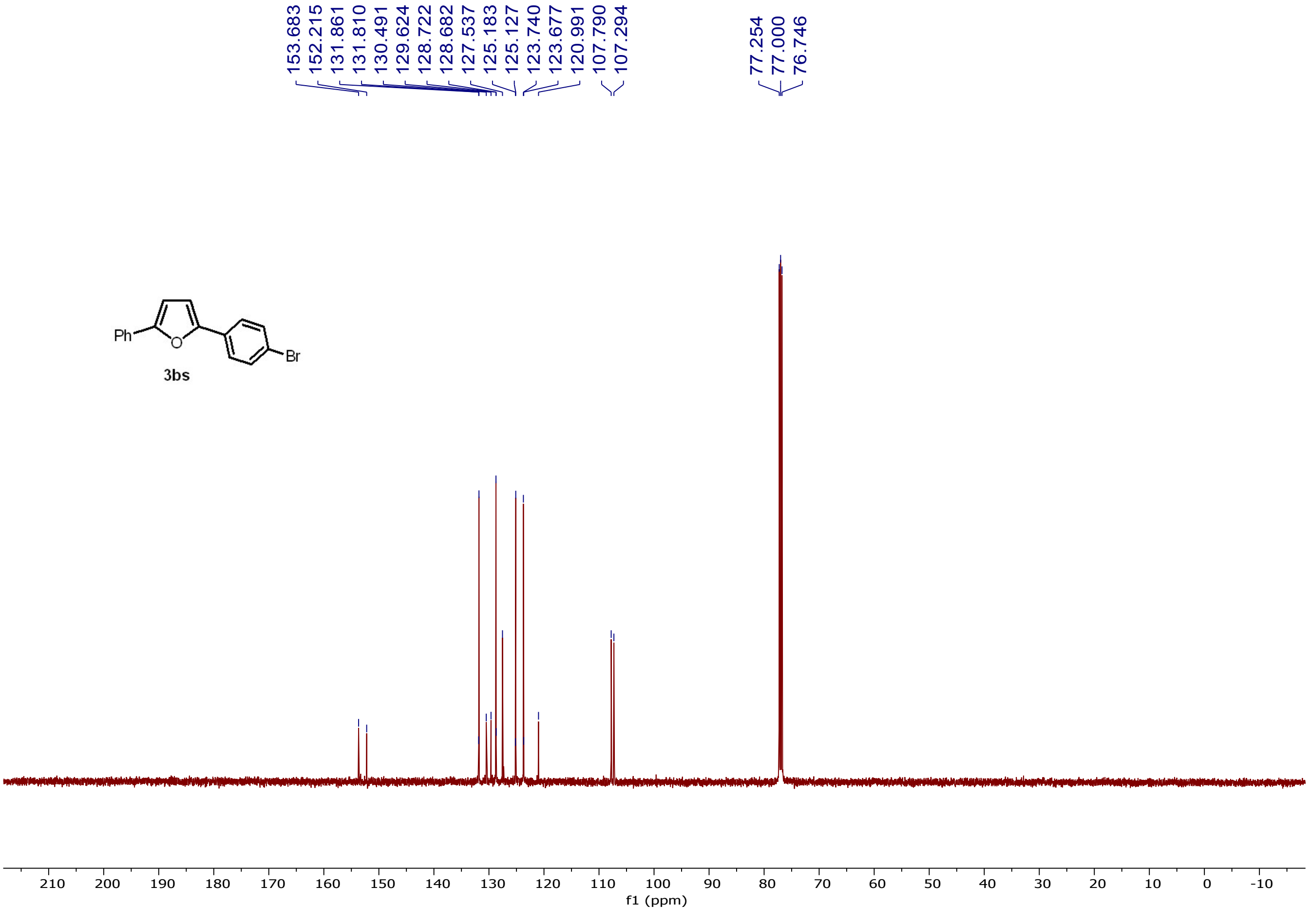
153.683
152.215
131.861
131.810
130.491
129.624
128.722
128.682
127.537
125.183
125.127
123.740
123.677
120.991
107.790
107.294
77.254
77.000
76.746
Ph
O
Br
3bs
f1 (ppm)

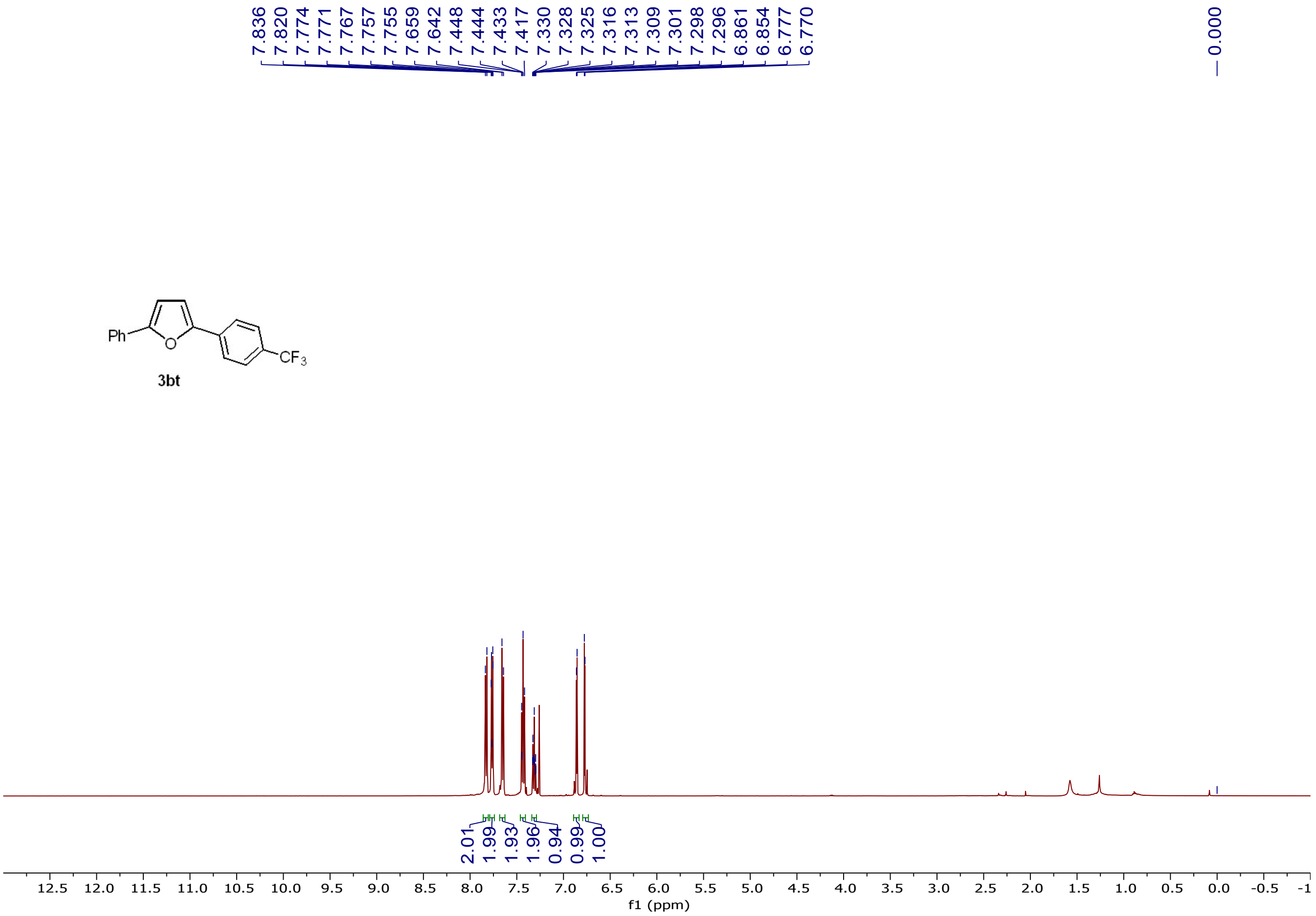
7.836
7.820
7.774
7.771
7.767
7.757
7.755
7.659
7.642
7.448
7.444
7.433
7.417
7.330
7.328
7.325
7.316
7.313
7.309
7.301
7.298
7.296
6.861
6.854
6.777
6.770
0.000
Ph
O
CF3
3bt
2.01
1.99
1.93
1.96
0.94
0.99
1.00
12.5 12.0 11.5 11.0 10.5 10.0 9.5 9.0 8.5 8.0 7.5 7.0 6.5 6.0 5.5 5.0 4.5 4.0 3.5 3.0 2.5 2.0 1.5 1.0 0.5 0.0 -0.5 -1
f1 (ppm)

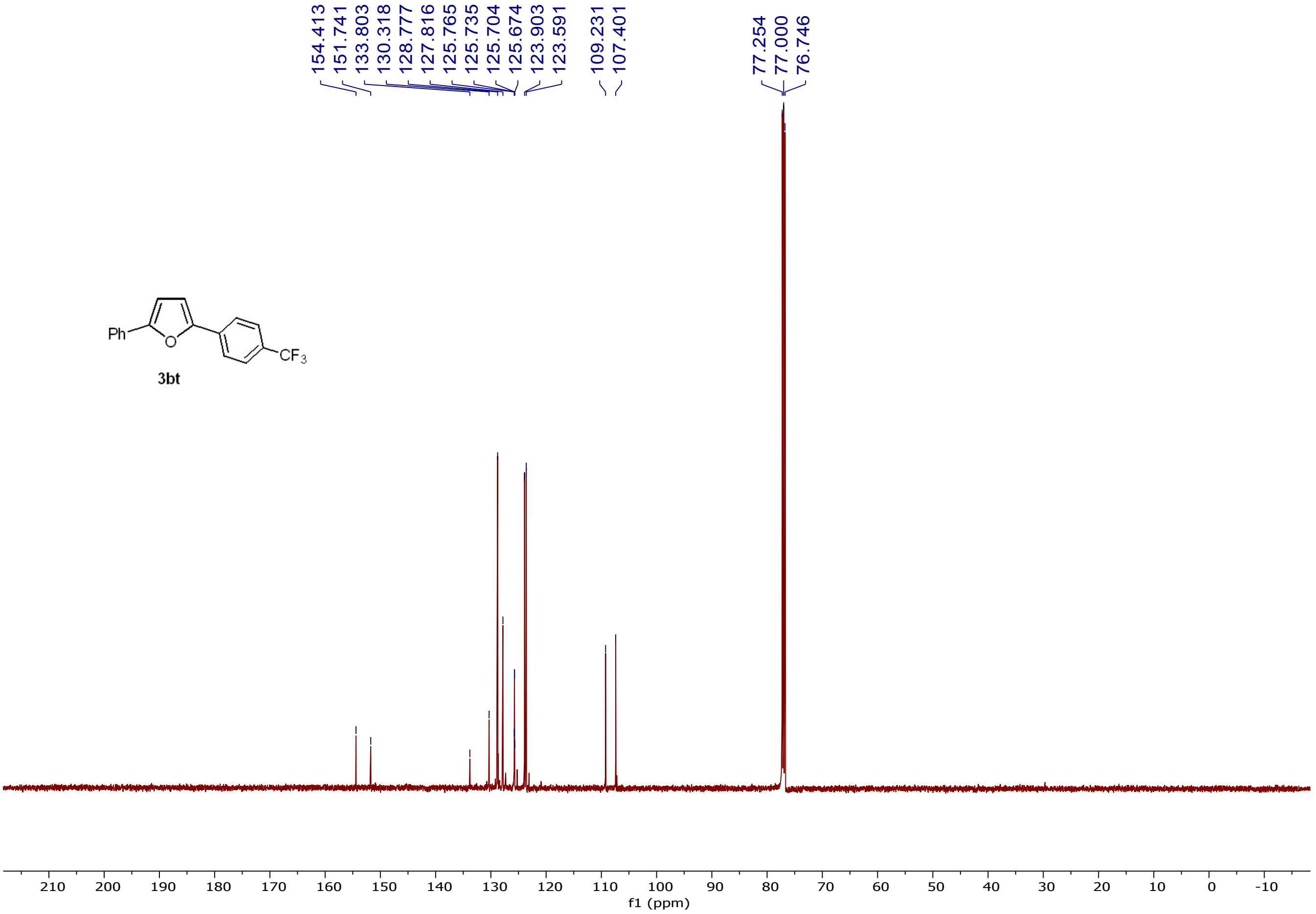
154.413
151.741
133.803
130.318
128.777
127.816
125.765
125.735
125.704
125.674
123.903
123.591
109.231
107.401
77.254
77.000
76.746
Ph
O
CF3
3bt
210 200 190 180 170 160 150 140 130 120 110 100 90 80 70 60 50 40 30 20 10 0 -10
f1 (ppm)

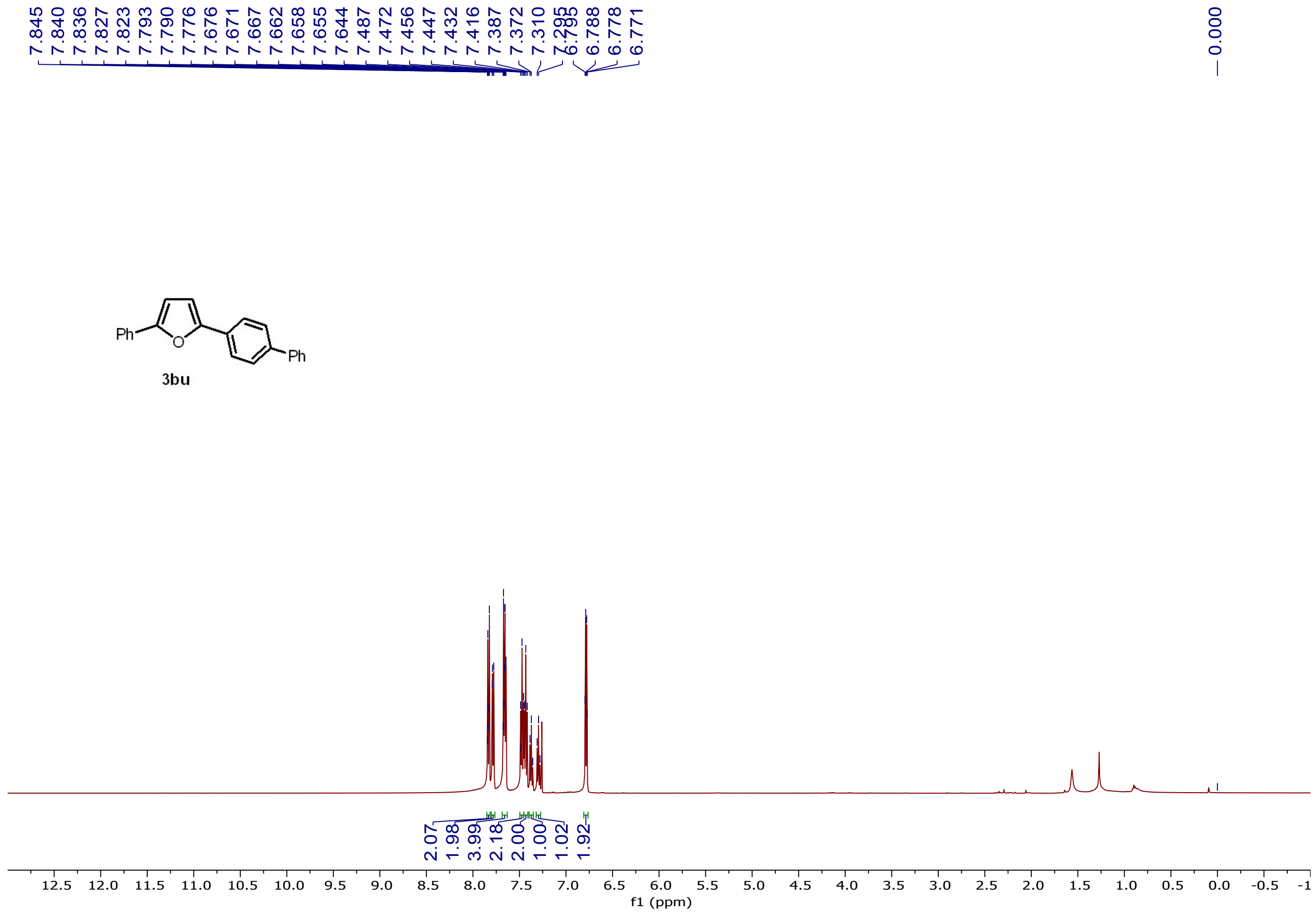

7.845
7.840
7.836
7.827
7.823
7.793
7.790
7.776
7.676
7.671
7.667
7.662
7.658
7.655
7.644
7.487
7.472
7.456
7.447
7.432
7.416
7.387
7.372
7.310
7.295
6.795
6.788
6.778
6.771
0.000
Ph
O
Ph
3bu
2.07
1.98
3.99
2.18
2.00
1.00
1.02
1.92
f1 (ppm)

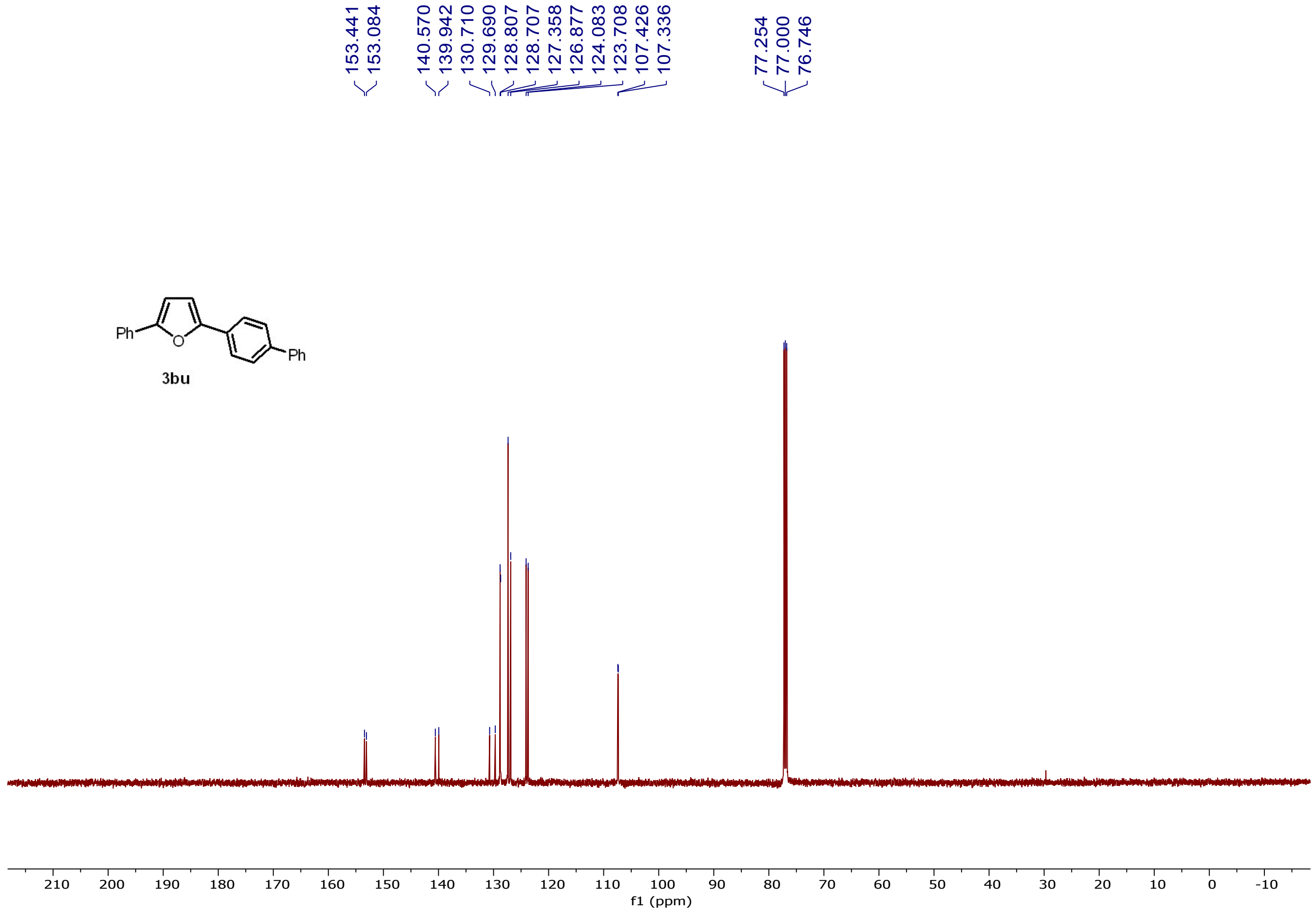

153.441
153.084
140.570
139.942
130.710
129.690
128.807
128.707
127.358
126.877
124.083
123.708
107.426
107.336
77.254
77.000
76.746
Ph
O
Ph
3bu
f1 (ppm)

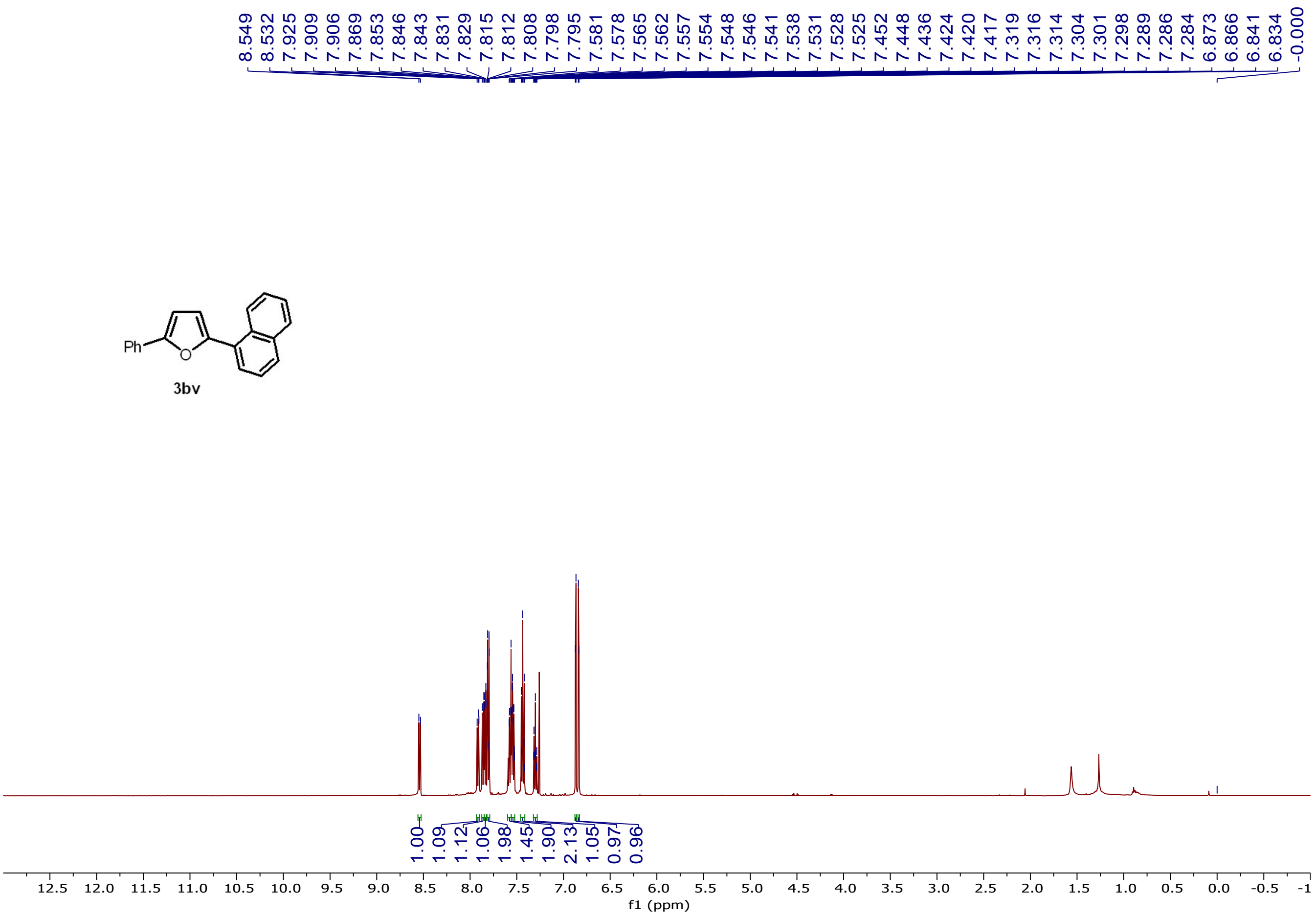
8.549 8.532 7.925 7.909 7.906 7.869 7.853 7.846 7.843 7.831 7.829 7.815 7.812 7.808 7.798 7.795 7.581 7.578 7.565 7.562 7.557 7.554 7.548 7.546 7.541 7.538 7.531 7.528 7.525 7.452 7.448 7.436 7.424 7.420 7.417 7.319 7.316 7.314 7.304 7.301 7.298 7.289 7.286 7.284 6.873 6.866 6.841 6.834 -0.000
Ph
O
3bv
1.00 1.09 1.12 1.06 1.98 1.45 1.90 2.13 1.05 0.97 0.96
f1 (ppm)

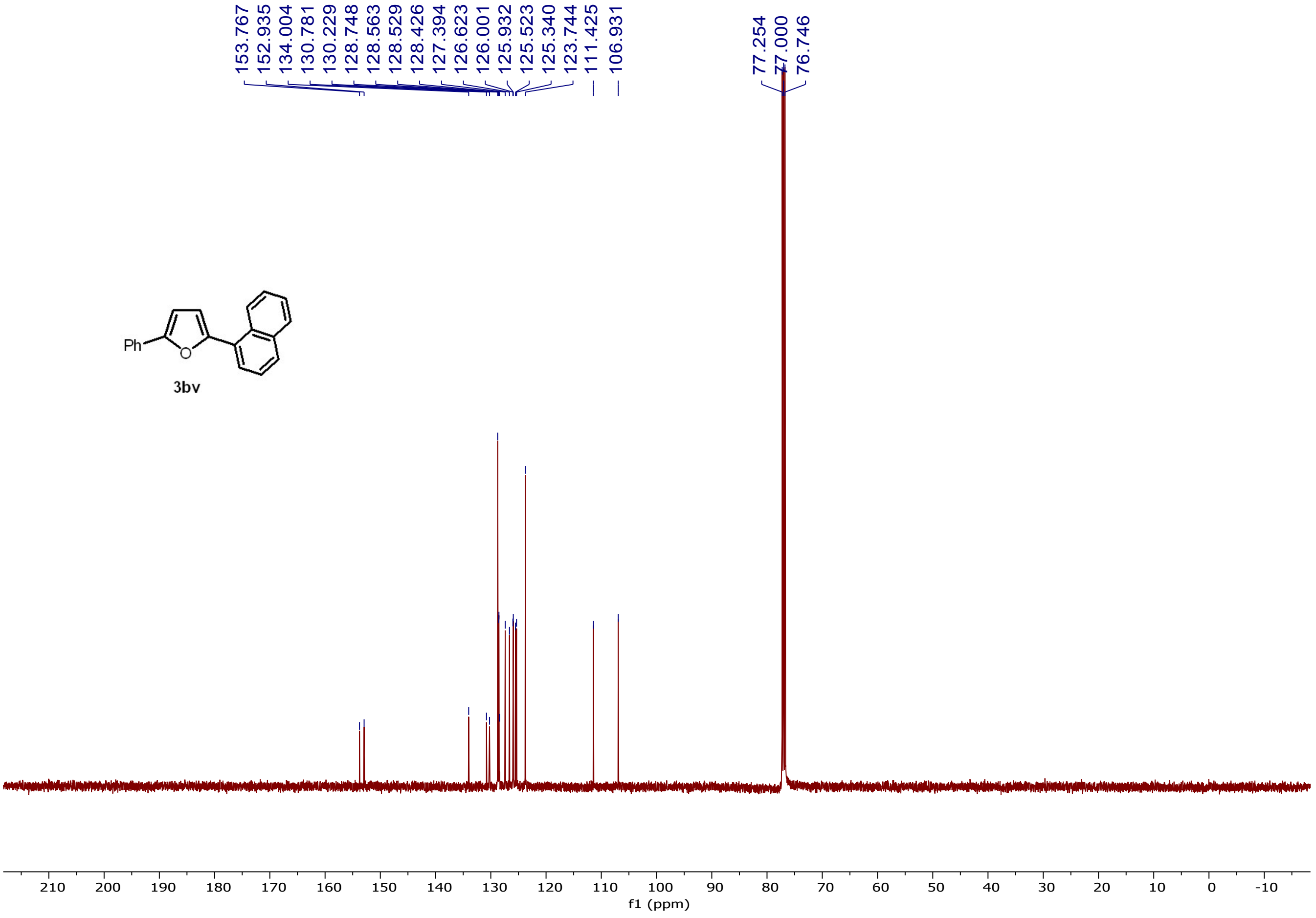
153.767 152.935 134.004 130.781 130.229 128.748 128.563 128.529 128.426 127.394 126.623 126.001 125.932 125.523 125.340 123.744 111.425 106.931
77.254 77.000 76.746
Ph
O
3bv
f1 (ppm)

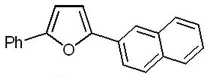

3bw

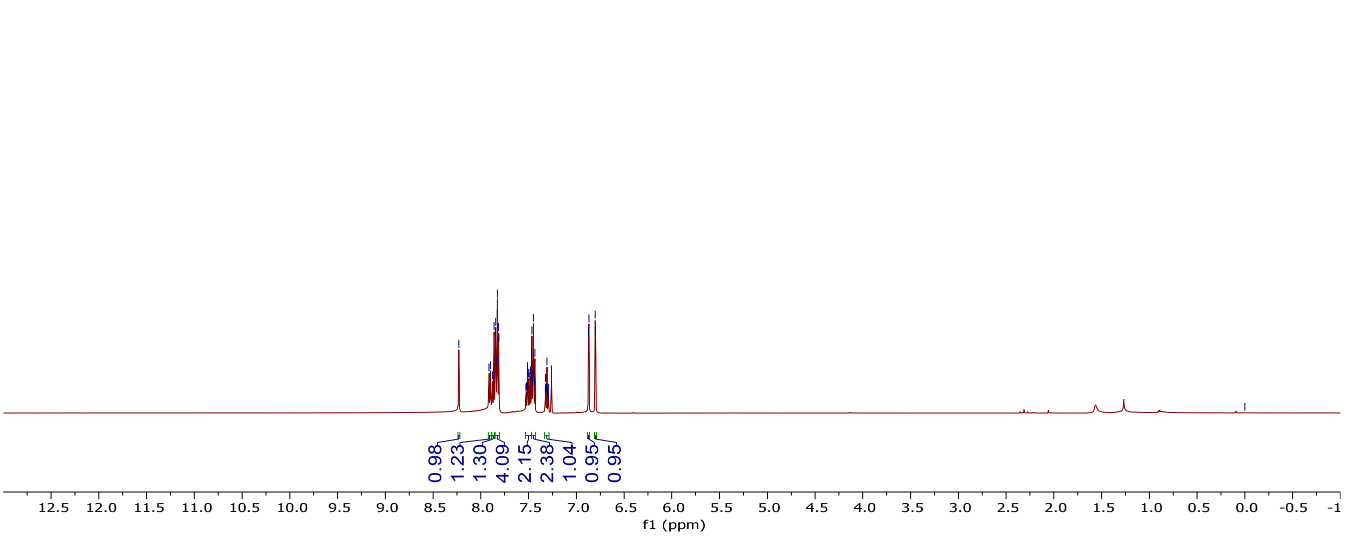
8.230
7.915
7.899
7.879
7.862
7.847
7.843
7.839
7.830
7.827
7.813
7.810
7.528
7.525
7.514
7.511
7.508
7.498
7.495
7.484
7.481
7.470
7.468
7.465
7.461
7.454
7.450
7.437
7.434
7.325
7.322
7.320
7.311
7.308
7.304
7.295
7.293
7.290
6.875
6.868
6.804
6.797
0.000
0.98
1.23
1.30
4.09
2.15
2.38
1.04
0.95
0.95
12.5 12.0 11.5 11.0 10.5 10.0 9.5 9.0 8.5 8.0 7.5 7.0 6.5 6.0 5.5 5.0 4.5 4.0 3.5 3.0 2.5 2.0 1.5 1.0 0.5 0.0 -0.5 -1
f1 (ppm)

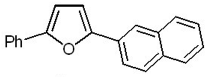

3bw

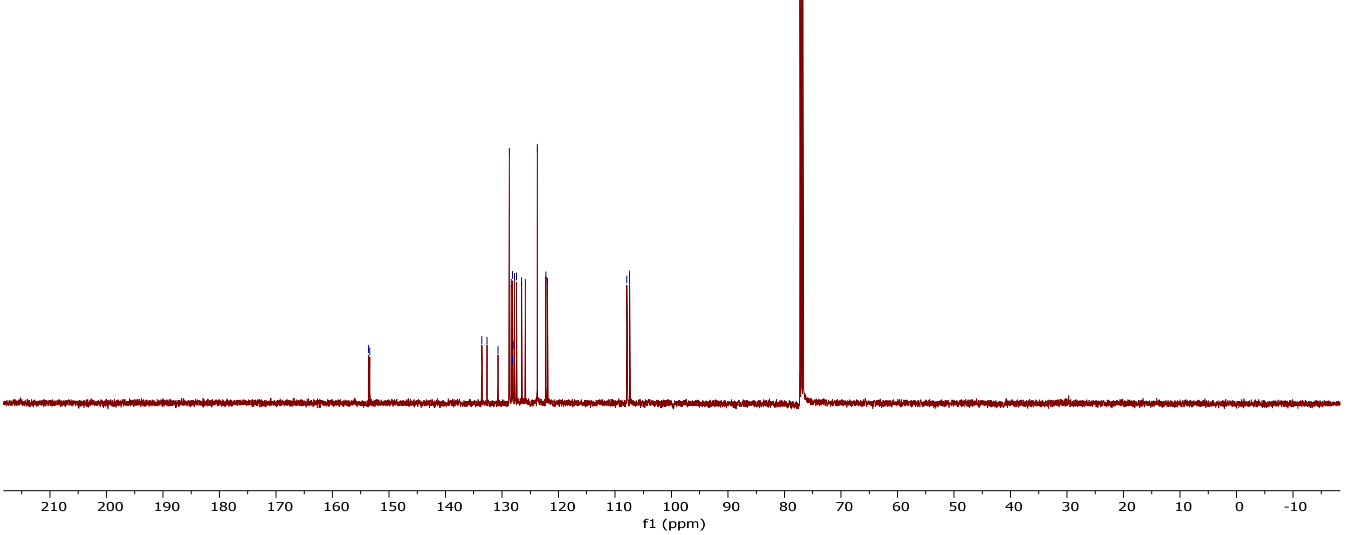
153.611
153.391
133.560
132.653
130.704
128.721
128.379
128.122
128.109
128.044
127.757
127.407
126.500
125.872
123.771
122.222
121.921
107.894
107.385
77.254
77.000
76.746
210 200 190 180 170 160 150 140 130 120 110 100 90 80 70 60 50 40 30 20 10 0 -10
f1 (ppm)

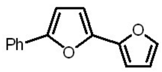

3bx

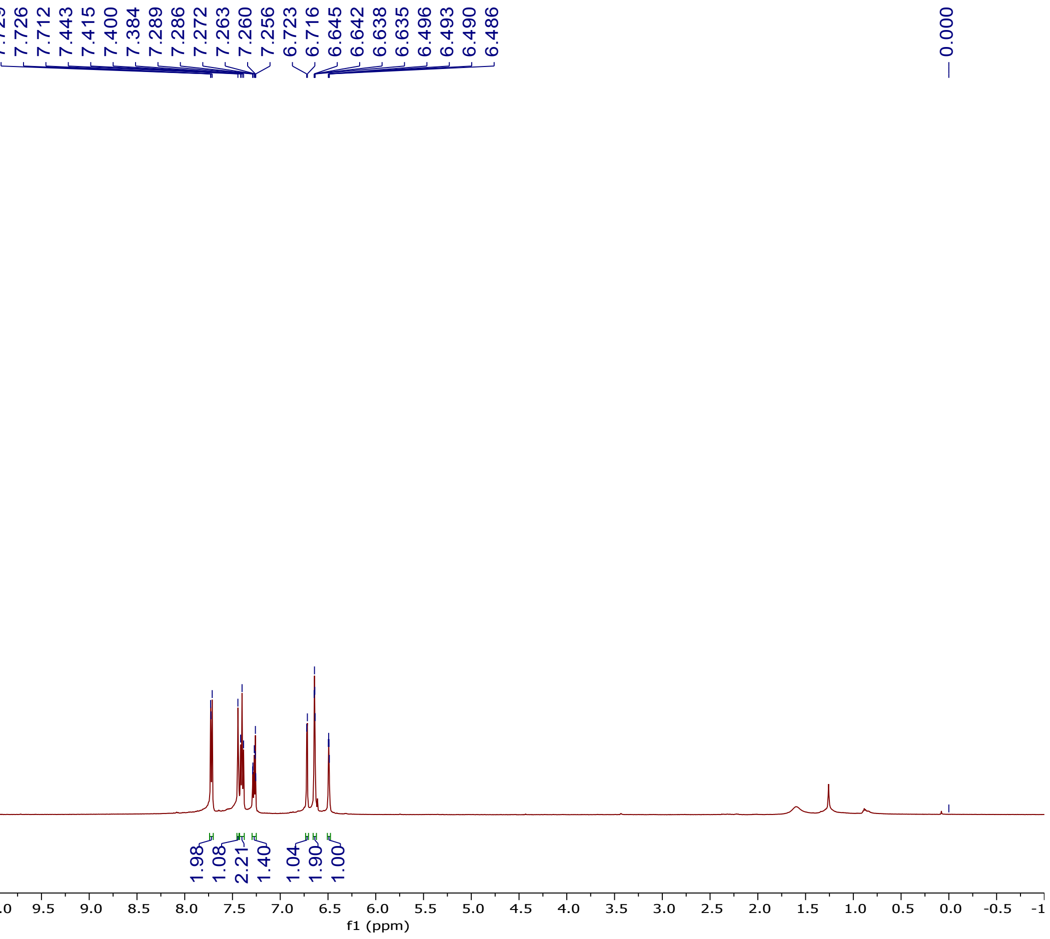

7.729
7.726
7.712
7.443
7.415
7.400
7.384
7.289
7.286
7.272
7.263
7.260
7.256
6.723
6.716
6.645
6.642
6.638
6.635
6.496
6.493
6.490
6.486
0.000
1.98
1.08
2.21
1.40
1.04
1.90
1.00
f1 (ppm)

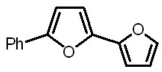

3bx

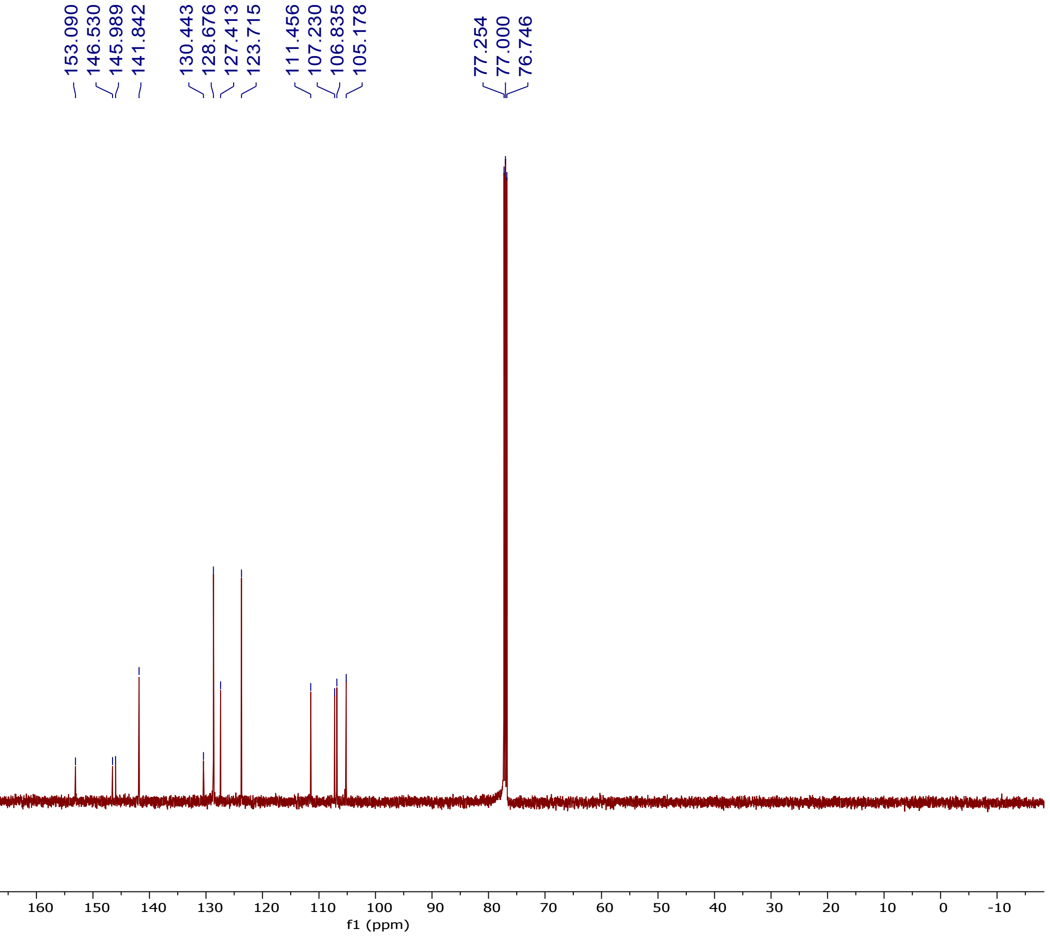

153.090
146.530
145.989
141.842
130.443
128.676
127.413
123.715
111.456
107.230
106.835
105.178
77.254
77.000
76.746
f1 (ppm)

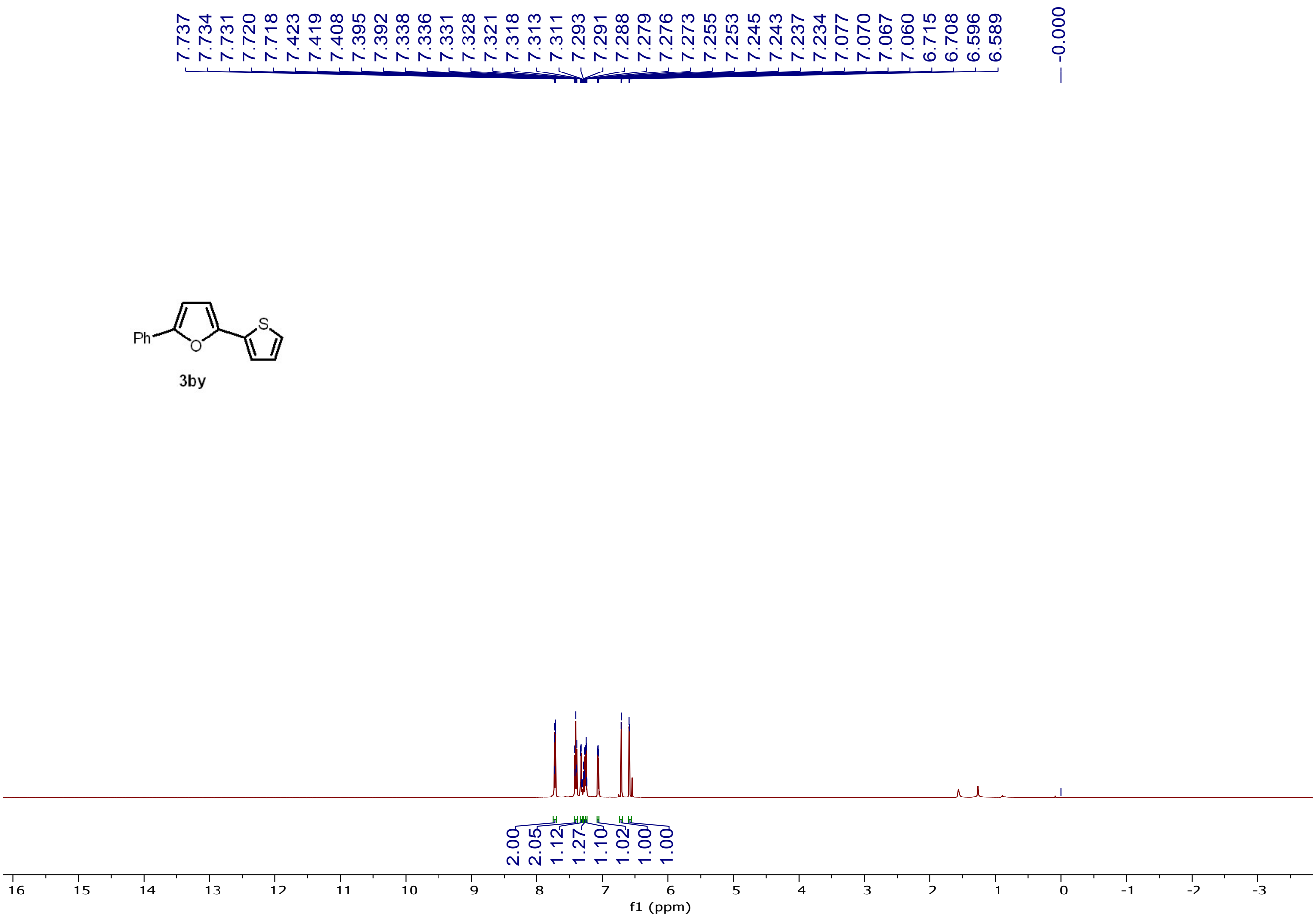
7.737
7.734
7.731
7.720
7.718
7.423
7.419
7.408
7.395
7.392
7.338
7.336
7.331
7.328
7.321
7.318
7.313
7.311
7.293
7.291
7.288
7.279
7.276
7.273
7.255
7.253
7.245
7.243
7.237
7.234
7.077
7.070
7.067
7.060
6.715
6.708
6.596
6.589
-0.000
Ph
O
S
3by
2.00
2.05
1.12
1.27
1.10
1.02
1.00
1.00
16 15 14 13 12 11 10 9 8 7 6 5 4 3 2 1 0 -1 -2 -3
f1 (ppm)

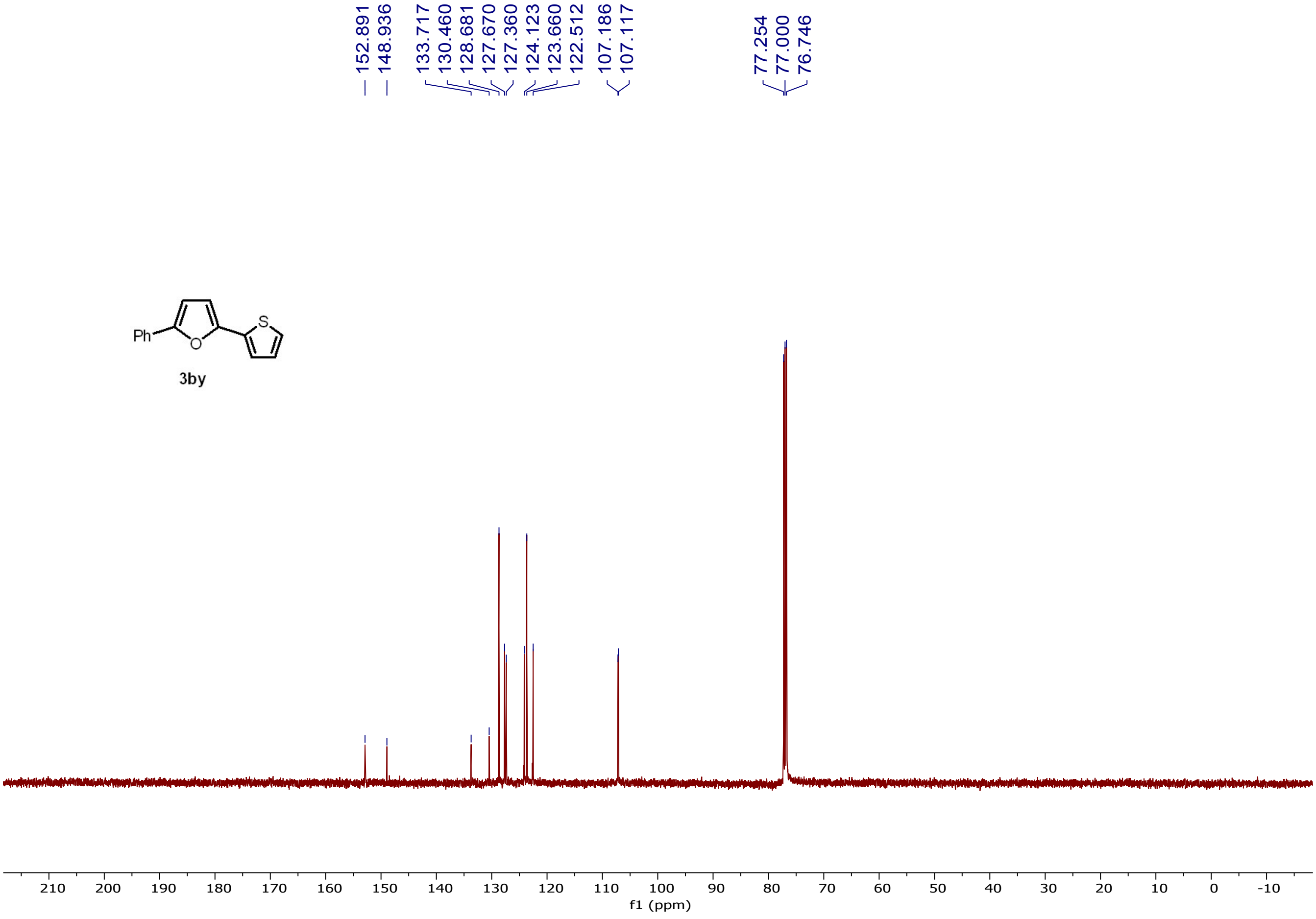
152.891
148.936
133.717
130.460
128.681
127.670
127.360
124.123
123.660
122.512
107.186
107.117
77.254
77.000
76.746
Ph
O
S
3by
210 200 190 180 170 160 150 140 130 120 110 100 90 80 70 60 50 40 30 20 10 0 -10
f1 (ppm)

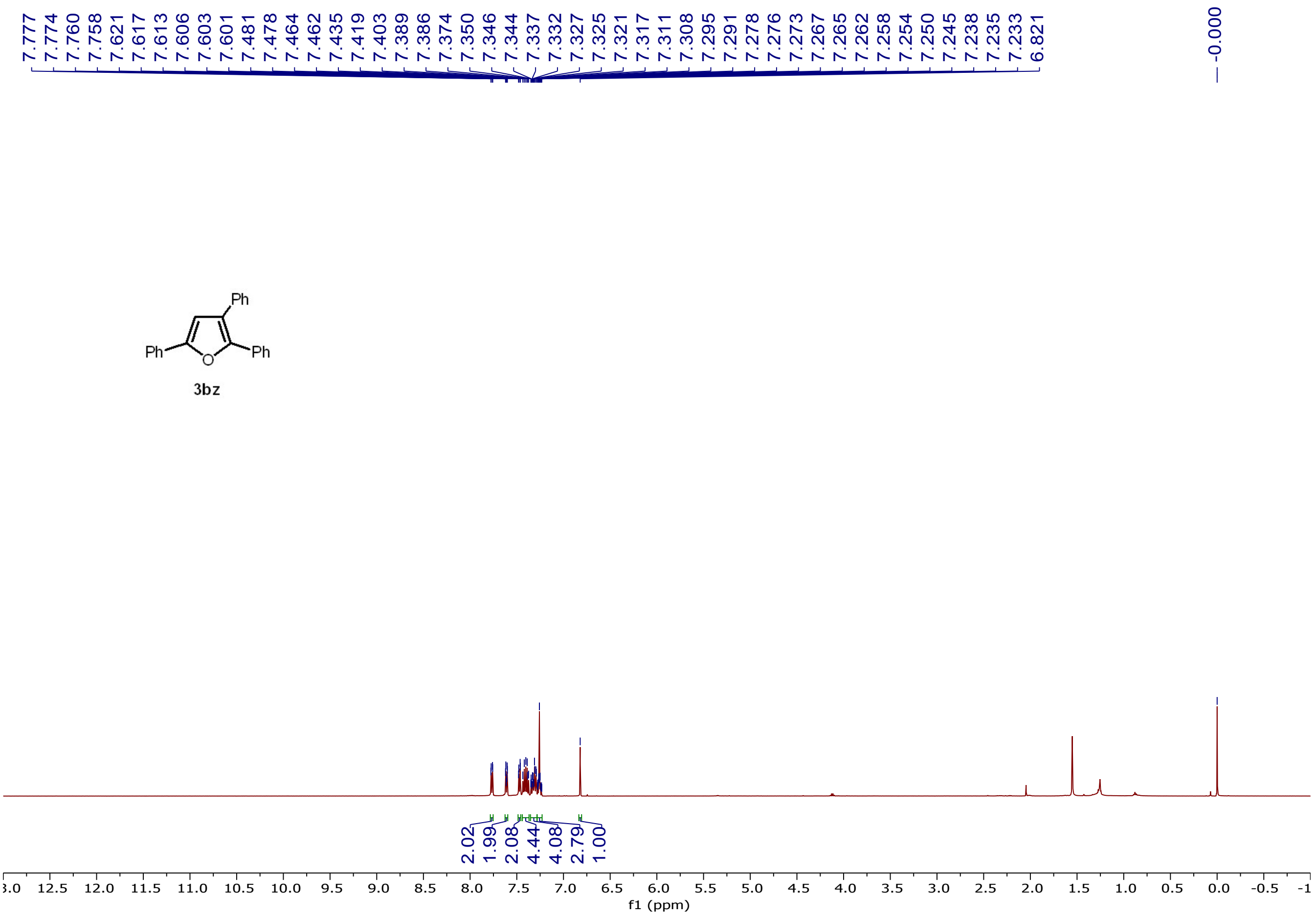
7.777
7.774
7.760
7.758
7.621
7.617
7.613
7.606
7.603
7.601
7.481
7.478
7.464
7.462
7.435
7.419
7.403
7.389
7.386
7.374
7.350
7.346
7.344
7.337
7.332
7.327
7.325
7.321
7.317
7.311
7.308
7.295
7.291
7.278
7.276
7.273
7.267
7.265
7.262
7.258
7.254
7.250
7.245
7.238
7.235
7.233
6.821
-0.000
Ph
Ph
O
Ph
3bz
2.02
1.99
2.08
4.44
4.08
2.79
1.00
f1 (ppm)

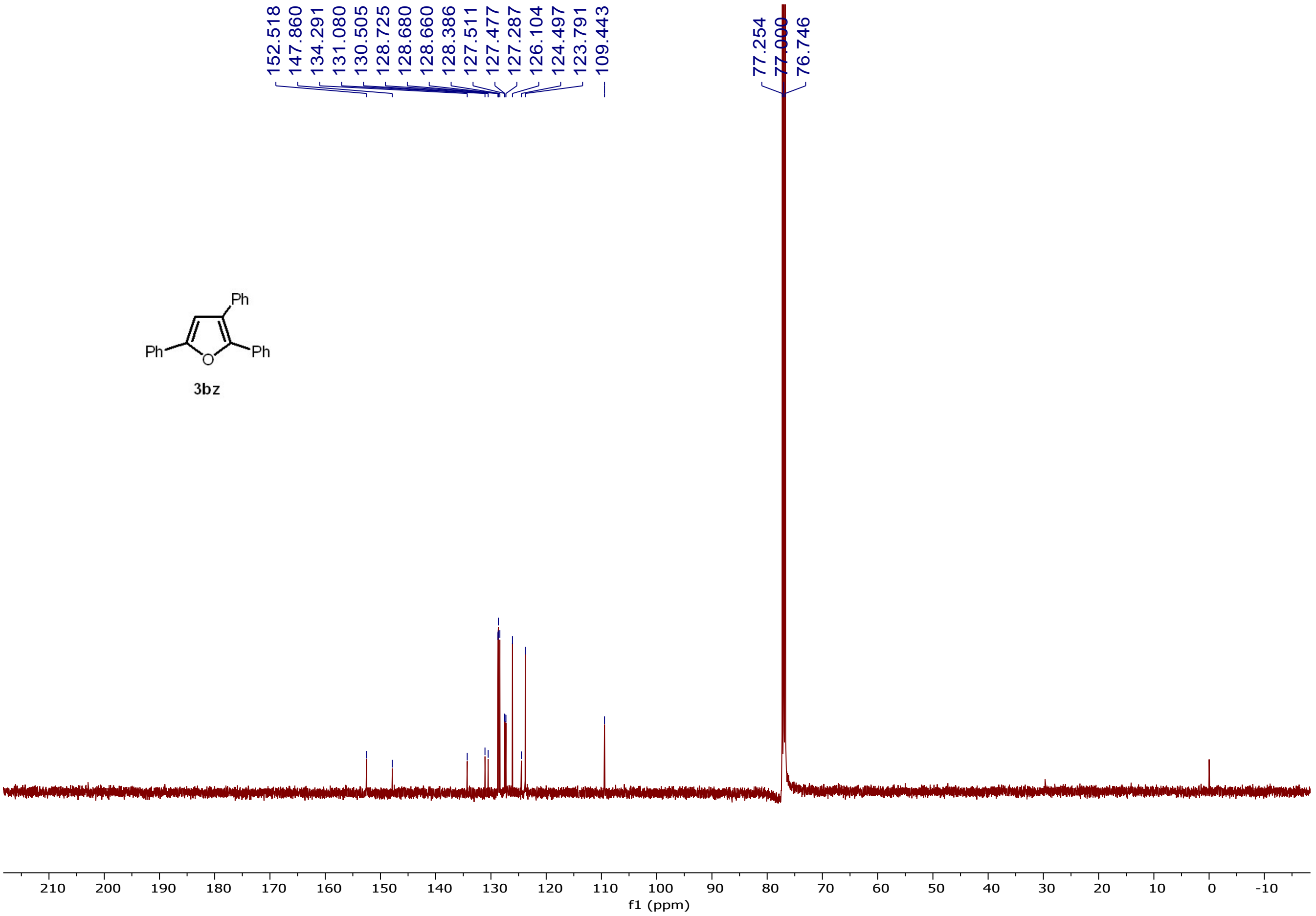
152.518
147.860
134.291
131.080
130.505
128.725
128.680
128.660
128.386
127.511
127.477
127.287
126.104
124.497
123.791
109.443
77.254
77.000
76.746
Ph
Ph
O
Ph
3bz
f1 (ppm)

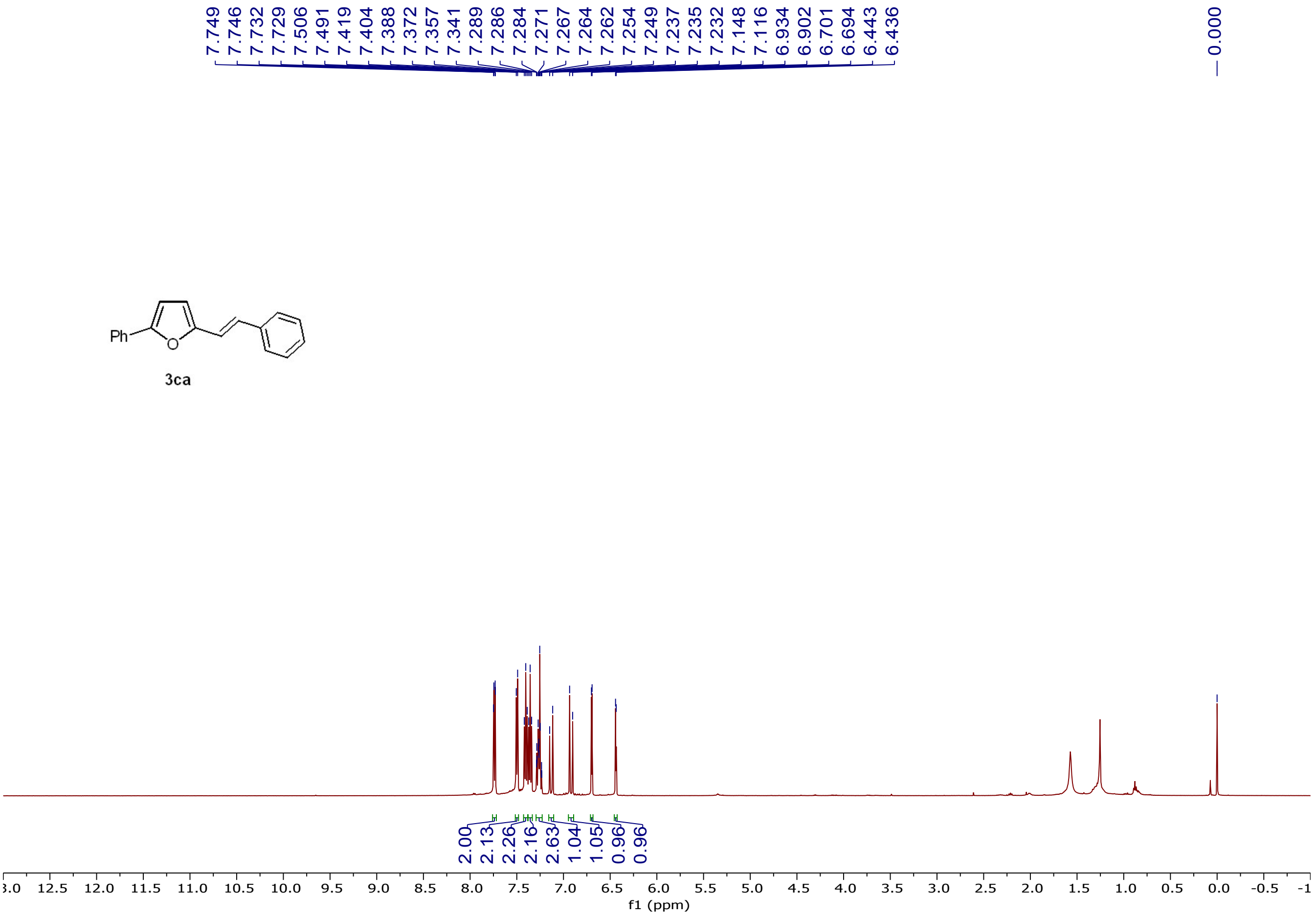
7.749
7.746
7.732
7.729
7.506
7.491
7.419
7.404
7.388
7.372
7.357
7.341
7.289
7.286
7.284
7.271
7.267
7.264
7.262
7.254
7.249
7.237
7.235
7.232
7.148
7.116
6.934
6.902
6.701
6.694
6.443
6.436
0.000
Ph
O
3ca
2.00
2.13
2.26
2.16
2.63
1.04
1.05
0.96
0.96
f1 (ppm)

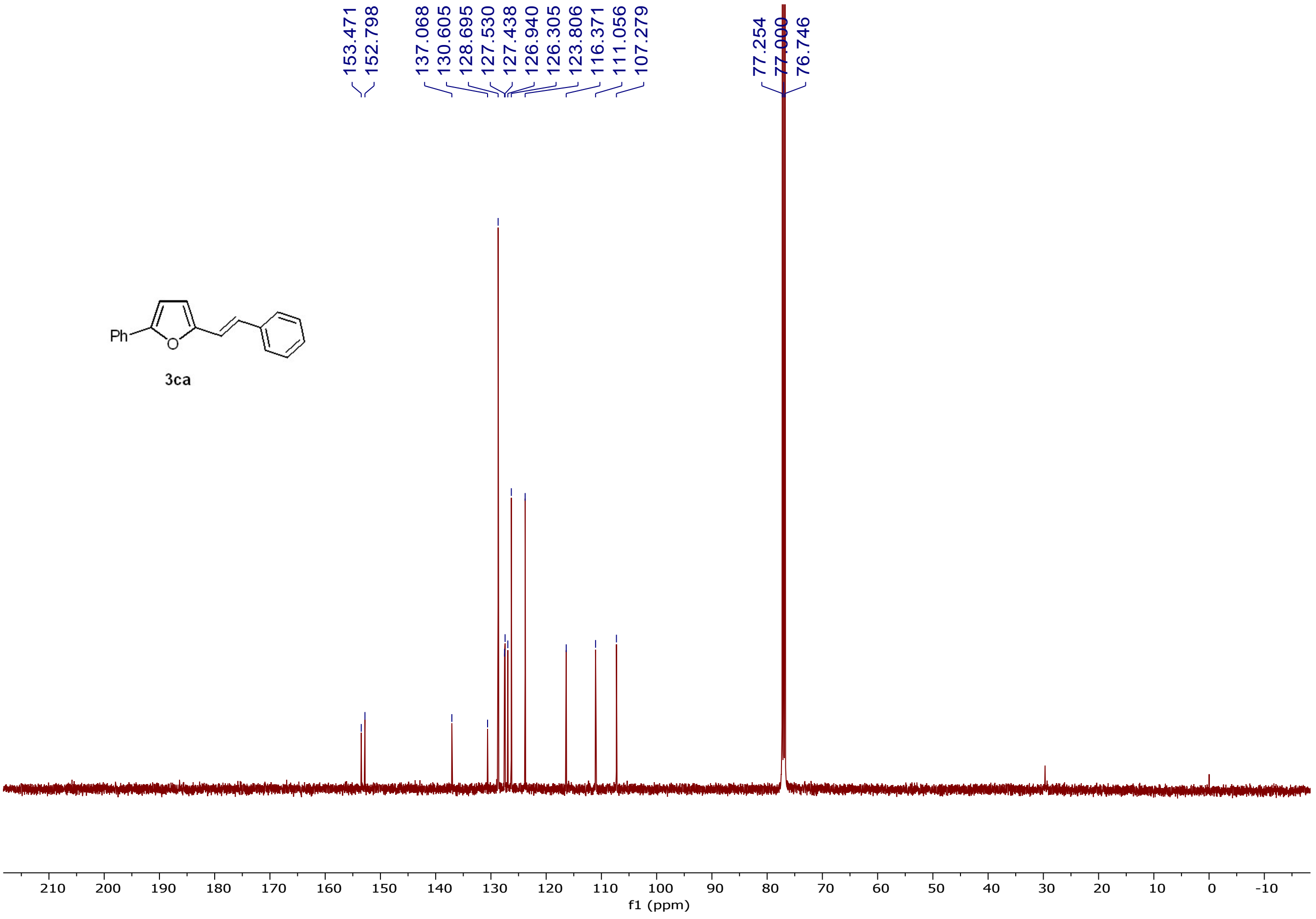
153.471
152.798
137.068
130.605
128.695
127.530
127.438
126.940
126.305
123.806
116.371
111.056
107.279
77.254
77.000
76.746
Ph
O
3ca
f1 (ppm)

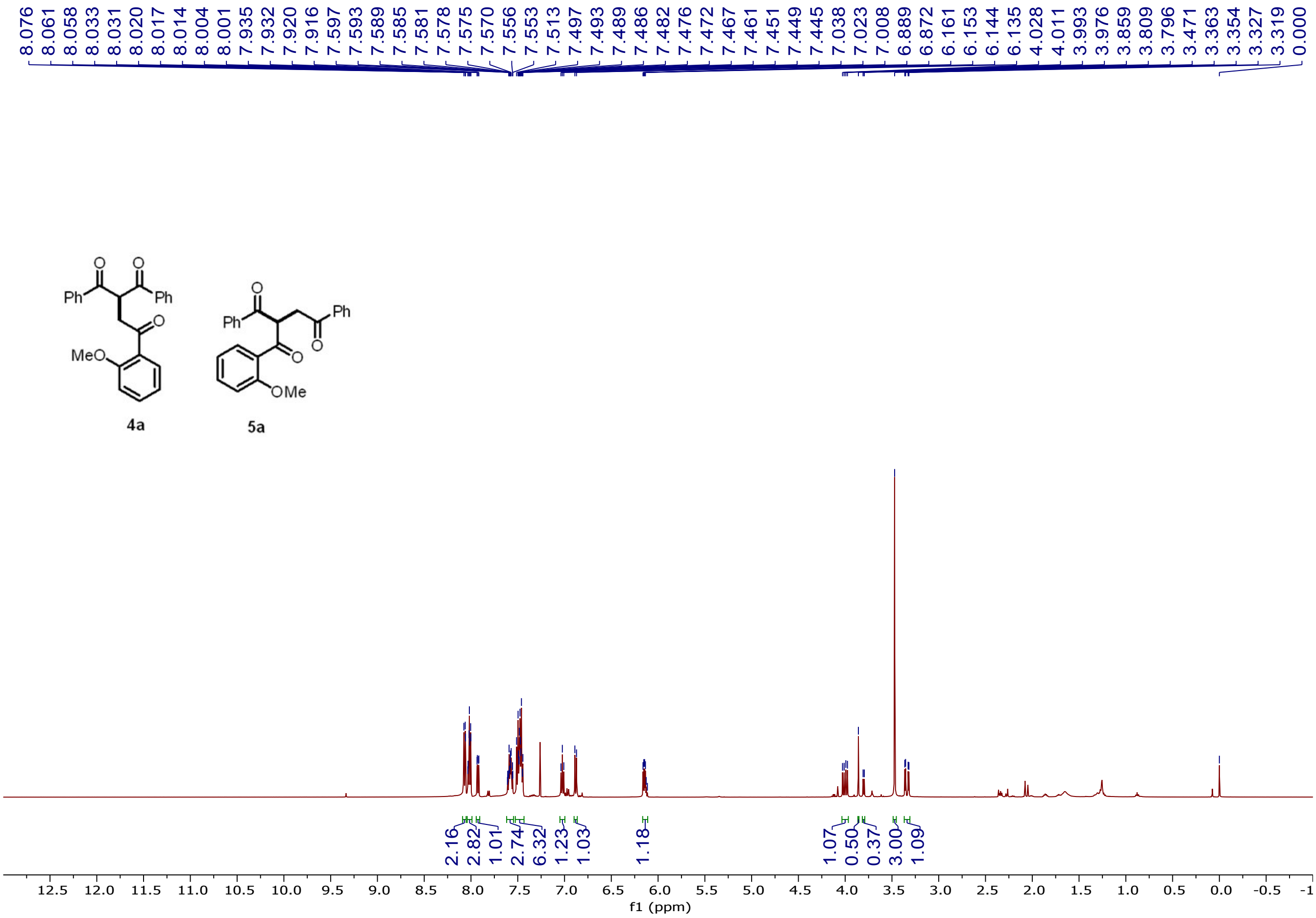
8.076
8.061
8.058
8.033
8.031
8.020
8.017
8.014
8.004
8.001
7.935
7.932
7.920
7.916
7.597
7.593
7.589
7.585
7.581
7.578
7.575
7.570
7.556
7.553
7.513
7.497
7.493
7.489
7.486
7.482
7.476
7.472
7.467
7.461
7.451
7.449
7.445
7.038
7.023
7.008
6.889
6.872
6.161
6.153
6.144
6.135
4.028
4.011
3.993
3.976
3.859
3.809
3.796
3.471
3.363
3.354
3.327
3.319
0.000
Ph
Ph
MeO
4a
Ph
Ph
OMe
5a
2.16
2.82
1.01
2.74
6.32
1.23
1.03
1.18
1.07
0.50
0.37
3.00
1.09
f1 (ppm)

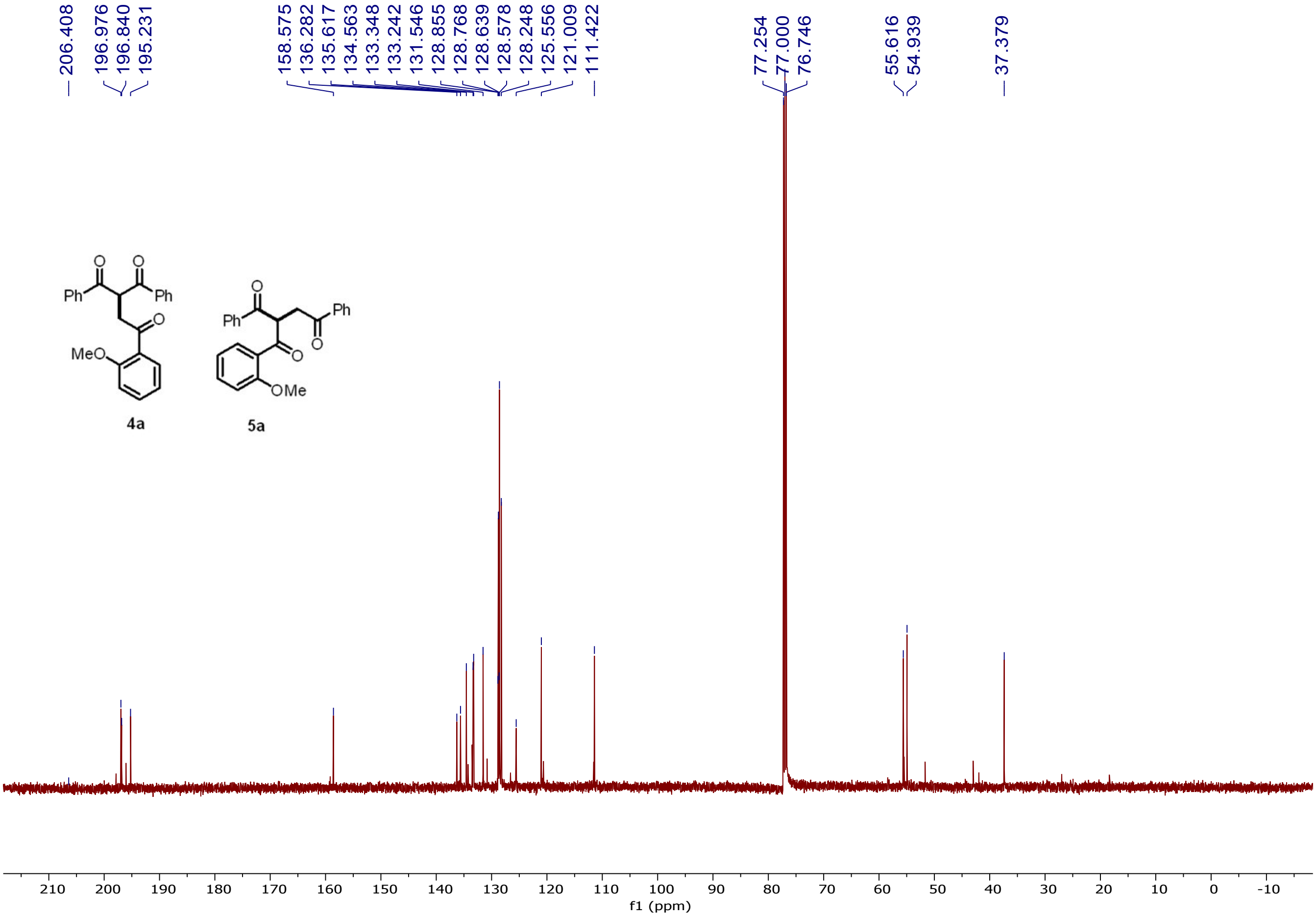
206.408
196.976
196.840
195.231
158.575
136.282
135.617
134.563
133.348
133.242
131.546
128.855
128.768
128.639
128.578
128.248
125.556
121.009
111.422
77.254
77.000
76.746
55.616
54.939
37.379
Ph
Ph
MeO
4a
Ph
Ph
OMe
5a
f1 (ppm)

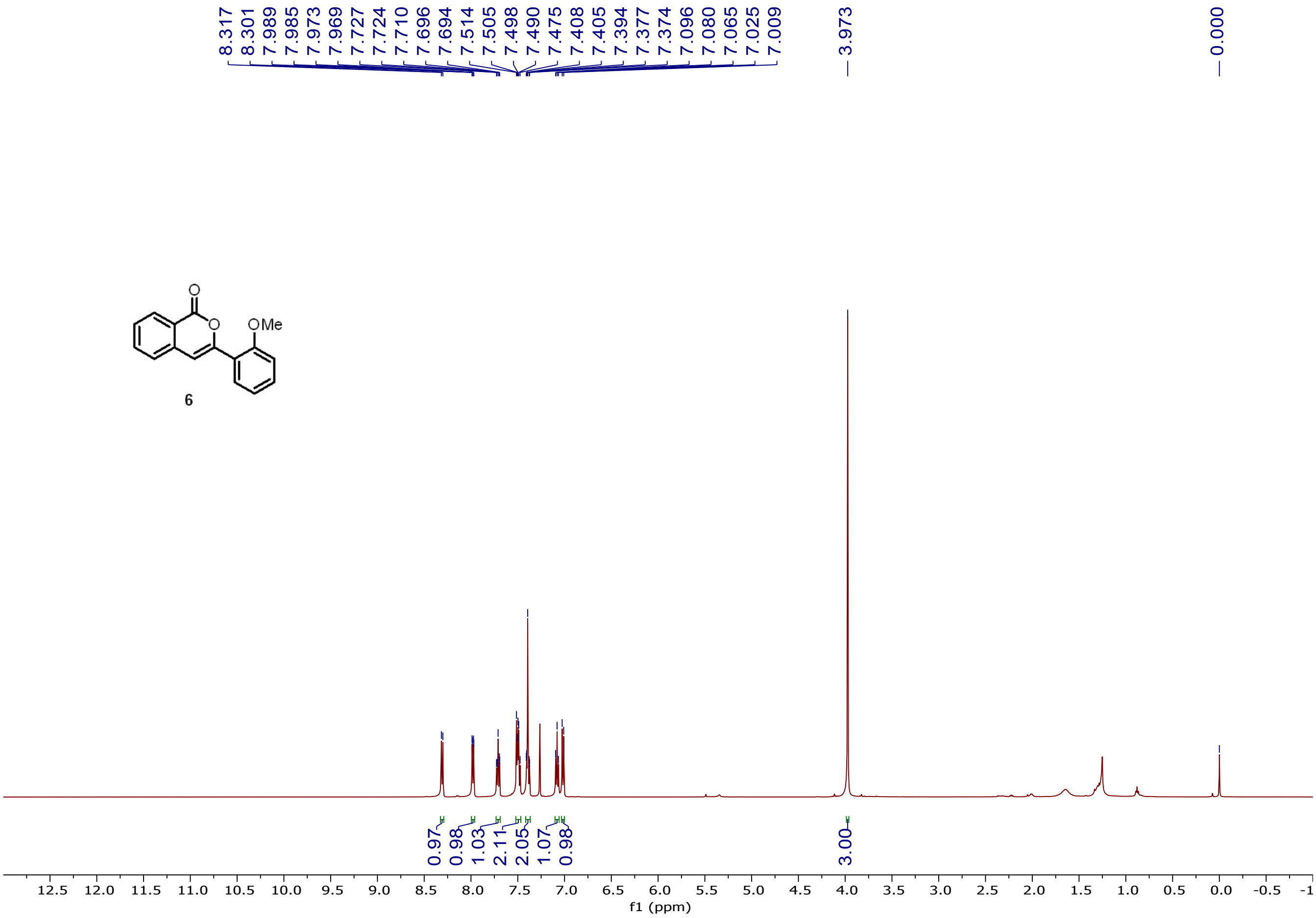
8.317 8.301 7.989 7.985 7.973 7.969 7.727 7.724 7.710 7.696 7.694 7.514 7.505 7.498 7.490 7.475 7.408 7.405 7.394 7.377 7.374 7.096 7.080 7.065 7.025 7.009
3.973
0.000
OMe
6
0.97 0.98 1.03 2.11 2.05 1.07 0.98
3.00
f1 (ppm)

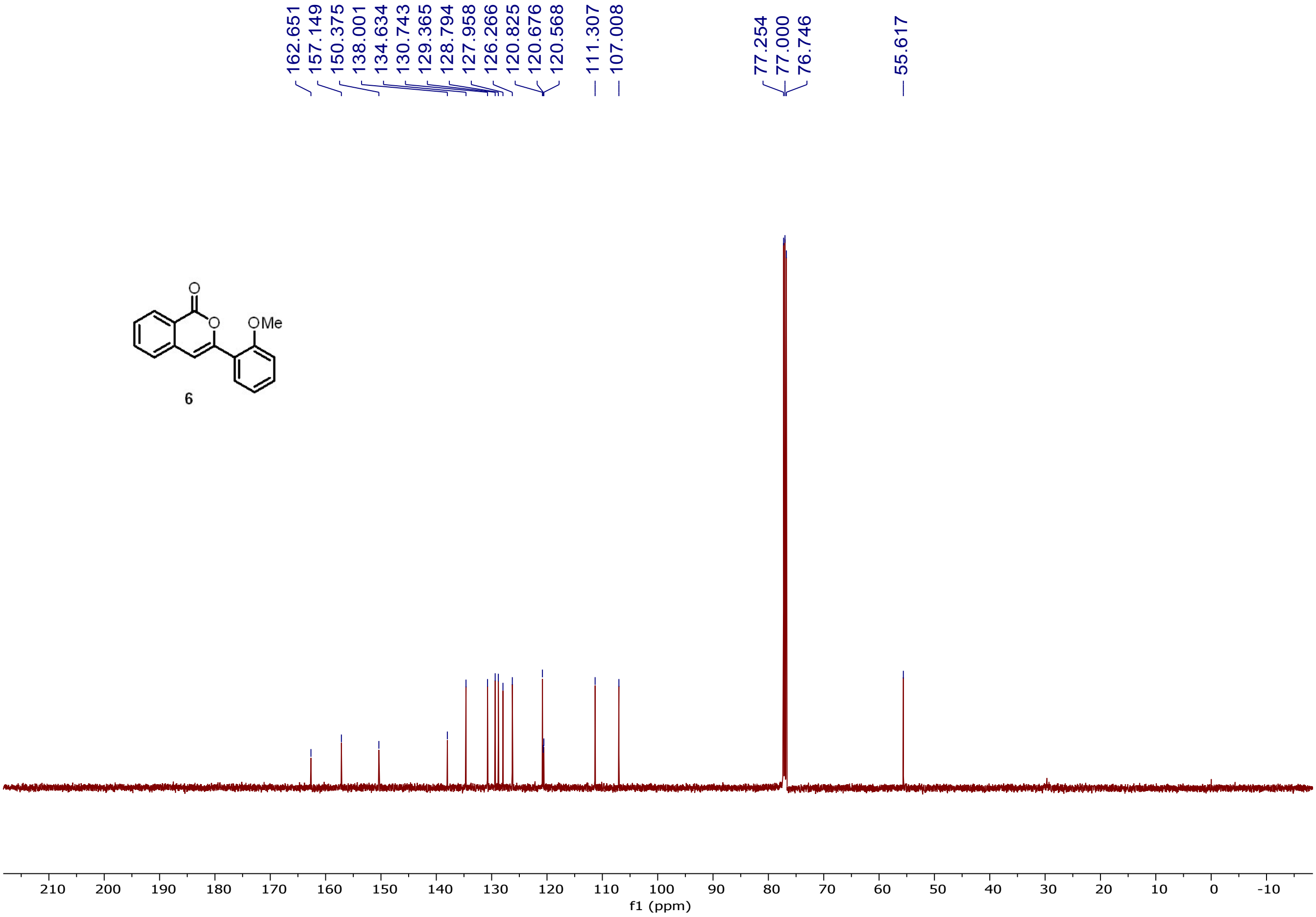
162.651 157.149 150.375 138.001 134.634 130.743 129.365 128.794 127.958 126.266 120.825 120.676 120.568
111.307 107.008
77.254 77.000 76.746
55.617
OMe
6
f1 (ppm)

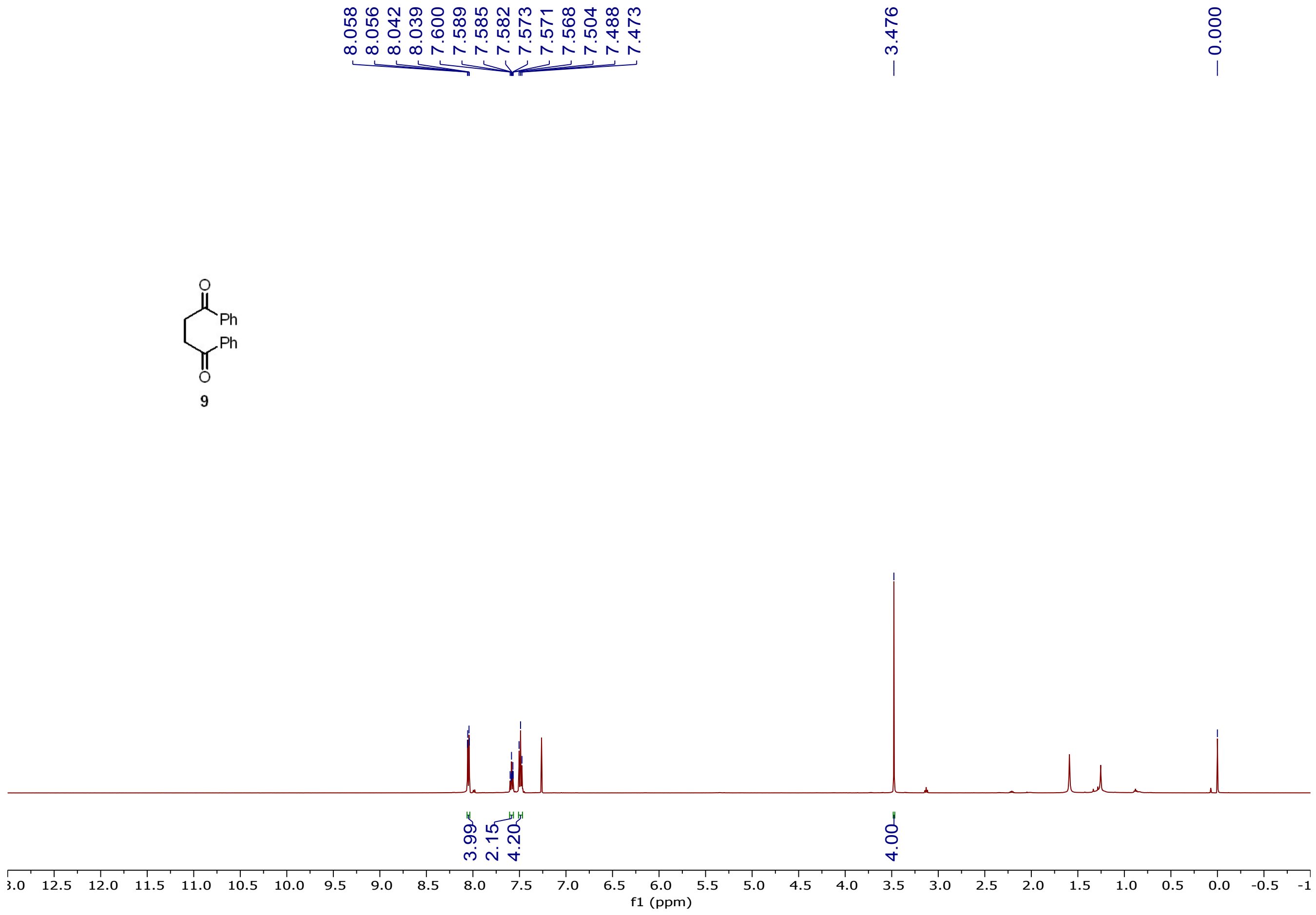
8.058
8.056
8.042
8.039
7.600
7.589
7.585
7.582
7.573
7.571
7.568
7.504
7.488
7.473
3.476
0.000
O
Ph
Ph
O
9
3.99
2.15
4.20
4.00
f1 (ppm)

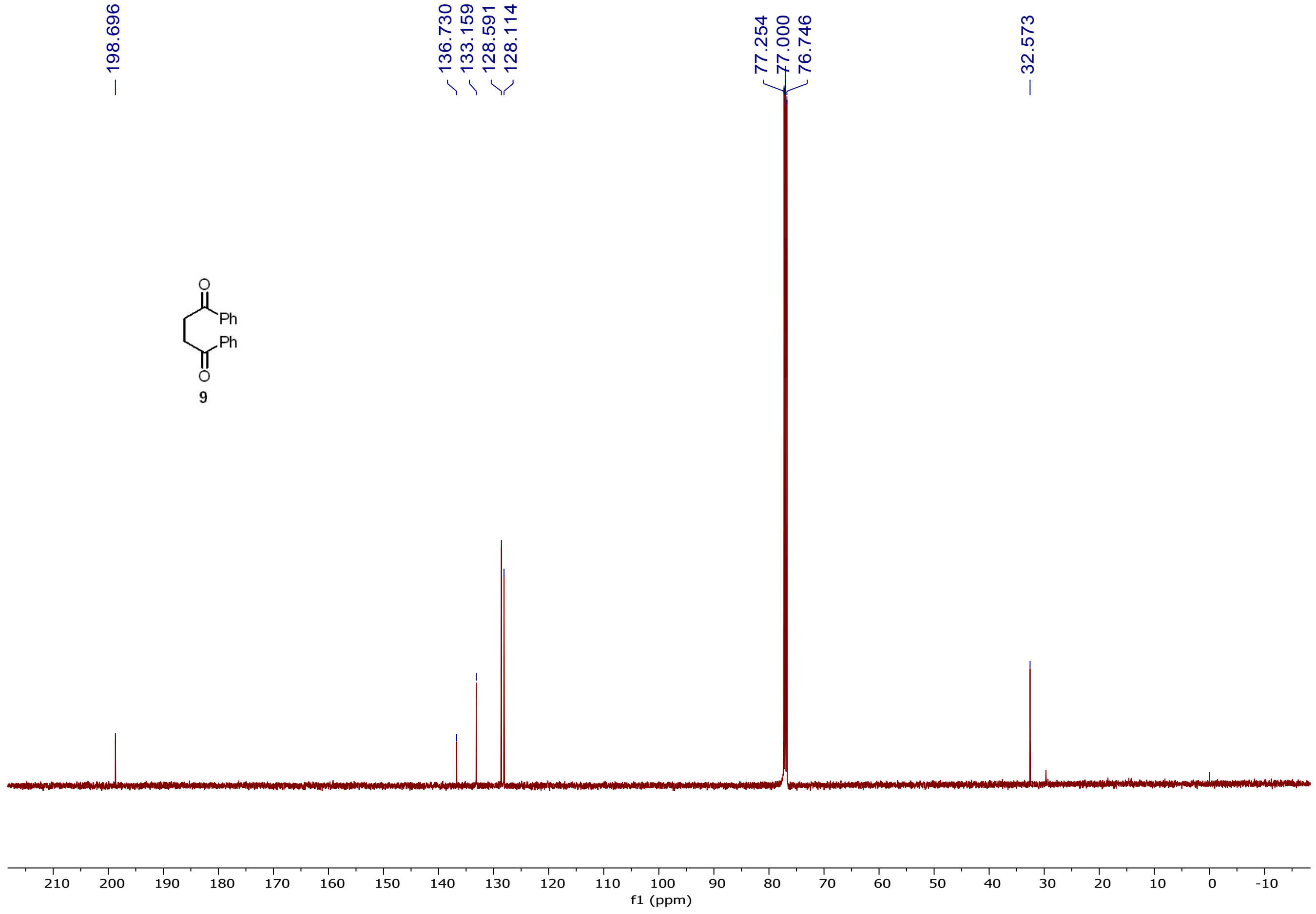
198.696
136.730
133.159
128.591
128.114
77.254
77.000
76.746
32.573
O
Ph
Ph
O
9
f1 (ppm)
